# Reply to “Do genome-scale models need exact solvers or clearer standards?”

**DOI:** 10.15252/msb.20156548

**Published:** 2015-10-14

**Authors:** Leonid Chindelevitch, Jason Trigg, Aviv Regev, Bonnie Berger

**Affiliations:** Massachusetts Institute of TechnologyCambridge, MA, USA

In their Correspondence entitled, “Do genome-scale models need exact solvers or clearer standards?”, Ebrahim *et al* (2015) suggest an unnecessary dichotomy. They discuss the findings of our paper, “An exact arithmetic toolbox for a consistent and reproducible structural analysis of metabolic network models” (Chindelevitch *et al*, 2014), and suggest that our work highlights the need for better model encoding standards. Moreover, the authors dispute our claims that multiple previously published metabolic network models are unable to produce growth when analyzed with an exact arithmetic approach. They attribute discrepancies between their findings and ours solely to a misinterpretation of the formatting conventions used to encode these models. The authors conclude that genome-scale metabolic network models need better standards, rather than the improvements in accuracy obtained with exact arithmetic. We argue here that improved standards and exact arithmetic are complementary advances that both benefit this field. Thus, the answer to the question posed by Ebrahim *et al* (2015) is “both.”

In this response, we acknowledge the discrepancies in model interpretation between our approach and that of Ebrahim *et al* (2015), but maintain the key conclusions of our original study. Namely, a number of published metabolic network models are unable to exhibit growth even when our interpretation of these models is identical to that of Ebrahim *et al* (2015). We attribute the remaining differences between the results of our original study and their study to significant changes made to the models since our results were initially published. Indeed, our MONGOOSE tool provides a model verification platform, which will continue to be useful in identifying errors in model functionality, helping curators to fix them. Additionally, we demonstrate on a specific real-model example that exact arithmetic can change the results of the analysis of genome-scale metabolic network models. We conclude that exact arithmetic remains an important tool for the verification and analysis of metabolic network models.

The original parser for models in SBML format used in our study interprets some boundary metabolites as subject to flux balance constraints; as the authors point out, this is contrary to the tacit convention in the field. However, we find blockage in many of the same genome-scale models, even when we interpret them according to this convention. Specifically, we initially reported that 16 out of 39 SBML models (the currently preferred format) had a blocked biomass reaction when only flux balance and irreversibility constraints are taken into consideration (Chindelevitch *et al*, 2014). After re-analysis with the parsing interpretations used by Ebrahim *et al* (2015), we find that 8 SBML published models still have a blocked biomass reaction under these conditions (Dataset EV1). Notably, all 10 highly curated SBML models reported in the BiGG database, which includes 3 models we previously reported as blocked, EC3 (iAF1260), HP2 (iIT341), and MT1 (iNJ661), exhibit flux through the biomass reaction after re-analysis (Dataset EV1). Combined with the 28 out of 50 non-SBML models (typically provided as an Excel spreadsheet) that we reported as blocked (Chindelevitch *et al*, 2014), a total of 36 out of 89 genome-scale models (rather than the original 44 out of 89) still exhibit the problem of having a blocked biomass reaction, which, as we already pointed out in the original paper, can be easily corrected in a systematic way by the MONGOOSE toolbox.

In order to understand the outstanding discrepancies between the two analyses of SBML models, we performed a comparison between the inputs used in our approach and that of Ebrahim *et al* (2015). We found that the original models we analyzed were modified in Ebrahim *et al* (2015) in one of two ways. First, the source files of many of the models have been altered—beyond the 9 models Ebrahim *et al* (2015) explicitly state to have modified in consultation with their creators, 10 other models appear to be different between the original versions we analyzed and those analyzed now by Ebrahim *et al* (2015). All model differences, as well as the scripts necessary to identify them, are included as Dataset EV2. Of the 8 SBML models that still have a blocked biomass reaction in our analysis, the source-file modifications account for four. Second, a number of models have been modified algorithmically during processing, as described in the worksheets provided by Ebrahim *et al* (2015), including 2 of the 8 SBML models we still find to have a blocked biomass reaction. Of the remaining two models, one is correctly identified as blocked, and the last one is absent from Ebrahim *et al*'s analysis (see Dataset EV3 for details). Note, that while there are also discrepancies in the analysis of non-SBML models (Dataset EV3), it is challenging to definitively compare those because their interpretation is not yet standardized.

Importantly, our comparison demonstrates that the correction type described by Ebrahim *et al* (2015), where a difference in capitalization was responsible for the creation of two constraints for the same metabolite, is the exception rather than the rule. The majority of the corrections we documented alter the underlying model more substantially, by removing (and sometimes introducing) constraint-generating metabolites, introducing (and sometimes removing) new reactions, or changing the irreversibility of model reactions, suggesting that the changes are far from cosmetic or typographical. Ebrahim *et al* (2015) count all models that underwent corrections as able to grow, as long as the corrections resolve any initial blockage of the biomass reaction. On the other hand, our analysis focuses on the models as they were published, and while the status of the growth reaction we report is the one after the modifications proposed by MONGOOSE during analysis, we count as blocked any model that was blocked before these modifications. We have been delighted to see that updated versions of many of these models have been posted after the release of our publication, and we are confident that all outstanding issues will be addressed by the community shortly.

The authors also claim that exact arithmetic is not necessary to ascertain the blockage status of metabolic network models, or, more broadly, of metabolic reactions in these models. This claim may hold for the specific solvers and models the authors have now posted, but cannot be generalized. For instance, we show (Dataset EV3) that one of the biomass reactions in the SC4 model (iIN800, *Saccharomyces cerevisiae*) is incorrectly predicted to be able to exhibit growth (nonzero flux) by 5 out of the 7 floating-point linear program solvers available on the NEOS server, even when we interpret the model in exactly the same way as Ebrahim *et al* (2015). A more detailed investigation of this phenomenon shows that even CPLEX, arguably the most reliable floating-point linear program solver available, falsely predicts growth for this model at its default settings (Dataset EV3) and recognizes its blockage only after significant tuning of its feasibility tolerance parameter. (Incidentally, the current version of the model analyzed by Ebrahim *et al* (2015) has been modified to use a different biomass reaction, which makes it appear to be blocked using both exact arithmetic as well as floating-point solvers.) This example is consistent with our originally reported results, illustrating the accuracy problems from which floating-point solvers can suffer when dealing with genome-scale metabolic network models. Although every model has some feasibility tolerance threshold below which floating-point solvers will get correct results, this threshold varies from model to model and is intractable to compute precisely.

While energy balance analysis is not the main focus of our paper, we point out that in practice we used a commonly accepted (Beard *et al*, 2002, 2004; Nigam & Liang, 2005; Yang *et al*, 2005) but stricter condition than the one used by some other approaches (Schellenberger *et al*, 2011) to identify energy-blocked reactions—those whose status changes from feasible to infeasible when energy balance, or “thermodynamic,” constraints are added. In particular, our original analysis looked for strongly feasible flux modes as opposed to weakly feasible flux modes, also called T-feasible in Beard *et al* (2014); thus, the implication arrow in equation (2) in Chindelevitch *et al* (2014), which implies that we are looking for weakly feasible modes, should have been bidirectional for consistency with our analysis in practice.

We are delighted that our work has spurred the models' authors to modify their models, as this is the goal of a model verification platform. The majority of our results, which apply to the models as they were published at the time of our analysis, as well as the main point of our paper, remain valid after re-analysis. While we agree that exact arithmetic is not always necessary to produce correct results, our point is a broader one: exact arithmetic is the only mathematically sound way to ensure that any model analysis is correct. No matter how good floating-point solvers are, there will always be models on which they break down. Moreover, their numerical sensitivity will only increase as these models scale. These facts are why we believe that exact arithmetic analysis, as implemented in our software MONGOOSE, provides a valuable service to the metabolic network modeling community. We are grateful to Ebrahim *et al* (2015) for raising the important issue of data formats and explicit versus implicit conventions. More broadly, our discordant analysis, the need for exact arithmetic, and the subtle yet critical data format conventions are part of the bigger question of data analysis, methods, and reproducibility in computational biology.

## Supporting Information

Dataset EV1

Dataset EV2

Dataset EV3
