# Supplementary material for: Reply to “Do genome-scale models need exact solvers or clearer standards?”
Source: Mol Syst Biol. 2015 Oct 14;11(10):830. doi: 10.15252/msb.20156548 (PMC4631201; doi:10.15252/msb.20156548)
Supplement: Supplementary file 1 — Dataset EV1 [file msb0011-0830-sd1.zip › Dataset1/Dataset1.docx]

This table contains the results of the re-analysis of all models in our original publication (Chindelevitch et al 2014) with the parsing conventions of Ebrahim et al. We mark models that underwent typo corrections or received a biomass export reaction in yellow, and those whose export reactions underwent compartment changes, in blue, consistently with our original publication.
