# Supplementary material for: Reply to “Do genome-scale models need exact solvers or clearer standards?”
Source: Mol Syst Biol. 2015 Oct 14;11(10):830. doi: 10.15252/msb.20156548 (PMC4631201; doi:10.15252/msb.20156548)
Supplement: Supplementary file 2 — Dataset EV2 [file msb0011-0830-sd2.zip › Dataset2/Dataset2.docx]

The table ModelFixes.xls summarizes the results of the included script, FileComparison.py, that identifies differences between the models used in our original analysis (listed [here](http://groups.csail.mit.edu/cb/mongoose/models.html)) and the fixed versions of these models (listed [here](https://github.com/aebrahim/m_model_collection)). For each item (metabolites, reactions or irreversible reactions), we list the number of these that appears only in the original model (first column) and the number that appears only in the fixed model (second column).

The included worksheet, ModelFixes.pyw, was used for computing these differences; note that in the case of SM2 the number of differences returned by the script is lower because some of the correspondences between non-matching metabolites and reactions can be identified by inspection. In the models with abbreviations in **bold**, the comparison was not fully carried out due to the vastly different number of metabolites. For 34 of the 78 models, no comparison could be performed at all because their source files we used for them in our original analysis were not in an SBML format, but rather in Excel or another format.

Removing metabolites leads to relaxation of the corresponding flux-balance constraints; adding reactions extends model functionality; and shifting reactions from irreversible to reversible leads to relaxation of the corresponding irreversibility constraints.
