# Supplementary material for: Reply to “Do genome-scale models need exact solvers or clearer standards?”
Source: Mol Syst Biol. 2015 Oct 14;11(10):830. doi: 10.15252/msb.20156548 (PMC4631201; doi:10.15252/msb.20156548)
Supplement: Supplementary file 3 — Dataset EV3 [file msb0011-0830-sd3.zip › msb0011-0830-sd3/Dataset3/Dataset3.docx]

**Example 1: demonstrating the failure of some floating-point solvers on iIN800**

The included script shows how to create a linear program file in MPS format. Note that this linear program is NOT feasible, i.e. the solvers should output the answer "infeasible". Running it through various linear program solvers available on the NEOS solvers produces the following results:

cbc, clp, scip, SYMPHONY, XpressMP find a feasible solution;

Gurobi and QSOpt_ex (the only exact linear program solver on NEOS) correctly find the problem infeasible;

MOSEK seems confused - it says the problem is feasible, but the primal and dual values it returns differ;

Proxy and feaspump are unable to return an answer because they expect problems with integral variables.

Submitting the problem to CPLEX results in getting a feasible solution with the default feasibility tolerance of 1e-6; it is not until the feasibility tolerance is lowered to 1e-8 that the infeasibility is detected.

**Example 2: the list of SBML models exhibiting topological blockage, with proof**

There are 4 topology-blocked models in SBML format in our analysis: AG1 (iMA871), AN1 (iHD666), PF1 (PlasmoNet) and SM2 (S_cœlicolor). Of these:

PF1 is correctly listed among the models that failed to produce biomass in the [newly created repository](https://github.com/aebrahim/m_model_collection);

SM2 has been fixed by removing a constraint, and AN1 by removing many constraints, as demonstrated in Dataset 2;

AG1 has been fixed by adding a biomass export reaction ([script](http://nbviewer.ipython.org/github/aebrahim/m_model_collection/blob/master/convert_models_to_mat.ipynb)).

The included worksheet identifies the chain of dead ends causing the topological blockage for all 4 models. In this case, a single metabolite; however, longer chains are sometimes encountered as well (see, for instance, the SBMLRecords3.pyw file).

**Example 3: the list of SBML models exhibiting stoichiometry blockage, with proof**

There is a single SBML model that is stoichiometry-blocked, and that is the AT1 (C4GEM) model. It is not part of the repository cited above.

The included worksheet provides the script that generates a linear program and its solution file. Together with the theoretical results in our paper (see Supplementary Note 2), they demonstrate that the biomass reaction in this model is indeed stoichiometry-blocked.

**Example 4: the list of SBML models exhibiting irreversibility blockage, with proof**

There are 4 irreversibility-blocked models in SBML format in our analysis: HS1 (Recon1), LL1 (iAO358), MG1 (iPS189) and RS1 (iRsp1095). Of these:

HS1 is not listed among the models in the repository cited above (the presence of a biomass reaction in it appears to be an error so we don't count it);

LL1 has been fixed by adding a reaction, and MG1 by adding a reaction and changing 5 irreversibility constraints, as demonstrated in Dataset 2;

RS1 has been fixed by opening the boundaries ([script](http://nbviewer.ipython.org/github/aebrahim/m_model_collection/blob/master/convert_models_to_mat.ipynb)).

The included worksheet provides the script that generates four linear program files, whose solutions are verified for correctness. Together with the theoretical results in our paper (see Supplementary Note 3), they demonstrate that the biomass reaction in each of these models is indeed irreversibility-blocked.

**Note about discrepancies between non-SBML models**

We discovered three further discrepancies between the two analyses with respect to the models that Ebrahim et al (2015) claim to have failed to produce any biomass. Two of these models, CG1 (iKK446) and CG2 (iYS277), we were able to get to work by correcting typos, and the discrepancy on the third one, BA1 (iGT196), may have been the result of Ebrahim et al. arbitrarily imposing a balance constraint on a dummy metabolite called “Nothing”. Unfortunately, it is challenging to compare models produced from non-SBML files directly because their interpretation is not yet standardized, so these conclusions are speculative. This is also why we are not able to ascertain whether the remaining differences with non-SBML files are due to modifications of the source files before or during processing, or floating-point error.
