# Supplementary material for: Reply to “Do genome-scale models need exact solvers or clearer standards?”
Source: Mol Syst Biol. 2015 Oct 14;11(10):830. doi: 10.15252/msb.20156548 (PMC4631201; doi:10.15252/msb.20156548)
Supplement: Supplementary file 3 — Dataset EV3 [file msb0011-0830-sd3.zip › msb0011-0830-sd3/Dataset3/Example1-NEOSsolvers/NEOS-cbc.pdf]

```
*************************************************************

   NEOS Server Version 5.0
   Job#     : 3432325
   Password : XIiJgcNW
   Solver   : milp:Cbc:MPS
   Start    : 2015-01-01 15:31:58
   End      : 2015-01-01 15:32:03
   Host     : NEOS HTCondor Pool

   Disclaimer:

   This information is provided without any express or
   implied warranty. In particular, there is no warranty
   of any kind concerning the fitness of this
   information  for any particular purpose.
*************************************************************
You are using the solver cbc-mps.

\%%%%%%%%%%%%%%%%%%%% CBC Results %%%%%%%%%%%%%%%%%%%%

Load Avg: ( 0.07 , 0.07 , 0.08 )
Welcome to the CBC MILP Solver
Version: 2.7.8
Build Date: Mar  4 2013
Revision Number: 1874

command line - /opt/neos/Solvers/release/coin-cbc/bin/cbc cbc.mps (default strategy 1)
At line 1 NAME          SC4cInfeasible
At line 2 ROWS
At line 1698 COLUMNS
At line 7132 RHS
At line 8827 BOUNDS
At line 9285 ENDATA
Problem SC4cInfeasible has 1694 rows, 1706 columns and 5432 elements
Coin0008I SC4cInfeasible read with 0 errors
Short match for time - completion: timeM(ode)
No match for 3600 - ? for list of commands
Presolve 126 (-1568) rows, 286 (-1420) columns and 1111 (-4321) elements
0  Obj 1 Primal inf 54.821786 (33) Dual inf 1542.1154 (111) w.o. free dual inf (74)
77  Obj 1 Primal inf 0.095877837 (5) Dual inf 62.03136 (34)
83  Obj 1
Optimal - objective value 1
After Postsolve, objective 1, infeasibilities - dual 0 (0), primal 0 (0)
Optimal objective 1 - 83 iterations time 0.012, Presolve 0.01
Total time (CPU seconds):       0.02   (Wallclock seconds):       0.02



%%%%%%%%%%%%%%%%%%%% CBC Results %%%%%%%%%%%%%%%%%%%%
```
