# Supplementary material for: Reply to “Do genome-scale models need exact solvers or clearer standards?”
Source: Mol Syst Biol. 2015 Oct 14;11(10):830. doi: 10.15252/msb.20156548 (PMC4631201; doi:10.15252/msb.20156548)
Supplement: Supplementary file 3 — Dataset EV3 [file msb0011-0830-sd3.zip › msb0011-0830-sd3/Dataset3/Example1-NEOSsolvers/NEOS-scip.pdf]

```
*************************************************************

   NEOS Server Version 5.0
   Job#     : 3432320
   Password : LOkQYgiI
   Solver   : go:scip:MPS
   Start    : 2015-01-01 15:13:14
   End      : 2015-01-01 15:13:23
   Host     : thales.la.asu.edu

   Disclaimer:

   This information is provided without any express or
   implied warranty. In particular, there is no warranty
   of any kind concerning the fitness of this
   information  for any particular purpose.
*************************************************************
SCIP version 3.1.1 [precision: 8 byte] [memory: block] [mode: optimized] [LP solver: CPLEX 12.6.1.0] [GitHash: bade511]
Copyright (c) 2002-2014 Konrad-Zuse-Zentrum fuer Informationstechnik Berlin (ZIB)

External codes:
  CPLEX 12.6.1.0         Linear Programming Solver developed by IBM (www.cplex.com)
  cppad-20140000.1       Algorithmic Differentiation of C++ algorithms developed by B. Bell (www.coin-or.org/CppAD)
  ZLIB 1.2.8             General purpose compression library by J. Gailly and M. Adler (zlib.net)
  GMP 5.1.3              GNU Multiple Precision Arithmetic Library developed by T. Granlund (gmplib.org)
  ZIMPL 3.3.2            Zuse Institute Mathematical Programming Language developed by T. Koch (zimpl.zib.de)
  Ipopt 3.11.9           Interior Point Optimizer developed by A. Waechter et.al. (www.coin-or.org/Ipopt)

reading user parameter file <scip.set>

SCIP> file <sample.lp> not found
SCIP> file <sample.zpl> not found
SCIP>
read problem <sample.mps>
============

original problem has 1706 variables (0 bin, 0 int, 0 impl, 1706 cont) and 1694 constraints
SCIP> file <sample.osil> not found
SCIP> limits/time = 36000
SCIP> loaded parameter file <scip.set>
SCIP>
presolving:
(round 1) 539 del vars, 1268 del conss, 0 add conss, 380 chg bounds, 0 chg sides, 0 chg coeffs, 0 upgd conss, 0 impls, 0 clqs
(round 2) 647 del vars, 1306 del conss, 0 add conss, 413 chg bounds, 0 chg sides, 0 chg coeffs, 0 upgd conss, 0 impls, 0 clqs
(round 3) 661 del vars, 1310 del conss, 0 add conss, 416 chg bounds, 0 chg sides, 0 chg coeffs, 0 upgd conss, 0 impls, 0 clqs
(round 4) 661 del vars, 1315 del conss, 0 add conss, 416 chg bounds, 0 chg sides, 0 chg coeffs, 0 upgd conss, 0 impls, 0 clqs
(round 5) 889 del vars, 1315 del conss, 0 add conss, 416 chg bounds, 0 chg sides, 0 chg coeffs, 0 upgd conss, 0 impls, 0 clqs
(round 6) 924 del vars, 1350 del conss, 0 add conss, 420 chg bounds, 0 chg sides, 0 chg coeffs, 0 upgd conss, 0 impls, 0 clqs
(round 7) 924 del vars, 1350 del conss, 0 add conss, 421 chg bounds, 0 chg sides, 0 chg coeffs, 0 upgd conss, 0 impls, 0 clqs
(round 8) 931 del vars, 1350 del conss, 0 add conss, 421 chg bounds, 0 chg sides, 0 chg coeffs, 0 upgd conss, 0 impls, 0 clqs
presolving (9 rounds):
 931 deleted vars, 1350 deleted constraints, 0 added constraints, 421 tightened bounds, 0 added holes, 0 changed sides, 0 changed coefficients
 0 implications, 0 cliques
presolved problem has 775 variables (0 bin, 0 int, 0 impl, 775 cont) and 344 constraints
    344 constraints of type <linear>
transformed objective value is always integral (scale: 1)
Presolving Time: 0.01

 time | node  | left  |LP iter|LP it/n| mem |mdpt |frac |vars |cons |cols |rows |cuts |confs|strbr|  dualbound   | primalbound  |  gap
* 0.0s|     1 |     0 |    22 |     - |4734k|   0 |   - | 775 | 344 | 775 | 344 |   0 |   0 |   0 | 1.000000e+00 | 1.000000e+00 |   0.00%
  0.0s|     1 |     0 |    22 |     - |4734k|   0 |   - | 775 | 344 | 775 | 344 |   0 |   0 |   0 | 1.000000e+00 | 1.000000e+00 |   0.00%

SCIP Status        : problem is solved [optimal solution found]
Solving Time (sec) : 0.02
Solving Nodes      : 1
Primal Bound       : +1.00000000000000e+00 (1 solutions)
Dual Bound         : +1.00000000000000e+00
Gap                : 0.00 %

SCIP>
written solution information to file <results1>

SCIP>
written statistics to file <results2>

SCIP> solution status: optimal solution found
objective value:                                    1
C0000004                                   0.192767   (obj:0)
C0000010                                   0.000417   (obj:0)
C0000014                                   0.000417   (obj:0)
C0000025                                   0.192767   (obj:0)
C0000027                                   0.192767   (obj:0)
```

C0000036	6.6e-05	(obj:0)
C0000038	0.191891967589996	(obj:0)
C0000044	0.000417	(obj:0)
C0000047	0.0256139999999998	(obj:0)
C0000048	0.096481	(obj:0)
C0000049	0.25728	(obj:0)
C0000050	0.451054	(obj:0)
C0000051	0.19653	(obj:0)
C0000052	0.35734	(obj:0)
C0000053	0.17152	(obj:0)
C0000054	0.318	(obj:0)
C0000055	0.075041	(obj:0)
C0000056	0.17152	(obj:0)
C0000057	0.25014	(obj:0)
C0000058	-0.14274	(obj:0)
C0000059	0.11435	(obj:0)
C0000062	0.17152	(obj:0)
C0000072	0.13579	(obj:0)
C0000087	0.000812	(obj:0)
C0000115	6.6e-05	(obj:0)
C0000116	0.000351	(obj:0)
C0000117	0.006415	(obj:0)
C0000118	0.183512	(obj:0)
C0000119	3.2e-05	(obj:0)
C0000120	5.6e-05	(obj:0)
C0000121	-0.183383	(obj:0)
C0000122	9.6e-05	(obj:0)
C0000124	0.000125	(obj:0)
C0000125	0.007306	(obj:0)
C0000126	0.042879983795	(obj:0)
C0000131	0.028	(obj:0)
C0000165	0.113413	(obj:0)
C0000169	0.006525	(obj:0)
C0000180	0.00416	(obj:0)
C0000182	0.00309	(obj:0)
C0000201	6.6e-05	(obj:0)
C0000202	0.000351	(obj:0)
C0000220	-0.002432	(obj:0)
C0000230	0.000781	(obj:0)
C0000240	0.132413	(obj:0)
C0000247	0.82099	(obj:0)
C0000248	0.000781	(obj:0)
C0000270	0.000417	(obj:0)
C0000299	0.183497	(obj:0)
C0000318	0.00030974237	(obj:0)
C0000320	0.00013433945	(obj:0)
C0000321	0.000134128785	(obj:0)
C0000324	0.00291629715	(obj:0)
C0000376	0.000608189855	(obj:0)
C0000377	0.00980321475	(obj:0)
C0000378	0.0026235895	(obj:0)
C0000379	9.2481935e-05	(obj:0)
C0000380	0.000417	(obj:0)
C0000382	62.73124587036	(obj:0)
C0000389	0.0538490000000002	(obj:0)
C0000397	-1.62049999964783e-08	(obj:0)
C0000440	0.117	(obj:0)
C0000454	0.12864	(obj:0)
C0000461	0.23942	(obj:0)
C0000476	0.192386	(obj:0)
C0000478	-0.000206	(obj:0)
C0000515	1.1358	(obj:0)
C0000518	0.51852	(obj:0)
C0000522	-0.002432	(obj:0)
C0000523	0.00284900000000016	(obj:0)
C0000556	0.0538490000000002	(obj:0)
C0000642	0.000417	(obj:0)
C0000655	0.002365	(obj:0)
C0000656	0.002365	(obj:0)
C0000668	2.670556951385	(obj:0)
C0000669	6.6e-05	(obj:0)
C0000670	0.000351	(obj:0)
C0000690	0.000417	(obj:0)
C0000695	0.000417	(obj:0)
C0000706	0.000417	(obj:0)
C0000726	0.192386	(obj:0)
C0000732	-2.461429	(obj:0)
C0000746	0.0545869837949955	(obj:0)
C0000788	0.000206	(obj:0)
C0000805	0.00309	(obj:0)
C0000806	0.00309	(obj:0)
C0000837	-1.656685	(obj:0)
C0000842	-1.65432	(obj:0)
C0000843	0.192767	(obj:0)
C0000849	-2.02308401620501	(obj:0)
C0000853	0.002365	(obj:0)
C0000860	-0.821407	(obj:0)
C0000863	0.82099	(obj:0)
C0000871	0.117	(obj:0)
C0000874	-0.117	(obj:0)
C0000879	-0.192767	(obj:0)
C0000883	0.016205	(obj:0)
C0000913	0.170849	(obj:0)
C0000918	0.821407	(obj:0)
C0000920	0.003787	(obj:0)
C0000953	-0.014612	(obj:0)
C0000956	0.002432	(obj:0)
C0000959	0.003587	(obj:0)

C0000960	0.014612	(obj:0)
C0000964	0.192767	(obj:0)
C0000967	0.192767	(obj:0)
C0000969	6.6e-05	(obj:0)
C0000971	0.007306	(obj:0)
C0000974	0.821407	(obj:0)
C0000985	0.192767	(obj:0)
C0000987	0.007306	(obj:0)
C0001017	0.000417	(obj:0)
C0001054	0.007306	(obj:0)
C0001055	0.007306	(obj:0)
C0001059	-0.007306	(obj:0)
C0001073	0.006019	(obj:0)
C0001075	0.000417	(obj:0)
C0001082	0.000417	(obj:0)
C0001088	6.60000000000105e-05	(obj:0)
C0001101	-0.192767	(obj:0)
C0001102	0.192767	(obj:0)
C0001103	-0.192767	(obj:0)
C0001104	0.192767	(obj:0)
C0001136	0.002432	(obj:0)
C0001141	-0.192386	(obj:0)
C0001146	0.117	(obj:0)
C0001154	0.023371	(obj:0)
C0001166	-0.043475016205	(obj:0)
C0001171	0.119432	(obj:0)
C0001177	-0.003587	(obj:0)
C0001191	-0.192767	(obj:0)
C0001194	6.6e-05	(obj:0)
C0001198	0.00030974237	(obj:0)
C0001199	9.2481935e-05	(obj:0)
C0001200	0.00291629715	(obj:0)
C0001202	0.00980321475	(obj:0)
C0001203	0.000608189855	(obj:0)
C0001204	0.0026235895	(obj:0)
C0001208	-6.6e-05	(obj:0)
C0001210	0.0545869837949955	(obj:0)
C0001211	-0.113413	(obj:0)
C0001213	0.117	(obj:0)
C0001214	2.46348	(obj:0)
C0001220	0.000483	(obj:0)
C0001221	-0.004203983795	(obj:0)
C0001244	2.46348	(obj:0)
C0001247	0.192767	(obj:0)
C0001291	0.192767	(obj:0)
C0001292	-1.62049999964783e-08	(obj:0)
C0001293	-1.62049999964783e-08	(obj:0)
C0001294	-1.62049999964783e-08	(obj:0)
C0001296	-1.62049999964783e-08	(obj:0)
C0001309	0.00013433945	(obj:0)
C0001310	0.000134128785	(obj:0)
C0001311	0.000417	(obj:0)
C0001319	0.000417	(obj:0)
C0001331	0.05	(obj:0)
C0001336	0.05	(obj:0)
C0001378	-1.62049999964783e-08	(obj:0)
C0001395	0.007306	(obj:0)
C0001396	0.02	(obj:0)
C0001398	1.65432	(obj:0)
C0001419	1.70432	(obj:0)
C0001420	-0.043475016205	(obj:0)
C0001424	0.113413	(obj:0)
C0001425	0.00284900000000016	(obj:0)
C0001426	0.821407	(obj:0)
C0001439	5.6e-05	(obj:0)
C0001441	0.000417	(obj:0)
C0001444	0.192386	(obj:0)
C0001446	0.35734	(obj:0)
C0001450	0.13579	(obj:0)
C0001451	0.17152	(obj:0)
C0001452	0.17152	(obj:0)
C0001457	0.00013433945	(obj:0)
C0001458	0.000134128785	(obj:0)
C0001459	0.00030974237	(obj:0)
C0001460	9.2481935e-05	(obj:0)
C0001461	0.00291629715	(obj:0)
C0001462	0.00980321475	(obj:0)
C0001463	0.000608189855	(obj:0)
C0001464	0.0026235895	(obj:0)
C0001465	0.000417	(obj:0)
C0001471	0.042879983795	(obj:0)
C0001482	0.119432	(obj:0)
C0001483	9.6e-05	(obj:0)
C0001484	0.000125	(obj:0)
C0001486	0.006415	(obj:0)
C0001487	6.6e-05	(obj:0)
C0001493	2.46348	(obj:0)
C0001499	0.318	(obj:0)
C0001501	0.0256139999999998	(obj:0)
C0001503	0.132413	(obj:0)
C0001504	0.0538490000000002	(obj:0)
C0001508	1.44454995138499	(obj:0)
C0001509	0.075041	(obj:0)
C0001511	0.17152	(obj:0)
C0001515	3.2e-05	(obj:0)
C0001516	0.25014	(obj:0)
C0001517	0.23942	(obj:0)
C0001531	0.000483	(obj:0)

```
C0001535                          0.0545869837949955   (obj:0)
C0001537                                     0.11435   (obj:0)
C0001541                                     0.12864   (obj:0)
C0001547                                    0.192767   (obj:0)
C0001548                                    0.451054   (obj:0)
C0001549                                        0.02   (obj:0)
C0001556                                     0.19653   (obj:0)
C0001557                                       0.117   (obj:0)
C0001558                                    0.023371   (obj:0)
C0001559                                       0.028   (obj:0)
C0001560                                    0.096481   (obj:0)
C0001564                                     0.25728   (obj:0)
C0001569                                    0.183512   (obj:0)
C0001571                                    6.6e-05    (obj:0)
C0001586                                           1   (obj:0)
C0001601                              0.004203983795   (obj:0)
C0001611                                    0.113413   (obj:0)
C0001619                                    0.183383   (obj:0)
C0001636                                    0.000206   (obj:0)
C0001641                                    0.192767   (obj:0)
C0001652                                     0.14274   (obj:0)
C0001660                                    2.461429   (obj:0)
C0001670                            2.02308401620501   (obj:0)
C0001671                        1.62049999964783e-08   (obj:0)
C0001701                                           1   (obj:0)
C0001702                                           1   (obj:1)
C0001705                                           1   (obj:0)
SCIP Status        : problem is solved [optimal solution found]
Total Time         :       0.03
  solving          :       0.02
  presolving       :       0.01 (included in solving)
  reading          :       0.01
  copying          :       0.00 (0 times copied the problem)
Original Problem   :
  Problem name     : SC4cInfeasible
  Variables        : 1706 (0 binary, 0 integer, 0 implicit integer, 1706 continuous)
  Constraints      : 1694 initial, 1694 maximal
  Objective sense  : minimize
Presolved Problem  :
  Problem name     : t_SC4cInfeasible
  Variables        : 775 (0 binary, 0 integer, 0 implicit integer, 775 continuous)
  Constraints      : 344 initial, 344 maximal
Presolvers         :   ExecTime  SetupTime  Calls  FixedVars   AggrVars   ChgTypes  ChgBounds   AddHoles    DelCons    AddCons   ChgSides   Ch
  boundshift       :       0.00       0.00      0          0          0          0          0          0          0          0          0
  components       :       0.00       0.00      1          0          0          0          0          0          0          0          0
  convertinttobin  :       0.00       0.00      0          0          0          0          0          0          0          0          0
  domcol           :       0.00       0.00      3        235          0          0          0          0          0          0          0
  dualinfer        :       0.00       0.00      0          0          0          0          0          0          0          0          0
  gateextraction   :       0.00       0.00      0          0          0          0          0          0          0          0          0
  implics          :       0.00       0.00      9          0          0          0          0          0          0          0          0
  inttobinary      :       0.00       0.00      0          0          0          0          0          0          0          0          0
  trivial          :       0.00       0.00      9          0          0          0          0          0          0          0          0
  dualfix          :       0.00       0.00      9         73          0          0          0          0          0          0          0
  genvbounds       :       0.00       0.00      0          0          0          0          0          0          0          0          0
  probing          :       0.00       0.00      0          0          0          0          0          0          0          0          0
  pseudoobj        :       0.00       0.00      0          0          0          0          0          0          0          0          0
  linear           :       0.01       0.00      4        259        364          0        421          0       1350          0          0
  root node        :          -          -      -          0          -          -          0          -          -          -          -
Constraints        :     Number  MaxNumber  #Separate #Propagate    #EnfoLP    #EnfoPS     #Check   #ResProp    Cutoffs    DomReds       Cuts
  integral         :          0          0          0          0          0          0          5          0          0          0          0
  linear           :        344        344          0          1          0          0          4          0          0          0          0
  countsols        :          0          0          0          0          0          0          2          0          0          0          0
Constraint Timings :  TotalTime  SetupTime   Separate  Propagate     EnfoLP     EnfoPS      Check    ResProp    SB-Prop
  integral         :       0.00       0.00       0.00       0.00       0.00       0.00       0.00       0.00       0.00
  linear           :       0.00       0.00       0.00       0.00       0.00       0.00       0.00       0.00       0.00
  countsols        :       0.00       0.00       0.00       0.00       0.00       0.00       0.00       0.00       0.00
Propagators        : #Propagate   #ResProp    Cutoffs    DomReds
  dualfix          :          1          0          0          0
  genvbounds       :          0          0          0          0
  obbt             :          0          0          0          0
  probing          :          0          0          0          0
  pseudoobj        :          0          0          0          0
  redcost          :          0          0          0          0
  rootredcost      :          0          0          0          0
  vbounds          :          1          0          0          0
Propagator Timings :  TotalTime  SetupTime   Presolve  Propagate    ResProp    SB-Prop
  dualfix          :       0.00       0.00       0.00       0.00       0.00       0.00
  genvbounds       :       0.00       0.00       0.00       0.00       0.00       0.00
  obbt             :       0.00       0.00       0.00       0.00       0.00       0.00
  probing          :       0.00       0.00       0.00       0.00       0.00       0.00
  pseudoobj        :       0.00       0.00       0.00       0.00       0.00       0.00
  redcost          :       0.00       0.00       0.00       0.00       0.00       0.00
  rootredcost      :       0.00       0.00       0.00       0.00       0.00       0.00
  vbounds          :       0.00       0.00       0.00       0.00       0.00       0.00
Conflict Analysis  :       Time      Calls    Success    DomReds  Conflicts   Literals    Reconvs ReconvLits   LP Iters
  propagation      :       0.00          0          0          -          0        0.0          0        0.0          -
  infeasible LP    :       0.00          0          0          -          0        0.0          0        0.0          0
  bound exceed. LP :       0.00          0          0          -          0        0.0          0        0.0          0
  strong branching :       0.00          0          0          -          0        0.0          0        0.0          0
  pseudo solution  :       0.00          1          0          -          0        0.0          0        0.0          -
  applied globally :       0.00          -          -          0          0        0.0          -          -          -
  applied locally  :          -          -          -          0          0        0.0          -          -          -
Separators         :   ExecTime  SetupTime      Calls    Cutoffs    DomReds       Cuts    Applied      Conss
  cut pool         :       0.00                     0          -          -          0          -          -    (maximal pool size: 0)
  cgmip            :       0.00       0.00          0          0          0          0          0          0
  clique           :       0.00       0.00          0          0          0          0          0          0
  closecuts        :       0.00       0.00          0          0          0          0          0          0
  cmir             :       0.00       0.00          0          0          0          0          0          0
```

```
  flowcover        :       0.00       0.00          0          0          0          0          0          0
  gomory           :       0.00       0.00          0          0          0          0          0          0
  impliedbounds    :       0.00       0.00          0          0          0          0          0          0
  intobj           :       0.00       0.00          0          0          0          0          0          0
  mcf              :       0.00       0.00          0          0          0          0          0          0
  oddcycle         :       0.00       0.00          0          0          0          0          0          0
  rapidlearning    :       0.00       0.00          0          0          0          0          0          0
  strongcg         :       0.00       0.00          0          0          0          0          0          0
  zerohalf         :       0.00       0.00          0          0          0          0          0          0
Pricers            :   ExecTime  SetupTime      Calls       Vars
  problem variables:       0.00          -          0          0
Branching Rules    :   ExecTime  SetupTime   BranchLP  BranchExt   BranchPS    Cutoffs    DomReds       Cuts      Conss   Children
  allfullstrong    :       0.00       0.00          0          0          0          0          0          0          0          0
  cloud            :       0.00       0.00          0          0          0          0          0          0          0          0
  fullstrong       :       0.00       0.00          0          0          0          0          0          0          0          0
  inference        :       0.00       0.00          0          0          0          0          0          0          0          0
  leastinf         :       0.00       0.00          0          0          0          0          0          0          0          0
  mostinf          :       0.00       0.00          0          0          0          0          0          0          0          0
  pscost           :       0.00       0.00          0          0          0          0          0          0          0          0
  random           :       0.00       0.00          0          0          0          0          0          0          0          0
  relpscost        :       0.00       0.00          0          0          0          0          0          0          0          0
Primal Heuristics  :   ExecTime  SetupTime      Calls      Found
  LP solutions     :       0.00          -          -          1
  pseudo solutions :       0.00          -          -          0
  strong branching :       0.00          -          -          0
  actconsdiving    :       0.00       0.00          0          0
  clique           :       0.00       0.00          0          0
  coefdiving       :       0.00       0.00          0          0
  crossover        :       0.00       0.00          0          0
  dins             :       0.00       0.00          0          0
  dualval          :       0.00       0.00          0          0
  feaspump         :       0.00       0.00          0          0
  fixandinfer      :       0.00       0.00          0          0
  fracdiving       :       0.00       0.00          0          0
  guideddiving     :       0.00       0.00          0          0
  intdiving        :       0.00       0.00          0          0
  intshifting      :       0.00       0.00          0          0
  linesearchdiving :       0.00       0.00          0          0
  localbranching   :       0.00       0.00          0          0
  mutation         :       0.00       0.00          0          0
  nlpdiving        :       0.00       0.00          0          0
  objpscostdiving  :       0.00       0.00          0          0
  octane           :       0.00       0.00          0          0
  oneopt           :       0.00       0.00          0          0
  proximity        :       0.00       0.00          0          0
  pscostdiving     :       0.00       0.00          0          0
  randrounding     :       0.00       0.00          0          0
  rens             :       0.00       0.00          0          0
  rins             :       0.00       0.00          0          0
  rootsoldiving    :       0.00       0.00          0          0
  rounding         :       0.00       0.00          0          0
  shiftandpropagate:       0.00       0.00          0          0
  shifting         :       0.00       0.00          0          0
  simplerounding   :       0.00       0.00          0          0
  subnlp           :       0.00       0.00          0          0
  trivial          :       0.00       0.00          2          0
  trysol           :       0.00       0.00          0          0
  twoopt           :       0.00       0.00          0          0
  undercover       :       0.00       0.00          0          0
  vbounds          :       0.00       0.00          0          0
  veclendiving     :       0.00       0.00          0          0
  zeroobj          :       0.00       0.00          0          0
  zirounding       :       0.00       0.00          0          0
  other solutions  :          -          -          -          0
LP                 :       Time      Calls Iterations  Iter/call   Iter/sec  Time-0-It Calls-0-It
  primal LP        :       0.00          0          0       0.00          -       0.00          0
  dual LP          :       0.00          1         22      22.00          -       0.00          0
  lex dual LP      :       0.00          0          0       0.00          -
  barrier LP       :       0.00          0          0       0.00          -       0.00          0
  diving/probing LP:       0.00          0          0       0.00          -
  strong branching :       0.00          0          0       0.00          -
    (at root node) :          -          0          0       0.00          -
  conflict analysis:       0.00          0          0       0.00          -
B&B Tree           :
  number of runs   :          1
  nodes            :          1 (0 internal, 1 leaves)
  nodes (total)    :          1 (0 internal, 1 leaves)
  nodes left       :          0
  max depth        :          0
  max depth (total):          0
  backtracks       :          0 (0.0%)
  delayed cutoffs  :          0
  repropagations   :          0 (0 domain reductions, 0 cutoffs)
  avg switch length:       2.00
  switching time   :       0.00
Root Node          :
  First LP value   : +1.00000000000000e+00
  First LP Iters   :         22 (2200.00 Iter/sec)
  First LP Time    :       0.01
  Final Dual Bound : +1.00000000000000e+00
  Final Root Iters :         22
Solution           :
  Solutions found  :          1 (1 improvements)
  First Solution   : +1.00000000000000e+00   (in run 1, after 1 nodes, 0.02 seconds, depth 0, found by <relaxation>)
  Gap First Sol.   :       0.00 %
  Gap Last Sol.    :       0.00 %
  Primal Bound     : +1.00000000000000e+00   (in run 1, after 1 nodes, 0.02 seconds, depth 0, found by <relaxation>)
  Dual Bound       : +1.00000000000000e+00
```

```
Gap               :       0.00 %
Avg. Gap          :     100.00 % (2.00 primal-dual integral)
```

Home
