# Supplementary material for: Reply to “Do genome-scale models need exact solvers or clearer standards?”
Source: Mol Syst Biol. 2015 Oct 14;11(10):830. doi: 10.15252/msb.20156548 (PMC4631201; doi:10.15252/msb.20156548)
Supplement: Supplementary file 3 — Dataset EV3 [file msb0011-0830-sd3.zip › msb0011-0830-sd3/Dataset3/Example1-NEOSsolvers/CPLEX.pdf]

```
Last login: Thu Jan  1 19:23:55 on ttys001
Leonids-MacBook-Pro:trunk leonidc$ cd ../../../../../Applications/IBM/ILOG/CPLEX_Studio1251/cplex/bin/x86-64_osx/
-bash: cd: ../../../../../Applications/IBM/ILOG/CPLEX_Studio1251/cplex/bin/x86-64_osx/: No such file or directory
Leonids-MacBook-Pro:trunk leonidc$ cd MetaMerge/
Leonids-MacBook-Pro:MetaMerge leonidc$ cd ../../../../../Applications/IBM/ILOG/CPLEX_Studio1251/cplex/bin/x86-64_osx/
Leonids-MacBook-Pro:x86-64_osx leonidc$ ./cplex

Welcome to IBM(R) ILOG(R) CPLEX(R) Interactive Optimizer 12.5.1.0
  with Simplex, Mixed Integer & Barrier Optimizers
5725-A06 5725-A29 5724-Y48 5724-Y49 5724-Y54 5724-Y55 5655-Y21
Copyright IBM Corp. 1988, 2013.  All Rights Reserved.

Type 'help' for a list of available commands.
Type 'help' followed by a command name for more
information on commands.

CPLEX> read SC4cInfeasible.mps
Selected objective sense:  MINIMIZE
Selected objective  name:  OBJ
Selected RHS        name:  RHS
Selected bound      name:  B0000000
Problem 'SC4cInfeasible.mps' read.
Read time = 0.00 sec. (0.48 ticks)
CPLEX> opt
Tried aggregator 1 time.
LP Presolve eliminated 1330 rows and 1309 columns.
Aggregator did 313 substitutions.
Reduced LP has 51 rows, 84 columns, and 312 nonzeros.
Presolve time = 0.00 sec. (1.17 ticks)

Iteration log . . .
Iteration:     1   Dual objective     =             1.000000

Dual simplex - Optimal:  Objective =  1.0000000000e+00
Solution time =    0.00 sec.  Iterations = 18 (0)
Deterministic time = 1.86 ticks  (478.85 ticks/sec)

CPLEX> set sim tol feas 1e-7
New value for feasibility tolerance: 1e-07
CPLEX> opt
Using devex.

Primal simplex - Optimal:  Objective =  1.0000000000e+00
Solution time =    0.00 sec.  Iterations = 0 (0)
Deterministic time = 0.22 ticks  (223.85 ticks/sec)

CPLEX> set sim tol feas 2e-8
New value for feasibility tolerance: 2e-08
CPLEX> opt
Using devex.

Primal simplex - Optimal:  Objective =  1.0000000000e+00
Solution time =    0.00 sec.  Iterations = 0 (0)
Deterministic time = 0.21 ticks  (211.72 ticks/sec)

CPLEX> set sim tol feas 1e-8
New value for feasibility tolerance: 1e-08
CPLEX> opt

Iteration log . . .
Iteration:     1   Dual objective     =             1.000000

Dual simplex - Infeasible:  Objective =  1.0000000000e+00
Solution time =    0.00 sec.  Iterations = 13 (0)
Infeasible variable = C0000788
Deterministic time = 0.79 ticks  (683.91 ticks/sec)

CPLEX>
```
