# Supplementary material for: Reply to “Do genome-scale models need exact solvers or clearer standards?”
Source: Mol Syst Biol. 2015 Oct 14;11(10):830. doi: 10.15252/msb.20156548 (PMC4631201; doi:10.15252/msb.20156548)
Supplement: Supplementary file 3 — Dataset EV3 [file msb0011-0830-sd3.zip › msb0011-0830-sd3/Dataset3/Example1-NEOSsolvers/NEOS-XpressMP.pdf]

```
*************************************************************

   NEOS Server Version 5.0
   Job#     : 3432323
   Password : PJKhStsT
   Solver   : lp:XpressMP:MPS
   Start    : 2015-01-01 15:29:40
   End      : 2015-01-01 15:29:46
   Host     : NEOS HTCondor Pool

   Disclaimer:

   This information is provided without any express or
   implied warranty. In particular, there is no warranty
   of any kind concerning the fitness of this
   information  for any particular purpose.
*************************************************************

## YOUR PARAMETER FILE ################################
MAXTIME=1400
READPROB
MINIM
GLOBAL
WRITEPRTSOL
QUIT


#######################################################
Load Avg: ( 0.11 , 0.08 , 0.09 )
Starting solver XPRESS-INTEGER
Executing on neos-3.neos-server.org

## OUTPUT TO SCREEN ################################
FICO Xpress Optimizer 64-bit v25.01.05 (Hyper64 capacity)
(c) Copyright Fair Isaac Corporation 1983-2013. All rights reserved
Reading Problem SC4cInfeasible
Problem Statistics
        1695 (      0 spare) rows
        1706 (      0 spare) structural columns
        5433 (      0 spare) non-zero elements
Global Statistics
           0 entities          0 sets         0 set members
Minimizing LP SC4cInfeasible
Original problem has:
      1695 rows          1706 cols         5433 elements
Presolved problem has:
        29 rows            84 cols          197 elements
Crash basis containing 12 structural columns created

   Its         Obj Value      S   Ninf  Nneg   Sum Dual Inf  Time
     0          1.000000      D      1     0        .000000     0
    14          1.000000      D      0     0        .000000     0
Uncrunching matrix
    14          1.000000      D      0     0        .000000     0
Optimal solution found
```

?1055 Error: Can not resume global search - not currently solving a MIP.
###

SOLUTION OUTPUT

Problem Statistics
Matrix SC4cInfeasible
Objective OBJ

RHS RHS
Problem has   1695 rows and   1706 structural columns

Solution Statistics
Minimization performed
Optimal solution found after   14 iterations
Objective function value is   1.000000

Rows Section

	Number	Row	At	Value	Slack Value	Dual Value	RHS
N	1	OBJ	BS	1.000000	-1.000000	.000000	.000000
E	2	R0000000	EQ	.000000	.000000	.000000	.000000
E	3	R0000001	EQ	.000000	.000000	.000000	.000000
E	4	R0000002	EQ	.000000	.000000	.000000	.000000
E	5	R0000003	EQ	.000000	.000000	.000000	.000000
E	6	R0000004	EQ	.000000	.000000	.000000	.000000
E	7	R0000005	EQ	.000000	.000000	.000000	.000000
E	8	R0000006	EQ	.000000	.000000	.000000	.000000
E	9	R0000007	EQ	.000000	.000000	.000000	.000000
E	10	R0000008	EQ	.000000	.000000	.000000	.000000
E	11	R0000009	EQ	.000000	.000000	.000000	.000000
E	12	R0000010	EQ	.000000	.000000	.000000	.000000
E	13	R0000011	EQ	.000000	.000000	.000000	.000000
E	14	R0000012	EQ	.000000	.000000	.000000	.000000
E	15	R0000013	EQ	.000000	.000000	.000000	.000000
E	16	R0000014	EQ	.000000	.000000	.000000	.000000
E	17	R0000015	EQ	.000000	.000000	.000000	.000000
E	18	R0000016	EQ	.000000	.000000	.000000	.000000
E	19	R0000017	EQ	.000000	.000000	.000000	.000000
E	20	R0000018	EQ	.000000	.000000	.000000	.000000
E	21	R0000019	EQ	.000000	.000000	.000000	.000000
	Number	Row	At	Value	Slack Value	Dual Value	RHS
E	22	R0000020	EQ	.000000	.000000	.000000	.000000
E	23	R0000021	EQ	.000000	.000000	.000000	.000000
E	24	R0000022	EQ	.000000	.000000	.000000	.000000
E	25	R0000023	EQ	.000000	.000000	.000000	.000000
E	26	R0000024	EQ	.000000	.000000	.000000	.000000
E	27	R0000025	EQ	.000000	.000000	.000000	.000000
E	28	R0000026	EQ	.000000	.000000	.000000	.000000
E	29	R0000027	EQ	.000000	.000000	.000000	.000000
E	30	R0000028	EQ	.000000	.000000	.000000	.000000
E	31	R0000029	EQ	.000000	.000000	.000000	.000000
E	32	R0000030	EQ	.000000	.000000	.000000	.000000
E	33	R0000031	EQ	.000000	.000000	.000000	.000000
E	34	R0000032	EQ	.000000	.000000	.000000	.000000
E	35	R0000033	EQ	.000000	.000000	.000000	.000000
E	36	R0000034	EQ	.000000	.000000	.000000	.000000
E	37	R0000035	EQ	.000000	.000000	.000000	.000000
E	38	R0000036	EQ	.000000	.000000	.000000	.000000
E	39	R0000037	EQ	.000000	.000000	.000000	.000000
E	40	R0000038	EQ	.000000	.000000	.000000	.000000
E	41	R0000039	EQ	.000000	.000000	.000000	.000000
E	42	R0000040	EQ	.000000	.000000	.000000	.000000
	Number	Row	At	Value	Slack Value	Dual Value	RHS
E	43	R0000041	EQ	.000000	.000000	.000000	.000000
E	44	R0000042	EQ	.000000	.000000	.000000	.000000
E	45	R0000043	EQ	.000000	.000000	.000000	.000000
E	46	R0000044	EQ	.000000	.000000	.000000	.000000
E	47	R0000045	EQ	.000000	.000000	.000000	.000000
E	48	R0000046	EQ	.000000	.000000	.000000	.000000
E	49	R0000047	EQ	.000000	.000000	.000000	.000000

E	50	R0000048	EQ	.000000	.000000	.000000	.000000
E	51	R0000049	EQ	.000000	.000000	.000000	.000000
E	52	R0000050	EQ	.000000	.000000	.000000	.000000
E	53	R0000051	EQ	.000000	.000000	.000000	.000000
E	54	R0000052	EQ	.000000	.000000	.000000	.000000
E	55	R0000053	EQ	.000000	.000000	.000000	.000000
E	56	R0000054	EQ	.000000	.000000	.000000	.000000
E	57	R0000055	EQ	.000000	.000000	.000000	.000000
E	58	R0000056	EQ	.000000	.000000	.000000	.000000
E	59	R0000057	EQ	.000000	.000000	.000000	.000000
E	60	R0000058	EQ	.000000	.000000	.000000	.000000
E	61	R0000059	EQ	.000000	.000000	.000000	.000000
E	62	R0000060	EQ	.000000	.000000	.000000	.000000
E	63	R0000061	EQ	.000000	.000000	.000000	.000000
	Number	Row	At	Value	Slack Value	Dual Value	RHS
E	64	R0000062	EQ	.000000	.000000	.000000	.000000
E	65	R0000063	EQ	.000000	.000000	.000000	.000000
E	66	R0000064	EQ	.000000	.000000	.000000	.000000
E	67	R0000065	EQ	.000000	.000000	.000000	.000000
E	68	R0000066	EQ	.000000	.000000	.000000	.000000
E	69	R0000067	EQ	.000000	.000000	.000000	.000000
E	70	R0000068	EQ	.000000	.000000	.000000	.000000
E	71	R0000069	EQ	.000000	.000000	.000000	.000000
E	72	R0000070	EQ	.000000	.000000	.000000	.000000
E	73	R0000071	EQ	.000000	.000000	.000000	.000000
E	74	R0000072	EQ	.000000	.000000	.000000	.000000
E	75	R0000073	EQ	.000000	.000000	.000000	.000000
E	76	R0000074	EQ	.000000	.000000	.000000	.000000
E	77	R0000075	EQ	.000000	.000000	.000000	.000000
E	78	R0000076	EQ	.000000	.000000	.000000	.000000
E	79	R0000077	EQ	.000000	.000000	.000000	.000000
E	80	R0000078	EQ	.000000	.000000	.000000	.000000
E	81	R0000079	EQ	.000000	.000000	.000000	.000000
E	82	R0000080	EQ	.000000	.000000	.000000	.000000
E	83	R0000081	EQ	.000000	.000000	.000000	.000000
E	84	R0000082	EQ	.000000	.000000	.000000	.000000
	Number	Row	At	Value	Slack Value	Dual Value	RHS
E	85	R0000083	EQ	.000000	.000000	.000000	.000000
E	86	R0000084	EQ	.000000	.000000	.000000	.000000
E	87	R0000085	EQ	.000000	.000000	.000000	.000000
E	88	R0000086	EQ	.000000	.000000	.000000	.000000
E	89	R0000087	EQ	.000000	.000000	.000000	.000000
E	90	R0000088	EQ	.000000	.000000	.000000	.000000
E	91	R0000089	EQ	.000000	.000000	.000000	.000000
E	92	R0000090	EQ	.000000	.000000	.000000	.000000
E	93	R0000091	EQ	.000000	.000000	.000000	.000000
E	94	R0000092	EQ	.000000	.000000	.000000	.000000
E	95	R0000093	EQ	.000000	.000000	.000000	.000000
E	96	R0000094	EQ	.000000	.000000	.000000	.000000
E	97	R0000095	EQ	.000000	.000000	.000000	.000000
E	98	R0000096	EQ	.000000	.000000	.000000	.000000
E	99	R0000097	EQ	.000000	.000000	.000000	.000000
E	100	R0000098	EQ	.000000	.000000	.000000	.000000
E	101	R0000099	EQ	.000000	.000000	.000000	.000000
E	102	R0000100	EQ	.000000	.000000	.000000	.000000
E	103	R0000101	EQ	.000000	.000000	.000000	.000000
E	104	R0000102	EQ	.000000	.000000	.000000	.000000
E	105	R0000103	EQ	.000000	.000000	.000000	.000000
	Number	Row	At	Value	Slack Value	Dual Value	RHS
E	106	R0000104	EQ	.000000	.000000	.000000	.000000
E	107	R0000105	EQ	.000000	.000000	.000000	.000000
E	108	R0000106	EQ	.000000	.000000	.000000	.000000
E	109	R0000107	EQ	.000000	.000000	.000000	.000000
E	110	R0000108	EQ	.000000	.000000	.000000	.000000
E	111	R0000109	EQ	.000000	.000000	.000000	.000000
E	112	R0000110	EQ	.000000	.000000	.000000	.000000
E	113	R0000111	EQ	.000000	.000000	.000000	.000000
E	114	R0000112	EQ	.000000	.000000	.000000	.000000
E	115	R0000113	EQ	.000000	.000000	.000000	.000000
E	116	R0000114	EQ	.000000	.000000	.000000	.000000
E	117	R0000115	EQ	.000000	.000000	.000000	.000000

E	118	R0000116	EQ	.000000	.000000	.000000	.000000
E	119	R0000117	EQ	.000000	.000000	.000000	.000000
E	120	R0000118	EQ	.000000	.000000	.000000	.000000
E	121	R0000119	EQ	.000000	.000000	.000000	.000000
E	122	R0000120	EQ	.000000	.000000	.000000	.000000
E	123	R0000121	EQ	.000000	.000000	.000000	.000000
E	124	R0000122	EQ	.000000	.000000	.000000	.000000
E	125	R0000123	EQ	.000000	.000000	.000000	.000000
E	126	R0000124	EQ	.000000	.000000	.000000	.000000
	Number	Row	At	Value	Slack Value	Dual Value	RHS
E	127	R0000125	EQ	.000000	.000000	.000000	.000000
E	128	R0000126	EQ	.000000	.000000	.000000	.000000
E	129	R0000127	EQ	.000000	.000000	.000000	.000000
E	130	R0000128	EQ	.000000	.000000	.000000	.000000
E	131	R0000129	EQ	.000000	.000000	.000000	.000000
E	132	R0000130	EQ	.000000	.000000	.000000	.000000
E	133	R0000131	EQ	.000000	.000000	.000000	.000000
E	134	R0000132	EQ	.000000	.000000	.000000	.000000
E	135	R0000133	EQ	.000000	.000000	.000000	.000000
E	136	R0000134	EQ	.000000	.000000	.000000	.000000
E	137	R0000135	EQ	.000000	.000000	.000000	.000000
E	138	R0000136	EQ	.000000	.000000	.000000	.000000
E	139	R0000137	EQ	.000000	.000000	.000000	.000000
E	140	R0000138	EQ	.000000	.000000	.000000	.000000
E	141	R0000139	EQ	.000000	.000000	.000000	.000000
E	142	R0000140	EQ	.000000	.000000	.000000	.000000
E	143	R0000141	EQ	.000000	.000000	.000000	.000000
E	144	R0000142	EQ	.000000	.000000	.000000	.000000
E	145	R0000143	EQ	.000000	.000000	.000000	.000000
E	146	R0000144	EQ	.000000	.000000	.000000	.000000
E	147	R0000145	EQ	.000000	.000000	.000000	.000000
	Number	Row	At	Value	Slack Value	Dual Value	RHS
E	148	R0000146	EQ	.000000	.000000	.000000	.000000
E	149	R0000147	EQ	.000000	.000000	.000000	.000000
E	150	R0000148	EQ	.000000	.000000	.000000	.000000
E	151	R0000149	EQ	.000000	.000000	.000000	.000000
E	152	R0000150	EQ	.000000	.000000	.000000	.000000
E	153	R0000151	EQ	.000000	.000000	.000000	.000000
E	154	R0000152	EQ	.000000	.000000	.000000	.000000
E	155	R0000153	EQ	.000000	.000000	.000000	.000000
E	156	R0000154	EQ	.000000	.000000	.000000	.000000
E	157	R0000155	EQ	.000000	.000000	.000000	.000000
E	158	R0000156	EQ	.000000	.000000	.000000	.000000
E	159	R0000157	EQ	.000000	.000000	.000000	.000000
E	160	R0000158	EQ	.000000	.000000	.000000	.000000
E	161	R0000159	EQ	.000000	.000000	.000000	.000000
E	162	R0000160	EQ	.000000	.000000	.000000	.000000
E	163	R0000161	EQ	.000000	.000000	.000000	.000000
E	164	R0000162	EQ	.000000	.000000	.000000	.000000
E	165	R0000163	EQ	.000000	.000000	.000000	.000000
E	166	R0000164	EQ	.000000	.000000	.000000	.000000
E	167	R0000165	EQ	.000000	.000000	.000000	.000000
E	168	R0000166	EQ	.000000	.000000	.000000	.000000
	Number	Row	At	Value	Slack Value	Dual Value	RHS
E	169	R0000167	EQ	.000000	.000000	.000000	.000000
E	170	R0000168	EQ	.000000	.000000	.000000	.000000
E	171	R0000169	EQ	.000000	.000000	.000000	.000000
E	172	R0000170	EQ	.000000	.000000	.000000	.000000
E	173	R0000171	EQ	.000000	.000000	.000000	.000000
E	174	R0000172	EQ	.000000	.000000	.000000	.000000
E	175	R0000173	EQ	.000000	.000000	.000000	.000000
E	176	R0000174	EQ	.000000	.000000	.000000	.000000
E	177	R0000175	EQ	.000000	.000000	.000000	.000000
E	178	R0000176	EQ	.000000	.000000	.000000	.000000
E	179	R0000177	EQ	.000000	.000000	.000000	.000000
E	180	R0000178	EQ	.000000	.000000	.000000	.000000
E	181	R0000179	EQ	.000000	.000000	.000000	.000000
E	182	R0000180	EQ	.000000	.000000	.000000	.000000
E	183	R0000181	EQ	.000000	.000000	.000000	.000000
E	184	R0000182	EQ	.000000	.000000	.000000	.000000
E	185	R0000183	EQ	.000000	.000000	.000000	.000000

E	186	R0000184	EQ	.000000	.000000	.000000	.000000
E	187	R0000185	EQ	.000000	.000000	.000000	.000000
E	188	R0000186	EQ	.000000	.000000	.000000	.000000
E	189	R0000187	EQ	.000000	.000000	.000000	.000000
	Number	Row	At	Value	Slack Value	Dual Value	RHS
E	190	R0000188	EQ	.000000	.000000	.000000	.000000
E	191	R0000189	EQ	.000000	.000000	.000000	.000000
E	192	R0000190	EQ	.000000	.000000	.000000	.000000
E	193	R0000191	EQ	.000000	.000000	.000000	.000000
E	194	R0000192	EQ	.000000	.000000	.000000	.000000
E	195	R0000193	EQ	.000000	.000000	.000000	.000000
E	196	R0000194	EQ	.000000	.000000	.000000	.000000
E	197	R0000195	EQ	.000000	.000000	.000000	.000000
E	198	R0000196	EQ	.000000	.000000	.000000	.000000
E	199	R0000197	EQ	.000000	.000000	.000000	.000000
E	200	R0000198	EQ	.000000	.000000	.000000	.000000
E	201	R0000199	EQ	.000000	.000000	.000000	.000000
E	202	R0000200	EQ	.000000	.000000	.000000	.000000
E	203	R0000201	EQ	.000000	.000000	.000000	.000000
E	204	R0000202	EQ	.000000	.000000	.000000	.000000
E	205	R0000203	EQ	.000000	.000000	.000000	.000000
E	206	R0000204	EQ	.000000	.000000	.000000	.000000
E	207	R0000205	EQ	.000000	.000000	.000000	.000000
E	208	R0000206	EQ	.000000	.000000	.000000	.000000
E	209	R0000207	EQ	.000000	.000000	.000000	.000000
E	210	R0000208	EQ	.000000	.000000	.000000	.000000
	Number	Row	At	Value	Slack Value	Dual Value	RHS
E	211	R0000209	EQ	.000000	.000000	.000000	.000000
E	212	R0000210	EQ	.000000	.000000	.000000	.000000
E	213	R0000211	EQ	.000000	.000000	.000000	.000000
E	214	R0000212	EQ	.000000	.000000	.000000	.000000
E	215	R0000213	EQ	.000000	.000000	.000000	.000000
E	216	R0000214	EQ	.000000	.000000	.000000	.000000
E	217	R0000215	EQ	.000000	.000000	.000000	.000000
E	218	R0000216	EQ	.000000	.000000	.000000	.000000
E	219	R0000217	EQ	.000000	.000000	.000000	.000000
E	220	R0000218	EQ	.000000	.000000	.000000	.000000
E	221	R0000219	EQ	.000000	.000000	.000000	.000000
E	222	R0000220	EQ	.000000	.000000	.000000	.000000
E	223	R0000221	EQ	.000000	.000000	.000000	.000000
E	224	R0000222	EQ	.000000	.000000	.000000	.000000
E	225	R0000223	EQ	.000000	.000000	.000000	.000000
E	226	R0000224	EQ	.000000	.000000	.000000	.000000
E	227	R0000225	EQ	.000000	.000000	.000000	.000000
E	228	R0000226	EQ	.000000	.000000	.000000	.000000
E	229	R0000227	EQ	.000000	.000000	.000000	.000000
E	230	R0000228	EQ	.000000	.000000	.000000	.000000
E	231	R0000229	EQ	.000000	.000000	.000000	.000000
	Number	Row	At	Value	Slack Value	Dual Value	RHS
E	232	R0000230	EQ	.000000	.000000	.000000	.000000
E	233	R0000231	EQ	.000000	.000000	.000000	.000000
E	234	R0000232	EQ	.000000	.000000	.000000	.000000
E	235	R0000233	EQ	.000000	.000000	.000000	.000000
E	236	R0000234	EQ	.000000	.000000	.000000	.000000
E	237	R0000235	EQ	.000000	.000000	.000000	.000000
E	238	R0000236	EQ	.000000	.000000	.000000	.000000
E	239	R0000237	EQ	.000000	.000000	.000000	.000000
E	240	R0000238	EQ	.000000	.000000	.000000	.000000
E	241	R0000239	EQ	.000000	.000000	.000000	.000000
E	242	R0000240	EQ	.000000	.000000	.000000	.000000
E	243	R0000241	EQ	.000000	.000000	.000000	.000000
E	244	R0000242	EQ	.000000	.000000	.000000	.000000
E	245	R0000243	EQ	.000000	.000000	.000000	.000000
E	246	R0000244	EQ	.000000	.000000	.000000	.000000
E	247	R0000245	EQ	.000000	.000000	.000000	.000000
E	248	R0000246	EQ	.000000	.000000	.000000	.000000
E	249	R0000247	EQ	.000000	.000000	.000000	.000000
E	250	R0000248	EQ	.000000	.000000	.000000	.000000
E	251	R0000249	EQ	.000000	.000000	.000000	.000000
E	252	R0000250	EQ	.000000	.000000	.000000	.000000
	Number	Row	At	Value	Slack Value	Dual Value	RHS

E	253	R0000251	EQ	.000000	.000000	.000000	.000000
E	254	R0000252	EQ	.000000	.000000	.000000	.000000
E	255	R0000253	EQ	.000000	.000000	.000000	.000000
E	256	R0000254	EQ	.000000	.000000	.000000	.000000
E	257	R0000255	EQ	.000000	.000000	.000000	.000000
E	258	R0000256	EQ	.000000	.000000	.000000	.000000
E	259	R0000257	EQ	.000000	.000000	.000000	.000000
E	260	R0000258	EQ	.000000	.000000	.000000	.000000
E	261	R0000259	EQ	.000000	.000000	.000000	.000000
E	262	R0000260	EQ	.000000	.000000	.000000	.000000
E	263	R0000261	EQ	.000000	.000000	.000000	.000000
E	264	R0000262	EQ	.000000	.000000	.000000	.000000
E	265	R0000263	EQ	.000000	.000000	.000000	.000000
E	266	R0000264	EQ	.000000	.000000	.000000	.000000
E	267	R0000265	EQ	.000000	.000000	.000000	.000000
E	268	R0000266	EQ	.000000	.000000	.000000	.000000
E	269	R0000267	EQ	.000000	.000000	.000000	.000000
E	270	R0000268	EQ	.000000	.000000	.000000	.000000
E	271	R0000269	EQ	.000000	.000000	.000000	.000000
E	272	R0000270	EQ	.000000	.000000	.000000	.000000
E	273	R0000271	EQ	.000000	.000000	.000000	.000000
	Number	Row	At	Value	Slack Value	Dual Value	RHS
E	274	R0000272	EQ	.000000	.000000	.000000	.000000
E	275	R0000273	EQ	.000000	.000000	.000000	.000000
E	276	R0000274	EQ	.000000	.000000	.000000	.000000
E	277	R0000275	EQ	.000000	.000000	.000000	.000000
E	278	R0000276	EQ	.000000	.000000	.000000	.000000
E	279	R0000277	EQ	.000000	.000000	.000000	.000000
E	280	R0000278	EQ	.000000	.000000	.000000	.000000
E	281	R0000279	EQ	.000000	.000000	.000000	.000000
E	282	R0000280	EQ	.000000	.000000	.000000	.000000
E	283	R0000281	EQ	.000000	.000000	.000000	.000000
E	284	R0000282	EQ	.000000	.000000	.000000	.000000
E	285	R0000283	EQ	.000000	.000000	.000000	.000000
E	286	R0000284	EQ	.000000	.000000	.000000	.000000
E	287	R0000285	EQ	.000000	.000000	.000000	.000000
E	288	R0000286	EQ	.000000	.000000	.000000	.000000
E	289	R0000287	EQ	.000000	.000000	.000000	.000000
E	290	R0000288	EQ	.000000	.000000	.000000	.000000
E	291	R0000289	EQ	.000000	.000000	.000000	.000000
E	292	R0000290	EQ	.000000	.000000	.000000	.000000
E	293	R0000291	EQ	.000000	.000000	.000000	.000000
E	294	R0000292	EQ	.000000	.000000	.000000	.000000
	Number	Row	At	Value	Slack Value	Dual Value	RHS
E	295	R0000293	EQ	.000000	.000000	.000000	.000000
E	296	R0000294	EQ	.000000	.000000	.000000	.000000
E	297	R0000295	EQ	.000000	.000000	.000000	.000000
E	298	R0000296	EQ	.000000	.000000	.000000	.000000
E	299	R0000297	EQ	.000000	.000000	.000000	.000000
E	300	R0000298	EQ	.000000	.000000	.000000	.000000
E	301	R0000299	EQ	.000000	.000000	.000000	.000000
E	302	R0000300	EQ	.000000	.000000	.000000	.000000
E	303	R0000301	EQ	.000000	.000000	.000000	.000000
E	304	R0000302	EQ	.000000	.000000	.000000	.000000
E	305	R0000303	EQ	.000000	.000000	.000000	.000000
E	306	R0000304	EQ	.000000	.000000	.000000	.000000
E	307	R0000305	EQ	.000000	.000000	.000000	.000000
E	308	R0000306	EQ	.000000	.000000	.000000	.000000
E	309	R0000307	EQ	.000000	.000000	.000000	.000000
E	310	R0000308	EQ	.000000	.000000	.000000	.000000
E	311	R0000309	EQ	.000000	.000000	.000000	.000000
E	312	R0000310	EQ	.000000	.000000	.000000	.000000
E	313	R0000311	EQ	.000000	.000000	.000000	.000000
E	314	R0000312	EQ	.000000	.000000	.000000	.000000
E	315	R0000313	EQ	.000000	.000000	.000000	.000000
	Number	Row	At	Value	Slack Value	Dual Value	RHS
E	316	R0000314	EQ	.000000	.000000	.000000	.000000
E	317	R0000315	EQ	.000000	.000000	.000000	.000000
E	318	R0000316	EQ	.000000	.000000	.000000	.000000
E	319	R0000317	EQ	.000000	.000000	.000000	.000000
E	320	R0000318	EQ	.000000	.000000	.000000	.000000

E	321	R0000319	EQ	.000000	.000000	.000000	.000000
E	322	R0000320	EQ	.000000	.000000	.000000	.000000
E	323	R0000321	EQ	.000000	.000000	.000000	.000000
E	324	R0000322	EQ	.000000	.000000	.000000	.000000
E	325	R0000323	EQ	.000000	.000000	.000000	.000000
E	326	R0000324	EQ	.000000	.000000	.000000	.000000
E	327	R0000325	EQ	.000000	.000000	.000000	.000000
E	328	R0000326	EQ	.000000	.000000	.000000	.000000
E	329	R0000327	EQ	.000000	.000000	.000000	.000000
E	330	R0000328	EQ	.000000	.000000	.000000	.000000
E	331	R0000329	EQ	.000000	.000000	.000000	.000000
E	332	R0000330	EQ	.000000	.000000	.000000	.000000
E	333	R0000331	EQ	.000000	.000000	.000000	.000000
E	334	R0000332	EQ	.000000	.000000	.000000	.000000
E	335	R0000333	EQ	.000000	.000000	.000000	.000000
E	336	R0000334	EQ	.000000	.000000	.000000	.000000
	Number	Row	At	Value	Slack Value	Dual Value	RHS
E	337	R0000335	EQ	.000000	.000000	.000000	.000000
E	338	R0000336	EQ	.000000	.000000	.000000	.000000
E	339	R0000337	EQ	.000000	.000000	.000000	.000000
E	340	R0000338	EQ	.000000	.000000	.000000	.000000
E	341	R0000339	EQ	.000000	.000000	.000000	.000000
E	342	R0000340	EQ	.000000	.000000	.000000	.000000
E	343	R0000341	EQ	.000000	.000000	.000000	.000000
E	344	R0000342	EQ	.000000	.000000	.000000	.000000
E	345	R0000343	EQ	.000000	.000000	.000000	.000000
E	346	R0000344	EQ	.000000	.000000	.000000	.000000
E	347	R0000345	EQ	.000000	.000000	.000000	.000000
E	348	R0000346	EQ	.000000	.000000	.000000	.000000
E	349	R0000347	EQ	.000000	.000000	.000000	.000000
E	350	R0000348	EQ	.000000	.000000	.000000	.000000
E	351	R0000349	EQ	.000000	.000000	.000000	.000000
E	352	R0000350	EQ	.000000	.000000	.000000	.000000
E	353	R0000351	EQ	.000000	.000000	.000000	.000000
E	354	R0000352	EQ	.000000	.000000	.000000	.000000
E	355	R0000353	EQ	.000000	.000000	.000000	.000000
E	356	R0000354	EQ	.000000	.000000	.000000	.000000
E	357	R0000355	EQ	.000000	.000000	.000000	.000000
	Number	Row	At	Value	Slack Value	Dual Value	RHS
E	358	R0000356	EQ	.000000	.000000	.000000	.000000
E	359	R0000357	EQ	.000000	.000000	.000000	.000000
E	360	R0000358	EQ	.000000	.000000	.000000	.000000
E	361	R0000359	EQ	.000000	.000000	.000000	.000000
E	362	R0000360	EQ	.000000	.000000	.000000	.000000
E	363	R0000361	EQ	.000000	.000000	.000000	.000000
E	364	R0000362	EQ	.000000	.000000	.000000	.000000
E	365	R0000363	EQ	.000000	.000000	.000000	.000000
E	366	R0000364	EQ	.000000	.000000	.000000	.000000
E	367	R0000365	EQ	.000000	.000000	.000000	.000000
E	368	R0000366	EQ	.000000	.000000	.000000	.000000
E	369	R0000367	EQ	.000000	.000000	.000000	.000000
E	370	R0000368	EQ	.000000	.000000	.000000	.000000
E	371	R0000369	EQ	.000000	.000000	.000000	.000000
E	372	R0000370	EQ	.000000	.000000	.000000	.000000
E	373	R0000371	EQ	.000000	.000000	.000000	.000000
E	374	R0000372	EQ	.000000	.000000	.000000	.000000
E	375	R0000373	EQ	.000000	.000000	.000000	.000000
E	376	R0000374	EQ	.000000	.000000	.000000	.000000
E	377	R0000375	EQ	.000000	.000000	.000000	.000000
E	378	R0000376	EQ	.000000	.000000	.000000	.000000
	Number	Row	At	Value	Slack Value	Dual Value	RHS
E	379	R0000377	EQ	.000000	.000000	.000000	.000000
E	380	R0000378	EQ	.000000	.000000	.000000	.000000
E	381	R0000379	EQ	.000000	.000000	.000000	.000000
E	382	R0000380	EQ	.000000	.000000	.000000	.000000
E	383	R0000381	EQ	.000000	.000000	.000000	.000000
E	384	R0000382	EQ	.000000	.000000	.000000	.000000
E	385	R0000383	EQ	.000000	.000000	.000000	.000000
E	386	R0000384	EQ	.000000	.000000	.000000	.000000
E	387	R0000385	EQ	.000000	.000000	.000000	.000000
E	388	R0000386	EQ	.000000	.000000	.000000	.000000

E	389	R0000387	EQ	.000000	.000000	.000000	.000000
E	390	R0000388	EQ	.000000	.000000	.000000	.000000
E	391	R0000389	EQ	.000000	.000000	.000000	.000000
E	392	R0000390	EQ	.000000	.000000	.000000	.000000
E	393	R0000391	EQ	.000000	.000000	.000000	.000000
E	394	R0000392	EQ	.000000	.000000	.000000	.000000
E	395	R0000393	EQ	.000000	.000000	.000000	.000000
E	396	R0000394	EQ	.000000	.000000	.000000	.000000
E	397	R0000395	EQ	.000000	.000000	.000000	.000000
E	398	R0000396	EQ	.000000	.000000	.000000	.000000
E	399	R0000397	EQ	.000000	.000000	.000000	.000000
	Number	Row	At	Value	Slack Value	Dual Value	RHS
E	400	R0000398	EQ	.000000	.000000	.000000	.000000
E	401	R0000399	EQ	.000000	.000000	.000000	.000000
E	402	R0000400	EQ	.000000	.000000	.000000	.000000
E	403	R0000401	EQ	.000000	.000000	.000000	.000000
E	404	R0000402	EQ	.000000	.000000	.000000	.000000
E	405	R0000403	EQ	.000000	.000000	.000000	.000000
E	406	R0000404	EQ	.000000	.000000	.000000	.000000
E	407	R0000405	EQ	.000000	.000000	.000000	.000000
E	408	R0000406	EQ	.000000	.000000	.000000	.000000
E	409	R0000407	EQ	.000000	.000000	.000000	.000000
E	410	R0000408	EQ	.000000	.000000	.000000	.000000
E	411	R0000409	EQ	.000000	.000000	.000000	.000000
E	412	R0000410	EQ	.000000	.000000	.000000	.000000
E	413	R0000411	EQ	.000000	.000000	.000000	.000000
E	414	R0000412	EQ	.000000	.000000	.000000	.000000
E	415	R0000413	EQ	.000000	.000000	.000000	.000000
E	416	R0000414	EQ	.000000	.000000	.000000	.000000
E	417	R0000415	EQ	.000000	.000000	.000000	.000000
E	418	R0000416	EQ	.000000	.000000	.000000	.000000
E	419	R0000417	EQ	.000000	.000000	.000000	.000000
E	420	R0000418	EQ	.000000	.000000	.000000	.000000
	Number	Row	At	Value	Slack Value	Dual Value	RHS
E	421	R0000419	EQ	.000000	.000000	.000000	.000000
E	422	R0000420	EQ	.000000	.000000	.000000	.000000
E	423	R0000421	EQ	.000000	.000000	.000000	.000000
E	424	R0000422	EQ	.000000	.000000	.000000	.000000
E	425	R0000423	EQ	.000000	.000000	.000000	.000000
E	426	R0000424	EQ	.000000	.000000	.000000	.000000
E	427	R0000425	EQ	.000000	.000000	.000000	.000000
E	428	R0000426	EQ	.000000	.000000	.000000	.000000
E	429	R0000427	EQ	.000000	.000000	.000000	.000000
E	430	R0000428	EQ	.000000	.000000	.000000	.000000
E	431	R0000429	EQ	.000000	.000000	.000000	.000000
E	432	R0000430	EQ	.000000	.000000	.000000	.000000
E	433	R0000431	EQ	.000000	.000000	.000000	.000000
E	434	R0000432	EQ	.000000	.000000	.000000	.000000
E	435	R0000433	EQ	.000000	.000000	.000000	.000000
E	436	R0000434	EQ	.000000	.000000	.000000	.000000
E	437	R0000435	EQ	.000000	.000000	.000000	.000000
E	438	R0000436	EQ	.000000	.000000	.000000	.000000
E	439	R0000437	EQ	.000000	.000000	.000000	.000000
E	440	R0000438	EQ	.000000	.000000	.000000	.000000
E	441	R0000439	EQ	.000000	.000000	.000000	.000000
	Number	Row	At	Value	Slack Value	Dual Value	RHS
E	442	R0000440	EQ	.000000	.000000	.000000	.000000
E	443	R0000441	EQ	.000000	.000000	.000000	.000000
E	444	R0000442	EQ	.000000	.000000	.000000	.000000
E	445	R0000443	EQ	.000000	.000000	.000000	.000000
E	446	R0000444	EQ	.000000	.000000	.000000	.000000
E	447	R0000445	EQ	.000000	.000000	.000000	.000000
E	448	R0000446	EQ	.000000	.000000	.000000	.000000
E	449	R0000447	EQ	.000000	.000000	.000000	.000000
E	450	R0000448	EQ	.000000	.000000	.000000	.000000
E	451	R0000449	EQ	.000000	.000000	.000000	.000000
E	452	R0000450	EQ	.000000	.000000	.000000	.000000
E	453	R0000451	EQ	.000000	.000000	.000000	.000000
E	454	R0000452	EQ	.000000	.000000	.000000	.000000
E	455	R0000453	EQ	.000000	.000000	.000000	.000000
E	456	R0000454	EQ	.000000	.000000	.000000	.000000

E	457	R0000455	EQ	.000000	.000000	.000000	.000000
E	458	R0000456	EQ	.000000	.000000	.000000	.000000
E	459	R0000457	EQ	.000000	.000000	.000000	.000000
E	460	R0000458	EQ	.000000	.000000	.000000	.000000
E	461	R0000459	EQ	.000000	.000000	.000000	.000000
E	462	R0000460	EQ	.000000	.000000	.000000	.000000
	Number	Row	At	Value	Slack Value	Dual Value	RHS
E	463	R0000461	EQ	.000000	.000000	.000000	.000000
E	464	R0000462	EQ	.000000	.000000	.000000	.000000
E	465	R0000463	EQ	.000000	.000000	.000000	.000000
E	466	R0000464	EQ	.000000	.000000	.000000	.000000
E	467	R0000465	EQ	.000000	.000000	.000000	.000000
E	468	R0000466	EQ	.000000	.000000	.000000	.000000
E	469	R0000467	EQ	.000000	.000000	.000000	.000000
E	470	R0000468	EQ	.000000	.000000	.000000	.000000
E	471	R0000469	EQ	.000000	.000000	.000000	.000000
E	472	R0000470	EQ	.000000	.000000	.000000	.000000
E	473	R0000471	EQ	.000000	.000000	.000000	.000000
E	474	R0000472	EQ	.000000	.000000	.000000	.000000
E	475	R0000473	EQ	.000000	.000000	.000000	.000000
E	476	R0000474	EQ	.000000	.000000	.000000	.000000
E	477	R0000475	EQ	.000000	.000000	.000000	.000000
E	478	R0000476	EQ	.000000	.000000	.000000	.000000
E	479	R0000477	EQ	.000000	.000000	.000000	.000000
E	480	R0000478	EQ	.000000	.000000	.000000	.000000
E	481	R0000479	EQ	.000000	.000000	.000000	.000000
E	482	R0000480	EQ	.000000	.000000	.000000	.000000
E	483	R0000481	EQ	.000000	.000000	.000000	.000000
	Number	Row	At	Value	Slack Value	Dual Value	RHS
E	484	R0000482	EQ	.000000	.000000	.000000	.000000
E	485	R0000483	EQ	.000000	.000000	.000000	.000000
E	486	R0000484	EQ	.000000	.000000	.000000	.000000
E	487	R0000485	EQ	.000000	.000000	.000000	.000000
E	488	R0000486	EQ	.000000	.000000	.000000	.000000
E	489	R0000487	EQ	.000000	.000000	.000000	.000000
E	490	R0000488	EQ	.000000	.000000	.000000	.000000
E	491	R0000489	EQ	.000000	.000000	.000000	.000000
E	492	R0000490	EQ	.000000	.000000	.000000	.000000
E	493	R0000491	EQ	.000000	.000000	.000000	.000000
E	494	R0000492	EQ	.000000	.000000	.000000	.000000
E	495	R0000493	EQ	.000000	.000000	.000000	.000000
E	496	R0000494	EQ	.000000	.000000	.000000	.000000
E	497	R0000495	EQ	.000000	.000000	.000000	.000000
E	498	R0000496	EQ	.000000	.000000	.000000	.000000
E	499	R0000497	EQ	.000000	.000000	.000000	.000000
E	500	R0000498	EQ	.000000	.000000	.000000	.000000
E	501	R0000499	EQ	.000000	.000000	.000000	.000000
E	502	R0000500	EQ	.000000	.000000	.000000	.000000
E	503	R0000501	EQ	.000000	.000000	.000000	.000000
E	504	R0000502	EQ	.000000	.000000	.000000	.000000
	Number	Row	At	Value	Slack Value	Dual Value	RHS
E	505	R0000503	EQ	.000000	.000000	.000000	.000000
E	506	R0000504	EQ	.000000	.000000	.000000	.000000
E	507	R0000505	EQ	.000000	.000000	.000000	.000000
E	508	R0000506	EQ	.000000	.000000	.000000	.000000
E	509	R0000507	EQ	.000000	.000000	.000000	.000000
E	510	R0000508	EQ	.000000	.000000	.000000	.000000
E	511	R0000509	EQ	.000000	.000000	.000000	.000000
E	512	R0000510	EQ	.000000	.000000	.000000	.000000
E	513	R0000511	EQ	.000000	.000000	.000000	.000000
E	514	R0000512	EQ	.000000	.000000	.000000	.000000
E	515	R0000513	EQ	.000000	.000000	.000000	.000000
E	516	R0000514	EQ	.000000	.000000	.000000	.000000
E	517	R0000515	EQ	.000000	.000000	.000000	.000000
E	518	R0000516	EQ	.000000	.000000	.000000	.000000
E	519	R0000517	EQ	.000000	.000000	.000000	.000000
E	520	R0000518	EQ	.000000	.000000	.000000	.000000
E	521	R0000519	EQ	.000000	.000000	.000000	.000000
E	522	R0000520	EQ	.000000	.000000	.000000	.000000
E	523	R0000521	EQ	.000000	.000000	.000000	.000000
E	524	R0000522	EQ	.000000	.000000	.000000	.000000

	Number	Row	At	Value	Slack Value	Dual Value	RHS
E	525	R0000523	EQ	.000000	.000000	.000000	.000000
	Number	Row	At	Value	Slack Value	Dual Value	RHS
E	526	R0000524	EQ	.000000	.000000	.000000	.000000
E	527	R0000525	EQ	.000000	.000000	.000000	.000000
E	528	R0000526	EQ	.000000	.000000	.000000	.000000
E	529	R0000527	EQ	.000000	.000000	.000000	.000000
E	530	R0000528	EQ	.000000	.000000	.000000	.000000
E	531	R0000529	EQ	.000000	.000000	.000000	.000000
E	532	R0000530	EQ	.000000	.000000	.000000	.000000
E	533	R0000531	EQ	.000000	.000000	.000000	.000000
E	534	R0000532	EQ	.000000	.000000	.000000	.000000
E	535	R0000533	EQ	.000000	.000000	.000000	.000000
E	536	R0000534	EQ	.000000	.000000	.000000	.000000
E	537	R0000535	EQ	.000000	.000000	.000000	.000000
E	538	R0000536	EQ	.000000	.000000	.000000	.000000
E	539	R0000537	EQ	.000000	.000000	.000000	.000000
E	540	R0000538	EQ	.000000	.000000	.000000	.000000
E	541	R0000539	EQ	.000000	.000000	.000000	.000000
E	542	R0000540	EQ	.000000	.000000	.000000	.000000
E	543	R0000541	EQ	.000000	.000000	.000000	.000000
E	544	R0000542	EQ	.000000	.000000	.000000	.000000
E	545	R0000543	EQ	.000000	.000000	.000000	.000000
E	546	R0000544	EQ	.000000	.000000	.000000	.000000
	Number	Row	At	Value	Slack Value	Dual Value	RHS
E	547	R0000545	EQ	.000000	.000000	.000000	.000000
E	548	R0000546	EQ	.000000	.000000	.000000	.000000
E	549	R0000547	EQ	.000000	.000000	.000000	.000000
E	550	R0000548	EQ	.000000	.000000	.000000	.000000
E	551	R0000549	EQ	.000000	.000000	.000000	.000000
E	552	R0000550	EQ	.000000	.000000	.000000	.000000
E	553	R0000551	EQ	.000000	.000000	.000000	.000000
E	554	R0000552	EQ	.000000	.000000	.000000	.000000
E	555	R0000553	EQ	.000000	.000000	.000000	.000000
E	556	R0000554	EQ	.000000	.000000	.000000	.000000
E	557	R0000555	EQ	.000000	.000000	.000000	.000000
E	558	R0000556	EQ	.000000	.000000	.000000	.000000
E	559	R0000557	EQ	.000000	.000000	.000000	.000000
E	560	R0000558	EQ	.000000	.000000	.000000	.000000
E	561	R0000559	EQ	.000000	.000000	.000000	.000000
E	562	R0000560	EQ	.000000	.000000	.000000	.000000
E	563	R0000561	EQ	.000000	.000000	.000000	.000000
E	564	R0000562	EQ	.000000	.000000	.000000	.000000
E	565	R0000563	EQ	.000000	.000000	.000000	.000000
E	566	R0000564	EQ	.000000	.000000	.000000	.000000
E	567	R0000565	EQ	.000000	.000000	.000000	.000000
	Number	Row	At	Value	Slack Value	Dual Value	RHS
E	568	R0000566	EQ	.000000	.000000	.000000	.000000
E	569	R0000567	EQ	.000000	.000000	.000000	.000000
E	570	R0000568	EQ	.000000	.000000	.000000	.000000
E	571	R0000569	EQ	.000000	.000000	.000000	.000000
E	572	R0000570	EQ	.000000	.000000	.000000	.000000
E	573	R0000571	EQ	.000000	.000000	.000000	.000000
E	574	R0000572	EQ	.000000	.000000	.000000	.000000
E	575	R0000573	EQ	.000000	.000000	.000000	.000000
E	576	R0000574	EQ	.000000	.000000	.000000	.000000
E	577	R0000575	EQ	.000000	.000000	.000000	.000000
E	578	R0000576	EQ	.000000	.000000	.000000	.000000
E	579	R0000577	EQ	.000000	.000000	.000000	.000000
E	580	R0000578	EQ	.000000	.000000	.000000	.000000
E	581	R0000579	EQ	.000000	.000000	.000000	.000000
E	582	R0000580	EQ	.000000	.000000	.000000	.000000
E	583	R0000581	EQ	.000000	.000000	.000000	.000000
E	584	R0000582	EQ	.000000	.000000	.000000	.000000
E	585	R0000583	EQ	.000000	.000000	.000000	.000000
E	586	R0000584	EQ	.000000	.000000	.000000	.000000
E	587	R0000585	EQ	.000000	.000000	.000000	.000000
E	588	R0000586	EQ	.000000	.000000	.000000	.000000
	Number	Row	At	Value	Slack Value	Dual Value	RHS
E	589	R0000587	EQ	.000000	.000000	.000000	.000000
E	590	R0000588	EQ	.000000	.000000	.000000	.000000
E	591	R0000589	EQ	.000000	.000000	.000000	.000000

E	592	R0000590	EQ	.000000	.000000	.000000	.000000
E	593	R0000591	EQ	.000000	.000000	.000000	.000000
E	594	R0000592	EQ	.000000	.000000	.000000	.000000
E	595	R0000593	EQ	.000000	.000000	.000000	.000000
E	596	R0000594	EQ	.000000	.000000	.000000	.000000
E	597	R0000595	EQ	.000000	.000000	.000000	.000000
E	598	R0000596	EQ	.000000	.000000	.000000	.000000
E	599	R0000597	EQ	.000000	.000000	.000000	.000000
E	600	R0000598	EQ	.000000	.000000	.000000	.000000
E	601	R0000599	EQ	.000000	.000000	.000000	.000000
E	602	R0000600	EQ	.000000	.000000	.000000	.000000
E	603	R0000601	EQ	.000000	.000000	.000000	.000000
E	604	R0000602	EQ	.000000	.000000	.000000	.000000
E	605	R0000603	EQ	.000000	.000000	.000000	.000000
E	606	R0000604	EQ	.000000	.000000	.000000	.000000
E	607	R0000605	EQ	.000000	.000000	.000000	.000000
E	608	R0000606	EQ	.000000	.000000	.000000	.000000
E	609	R0000607	EQ	.000000	.000000	.000000	.000000
	Number	Row	At	Value	Slack Value	Dual Value	RHS
E	610	R0000608	EQ	.000000	.000000	.000000	.000000
E	611	R0000609	EQ	.000000	.000000	.000000	.000000
E	612	R0000610	EQ	.000000	.000000	.000000	.000000
E	613	R0000611	EQ	.000000	.000000	.000000	.000000
E	614	R0000612	EQ	.000000	.000000	.000000	.000000
E	615	R0000613	EQ	.000000	.000000	.000000	.000000
E	616	R0000614	EQ	.000000	.000000	.000000	.000000
E	617	R0000615	EQ	.000000	.000000	.000000	.000000
E	618	R0000616	EQ	.000000	.000000	.000000	.000000
E	619	R0000617	EQ	.000000	.000000	.000000	.000000
E	620	R0000618	EQ	.000000	.000000	.000000	.000000
E	621	R0000619	EQ	.000000	.000000	.000000	.000000
E	622	R0000620	EQ	.000000	.000000	.000000	.000000
E	623	R0000621	EQ	.000000	.000000	.000000	.000000
E	624	R0000622	EQ	.000000	.000000	.000000	.000000
E	625	R0000623	EQ	.000000	.000000	.000000	.000000
E	626	R0000624	EQ	.000000	.000000	.000000	.000000
E	627	R0000625	EQ	.000000	.000000	.000000	.000000
E	628	R0000626	EQ	.000000	.000000	.000000	.000000
E	629	R0000627	EQ	.000000	.000000	.000000	.000000
E	630	R0000628	EQ	.000000	.000000	.000000	.000000
	Number	Row	At	Value	Slack Value	Dual Value	RHS
E	631	R0000629	EQ	.000000	.000000	.000000	.000000
E	632	R0000630	EQ	.000000	.000000	.000000	.000000
E	633	R0000631	EQ	.000000	.000000	.000000	.000000
E	634	R0000632	EQ	.000000	.000000	.000000	.000000
E	635	R0000633	EQ	.000000	.000000	.000000	.000000
E	636	R0000634	EQ	.000000	.000000	.000000	.000000
E	637	R0000635	EQ	.000000	.000000	.000000	.000000
E	638	R0000636	EQ	.000000	.000000	.000000	.000000
E	639	R0000637	EQ	.000000	.000000	.000000	.000000
E	640	R0000638	EQ	.000000	.000000	.000000	.000000
E	641	R0000639	EQ	.000000	.000000	.000000	.000000
E	642	R0000640	EQ	.000000	.000000	.000000	.000000
E	643	R0000641	EQ	.000000	.000000	.000000	.000000
E	644	R0000642	EQ	.000000	.000000	.000000	.000000
E	645	R0000643	EQ	.000000	.000000	.000000	.000000
E	646	R0000644	EQ	.000000	.000000	.000000	.000000
E	647	R0000645	EQ	.000000	.000000	.000000	.000000
E	648	R0000646	EQ	.000000	.000000	.000000	.000000
E	649	R0000647	EQ	.000000	.000000	.000000	.000000
E	650	R0000648	EQ	.000000	.000000	.000000	.000000
E	651	R0000649	EQ	.000000	.000000	.000000	.000000
	Number	Row	At	Value	Slack Value	Dual Value	RHS
E	652	R0000650	EQ	.000000	.000000	.000000	.000000
E	653	R0000651	EQ	.000000	.000000	.000000	.000000
E	654	R0000652	EQ	.000000	.000000	.000000	.000000
E	655	R0000653	EQ	.000000	.000000	.000000	.000000
E	656	R0000654	EQ	.000000	.000000	.000000	.000000
E	657	R0000655	EQ	.000000	.000000	.000000	.000000
E	658	R0000656	EQ	.000000	.000000	.000000	.000000
E	659	R0000657	EQ	.000000	.000000	.000000	.000000

	Number	Row	At	Value	Slack Value	Dual Value	RHS
E	660	R0000658	EQ	.000000	.000000	.000000	.000000
E	661	R0000659	EQ	.000000	.000000	.000000	.000000
E	662	R0000660	EQ	.000000	.000000	.000000	.000000
E	663	R0000661	EQ	.000000	.000000	.000000	.000000
E	664	R0000662	EQ	.000000	.000000	.000000	.000000
E	665	R0000663	EQ	.000000	.000000	.000000	.000000
E	666	R0000664	EQ	.000000	.000000	.000000	.000000
E	667	R0000665	EQ	.000000	.000000	.000000	.000000
E	668	R0000666	EQ	.000000	.000000	.000000	.000000
E	669	R0000667	EQ	.000000	.000000	.000000	.000000
E	670	R0000668	EQ	.000000	.000000	.000000	.000000
E	671	R0000669	EQ	.000000	.000000	.000000	.000000
E	672	R0000670	EQ	.000000	.000000	.000000	.000000
	Number	Row	At	Value	Slack Value	Dual Value	RHS
E	673	R0000671	EQ	.000000	.000000	.000000	.000000
E	674	R0000672	EQ	.000000	.000000	.000000	.000000
E	675	R0000673	EQ	.000000	.000000	.000000	.000000
E	676	R0000674	EQ	.000000	.000000	.000000	.000000
E	677	R0000675	EQ	.000000	.000000	.000000	.000000
E	678	R0000676	EQ	.000000	.000000	.000000	.000000
E	679	R0000677	EQ	.000000	.000000	.000000	.000000
E	680	R0000678	EQ	.000000	.000000	.000000	.000000
E	681	R0000679	EQ	.000000	.000000	.000000	.000000
E	682	R0000680	EQ	.000000	.000000	.000000	.000000
E	683	R0000681	EQ	.000000	.000000	.000000	.000000
E	684	R0000682	EQ	.000000	.000000	.000000	.000000
E	685	R0000683	EQ	.000000	.000000	.000000	.000000
E	686	R0000684	EQ	.000000	.000000	.000000	.000000
E	687	R0000685	EQ	.000000	.000000	.000000	.000000
E	688	R0000686	EQ	.000000	.000000	.000000	.000000
E	689	R0000687	EQ	.000000	.000000	.000000	.000000
E	690	R0000688	EQ	.000000	.000000	.000000	.000000
E	691	R0000689	EQ	.000000	.000000	.000000	.000000
E	692	R0000690	EQ	.000000	.000000	.000000	.000000
E	693	R0000691	EQ	.000000	.000000	.000000	.000000
	Number	Row	At	Value	Slack Value	Dual Value	RHS
E	694	R0000692	EQ	.000000	.000000	.000000	.000000
E	695	R0000693	EQ	.000000	.000000	.000000	.000000
E	696	R0000694	EQ	.000000	.000000	.000000	.000000
E	697	R0000695	EQ	.000000	.000000	.000000	.000000
E	698	R0000696	EQ	.000000	.000000	.000000	.000000
E	699	R0000697	EQ	.000000	.000000	.000000	.000000
E	700	R0000698	EQ	.000000	.000000	.000000	.000000
E	701	R0000699	EQ	.000000	.000000	.000000	.000000
E	702	R0000700	EQ	.000000	.000000	.000000	.000000
E	703	R0000701	EQ	.000000	.000000	.000000	.000000
E	704	R0000702	EQ	.000000	.000000	.000000	.000000
E	705	R0000703	EQ	.000000	.000000	.000000	.000000
E	706	R0000704	EQ	.000000	.000000	.000000	.000000
E	707	R0000705	EQ	.000000	.000000	.000000	.000000
E	708	R0000706	EQ	.000000	.000000	.000000	.000000
E	709	R0000707	EQ	.000000	.000000	.000000	.000000
E	710	R0000708	EQ	.000000	.000000	.000000	.000000
E	711	R0000709	EQ	.000000	.000000	.000000	.000000
E	712	R0000710	EQ	.000000	.000000	.000000	.000000
E	713	R0000711	EQ	.000000	.000000	.000000	.000000
E	714	R0000712	EQ	.000000	.000000	.000000	.000000
	Number	Row	At	Value	Slack Value	Dual Value	RHS
E	715	R0000713	EQ	.000000	.000000	.000000	.000000
E	716	R0000714	EQ	.000000	.000000	.000000	.000000
E	717	R0000715	EQ	.000000	.000000	.000000	.000000
E	718	R0000716	EQ	.000000	.000000	.000000	.000000
E	719	R0000717	EQ	.000000	.000000	.000000	.000000
E	720	R0000718	EQ	.000000	.000000	.000000	.000000
E	721	R0000719	EQ	.000000	.000000	.000000	.000000
E	722	R0000720	EQ	.000000	.000000	.000000	.000000
E	723	R0000721	EQ	.000000	.000000	.000000	.000000
E	724	R0000722	EQ	.000000	.000000	.000000	.000000
E	725	R0000723	EQ	.000000	.000000	.000000	.000000
E	726	R0000724	EQ	.000000	.000000	.000000	.000000
E	727	R0000725	EQ	.000000	.000000	.000000	.000000

E	728	R0000726	EQ	.000000	.000000	.000000	.000000
E	729	R0000727	EQ	.000000	.000000	.000000	.000000
E	730	R0000728	EQ	.000000	.000000	.000000	.000000
E	731	R0000729	EQ	.000000	.000000	.000000	.000000
E	732	R0000730	EQ	.000000	.000000	.000000	.000000
E	733	R0000731	EQ	.000000	.000000	.000000	.000000
E	734	R0000732	EQ	.000000	.000000	.000000	.000000
E	735	R0000733	EQ	.000000	.000000	.000000	.000000
	Number	Row	At	Value	Slack Value	Dual Value	RHS
E	736	R0000734	EQ	.000000	.000000	.000000	.000000
E	737	R0000735	EQ	.000000	.000000	.000000	.000000
E	738	R0000736	EQ	.000000	.000000	.000000	.000000
E	739	R0000737	EQ	.000000	.000000	.000000	.000000
E	740	R0000738	EQ	.000000	.000000	.000000	.000000
E	741	R0000739	EQ	.000000	.000000	.000000	.000000
E	742	R0000740	EQ	.000000	.000000	.000000	.000000
E	743	R0000741	EQ	.000000	.000000	.000000	.000000
E	744	R0000742	EQ	.000000	.000000	.000000	.000000
E	745	R0000743	EQ	.000000	.000000	.000000	.000000
E	746	R0000744	EQ	.000000	.000000	.000000	.000000
E	747	R0000745	EQ	.000000	.000000	.000000	.000000
E	748	R0000746	EQ	.000000	.000000	.000000	.000000
E	749	R0000747	EQ	.000000	.000000	.000000	.000000
E	750	R0000748	EQ	.000000	.000000	.000000	.000000
E	751	R0000749	EQ	.000000	.000000	.000000	.000000
E	752	R0000750	EQ	.000000	.000000	.000000	.000000
E	753	R0000751	EQ	.000000	.000000	.000000	.000000
E	754	R0000752	EQ	.000000	.000000	.000000	.000000
E	755	R0000753	EQ	.000000	.000000	.000000	.000000
E	756	R0000754	EQ	.000000	.000000	.000000	.000000
	Number	Row	At	Value	Slack Value	Dual Value	RHS
E	757	R0000755	EQ	.000000	.000000	.000000	.000000
E	758	R0000756	EQ	.000000	.000000	.000000	.000000
E	759	R0000757	EQ	.000000	.000000	.000000	.000000
E	760	R0000758	EQ	.000000	.000000	.000000	.000000
E	761	R0000759	EQ	.000000	.000000	.000000	.000000
E	762	R0000760	EQ	.000000	.000000	.000000	.000000
E	763	R0000761	EQ	.000000	.000000	.000000	.000000
E	764	R0000762	EQ	.000000	.000000	.000000	.000000
E	765	R0000763	EQ	.000000	.000000	.000000	.000000
E	766	R0000764	EQ	.000000	.000000	.000000	.000000
E	767	R0000765	EQ	.000000	.000000	.000000	.000000
E	768	R0000766	EQ	.000000	.000000	.000000	.000000
E	769	R0000767	EQ	.000000	.000000	.000000	.000000
E	770	R0000768	EQ	.000000	.000000	.000000	.000000
E	771	R0000769	EQ	.000000	.000000	.000000	.000000
E	772	R0000770	EQ	.000000	.000000	.000000	.000000
E	773	R0000771	EQ	.000000	.000000	.000000	.000000
E	774	R0000772	EQ	.000000	.000000	.000000	.000000
E	775	R0000773	EQ	.000000	.000000	.000000	.000000
E	776	R0000774	EQ	.000000	.000000	.000000	.000000
E	777	R0000775	EQ	.000000	.000000	.000000	.000000
	Number	Row	At	Value	Slack Value	Dual Value	RHS
E	778	R0000776	EQ	.000000	.000000	.000000	.000000
E	779	R0000777	EQ	.000000	.000000	.000000	.000000
E	780	R0000778	EQ	.000000	.000000	.000000	.000000
E	781	R0000779	EQ	.000000	.000000	.000000	.000000
E	782	R0000780	EQ	.000000	.000000	.000000	.000000
E	783	R0000781	EQ	.000000	.000000	.000000	.000000
E	784	R0000782	EQ	.000000	.000000	.000000	.000000
E	785	R0000783	EQ	.000000	.000000	.000000	.000000
E	786	R0000784	EQ	.000000	.000000	.000000	.000000
E	787	R0000785	EQ	.000000	.000000	.000000	.000000
E	788	R0000786	EQ	.000000	.000000	.000000	.000000
E	789	R0000787	EQ	.000000	.000000	.000000	.000000
E	790	R0000788	EQ	.000000	.000000	.000000	.000000
E	791	R0000789	EQ	.000000	.000000	.000000	.000000
E	792	R0000790	EQ	.000000	.000000	.000000	.000000
E	793	R0000791	EQ	.000000	.000000	.000000	.000000
E	794	R0000792	EQ	.000000	.000000	.000000	.000000
E	795	R0000793	EQ	.000000	.000000	.000000	.000000

E	796	R0000794	EQ	.000000	.000000	.000000	.000000
E	797	R0000795	EQ	.000000	.000000	.000000	.000000
E	798	R0000796	EQ	.000000	.000000	.000000	.000000
	Number	Row	At	Value	Slack Value	Dual Value	RHS
E	799	R0000797	EQ	.000000	.000000	.000000	.000000
E	800	R0000798	EQ	.000000	.000000	.000000	.000000
E	801	R0000799	EQ	.000000	.000000	.000000	.000000
E	802	R0000800	EQ	.000000	.000000	.000000	.000000
E	803	R0000801	EQ	.000000	.000000	.000000	.000000
E	804	R0000802	EQ	.000000	.000000	.000000	.000000
E	805	R0000803	EQ	.000000	.000000	.000000	.000000
E	806	R0000804	EQ	.000000	.000000	.000000	.000000
E	807	R0000805	EQ	.000000	.000000	.000000	.000000
E	808	R0000806	EQ	.000000	.000000	.000000	.000000
E	809	R0000807	EQ	.000000	.000000	.000000	.000000
E	810	R0000808	EQ	.000000	.000000	.000000	.000000
E	811	R0000809	EQ	.000000	.000000	.000000	.000000
E	812	R0000810	EQ	.000000	.000000	.000000	.000000
E	813	R0000811	EQ	.000000	.000000	.000000	.000000
E	814	R0000812	EQ	.000000	.000000	.000000	.000000
E	815	R0000813	EQ	.000000	.000000	.000000	.000000
E	816	R0000814	EQ	.000000	.000000	.000000	.000000
E	817	R0000815	EQ	.000000	.000000	.000000	.000000
E	818	R0000816	EQ	.000000	.000000	.000000	.000000
E	819	R0000817	EQ	.000000	.000000	.000000	.000000
	Number	Row	At	Value	Slack Value	Dual Value	RHS
E	820	R0000818	EQ	.000000	.000000	.000000	.000000
E	821	R0000819	EQ	.000000	.000000	.000000	.000000
E	822	R0000820	EQ	.000000	.000000	.000000	.000000
E	823	R0000821	EQ	.000000	.000000	.000000	.000000
E	824	R0000822	EQ	.000000	.000000	.000000	.000000
E	825	R0000823	EQ	.000000	.000000	.000000	.000000
E	826	R0000824	EQ	.000000	.000000	.000000	.000000
E	827	R0000825	EQ	.000000	.000000	.000000	.000000
E	828	R0000826	EQ	.000000	.000000	.000000	.000000
E	829	R0000827	EQ	.000000	.000000	.000000	.000000
E	830	R0000828	EQ	.000000	.000000	.000000	.000000
E	831	R0000829	EQ	.000000	.000000	.000000	.000000
E	832	R0000830	EQ	.000000	.000000	.000000	.000000
E	833	R0000831	EQ	.000000	.000000	.000000	.000000
E	834	R0000832	EQ	.000000	.000000	.000000	.000000
E	835	R0000833	EQ	.000000	.000000	.000000	.000000
E	836	R0000834	EQ	.000000	.000000	.000000	.000000
E	837	R0000835	EQ	.000000	.000000	.000000	.000000
E	838	R0000836	EQ	.000000	.000000	.000000	.000000
E	839	R0000837	EQ	.000000	.000000	.000000	.000000
E	840	R0000838	EQ	.000000	.000000	.000000	.000000
	Number	Row	At	Value	Slack Value	Dual Value	RHS
E	841	R0000839	EQ	.000000	.000000	.000000	.000000
E	842	R0000840	EQ	.000000	.000000	.000000	.000000
E	843	R0000841	EQ	.000000	.000000	.000000	.000000
E	844	R0000842	EQ	.000000	.000000	.000000	.000000
E	845	R0000843	EQ	.000000	.000000	.000000	.000000
E	846	R0000844	EQ	.000000	.000000	.000000	.000000
E	847	R0000845	EQ	.000000	.000000	.000000	.000000
E	848	R0000846	EQ	.000000	.000000	.000000	.000000
E	849	R0000847	EQ	.000000	.000000	.000000	.000000
E	850	R0000848	EQ	.000000	.000000	.000000	.000000
E	851	R0000849	EQ	.000000	.000000	.000000	.000000
E	852	R0000850	EQ	.000000	.000000	.000000	.000000
E	853	R0000851	EQ	.000000	.000000	.000000	.000000
E	854	R0000852	EQ	.000000	.000000	.000000	.000000
E	855	R0000853	EQ	.000000	.000000	.000000	.000000
E	856	R0000854	EQ	.000000	.000000	.000000	.000000
E	857	R0000855	EQ	.000000	.000000	.000000	.000000
E	858	R0000856	EQ	.000000	.000000	.000000	.000000
E	859	R0000857	EQ	.000000	.000000	.000000	.000000
E	860	R0000858	EQ	.000000	.000000	.000000	.000000
E	861	R0000859	EQ	.000000	.000000	.000000	.000000
	Number	Row	At	Value	Slack Value	Dual Value	RHS
E	862	R0000860	EQ	.000000	.000000	.000000	.000000

	Number	Row	At	Value	Slack Value	Dual Value	RHS
E	863	R0000861	EQ	.000000	.000000	.000000	.000000
E	864	R0000862	EQ	.000000	.000000	.000000	.000000
E	865	R0000863	EQ	.000000	.000000	.000000	.000000
E	866	R0000864	EQ	.000000	.000000	.000000	.000000
E	867	R0000865	EQ	.000000	.000000	.000000	.000000
E	868	R0000866	EQ	.000000	.000000	.000000	.000000
E	869	R0000867	EQ	.000000	.000000	.000000	.000000
E	870	R0000868	EQ	.000000	.000000	.000000	.000000
E	871	R0000869	EQ	.000000	.000000	.000000	.000000
E	872	R0000870	EQ	.000000	.000000	.000000	.000000
E	873	R0000871	EQ	.000000	.000000	.000000	.000000
E	874	R0000872	EQ	.000000	.000000	.000000	.000000
E	875	R0000873	EQ	.000000	.000000	.000000	.000000
E	876	R0000874	EQ	.000000	.000000	.000000	.000000
E	877	R0000875	EQ	.000000	.000000	.000000	.000000
E	878	R0000876	EQ	.000000	.000000	.000000	.000000
E	879	R0000877	EQ	.000000	.000000	.000000	.000000
E	880	R0000878	EQ	.000000	.000000	.000000	.000000
E	881	R0000879	EQ	.000000	.000000	.000000	.000000
E	882	R0000880	EQ	.000000	.000000	.000000	.000000
	Number	Row	At	Value	Slack Value	Dual Value	RHS
E	883	R0000881	EQ	.000000	.000000	.000000	.000000
E	884	R0000882	EQ	.000000	.000000	.000000	.000000
E	885	R0000883	EQ	.000000	.000000	.000000	.000000
E	886	R0000884	EQ	.000000	.000000	.000000	.000000
E	887	R0000885	EQ	.000000	.000000	.000000	.000000
E	888	R0000886	EQ	.000000	.000000	.000000	.000000
E	889	R0000887	EQ	.000000	.000000	.000000	.000000
E	890	R0000888	EQ	.000000	.000000	.000000	.000000
E	891	R0000889	EQ	.000000	.000000	.000000	.000000
E	892	R0000890	EQ	.000000	.000000	.000000	.000000
E	893	R0000891	EQ	.000000	.000000	.000000	.000000
E	894	R0000892	EQ	.000000	.000000	.000000	.000000
E	895	R0000893	EQ	.000000	.000000	.000000	.000000
E	896	R0000894	EQ	.000000	.000000	.000000	.000000
E	897	R0000895	EQ	.000000	.000000	.000000	.000000
E	898	R0000896	EQ	.000000	.000000	.000000	.000000
E	899	R0000897	EQ	.000000	.000000	.000000	.000000
E	900	R0000898	EQ	.000000	.000000	.000000	.000000
E	901	R0000899	EQ	.000000	.000000	.000000	.000000
E	902	R0000900	EQ	.000000	.000000	.000000	.000000
E	903	R0000901	EQ	.000000	.000000	.000000	.000000
	Number	Row	At	Value	Slack Value	Dual Value	RHS
E	904	R0000902	EQ	.000000	.000000	.000000	.000000
E	905	R0000903	EQ	.000000	.000000	.000000	.000000
E	906	R0000904	EQ	.000000	.000000	.000000	.000000
E	907	R0000905	EQ	.000000	.000000	.000000	.000000
E	908	R0000906	EQ	.000000	.000000	.000000	.000000
E	909	R0000907	EQ	.000000	.000000	.000000	.000000
E	910	R0000908	EQ	.000000	.000000	.000000	.000000
E	911	R0000909	EQ	.000000	.000000	.000000	.000000
E	912	R0000910	EQ	.000000	.000000	.000000	.000000
E	913	R0000911	EQ	.000000	.000000	.000000	.000000
E	914	R0000912	EQ	.000000	.000000	.000000	.000000
E	915	R0000913	EQ	.000000	.000000	.000000	.000000
E	916	R0000914	EQ	.000000	.000000	.000000	.000000
E	917	R0000915	EQ	.000000	.000000	.000000	.000000
E	918	R0000916	EQ	.000000	.000000	.000000	.000000
E	919	R0000917	EQ	.000000	.000000	.000000	.000000
E	920	R0000918	EQ	.000000	.000000	.000000	.000000
E	921	R0000919	EQ	.000000	.000000	.000000	.000000
E	922	R0000920	EQ	.000000	.000000	.000000	.000000
E	923	R0000921	EQ	.000000	.000000	.000000	.000000
E	924	R0000922	EQ	.000000	.000000	.000000	.000000
	Number	Row	At	Value	Slack Value	Dual Value	RHS
E	925	R0000923	EQ	.000000	.000000	.000000	.000000
E	926	R0000924	EQ	.000000	.000000	.000000	.000000
E	927	R0000925	EQ	.000000	.000000	.000000	.000000
E	928	R0000926	EQ	.000000	.000000	.000000	.000000
E	929	R0000927	EQ	.000000	.000000	.000000	.000000
E	930	R0000928	EQ	.000000	.000000	.000000	.000000

	Number	Row	At	Value	Slack Value	Dual Value	RHS
E	931	R0000929	EQ	.000000	.000000	.000000	.000000
E	932	R0000930	EQ	.000000	.000000	.000000	.000000
E	933	R0000931	EQ	.000000	.000000	.000000	.000000
E	934	R0000932	EQ	.000000	.000000	.000000	.000000
E	935	R0000933	EQ	.000000	.000000	.000000	.000000
E	936	R0000934	EQ	.000000	.000000	.000000	.000000
E	937	R0000935	EQ	.000000	.000000	.000000	.000000
E	938	R0000936	EQ	.000000	.000000	.000000	.000000
E	939	R0000937	EQ	.000000	.000000	.000000	.000000
E	940	R0000938	EQ	.000000	.000000	.000000	.000000
E	941	R0000939	EQ	.000000	.000000	.000000	.000000
E	942	R0000940	EQ	.000000	.000000	.000000	.000000
E	943	R0000941	EQ	.000000	.000000	.000000	.000000
E	944	R0000942	EQ	.000000	.000000	.000000	.000000
E	945	R0000943	EQ	.000000	.000000	.000000	.000000
	Number	Row	At	Value	Slack Value	Dual Value	RHS
E	946	R0000944	EQ	.000000	.000000	.000000	.000000
E	947	R0000945	EQ	.000000	.000000	.000000	.000000
E	948	R0000946	EQ	.000000	.000000	.000000	.000000
E	949	R0000947	EQ	.000000	.000000	.000000	.000000
E	950	R0000948	EQ	.000000	.000000	.000000	.000000
E	951	R0000949	EQ	.000000	.000000	.000000	.000000
E	952	R0000950	EQ	.000000	.000000	.000000	.000000
E	953	R0000951	EQ	.000000	.000000	.000000	.000000
E	954	R0000952	EQ	.000000	.000000	.000000	.000000
E	955	R0000953	EQ	.000000	.000000	.000000	.000000
E	956	R0000954	EQ	.000000	.000000	.000000	.000000
E	957	R0000955	EQ	.000000	.000000	.000000	.000000
E	958	R0000956	EQ	.000000	.000000	.000000	.000000
E	959	R0000957	EQ	.000000	.000000	.000000	.000000
E	960	R0000958	EQ	.000000	.000000	.000000	.000000
E	961	R0000959	EQ	.000000	.000000	.000000	.000000
E	962	R0000960	EQ	.000000	.000000	.000000	.000000
E	963	R0000961	EQ	.000000	.000000	.000000	.000000
E	964	R0000962	EQ	.000000	.000000	.000000	.000000
E	965	R0000963	EQ	.000000	.000000	.000000	.000000
E	966	R0000964	EQ	.000000	.000000	.000000	.000000
	Number	Row	At	Value	Slack Value	Dual Value	RHS
E	967	R0000965	EQ	.000000	.000000	.000000	.000000
E	968	R0000966	EQ	.000000	.000000	.000000	.000000
E	969	R0000967	EQ	.000000	.000000	.000000	.000000
E	970	R0000968	EQ	.000000	.000000	.000000	.000000
E	971	R0000969	EQ	.000000	.000000	.000000	.000000
E	972	R0000970	EQ	.000000	.000000	.000000	.000000
E	973	R0000971	EQ	.000000	.000000	.000000	.000000
E	974	R0000972	EQ	.000000	.000000	.000000	.000000
E	975	R0000973	EQ	.000000	.000000	.000000	.000000
E	976	R0000974	EQ	.000000	.000000	.000000	.000000
E	977	R0000975	EQ	.000000	.000000	.000000	.000000
E	978	R0000976	EQ	.000000	.000000	.000000	.000000
E	979	R0000977	EQ	.000000	.000000	.000000	.000000
E	980	R0000978	EQ	.000000	.000000	.000000	.000000
E	981	R0000979	EQ	.000000	.000000	.000000	.000000
E	982	R0000980	EQ	.000000	.000000	.000000	.000000
E	983	R0000981	EQ	.000000	.000000	.000000	.000000
E	984	R0000982	EQ	.000000	.000000	.000000	.000000
E	985	R0000983	EQ	.000000	.000000	.000000	.000000
E	986	R0000984	EQ	.000000	.000000	.000000	.000000
E	987	R0000985	EQ	.000000	.000000	.000000	.000000
	Number	Row	At	Value	Slack Value	Dual Value	RHS
E	988	R0000986	EQ	.000000	.000000	.000000	.000000
E	989	R0000987	EQ	.000000	.000000	.000000	.000000
E	990	R0000988	EQ	.000000	.000000	.000000	.000000
E	991	R0000989	EQ	.000000	.000000	.000000	.000000
E	992	R0000990	EQ	.000000	.000000	.000000	.000000
E	993	R0000991	EQ	.000000	.000000	.000000	.000000
E	994	R0000992	EQ	.000000	.000000	.000000	.000000
E	995	R0000993	EQ	.000000	.000000	.000000	.000000
E	996	R0000994	EQ	.000000	.000000	.000000	.000000
E	997	R0000995	EQ	.000000	.000000	.000000	.000000
E	998	R0000996	EQ	.000000	.000000	.000000	.000000

E	999	R0000997	EQ	.000000	.000000	.000000	.000000
E	1000	R0000998	EQ	.000000	.000000	.000000	.000000
E	1001	R0000999	EQ	.000000	.000000	.000000	.000000
E	1002	R0001000	EQ	.000000	.000000	.000000	.000000
E	1003	R0001001	EQ	.000000	.000000	.000000	.000000
E	1004	R0001002	EQ	.000000	.000000	.000000	.000000
E	1005	R0001003	EQ	.000000	.000000	.000000	.000000
E	1006	R0001004	EQ	.000000	.000000	.000000	.000000
E	1007	R0001005	EQ	.000000	.000000	.000000	.000000
E	1008	R0001006	EQ	.000000	.000000	.000000	.000000
	Number	Row	At	Value	Slack Value	Dual Value	RHS
E	1009	R0001007	EQ	.000000	.000000	.000000	.000000
E	1010	R0001008	EQ	.000000	.000000	.000000	.000000
E	1011	R0001009	EQ	.000000	.000000	.000000	.000000
E	1012	R0001010	EQ	.000000	.000000	.000000	.000000
E	1013	R0001011	EQ	.000000	.000000	.000000	.000000
E	1014	R0001012	EQ	.000000	.000000	.000000	.000000
E	1015	R0001013	EQ	.000000	.000000	.000000	.000000
E	1016	R0001014	EQ	.000000	.000000	.000000	.000000
E	1017	R0001015	EQ	.000000	.000000	.000000	.000000
E	1018	R0001016	EQ	.000000	.000000	.000000	.000000
E	1019	R0001017	EQ	.000000	.000000	.000000	.000000
E	1020	R0001018	EQ	.000000	.000000	.000000	.000000
E	1021	R0001019	EQ	.000000	.000000	.000000	.000000
E	1022	R0001020	EQ	.000000	.000000	.000000	.000000
E	1023	R0001021	EQ	.000000	.000000	.000000	.000000
E	1024	R0001022	EQ	.000000	.000000	.000000	.000000
E	1025	R0001023	EQ	.000000	.000000	.000000	.000000
E	1026	R0001024	EQ	.000000	.000000	.000000	.000000
E	1027	R0001025	EQ	.000000	.000000	.000000	.000000
E	1028	R0001026	EQ	.000000	.000000	.000000	.000000
E	1029	R0001027	EQ	.000000	.000000	.000000	.000000
	Number	Row	At	Value	Slack Value	Dual Value	RHS
E	1030	R0001028	EQ	.000000	.000000	.000000	.000000
E	1031	R0001029	EQ	.000000	.000000	.000000	.000000
E	1032	R0001030	EQ	.000000	.000000	.000000	.000000
E	1033	R0001031	EQ	.000000	.000000	.000000	.000000
E	1034	R0001032	EQ	.000000	.000000	.000000	.000000
E	1035	R0001033	EQ	.000000	.000000	.000000	.000000
E	1036	R0001034	EQ	.000000	.000000	.000000	.000000
E	1037	R0001035	EQ	.000000	.000000	.000000	.000000
E	1038	R0001036	EQ	.000000	.000000	.000000	.000000
E	1039	R0001037	EQ	.000000	.000000	.000000	.000000
E	1040	R0001038	EQ	.000000	.000000	.000000	.000000
E	1041	R0001039	EQ	.000000	.000000	.000000	.000000
E	1042	R0001040	EQ	.000000	.000000	.000000	.000000
E	1043	R0001041	EQ	.000000	.000000	.000000	.000000
E	1044	R0001042	EQ	.000000	.000000	.000000	.000000
E	1045	R0001043	EQ	.000000	.000000	.000000	.000000
E	1046	R0001044	EQ	.000000	.000000	.000000	.000000
E	1047	R0001045	EQ	.000000	.000000	.000000	.000000
E	1048	R0001046	EQ	.000000	.000000	.000000	.000000
E	1049	R0001047	EQ	.000000	.000000	.000000	.000000
E	1050	R0001048	EQ	.000000	.000000	.000000	.000000
	Number	Row	At	Value	Slack Value	Dual Value	RHS
E	1051	R0001049	EQ	.000000	.000000	.000000	.000000
E	1052	R0001050	EQ	.000000	.000000	.000000	.000000
E	1053	R0001051	EQ	.000000	.000000	.000000	.000000
E	1054	R0001052	EQ	.000000	.000000	.000000	.000000
E	1055	R0001053	EQ	.000000	.000000	.000000	.000000
E	1056	R0001054	EQ	.000000	.000000	.000000	.000000
E	1057	R0001055	EQ	.000000	.000000	.000000	.000000
E	1058	R0001056	EQ	.000000	.000000	.000000	.000000
E	1059	R0001057	EQ	.000000	.000000	.000000	.000000
E	1060	R0001058	EQ	.000000	.000000	.000000	.000000
E	1061	R0001059	EQ	.000000	.000000	.000000	.000000
E	1062	R0001060	EQ	.000000	.000000	.000000	.000000
E	1063	R0001061	EQ	.000000	.000000	.000000	.000000
E	1064	R0001062	EQ	.000000	.000000	.000000	.000000
E	1065	R0001063	EQ	.000000	.000000	.000000	.000000
E	1066	R0001064	EQ	.000000	.000000	.000000	.000000

E	1067	R0001065	EQ	.000000	.000000	.000000	.000000
E	1068	R0001066	EQ	.000000	.000000	.000000	.000000
E	1069	R0001067	EQ	.000000	.000000	.000000	.000000
E	1070	R0001068	EQ	.000000	.000000	.000000	.000000
E	1071	R0001069	EQ	.000000	.000000	.000000	.000000
	Number	Row	At	Value	Slack Value	Dual Value	RHS
E	1072	R0001070	EQ	.000000	.000000	.000000	.000000
E	1073	R0001071	EQ	.000000	.000000	.000000	.000000
E	1074	R0001072	EQ	.000000	.000000	.000000	.000000
E	1075	R0001073	EQ	.000000	.000000	.000000	.000000
E	1076	R0001074	EQ	.000000	.000000	.000000	.000000
E	1077	R0001075	EQ	.000000	.000000	.000000	.000000
E	1078	R0001076	EQ	.000000	.000000	.000000	.000000
E	1079	R0001077	EQ	.000000	.000000	.000000	.000000
E	1080	R0001078	EQ	.000000	.000000	.000000	.000000
E	1081	R0001079	EQ	.000000	.000000	.000000	.000000
E	1082	R0001080	EQ	.000000	.000000	.000000	.000000
E	1083	R0001081	EQ	.000000	.000000	.000000	.000000
E	1084	R0001082	EQ	.000000	.000000	.000000	.000000
E	1085	R0001083	EQ	.000000	.000000	.000000	.000000
E	1086	R0001084	EQ	.000000	.000000	.000000	.000000
E	1087	R0001085	EQ	.000000	.000000	.000000	.000000
E	1088	R0001086	EQ	.000000	.000000	.000000	.000000
E	1089	R0001087	EQ	.000000	.000000	.000000	.000000
E	1090	R0001088	EQ	.000000	.000000	.000000	.000000
E	1091	R0001089	EQ	.000000	.000000	.000000	.000000
E	1092	R0001090	EQ	.000000	.000000	.000000	.000000
	Number	Row	At	Value	Slack Value	Dual Value	RHS
E	1093	R0001091	EQ	.000000	.000000	.000000	.000000
E	1094	R0001092	EQ	.000000	.000000	.000000	.000000
E	1095	R0001093	EQ	.000000	.000000	.000000	.000000
E	1096	R0001094	EQ	.000000	.000000	.000000	.000000
E	1097	R0001095	EQ	.000000	.000000	.000000	.000000
E	1098	R0001096	EQ	.000000	.000000	.000000	.000000
E	1099	R0001097	EQ	.000000	.000000	.000000	.000000
E	1100	R0001098	EQ	.000000	.000000	.000000	.000000
E	1101	R0001099	EQ	.000000	.000000	.000000	.000000
E	1102	R0001100	EQ	.000000	.000000	.000000	.000000
E	1103	R0001101	EQ	.000000	.000000	.000000	.000000
E	1104	R0001102	EQ	.000000	.000000	.000000	.000000
E	1105	R0001103	EQ	.000000	.000000	.000000	.000000
E	1106	R0001104	EQ	.000000	.000000	.000000	.000000
E	1107	R0001105	EQ	.000000	.000000	.000000	.000000
E	1108	R0001106	EQ	.000000	.000000	.000000	.000000
E	1109	R0001107	EQ	.000000	.000000	.000000	.000000
E	1110	R0001108	EQ	.000000	.000000	.000000	.000000
E	1111	R0001109	EQ	.000000	.000000	.000000	.000000
E	1112	R0001110	EQ	.000000	.000000	.000000	.000000
E	1113	R0001111	EQ	.000000	.000000	.000000	.000000
	Number	Row	At	Value	Slack Value	Dual Value	RHS
E	1114	R0001112	EQ	.000000	.000000	.000000	.000000
E	1115	R0001113	EQ	.000000	.000000	.000000	.000000
E	1116	R0001114	EQ	.000000	.000000	.000000	.000000
E	1117	R0001115	EQ	.000000	.000000	.000000	.000000
E	1118	R0001116	EQ	.000000	.000000	.000000	.000000
E	1119	R0001117	EQ	.000000	.000000	.000000	.000000
E	1120	R0001118	EQ	.000000	.000000	.000000	.000000
E	1121	R0001119	EQ	.000000	.000000	.000000	.000000
E	1122	R0001120	EQ	.000000	.000000	.000000	.000000
E	1123	R0001121	EQ	.000000	.000000	.000000	.000000
E	1124	R0001122	EQ	.000000	.000000	.000000	.000000
E	1125	R0001123	EQ	.000000	.000000	.000000	.000000
E	1126	R0001124	EQ	.000000	.000000	.000000	.000000
E	1127	R0001125	EQ	.000000	.000000	.000000	.000000
E	1128	R0001126	EQ	.000000	.000000	.000000	.000000
E	1129	R0001127	EQ	.000000	.000000	.000000	.000000
E	1130	R0001128	EQ	.000000	.000000	.000000	.000000
E	1131	R0001129	EQ	.000000	.000000	.000000	.000000
E	1132	R0001130	EQ	.000000	.000000	.000000	.000000
E	1133	R0001131	EQ	.000000	.000000	.000000	.000000
E	1134	R0001132	EQ	.000000	.000000	.000000	.000000

	Number	Row	At	Value	Slack Value	Dual Value	RHS
E	1135	R0001133	EQ	.000000	.000000	.000000	.000000
E	1136	R0001134	EQ	.000000	.000000	.000000	.000000
E	1137	R0001135	EQ	.000000	.000000	.000000	.000000
E	1138	R0001136	EQ	.000000	.000000	.000000	.000000
E	1139	R0001137	EQ	.000000	.000000	.000000	.000000
E	1140	R0001138	EQ	.000000	.000000	.000000	.000000
E	1141	R0001139	EQ	.000000	.000000	.000000	.000000
E	1142	R0001140	EQ	.000000	.000000	.000000	.000000
E	1143	R0001141	EQ	.000000	.000000	.000000	.000000
E	1144	R0001142	EQ	.000000	.000000	.000000	.000000
E	1145	R0001143	EQ	.000000	.000000	.000000	.000000
E	1146	R0001144	EQ	.000000	.000000	.000000	.000000
E	1147	R0001145	EQ	.000000	.000000	.000000	.000000
E	1148	R0001146	EQ	.000000	.000000	.000000	.000000
E	1149	R0001147	EQ	.000000	.000000	.000000	.000000
E	1150	R0001148	EQ	.000000	.000000	.000000	.000000
E	1151	R0001149	EQ	.000000	.000000	.000000	.000000
E	1152	R0001150	EQ	.000000	.000000	.000000	.000000
E	1153	R0001151	EQ	.000000	.000000	.000000	.000000
E	1154	R0001152	EQ	.000000	.000000	.000000	.000000
E	1155	R0001153	EQ	.000000	.000000	.000000	.000000
	Number	Row	At	Value	Slack Value	Dual Value	RHS
E	1156	R0001154	EQ	.000000	.000000	.000000	.000000
E	1157	R0001155	EQ	.000000	.000000	.000000	.000000
E	1158	R0001156	EQ	.000000	.000000	.000000	.000000
E	1159	R0001157	EQ	.000000	.000000	.000000	.000000
E	1160	R0001158	EQ	.000000	.000000	.000000	.000000
E	1161	R0001159	EQ	.000000	.000000	.000000	.000000
E	1162	R0001160	EQ	.000000	.000000	.000000	.000000
E	1163	R0001161	EQ	.000000	.000000	.000000	.000000
E	1164	R0001162	EQ	.000000	.000000	.000000	.000000
E	1165	R0001163	EQ	.000000	.000000	.000000	.000000
E	1166	R0001164	EQ	.000000	.000000	.000000	.000000
E	1167	R0001165	EQ	.000000	.000000	.000000	.000000
E	1168	R0001166	EQ	.000000	.000000	.000000	.000000
E	1169	R0001167	EQ	.000000	.000000	.000000	.000000
E	1170	R0001168	EQ	.000000	.000000	.000000	.000000
E	1171	R0001169	EQ	.000000	.000000	.000000	.000000
E	1172	R0001170	EQ	.000000	.000000	.000000	.000000
E	1173	R0001171	EQ	.000000	.000000	.000000	.000000
E	1174	R0001172	EQ	.000000	.000000	.000000	.000000
E	1175	R0001173	EQ	.000000	.000000	.000000	.000000
E	1176	R0001174	EQ	.000000	.000000	.000000	.000000
	Number	Row	At	Value	Slack Value	Dual Value	RHS
E	1177	R0001175	EQ	.000000	.000000	.000000	.000000
E	1178	R0001176	EQ	.000000	.000000	.000000	.000000
E	1179	R0001177	EQ	.000000	.000000	.000000	.000000
E	1180	R0001178	EQ	.000000	.000000	.000000	.000000
E	1181	R0001179	EQ	.000000	.000000	.000000	.000000
E	1182	R0001180	EQ	.000000	.000000	.000000	.000000
E	1183	R0001181	EQ	.000000	.000000	.000000	.000000
E	1184	R0001182	EQ	.000000	.000000	.000000	.000000
E	1185	R0001183	EQ	.000000	.000000	.000000	.000000
E	1186	R0001184	EQ	.000000	.000000	.000000	.000000
E	1187	R0001185	EQ	.000000	.000000	.000000	.000000
E	1188	R0001186	EQ	.000000	.000000	.000000	.000000
E	1189	R0001187	EQ	.000000	.000000	.000000	.000000
E	1190	R0001188	EQ	.000000	.000000	.000000	.000000
E	1191	R0001189	EQ	.000000	.000000	.000000	.000000
E	1192	R0001190	EQ	.000000	.000000	.000000	.000000
E	1193	R0001191	EQ	.000000	.000000	.000000	.000000
E	1194	R0001192	EQ	.000000	.000000	.000000	.000000
E	1195	R0001193	EQ	.000000	.000000	.000000	.000000
E	1196	R0001194	EQ	.000000	.000000	.000000	.000000
E	1197	R0001195	EQ	.000000	.000000	.000000	.000000
	Number	Row	At	Value	Slack Value	Dual Value	RHS
E	1198	R0001196	EQ	.000000	.000000	.000000	.000000
E	1199	R0001197	EQ	.000000	.000000	.000000	.000000
E	1200	R0001198	EQ	.000000	.000000	.000000	.000000
E	1201	R0001199	EQ	.000000	.000000	.000000	.000000

E	1202	R0001200	EQ	.000000	.000000	.000000	.000000
E	1203	R0001201	EQ	.000000	.000000	.000000	.000000
E	1204	R0001202	EQ	.000000	.000000	.000000	.000000
E	1205	R0001203	EQ	.000000	.000000	.000000	.000000
E	1206	R0001204	EQ	.000000	.000000	.000000	.000000
E	1207	R0001205	EQ	.000000	.000000	.000000	.000000
E	1208	R0001206	EQ	.000000	.000000	.000000	.000000
E	1209	R0001207	EQ	.000000	.000000	.000000	.000000
E	1210	R0001208	EQ	.000000	.000000	.000000	.000000
E	1211	R0001209	EQ	.000000	.000000	.000000	.000000
E	1212	R0001210	EQ	.000000	.000000	.000000	.000000
E	1213	R0001211	EQ	.000000	.000000	.000000	.000000
E	1214	R0001212	EQ	.000000	.000000	.000000	.000000
E	1215	R0001213	EQ	.000000	.000000	.000000	.000000
E	1216	R0001214	EQ	.000000	.000000	.000000	.000000
E	1217	R0001215	EQ	.000000	.000000	.000000	.000000
E	1218	R0001216	EQ	.000000	.000000	.000000	.000000
	Number	Row	At	Value	Slack Value	Dual Value	RHS
E	1219	R0001217	EQ	.000000	.000000	.000000	.000000
E	1220	R0001218	EQ	.000000	.000000	.000000	.000000
E	1221	R0001219	EQ	.000000	.000000	.000000	.000000
E	1222	R0001220	EQ	.000000	.000000	.000000	.000000
E	1223	R0001221	EQ	.000000	.000000	.000000	.000000
E	1224	R0001222	EQ	.000000	.000000	.000000	.000000
E	1225	R0001223	EQ	.000000	.000000	.000000	.000000
E	1226	R0001224	EQ	.000000	.000000	.000000	.000000
E	1227	R0001225	EQ	.000000	.000000	.000000	.000000
E	1228	R0001226	EQ	.000000	.000000	.000000	.000000
E	1229	R0001227	EQ	.000000	.000000	.000000	.000000
E	1230	R0001228	EQ	.000000	.000000	.000000	.000000
E	1231	R0001229	EQ	.000000	.000000	.000000	.000000
E	1232	R0001230	EQ	.000000	.000000	.000000	.000000
E	1233	R0001231	EQ	.000000	.000000	.000000	.000000
E	1234	R0001232	EQ	.000000	.000000	.000000	.000000
E	1235	R0001233	EQ	.000000	.000000	.000000	.000000
E	1236	R0001234	EQ	.000000	.000000	.000000	.000000
E	1237	R0001235	EQ	.000000	.000000	.000000	.000000
E	1238	R0001236	EQ	.000000	.000000	.000000	.000000
E	1239	R0001237	EQ	.000000	.000000	.000000	.000000
	Number	Row	At	Value	Slack Value	Dual Value	RHS
E	1240	R0001238	EQ	.000000	.000000	.000000	.000000
E	1241	R0001239	EQ	.000000	.000000	.000000	.000000
E	1242	R0001240	EQ	.000000	.000000	.000000	.000000
E	1243	R0001241	EQ	.000000	.000000	.000000	.000000
E	1244	R0001242	EQ	.000000	.000000	.000000	.000000
E	1245	R0001243	EQ	.000000	.000000	.000000	.000000
E	1246	R0001244	EQ	.000000	.000000	.000000	.000000
E	1247	R0001245	EQ	.000000	.000000	.000000	.000000
E	1248	R0001246	EQ	.000000	.000000	.000000	.000000
E	1249	R0001247	EQ	.000000	.000000	.000000	.000000
E	1250	R0001248	EQ	.000000	.000000	.000000	.000000
E	1251	R0001249	EQ	.000000	.000000	.000000	.000000
E	1252	R0001250	EQ	.000000	.000000	.000000	.000000
E	1253	R0001251	EQ	.000000	.000000	.000000	.000000
E	1254	R0001252	EQ	.000000	.000000	.000000	.000000
E	1255	R0001253	EQ	.000000	.000000	.000000	.000000
E	1256	R0001254	EQ	.000000	.000000	.000000	.000000
E	1257	R0001255	EQ	.000000	.000000	.000000	.000000
E	1258	R0001256	EQ	.000000	.000000	.000000	.000000
E	1259	R0001257	EQ	.000000	.000000	.000000	.000000
E	1260	R0001258	EQ	.000000	.000000	.000000	.000000
	Number	Row	At	Value	Slack Value	Dual Value	RHS
E	1261	R0001259	EQ	.000000	.000000	.000000	.000000
E	1262	R0001260	EQ	.000000	.000000	.000000	.000000
E	1263	R0001261	EQ	.000000	.000000	.000000	.000000
E	1264	R0001262	EQ	.000000	.000000	.000000	.000000
E	1265	R0001263	EQ	.000000	.000000	.000000	.000000
E	1266	R0001264	EQ	.000000	.000000	.000000	.000000
E	1267	R0001265	EQ	.000000	.000000	.000000	.000000
E	1268	R0001266	EQ	.000000	.000000	.000000	.000000
E	1269	R0001267	EQ	.000000	.000000	.000000	.000000

E	1270	R0001268	EQ	.000000	.000000	.000000	.000000
E	1271	R0001269	EQ	.000000	.000000	.000000	.000000
E	1272	R0001270	EQ	.000000	.000000	.000000	.000000
E	1273	R0001271	EQ	.000000	.000000	.000000	.000000
E	1274	R0001272	EQ	.000000	.000000	.000000	.000000
E	1275	R0001273	EQ	.000000	.000000	.000000	.000000
E	1276	R0001274	EQ	.000000	.000000	.000000	.000000
E	1277	R0001275	EQ	.000000	.000000	.000000	.000000
E	1278	R0001276	EQ	.000000	.000000	.000000	.000000
E	1279	R0001277	EQ	.000000	.000000	.000000	.000000
E	1280	R0001278	EQ	.000000	.000000	.000000	.000000
E	1281	R0001279	EQ	.000000	.000000	.000000	.000000
	Number	Row	At	Value	Slack Value	Dual Value	RHS
E	1282	R0001280	EQ	.000000	.000000	.000000	.000000
E	1283	R0001281	EQ	.000000	.000000	.000000	.000000
E	1284	R0001282	EQ	.000000	.000000	.000000	.000000
E	1285	R0001283	EQ	.000000	.000000	.000000	.000000
E	1286	R0001284	EQ	.000000	.000000	.000000	.000000
E	1287	R0001285	EQ	.000000	.000000	.000000	.000000
E	1288	R0001286	EQ	.000000	.000000	.000000	.000000
E	1289	R0001287	EQ	.000000	.000000	.000000	.000000
E	1290	R0001288	EQ	.000000	.000000	.000000	.000000
E	1291	R0001289	EQ	.000000	.000000	.000000	.000000
E	1292	R0001290	EQ	.000000	.000000	.000000	.000000
E	1293	R0001291	EQ	.000000	.000000	.000000	.000000
E	1294	R0001292	EQ	.000000	.000000	.000000	.000000
E	1295	R0001293	EQ	.000000	.000000	.000000	.000000
E	1296	R0001294	EQ	.000000	.000000	.000000	.000000
E	1297	R0001295	EQ	.000000	.000000	.000000	.000000
E	1298	R0001296	EQ	.000000	.000000	.000000	.000000
E	1299	R0001297	EQ	.000000	.000000	.000000	.000000
E	1300	R0001298	EQ	.000000	.000000	.000000	.000000
E	1301	R0001299	EQ	.000000	.000000	.000000	.000000
E	1302	R0001300	EQ	.000000	.000000	.000000	.000000
	Number	Row	At	Value	Slack Value	Dual Value	RHS
E	1303	R0001301	EQ	.000000	.000000	.000000	.000000
E	1304	R0001302	EQ	.000000	.000000	.000000	.000000
E	1305	R0001303	EQ	.000000	.000000	.000000	.000000
E	1306	R0001304	EQ	.000000	.000000	.000000	.000000
E	1307	R0001305	EQ	.000000	.000000	.000000	.000000
E	1308	R0001306	EQ	.000000	.000000	.000000	.000000
E	1309	R0001307	EQ	.000000	.000000	.000000	.000000
E	1310	R0001308	EQ	.000000	.000000	.000000	.000000
E	1311	R0001309	EQ	.000000	.000000	.000000	.000000
E	1312	R0001310	EQ	.000000	.000000	.000000	.000000
E	1313	R0001311	EQ	.000000	.000000	.000000	.000000
E	1314	R0001312	EQ	.000000	.000000	.000000	.000000
E	1315	R0001313	EQ	.000000	.000000	.000000	.000000
E	1316	R0001314	EQ	.000000	.000000	.000000	.000000
E	1317	R0001315	EQ	.000000	.000000	.000000	.000000
E	1318	R0001316	EQ	.000000	.000000	.000000	.000000
E	1319	R0001317	EQ	.000000	.000000	.000000	.000000
E	1320	R0001318	EQ	.000000	.000000	.000000	.000000
E	1321	R0001319	EQ	.000000	.000000	.000000	.000000
E	1322	R0001320	EQ	.000000	.000000	.000000	.000000
E	1323	R0001321	EQ	.000000	.000000	.000000	.000000
	Number	Row	At	Value	Slack Value	Dual Value	RHS
E	1324	R0001322	EQ	.000000	.000000	.000000	.000000
E	1325	R0001323	EQ	.000000	.000000	.000000	.000000
E	1326	R0001324	EQ	.000000	.000000	.000000	.000000
E	1327	R0001325	EQ	.000000	.000000	.000000	.000000
E	1328	R0001326	EQ	.000000	.000000	.000000	.000000
E	1329	R0001327	EQ	.000000	.000000	.000000	.000000
E	1330	R0001328	EQ	.000000	.000000	.000000	.000000
E	1331	R0001329	EQ	.000000	.000000	.000000	.000000
E	1332	R0001330	EQ	.000000	.000000	.000000	.000000
E	1333	R0001331	EQ	.000000	.000000	.000000	.000000
E	1334	R0001332	EQ	.000000	.000000	.000000	.000000
E	1335	R0001333	EQ	.000000	.000000	.000000	.000000
E	1336	R0001334	EQ	.000000	.000000	.000000	.000000
E	1337	R0001335	EQ	.000000	.000000	.000000	.000000

E	1338	R0001336	EQ	.000000	.000000	.000000	.000000
E	1339	R0001337	EQ	.000000	.000000	.000000	.000000
E	1340	R0001338	EQ	.000000	.000000	.000000	.000000
E	1341	R0001339	EQ	.000000	.000000	.000000	.000000
E	1342	R0001340	EQ	.000000	.000000	.000000	.000000
E	1343	R0001341	EQ	.000000	.000000	.000000	.000000
E	1344	R0001342	EQ	.000000	.000000	.000000	.000000
	Number	Row	At	Value	Slack Value	Dual Value	RHS
E	1345	R0001343	EQ	.000000	.000000	.000000	.000000
E	1346	R0001344	EQ	.000000	.000000	.000000	.000000
E	1347	R0001345	EQ	.000000	.000000	.000000	.000000
E	1348	R0001346	EQ	.000000	.000000	.000000	.000000
E	1349	R0001347	EQ	.000000	.000000	.000000	.000000
E	1350	R0001348	EQ	.000000	.000000	.000000	.000000
E	1351	R0001349	EQ	.000000	.000000	.000000	.000000
E	1352	R0001350	EQ	.000000	.000000	.000000	.000000
E	1353	R0001351	EQ	.000000	.000000	.000000	.000000
E	1354	R0001352	EQ	.000000	.000000	.000000	.000000
E	1355	R0001353	EQ	.000000	.000000	.000000	.000000
E	1356	R0001354	EQ	.000000	.000000	.000000	.000000
E	1357	R0001355	EQ	.000000	.000000	.000000	.000000
E	1358	R0001356	EQ	.000000	.000000	.000000	.000000
E	1359	R0001357	EQ	.000000	.000000	.000000	.000000
E	1360	R0001358	EQ	.000000	.000000	.000000	.000000
E	1361	R0001359	EQ	.000000	.000000	.000000	.000000
E	1362	R0001360	EQ	.000000	.000000	.000000	.000000
E	1363	R0001361	EQ	.000000	.000000	.000000	.000000
E	1364	R0001362	EQ	.000000	.000000	.000000	.000000
E	1365	R0001363	EQ	.000000	.000000	.000000	.000000
	Number	Row	At	Value	Slack Value	Dual Value	RHS
E	1366	R0001364	EQ	.000000	.000000	.000000	.000000
E	1367	R0001365	EQ	.000000	.000000	.000000	.000000
E	1368	R0001366	EQ	.000000	.000000	.000000	.000000
E	1369	R0001367	EQ	.000000	.000000	.000000	.000000
E	1370	R0001368	EQ	.000000	.000000	.000000	.000000
E	1371	R0001369	EQ	.000000	.000000	.000000	.000000
E	1372	R0001370	EQ	.000000	.000000	.000000	.000000
E	1373	R0001371	EQ	.000000	.000000	.000000	.000000
E	1374	R0001372	EQ	.000000	.000000	.000000	.000000
E	1375	R0001373	EQ	.000000	.000000	.000000	.000000
E	1376	R0001374	EQ	.000000	.000000	.000000	.000000
E	1377	R0001375	EQ	.000000	.000000	.000000	.000000
E	1378	R0001376	EQ	.000000	.000000	.000000	.000000
E	1379	R0001377	EQ	.000000	.000000	.000000	.000000
E	1380	R0001378	EQ	.000000	.000000	.000000	.000000
E	1381	R0001379	EQ	.000000	.000000	.000000	.000000
E	1382	R0001380	EQ	.000000	.000000	.000000	.000000
E	1383	R0001381	EQ	.000000	.000000	.000000	.000000
E	1384	R0001382	EQ	.000000	.000000	.000000	.000000
E	1385	R0001383	EQ	.000000	.000000	.000000	.000000
E	1386	R0001384	EQ	.000000	.000000	.000000	.000000
	Number	Row	At	Value	Slack Value	Dual Value	RHS
E	1387	R0001385	EQ	.000000	.000000	.000000	.000000
E	1388	R0001386	EQ	.000000	.000000	.000000	.000000
E	1389	R0001387	EQ	.000000	.000000	.000000	.000000
E	1390	R0001388	EQ	.000000	.000000	.000000	.000000
E	1391	R0001389	EQ	.000000	.000000	.000000	.000000
E	1392	R0001390	EQ	.000000	.000000	.000000	.000000
E	1393	R0001391	EQ	.000000	.000000	.000000	.000000
E	1394	R0001392	EQ	.000000	.000000	.000000	.000000
E	1395	R0001393	EQ	.000000	.000000	.000000	.000000
E	1396	R0001394	EQ	.000000	.000000	.000000	.000000
E	1397	R0001395	EQ	.000000	.000000	.000000	.000000
E	1398	R0001396	EQ	.000000	.000000	.000000	.000000
E	1399	R0001397	EQ	.000000	.000000	.000000	.000000
E	1400	R0001398	EQ	.000000	.000000	.000000	.000000
E	1401	R0001399	EQ	.000000	.000000	.000000	.000000
E	1402	R0001400	EQ	.000000	.000000	.000000	.000000
E	1403	R0001401	EQ	.000000	.000000	.000000	.000000
E	1404	R0001402	EQ	.000000	.000000	.000000	.000000
E	1405	R0001403	EQ	.000000	.000000	.000000	.000000

E	1406	R0001404	EQ	.000000	.000000	.000000	.000000
E	1407	R0001405	EQ	.000000	.000000	.000000	.000000
	Number	Row	At	Value	Slack Value	Dual Value	RHS
E	1408	R0001406	EQ	.000000	.000000	.000000	.000000
E	1409	R0001407	EQ	.000000	.000000	.000000	.000000
E	1410	R0001408	EQ	.000000	.000000	.000000	.000000
E	1411	R0001409	EQ	.000000	.000000	.000000	.000000
E	1412	R0001410	EQ	.000000	.000000	.000000	.000000
E	1413	R0001411	EQ	.000000	.000000	.000000	.000000
E	1414	R0001412	EQ	.000000	.000000	.000000	.000000
E	1415	R0001413	EQ	.000000	.000000	.000000	.000000
E	1416	R0001414	EQ	.000000	.000000	.000000	.000000
E	1417	R0001415	EQ	.000000	.000000	.000000	.000000
E	1418	R0001416	EQ	.000000	.000000	.000000	.000000
E	1419	R0001417	EQ	.000000	.000000	.000000	.000000
E	1420	R0001418	EQ	.000000	.000000	.000000	.000000
E	1421	R0001419	EQ	.000000	.000000	.000000	.000000
E	1422	R0001420	EQ	.000000	.000000	.000000	.000000
E	1423	R0001421	EQ	.000000	.000000	.000000	.000000
E	1424	R0001422	EQ	.000000	.000000	.000000	.000000
E	1425	R0001423	EQ	.000000	.000000	.000000	.000000
E	1426	R0001424	EQ	.000000	.000000	.000000	.000000
E	1427	R0001425	EQ	.000000	.000000	.000000	.000000
E	1428	R0001426	EQ	.000000	.000000	.000000	.000000
	Number	Row	At	Value	Slack Value	Dual Value	RHS
E	1429	R0001427	EQ	.000000	.000000	.000000	.000000
E	1430	R0001428	EQ	.000000	.000000	.000000	.000000
E	1431	R0001429	EQ	.000000	.000000	.000000	.000000
E	1432	R0001430	EQ	.000000	.000000	.000000	.000000
E	1433	R0001431	EQ	.000000	.000000	.000000	.000000
E	1434	R0001432	EQ	.000000	.000000	.000000	.000000
E	1435	R0001433	EQ	.000000	.000000	.000000	.000000
E	1436	R0001434	EQ	.000000	.000000	.000000	.000000
E	1437	R0001435	EQ	.000000	.000000	.000000	.000000
E	1438	R0001436	EQ	.000000	.000000	.000000	.000000
E	1439	R0001437	EQ	.000000	.000000	.000000	.000000
E	1440	R0001438	EQ	.000000	.000000	.000000	.000000
E	1441	R0001439	EQ	.000000	.000000	.000000	.000000
E	1442	R0001440	EQ	.000000	.000000	.000000	.000000
E	1443	R0001441	EQ	.000000	.000000	.000000	.000000
E	1444	R0001442	EQ	.000000	.000000	.000000	.000000
E	1445	R0001443	EQ	.000000	.000000	.000000	.000000
E	1446	R0001444	EQ	.000000	.000000	.000000	.000000
E	1447	R0001445	EQ	.000000	.000000	.000000	.000000
E	1448	R0001446	EQ	.000000	.000000	.000000	.000000
E	1449	R0001447	EQ	.000000	.000000	.000000	.000000
	Number	Row	At	Value	Slack Value	Dual Value	RHS
E	1450	R0001448	EQ	.000000	.000000	.000000	.000000
E	1451	R0001449	EQ	.000000	.000000	.000000	.000000
E	1452	R0001450	EQ	.000000	.000000	.000000	.000000
E	1453	R0001451	EQ	.000000	.000000	.000000	.000000
E	1454	R0001452	EQ	.000000	.000000	.000000	.000000
E	1455	R0001453	EQ	.000000	.000000	.000000	.000000
E	1456	R0001454	EQ	.000000	.000000	.000000	.000000
E	1457	R0001455	EQ	.000000	.000000	.000000	.000000
E	1458	R0001456	EQ	.000000	.000000	.000000	.000000
E	1459	R0001457	EQ	.000000	.000000	.000000	.000000
E	1460	R0001458	EQ	.000000	.000000	.000000	.000000
E	1461	R0001459	EQ	.000000	.000000	.000000	.000000
E	1462	R0001460	EQ	.000000	.000000	.000000	.000000
E	1463	R0001461	EQ	.000000	.000000	.000000	.000000
E	1464	R0001462	EQ	.000000	.000000	.000000	.000000
E	1465	R0001463	EQ	.000000	.000000	.000000	.000000
E	1466	R0001464	EQ	.000000	.000000	.000000	.000000
E	1467	R0001465	EQ	.000000	.000000	.000000	.000000
E	1468	R0001466	EQ	.000000	.000000	.000000	.000000
E	1469	R0001467	EQ	.000000	.000000	.000000	.000000
E	1470	R0001468	EQ	.000000	.000000	.000000	.000000
	Number	Row	At	Value	Slack Value	Dual Value	RHS
E	1471	R0001469	EQ	.000000	.000000	.000000	.000000
E	1472	R0001470	EQ	.000000	.000000	.000000	.000000

E	1473	R0001471	EQ	.000000	.000000	.000000	.000000
E	1474	R0001472	EQ	.000000	.000000	.000000	.000000
E	1475	R0001473	EQ	.000000	.000000	.000000	.000000
E	1476	R0001474	EQ	.000000	.000000	.000000	.000000
E	1477	R0001475	EQ	.000000	.000000	.000000	.000000
E	1478	R0001476	EQ	.000000	.000000	.000000	.000000
E	1479	R0001477	EQ	.000000	.000000	.000000	.000000
E	1480	R0001478	EQ	.000000	.000000	.000000	.000000
E	1481	R0001479	EQ	.000000	.000000	.000000	.000000
E	1482	R0001480	EQ	.000000	.000000	.000000	.000000
E	1483	R0001481	EQ	.000000	.000000	.000000	.000000
E	1484	R0001482	EQ	.000000	.000000	.000000	.000000
E	1485	R0001483	EQ	.000000	.000000	.000000	.000000
E	1486	R0001484	EQ	.000000	.000000	.000000	.000000
E	1487	R0001485	EQ	.000000	.000000	.000000	.000000
E	1488	R0001486	EQ	.000000	.000000	.000000	.000000
E	1489	R0001487	EQ	.000000	.000000	.000000	.000000
E	1490	R0001488	EQ	.000000	.000000	.000000	.000000
E	1491	R0001489	EQ	.000000	.000000	.000000	.000000

	Number	Row	At	Value	Slack Value	Dual Value	RHS
E	1492	R0001490	EQ	.000000	.000000	.000000	.000000
E	1493	R0001491	EQ	.000000	.000000	.000000	.000000
E	1494	R0001492	EQ	.000000	.000000	.000000	.000000
E	1495	R0001493	EQ	.000000	.000000	.000000	.000000
E	1496	R0001494	EQ	.000000	.000000	.000000	.000000
E	1497	R0001495	EQ	.000000	.000000	.000000	.000000
E	1498	R0001496	EQ	.000000	.000000	.000000	.000000
E	1499	R0001497	EQ	.000000	.000000	.000000	.000000
E	1500	R0001498	EQ	.000000	.000000	.000000	.000000
E	1501	R0001499	EQ	.000000	.000000	.000000	.000000
E	1502	R0001500	EQ	.000000	.000000	.000000	.000000
E	1503	R0001501	EQ	.000000	.000000	.000000	.000000
E	1504	R0001502	EQ	.000000	.000000	.000000	.000000
E	1505	R0001503	EQ	.000000	.000000	.000000	.000000
E	1506	R0001504	EQ	.000000	.000000	.000000	.000000
E	1507	R0001505	EQ	.000000	.000000	.000000	.000000
E	1508	R0001506	EQ	.000000	.000000	.000000	.000000
E	1509	R0001507	EQ	.000000	.000000	.000000	.000000
E	1510	R0001508	EQ	.000000	.000000	.000000	.000000
E	1511	R0001509	EQ	.000000	.000000	.000000	.000000
E	1512	R0001510	EQ	.000000	.000000	.000000	.000000

	Number	Row	At	Value	Slack Value	Dual Value	RHS
E	1513	R0001511	EQ	.000000	.000000	.000000	.000000
E	1514	R0001512	EQ	.000000	.000000	.000000	.000000
E	1515	R0001513	EQ	.000000	.000000	.000000	.000000
E	1516	R0001514	EQ	.000000	.000000	.000000	.000000
E	1517	R0001515	EQ	.000000	.000000	.000000	.000000
E	1518	R0001516	EQ	.000000	.000000	.000000	.000000
E	1519	R0001517	EQ	.000000	.000000	.000000	.000000
E	1520	R0001518	EQ	.000000	.000000	.000000	.000000
E	1521	R0001519	EQ	.000000	.000000	.000000	.000000
E	1522	R0001520	EQ	.000000	.000000	.000000	.000000
E	1523	R0001521	EQ	.000000	.000000	.000000	.000000
E	1524	R0001522	EQ	.000000	.000000	.000000	.000000
E	1525	R0001523	EQ	.000000	.000000	.000000	.000000
E	1526	R0001524	EQ	.000000	.000000	.000000	.000000
E	1527	R0001525	EQ	.000000	.000000	.000000	.000000
E	1528	R0001526	EQ	.000000	.000000	.000000	.000000
E	1529	R0001527	EQ	.000000	.000000	.000000	.000000
E	1530	R0001528	EQ	.000000	.000000	.000000	.000000
E	1531	R0001529	EQ	.000000	.000000	.000000	.000000
E	1532	R0001530	EQ	.000000	.000000	.000000	.000000
E	1533	R0001531	EQ	.000000	.000000	.000000	.000000

	Number	Row	At	Value	Slack Value	Dual Value	RHS
E	1534	R0001532	EQ	.000000	.000000	.000000	.000000
E	1535	R0001533	EQ	.000000	.000000	.000000	.000000
E	1536	R0001534	EQ	.000000	.000000	.000000	.000000
E	1537	R0001535	EQ	.000000	.000000	.000000	.000000
E	1538	R0001536	EQ	.000000	.000000	.000000	.000000
E	1539	R0001537	EQ	.000000	.000000	.000000	.000000
E	1540	R0001538	EQ	.000000	.000000	.000000	.000000

E	1541	R0001539	EQ	.000000	.000000	.000000	.000000
E	1542	R0001540	EQ	.000000	.000000	.000000	.000000
E	1543	R0001541	EQ	.000000	.000000	.000000	.000000
E	1544	R0001542	EQ	.000000	.000000	.000000	.000000
E	1545	R0001543	EQ	.000000	.000000	.000000	.000000
E	1546	R0001544	EQ	.000000	.000000	.000000	.000000
E	1547	R0001545	EQ	.000000	.000000	.000000	.000000
E	1548	R0001546	EQ	.000000	.000000	.000000	.000000
E	1549	R0001547	EQ	.000000	.000000	.000000	.000000
E	1550	R0001548	EQ	.000000	.000000	.000000	.000000
E	1551	R0001549	EQ	.000000	.000000	.000000	.000000
E	1552	R0001550	EQ	.000000	.000000	.000000	.000000
E	1553	R0001551	EQ	.000000	.000000	.000000	.000000
E	1554	R0001552	EQ	.000000	.000000	.000000	.000000
	Number	Row	At	Value	Slack Value	Dual Value	RHS
E	1555	R0001553	EQ	.000000	.000000	.000000	.000000
E	1556	R0001554	EQ	.000000	.000000	.000000	.000000
E	1557	R0001555	EQ	.000000	.000000	.000000	.000000
E	1558	R0001556	EQ	.000000	.000000	.000000	.000000
E	1559	R0001557	EQ	.000000	.000000	.000000	.000000
E	1560	R0001558	EQ	.000000	.000000	.000000	.000000
E	1561	R0001559	EQ	.000000	.000000	.000000	.000000
E	1562	R0001560	EQ	.000000	.000000	.000000	.000000
E	1563	R0001561	EQ	.000000	.000000	.000000	.000000
E	1564	R0001562	EQ	.000000	.000000	.000000	.000000
E	1565	R0001563	EQ	.000000	.000000	.000000	.000000
E	1566	R0001564	EQ	.000000	.000000	.000000	.000000
E	1567	R0001565	EQ	.000000	.000000	.000000	.000000
E	1568	R0001566	EQ	.000000	.000000	.000000	.000000
E	1569	R0001567	EQ	.000000	.000000	.000000	.000000
E	1570	R0001568	EQ	.000000	.000000	.000000	.000000
E	1571	R0001569	EQ	.000000	.000000	.000000	.000000
E	1572	R0001570	EQ	.000000	.000000	.000000	.000000
E	1573	R0001571	EQ	.000000	.000000	.000000	.000000
E	1574	R0001572	EQ	.000000	.000000	.000000	.000000
E	1575	R0001573	EQ	.000000	.000000	.000000	.000000
	Number	Row	At	Value	Slack Value	Dual Value	RHS
E	1576	R0001574	EQ	.000000	.000000	.000000	.000000
E	1577	R0001575	EQ	.000000	.000000	.000000	.000000
E	1578	R0001576	EQ	.000000	.000000	.000000	.000000
E	1579	R0001577	EQ	.000000	.000000	.000000	.000000
E	1580	R0001578	EQ	.000000	.000000	.000000	.000000
E	1581	R0001579	EQ	.000000	.000000	.000000	.000000
E	1582	R0001580	EQ	.000000	.000000	.000000	.000000
E	1583	R0001581	EQ	.000000	.000000	.000000	.000000
E	1584	R0001582	EQ	.000000	.000000	.000000	.000000
E	1585	R0001583	EQ	.000000	.000000	.000000	.000000
E	1586	R0001584	EQ	.000000	.000000	.000000	.000000
E	1587	R0001585	EQ	.000000	.000000	.000000	.000000
E	1588	R0001586	EQ	.000000	.000000	.000000	.000000
E	1589	R0001587	EQ	.000000	.000000	.000000	.000000
E	1590	R0001588	EQ	.000000	.000000	.000000	.000000
E	1591	R0001589	EQ	.000000	.000000	.000000	.000000
E	1592	R0001590	EQ	.000000	.000000	.000000	.000000
E	1593	R0001591	EQ	.000000	.000000	.000000	.000000
E	1594	R0001592	EQ	.000000	.000000	.000000	.000000
E	1595	R0001593	EQ	.000000	.000000	.000000	.000000
E	1596	R0001594	EQ	.000000	.000000	.000000	.000000
	Number	Row	At	Value	Slack Value	Dual Value	RHS
E	1597	R0001595	EQ	.000000	.000000	.000000	.000000
E	1598	R0001596	EQ	.000000	.000000	.000000	.000000
E	1599	R0001597	EQ	.000000	.000000	.000000	.000000
E	1600	R0001598	EQ	.000000	.000000	.000000	.000000
E	1601	R0001599	EQ	.000000	.000000	.000000	.000000
E	1602	R0001600	EQ	.000000	.000000	.000000	.000000
E	1603	R0001601	EQ	.000000	.000000	.000000	.000000
E	1604	R0001602	EQ	.000000	.000000	.000000	.000000
E	1605	R0001603	EQ	.000000	.000000	.000000	.000000
E	1606	R0001604	EQ	.000000	.000000	.000000	.000000
E	1607	R0001605	EQ	.000000	.000000	.000000	.000000
E	1608	R0001606	EQ	.000000	.000000	.000000	.000000

E	1609	R0001607	EQ	.000000	.000000	.000000	.000000
E	1610	R0001608	EQ	.000000	.000000	.000000	.000000
E	1611	R0001609	EQ	.000000	.000000	.000000	.000000
E	1612	R0001610	EQ	.000000	.000000	.000000	.000000
E	1613	R0001611	EQ	.000000	.000000	.000000	.000000
E	1614	R0001612	EQ	.000000	.000000	.000000	.000000
E	1615	R0001613	EQ	.000000	.000000	.000000	.000000
E	1616	R0001614	EQ	.000000	.000000	.000000	.000000
E	1617	R0001615	EQ	.000000	.000000	.000000	.000000
	Number	Row	At	Value	Slack Value	Dual Value	RHS
E	1618	R0001616	EQ	.000000	.000000	.000000	.000000
E	1619	R0001617	EQ	.000000	.000000	.000000	.000000
E	1620	R0001618	EQ	.000000	.000000	.000000	.000000
E	1621	R0001619	EQ	.000000	.000000	.000000	.000000
E	1622	R0001620	EQ	.000000	.000000	.000000	.000000
E	1623	R0001621	EQ	.000000	.000000	.000000	.000000
E	1624	R0001622	EQ	.000000	.000000	.000000	.000000
E	1625	R0001623	EQ	.000000	.000000	.000000	.000000
E	1626	R0001624	EQ	.000000	.000000	.000000	.000000
E	1627	R0001625	EQ	.000000	.000000	.000000	.000000
E	1628	R0001626	EQ	.000000	.000000	.000000	.000000
E	1629	R0001627	EQ	.000000	.000000	.000000	.000000
E	1630	R0001628	EQ	.000000	.000000	.000000	.000000
E	1631	R0001629	EQ	.000000	.000000	.000000	.000000
E	1632	R0001630	EQ	.000000	.000000	.000000	.000000
E	1633	R0001631	EQ	.000000	.000000	.000000	.000000
E	1634	R0001632	EQ	.000000	.000000	.000000	.000000
E	1635	R0001633	EQ	.000000	.000000	.000000	.000000
E	1636	R0001634	EQ	.000000	.000000	.000000	.000000
E	1637	R0001635	EQ	.000000	.000000	.000000	.000000
E	1638	R0001636	EQ	.000000	.000000	.000000	.000000
	Number	Row	At	Value	Slack Value	Dual Value	RHS
E	1639	R0001637	EQ	.000000	.000000	.000000	.000000
E	1640	R0001638	EQ	.000000	.000000	.000000	.000000
E	1641	R0001639	EQ	.000000	.000000	.000000	.000000
E	1642	R0001640	EQ	.000000	.000000	.000000	.000000
E	1643	R0001641	EQ	.000000	.000000	.000000	.000000
E	1644	R0001642	EQ	.000000	.000000	.000000	.000000
E	1645	R0001643	EQ	.000000	.000000	.000000	.000000
E	1646	R0001644	EQ	.000000	.000000	.000000	.000000
E	1647	R0001645	EQ	.000000	.000000	.000000	.000000
E	1648	R0001646	EQ	.000000	.000000	.000000	.000000
E	1649	R0001647	EQ	.000000	.000000	.000000	.000000
E	1650	R0001648	EQ	.000000	.000000	.000000	.000000
E	1651	R0001649	EQ	.000000	.000000	.000000	.000000
E	1652	R0001650	EQ	.000000	.000000	.000000	.000000
E	1653	R0001651	EQ	.000000	.000000	.000000	.000000
E	1654	R0001652	EQ	.000000	.000000	.000000	.000000
E	1655	R0001653	EQ	.000000	.000000	.000000	.000000
E	1656	R0001654	EQ	.000000	.000000	.000000	.000000
E	1657	R0001655	EQ	.000000	.000000	.000000	.000000
E	1658	R0001656	EQ	.000000	.000000	.000000	.000000
E	1659	R0001657	EQ	.000000	.000000	.000000	.000000
	Number	Row	At	Value	Slack Value	Dual Value	RHS
E	1660	R0001658	EQ	.000000	.000000	.000000	.000000
E	1661	R0001659	EQ	.000000	.000000	.000000	.000000
E	1662	R0001660	EQ	.000000	.000000	.000000	.000000
E	1663	R0001661	EQ	.000000	.000000	.000000	.000000
E	1664	R0001662	EQ	.000000	.000000	.000000	.000000
E	1665	R0001663	EQ	.000000	.000000	.000000	.000000
E	1666	R0001664	EQ	.000000	.000000	.000000	.000000
E	1667	R0001665	EQ	.000000	.000000	.000000	.000000
E	1668	R0001666	EQ	.000000	.000000	.000000	.000000
E	1669	R0001667	EQ	.000000	.000000	.000000	.000000
E	1670	R0001668	EQ	.000000	.000000	.000000	.000000
E	1671	R0001669	EQ	.000000	.000000	.000000	.000000
E	1672	R0001670	EQ	.000000	.000000	.000000	.000000
E	1673	R0001671	EQ	.000000	.000000	.000000	.000000
E	1674	R0001672	EQ	.000000	.000000	.000000	.000000
E	1675	R0001673	EQ	.000000	.000000	.000000	.000000
E	1676	R0001674	EQ	.000000	.000000	.000000	.000000

E	1677	R0001675	EQ	.000000	.000000	.000000	.000000
E	1678	R0001676	EQ	.000000	.000000	.000000	.000000
E	1679	R0001677	EQ	.000000	.000000	.000000	.000000
E	1680	R0001678	EQ	.000000	.000000	.000000	.000000
	Number	Row	At	Value	Slack Value	Dual Value	RHS
E	1681	R0001679	EQ	.000000	.000000	.000000	.000000
E	1682	R0001680	EQ	.000000	.000000	.000000	.000000
E	1683	R0001681	EQ	.000000	.000000	.000000	.000000
E	1684	R0001682	EQ	.000000	.000000	.000000	.000000
E	1685	R0001683	EQ	.000000	.000000	.000000	.000000
E	1686	R0001684	EQ	.000000	.000000	.000000	.000000
E	1687	R0001685	EQ	.000000	.000000	.000000	.000000
E	1688	R0001686	EQ	.000000	.000000	.000000	.000000
E	1689	R0001687	EQ	.000000	.000000	.000000	.000000
E	1690	R0001688	EQ	.000000	.000000	.000000	.000000
E	1691	R0001689	EQ	.000000	.000000	.000000	.000000
E	1692	R0001690	EQ	.000000	.000000	.000000	.000000
E	1693	R0001691	EQ	.000000	.000000	.000000	.000000
E	1694	R0001692	EQ	.000000	.000000	.000000	.000000
E	1695	R0001693	EQ	1.000000	.000000	1.000000	1.000000

Columns Section

	Number	Column	At	Value	Input Cost	Reduced Cost
C	1696	C0000000	LL	.000000	.000000	.000000
C	1697	C0000001	LL	.000000	.000000	.000000
C	1698	C0000002	LL	.000000	.000000	.000000
C	1699	C0000003	LL	.000000	.000000	.000000
C	1700	C0000004	BS	.009270	.000000	.000000
C	1701	C0000005	BS	.000000	.000000	.000000
C	1702	C0000006	SB	.000000	.000000	.000000
C	1703	C0000007	BS	-.053860	.000000	.000000
C	1704	C0000008	BS	.000000	.000000	.000000
C	1705	C0000009	BS	.000000	.000000	.000000
C	1706	C0000010	BS	9.17932E-04	.000000	.000000
C	1707	C0000011	LL	.000000	.000000	.000000
C	1708	C0000012	BS	.000000	.000000	.000000
C	1709	C0000013	LL	.000000	.000000	.000000
C	1710	C0000014	BS	9.17932E-04	.000000	.000000
C	1711	C0000015	LL	.000000	.000000	.000000
C	1712	C0000016	BS	.000000	.000000	.000000
C	1713	C0000017	LL	.000000	.000000	.000000
C	1714	C0000018	BS	.000000	.000000	.000000
C	1715	C0000019	BS	.000000	.000000	.000000
C	1716	C0000020	BS	.000000	.000000	.000000
	Number	Column	At	Value	Input Cost	Reduced Cost
C	1717	C0000021	BS	.000000	.000000	.000000
C	1718	C0000022	SB	.000000	.000000	.000000
C	1719	C0000023	SB	.000000	.000000	.000000
C	1720	C0000024	BS	.000000	.000000	.000000
C	1721	C0000025	BS	.009270	.000000	.000000
C	1722	C0000026	LL	.000000	.000000	.000000
C	1723	C0000027	BS	.009270	.000000	.000000
C	1724	C0000028	BS	.000000	.000000	.000000
C	1725	C0000029	BS	.000000	.000000	.000000
C	1726	C0000030	BS	.000000	.000000	.000000
C	1727	C0000031	BS	.000000	.000000	.000000
C	1728	C0000032	LL	.000000	.000000	.000000
C	1729	C0000033	SB	.000000	.000000	.000000
C	1730	C0000034	BS	.053860	.000000	.000000
C	1731	C0000035	BS	.000000	.000000	.000000
C	1732	C0000036	SB	.000000	.000000	.000000
C	1733	C0000037	SB	.000000	.000000	.000000
C	1734	C0000038	BS	.150246	.000000	.000000
C	1735	C0000039	SB	.000000	.000000	.000000
C	1736	C0000040	BS	.000000	.000000	.000000
C	1737	C0000041	BS	.000000	.000000	.000000
	Number	Column	At	Value	Input Cost	Reduced Cost
C	1738	C0000042	BS	.000000	.000000	.000000
C	1739	C0000043	BS	.000000	.000000	.000000
C	1740	C0000044	SB	.000000	.000000	.000000
C	1741	C0000045	BS	.000000	.000000	.000000

C	1742	C0000046	BS	.000000	.000000	.000000
C	1743	C0000047	SB	.000000	.000000	.000000
C	1744	C0000048	SB	.000000	.000000	.000000
C	1745	C0000049	BS	.257280	.000000	.000000
C	1746	C0000050	SB	.000000	.000000	.000000
C	1747	C0000051	SB	.000000	.000000	.000000
C	1748	C0000052	BS	.357340	.000000	.000000
C	1749	C0000053	SB	.000000	.000000	.000000
C	1750	C0000054	SB	.000000	.000000	.000000
C	1751	C0000055	BS	.075041	.000000	.000000
C	1752	C0000056	SB	.000000	.000000	.000000
C	1753	C0000057	SB	.000000	.000000	.000000
C	1754	C0000058	SB	.000000	.000000	.000000
C	1755	C0000059	SB	.000000	.000000	.000000
C	1756	C0000060	BS	.000000	.000000	.000000
C	1757	C0000061	BS	.044708	.000000	.000000
C	1758	C0000062	SB	.000000	.000000	.000000
	Number	Column	At	Value	Input Cost	Reduced Cost
C	1759	C0000063	SB	.000000	.000000	.000000
C	1760	C0000064	BS	.000000	.000000	.000000
C	1761	C0000065	LL	.000000	.000000	.000000
C	1762	C0000066	LL	.000000	.000000	.000000
C	1763	C0000067	LL	.000000	.000000	.000000
C	1764	C0000068	LL	.000000	.000000	.000000
C	1765	C0000069	LL	.000000	.000000	.000000
C	1766	C0000070	BS	.000000	.000000	.000000
C	1767	C0000071	LL	.000000	.000000	.000000
C	1768	C0000072	SB	.000000	.000000	.000000
C	1769	C0000073	LL	.000000	.000000	.000000
C	1770	C0000074	LL	.000000	.000000	.000000
C	1771	C0000075	BS	.000000	.000000	.000000
C	1772	C0000076	BS	.000000	.000000	.000000
C	1773	C0000077	BS	.000000	.000000	.000000
C	1774	C0000078	BS	.000000	.000000	.000000
C	1775	C0000079	BS	.000000	.000000	.000000
C	1776	C0000080	BS	.000000	.000000	.000000
C	1777	C0000081	LL	.000000	.000000	.000000
C	1778	C0000082	LL	.000000	.000000	.000000
C	1779	C0000083	LL	.000000	.000000	.000000
	Number	Column	At	Value	Input Cost	Reduced Cost
C	1780	C0000084	BS	.000000	.000000	.000000
C	1781	C0000085	LL	.000000	.000000	.000000
C	1782	C0000086	LL	.000000	.000000	.000000
C	1783	C0000087	BS	8.12000E-04	.000000	.000000
C	1784	C0000088	BS	.000000	.000000	.000000
C	1785	C0000089	LL	.000000	.000000	.000000
C	1786	C0000090	BS	.000000	.000000	.000000
C	1787	C0000091	BS	.000000	.000000	.000000
C	1788	C0000092	LL	.000000	.000000	.000000
C	1789	C0000093	BS	.000000	.000000	.000000
C	1790	C0000094	BS	.000000	.000000	.000000
C	1791	C0000095	BS	.000000	.000000	.000000
C	1792	C0000096	BS	.000000	.000000	.000000
C	1793	C0000097	LL	.000000	.000000	.000000
C	1794	C0000098	BS	.000000	.000000	.000000
C	1795	C0000099	BS	.000000	.000000	.000000
C	1796	C0000100	LL	.000000	.000000	.000000
C	1797	C0000101	BS	.000000	.000000	.000000
C	1798	C0000102	BS	.000000	.000000	.000000
C	1799	C0000103	LL	.000000	.000000	.000000
C	1800	C0000104	BS	.000000	.000000	.000000
	Number	Column	At	Value	Input Cost	Reduced Cost
C	1801	C0000105	LL	.000000	.000000	.000000
C	1802	C0000106	LL	.000000	.000000	.000000
C	1803	C0000107	LL	.000000	.000000	.000000
C	1804	C0000108	LL	.000000	.000000	.000000
C	1805	C0000109	LL	.000000	.000000	.000000
C	1806	C0000110	LL	.000000	.000000	.000000
C	1807	C0000111	LL	.000000	.000000	.000000
C	1808	C0000112	LL	.000000	.000000	.000000
C	1809	C0000113	LL	.000000	.000000	.000000

C	1810	C0000114	LL	.000000	.000000	.000000
C	1811	C0000115	BS	6.60000E-05	.000000	.000000
C	1812	C0000116	BS	3.51000E-04	.000000	.000000
C	1813	C0000117	BS	.006415	.000000	.000000
C	1814	C0000118	BS	1.50000E-05	.000000	.000000
C	1815	C0000119	BS	3.55568E-04	.000000	.000000
C	1816	C0000120	BS	-2.67568E-04	.000000	.000000
C	1817	C0000121	BS	1.14000E-04	.000000	.000000
C	1818	C0000122	BS	9.60000E-05	.000000	.000000
C	1819	C0000123	BS	.000000	.000000	.000000
C	1820	C0000124	BS	1.25000E-04	.000000	.000000
C	1821	C0000125	BS	.000000	.000000	.000000
	Number	Column	At	Value	Input Cost	Reduced Cost
C	1822	C0000126	SB	.000000	.000000	.000000
C	1823	C0000127	SB	.000000	.000000	.000000
C	1824	C0000128	SB	.000000	.000000	.000000
C	1825	C0000129	SB	.000000	.000000	.000000
C	1826	C0000130	SB	.000000	.000000	.000000
C	1827	C0000131	SB	.000000	.000000	.000000
C	1828	C0000132	SB	.000000	.000000	.000000
C	1829	C0000133	SB	.000000	.000000	.000000
C	1830	C0000134	SB	.000000	.000000	.000000
C	1831	C0000135	SB	.000000	.000000	.000000
C	1832	C0000136	BS	.250140	.000000	.000000
C	1833	C0000137	SB	.000000	.000000	.000000
C	1834	C0000138	SB	.000000	.000000	.000000
C	1835	C0000139	BS	.028000	.000000	.000000
C	1836	C0000140	SB	.000000	.000000	.000000
C	1837	C0000141	SB	.000000	.000000	.000000
C	1838	C0000142	BS	.000000	.000000	.000000
C	1839	C0000143	BS	.000000	.000000	.000000
C	1840	C0000144	BS	.000000	.000000	.000000
C	1841	C0000145	BS	.000000	.000000	.000000
C	1842	C0000146	BS	.000000	.000000	.000000
	Number	Column	At	Value	Input Cost	Reduced Cost
C	1843	C0000147	BS	.000000	.000000	.000000
C	1844	C0000148	LL	.000000	.000000	.000000
C	1845	C0000149	BS	.000000	.000000	.000000
C	1846	C0000150	BS	.000000	.000000	.000000
C	1847	C0000151	BS	.000000	.000000	.000000
C	1848	C0000152	LL	.000000	.000000	.000000
C	1849	C0000153	BS	.000000	.000000	.000000
C	1850	C0000154	BS	9.17932E-04	.000000	.000000
C	1851	C0000155	BS	.135790	.000000	.000000
C	1852	C0000156	BS	.000000	.000000	.000000
C	1853	C0000157	LL	.000000	.000000	.000000
C	1854	C0000158	LL	.000000	.000000	.000000
C	1855	C0000159	LL	.000000	.000000	.000000
C	1856	C0000160	BS	.000000	.000000	.000000
C	1857	C0000161	LL	.000000	.000000	.000000
C	1858	C0000162	LL	.000000	.000000	.000000
C	1859	C0000163	BS	.000000	.000000	.000000
C	1860	C0000164	BS	.000000	.000000	.000000
C	1861	C0000165	BS	-.003587	.000000	.000000
C	1862	C0000166	LL	.000000	.000000	.000000
C	1863	C0000167	BS	.000000	.000000	.000000
	Number	Column	At	Value	Input Cost	Reduced Cost
C	1864	C0000168	BS	.000000	.000000	.000000
C	1865	C0000169	BS	.006525	.000000	.000000
C	1866	C0000170	BS	.000000	.000000	.000000
C	1867	C0000171	BS	.000000	.000000	.000000
C	1868	C0000172	BS	.000000	.000000	.000000
C	1869	C0000173	BS	.000000	.000000	.000000
C	1870	C0000174	BS	.000000	.000000	.000000
C	1871	C0000175	BS	.000000	.000000	.000000
C	1872	C0000176	BS	.000000	.000000	.000000
C	1873	C0000177	BS	.000000	.000000	.000000
C	1874	C0000178	LL	.000000	.000000	.000000
C	1875	C0000179	LL	.000000	.000000	.000000
C	1876	C0000180	BS	.004160	.000000	.000000
C	1877	C0000181	BS	.000000	.000000	.000000

C	1878	C0000182	BS	.003090	.000000	.000000
C	1879	C0000183	LL	.000000	.000000	.000000
C	1880	C0000184	BS	.000000	.000000	.000000
C	1881	C0000185	LL	.000000	.000000	.000000
C	1882	C0000186	LL	.000000	.000000	.000000
C	1883	C0000187	LL	.000000	.000000	.000000
C	1884	C0000188	LL	.000000	.000000	.000000
	Number	Column	At	Value	Input Cost	Reduced Cost
C	1885	C0000189	BS	.000000	.000000	.000000
C	1886	C0000190	LL	.000000	.000000	.000000
C	1887	C0000191	LL	.000000	.000000	.000000
C	1888	C0000192	LL	.000000	.000000	.000000
C	1889	C0000193	LL	.000000	.000000	.000000
C	1890	C0000194	LL	.000000	.000000	.000000
C	1891	C0000195	BS	.000000	.000000	.000000
C	1892	C0000196	LL	.000000	.000000	.000000
C	1893	C0000197	LL	.000000	.000000	.000000
C	1894	C0000198	BS	.000000	.000000	.000000
C	1895	C0000199	LL	.000000	.000000	.000000
C	1896	C0000200	BS	.000000	.000000	.000000
C	1897	C0000201	BS	6.60000E-05	.000000	.000000
C	1898	C0000202	LL	.000000	.000000	.000000
C	1899	C0000203	LL	.000000	.000000	.000000
C	1900	C0000204	BS	.000000	.000000	.000000
C	1901	C0000205	BS	.000000	.000000	.000000
C	1902	C0000206	BS	.000000	.000000	.000000
C	1903	C0000207	LL	.000000	.000000	.000000
C	1904	C0000208	LL	.000000	.000000	.000000
C	1905	C0000209	LL	.000000	.000000	.000000
	Number	Column	At	Value	Input Cost	Reduced Cost
C	1906	C0000210	BS	.000000	.000000	.000000
C	1907	C0000211	LL	.000000	.000000	.000000
C	1908	C0000212	BS	.000000	.000000	.000000
C	1909	C0000213	LL	.000000	.000000	.000000
C	1910	C0000214	BS	.000000	.000000	.000000
C	1911	C0000215	BS	.000000	.000000	.000000
C	1912	C0000216	BS	.000000	.000000	.000000
C	1913	C0000217	LL	.000000	.000000	.000000
C	1914	C0000218	LL	.000000	.000000	.000000
C	1915	C0000219	LL	.000000	.000000	.000000
C	1916	C0000220	BS	-.002432	.000000	.000000
C	1917	C0000221	LL	.000000	.000000	.000000
C	1918	C0000222	LL	.000000	.000000	.000000
C	1919	C0000223	BS	.000000	.000000	.000000
C	1920	C0000224	LL	.000000	.000000	.000000
C	1921	C0000225	BS	.000000	.000000	.000000
C	1922	C0000226	BS	.000000	.000000	.000000
C	1923	C0000227	SB	.000000	.000000	.000000
C	1924	C0000228	LL	.000000	.000000	.000000
C	1925	C0000229	LL	.000000	.000000	.000000
C	1926	C0000230	BS	7.81000E-04	.000000	.000000
	Number	Column	At	Value	Input Cost	Reduced Cost
C	1927	C0000231	BS	.000000	.000000	.000000
C	1928	C0000232	BS	.119432	.000000	.000000
C	1929	C0000233	BS	.000000	.000000	.000000
C	1930	C0000234	LL	.000000	.000000	.000000
C	1931	C0000235	BS	.000000	.000000	.000000
C	1932	C0000236	SB	.000000	.000000	.000000
C	1933	C0000237	SB	.000000	.000000	.000000
C	1934	C0000238	SB	.000000	.000000	.000000
C	1935	C0000239	BS	.344812	.000000	.000000
C	1936	C0000240	SB	.000000	.000000	.000000
C	1937	C0000241	SB	.000000	.000000	.000000
C	1938	C0000242	SB	.000000	.000000	.000000
C	1939	C0000243	LL	.000000	.000000	.000000
C	1940	C0000244	BS	.000000	.000000	.000000
C	1941	C0000245	BS	.000000	.000000	.000000
C	1942	C0000246	LL	.000000	.000000	.000000
C	1943	C0000247	BS	.820990	.000000	.000000
C	1944	C0000248	BS	7.81000E-04	.000000	.000000
C	1945	C0000249	BS	.000000	.000000	.000000

C	1946	C0000250	BS	.000000	.000000	.000000
C	1947	C0000251	LL	.000000	.000000	.000000
	Number	Column	At	Value	Input Cost	Reduced Cost
C	1948	C0000252	BS	.000000	.000000	.000000
C	1949	C0000253	LL	.000000	.000000	.000000
C	1950	C0000254	BS	.000000	.000000	.000000
C	1951	C0000255	BS	.000000	.000000	.000000
C	1952	C0000256	LL	.000000	.000000	.000000
C	1953	C0000257	BS	.000000	.000000	.000000
C	1954	C0000258	BS	.000000	.000000	.000000
C	1955	C0000259	BS	.000000	.000000	.000000
C	1956	C0000260	BS	.000000	.000000	.000000
C	1957	C0000261	BS	.000000	.000000	.000000
C	1958	C0000262	BS	.000000	.000000	.000000
C	1959	C0000263	BS	.000000	.000000	.000000
C	1960	C0000264	LL	.000000	.000000	.000000
C	1961	C0000265	LL	.000000	.000000	.000000
C	1962	C0000266	BS	.000000	.000000	.000000
C	1963	C0000267	LL	.000000	.000000	.000000
C	1964	C0000268	BS	.000000	.000000	.000000
C	1965	C0000269	BS	.000000	.000000	.000000
C	1966	C0000270	BS	.000000	.000000	.000000
C	1967	C0000271	LL	.000000	.000000	.000000
C	1968	C0000272	LL	.000000	.000000	.000000
	Number	Column	At	Value	Input Cost	Reduced Cost
C	1969	C0000273	LL	.000000	.000000	.000000
C	1970	C0000274	LL	.000000	.000000	.000000
C	1971	C0000275	LL	.000000	.000000	.000000
C	1972	C0000276	BS	-.053860	.000000	.000000
C	1973	C0000277	SB	.000000	.000000	.000000
C	1974	C0000278	BS	.000000	.000000	.000000
C	1975	C0000279	BS	.000000	.000000	.000000
C	1976	C0000280	BS	.000000	.000000	.000000
C	1977	C0000281	BS	3.23568E-04	.000000	.000000
C	1978	C0000282	LL	.000000	.000000	.000000
C	1979	C0000283	LL	.000000	.000000	.000000
C	1980	C0000284	LL	.000000	.000000	.000000
C	1981	C0000285	LL	.000000	.000000	.000000
C	1982	C0000286	LL	.000000	.000000	.000000
C	1983	C0000287	BS	.000000	.000000	.000000
C	1984	C0000288	BS	.000000	.000000	.000000
C	1985	C0000289	BS	3.23568E-04	.000000	.000000
C	1986	C0000290	BS	.000000	.000000	.000000
C	1987	C0000291	BS	.000000	.000000	.000000
C	1988	C0000292	BS	.000000	.000000	.000000
C	1989	C0000293	BS	.000000	.000000	.000000
	Number	Column	At	Value	Input Cost	Reduced Cost
C	1990	C0000294	BS	.000000	.000000	.000000
C	1991	C0000295	LL	.000000	.000000	.000000
C	1992	C0000296	BS	.000000	.000000	.000000
C	1993	C0000297	LL	.000000	.000000	.000000
C	1994	C0000298	LL	.000000	.000000	.000000
C	1995	C0000299	LL	.000000	.000000	.000000
C	1996	C0000300	BS	.000000	.000000	.000000
C	1997	C0000301	BS	.000000	.000000	.000000
C	1998	C0000302	BS	.000000	.000000	.000000
C	1999	C0000303	BS	.000000	.000000	.000000
C	2000	C0000304	SB	.000000	.000000	.000000
C	2001	C0000305	SB	.000000	.000000	.000000
C	2002	C0000306	SB	.000000	.000000	.000000
C	2003	C0000307	BS	.000000	.000000	.000000
C	2004	C0000308	BS	.000000	.000000	.000000
C	2005	C0000309	BS	.000000	.000000	.000000
C	2006	C0000310	BS	.000000	.000000	.000000
C	2007	C0000311	BS	.000000	.000000	.000000
C	2008	C0000312	BS	.000000	.000000	.000000
C	2009	C0000313	BS	.000000	.000000	.000000
C	2010	C0000314	BS	.000000	.000000	.000000
	Number	Column	At	Value	Input Cost	Reduced Cost
C	2011	C0000315	LL	.000000	.000000	.000000
C	2012	C0000316	LL	.000000	.000000	.000000

C	2013	C0000317	LL	.000000	.000000	.000000
C	2014	C0000318	LL	.000000	.000000	.000000
C	2015	C0000319	LL	.000000	.000000	.000000
C	2016	C0000320	BS	1.34339E-04	.000000	.000000
C	2017	C0000321	BS	4.43871E-04	.000000	.000000
C	2018	C0000322	LL	.000000	.000000	.000000
C	2019	C0000323	LL	.000000	.000000	.000000
C	2020	C0000324	LL	.000000	.000000	.000000
C	2021	C0000325	LL	.000000	.000000	.000000
C	2022	C0000326	BS	.009803	.000000	.000000
C	2023	C0000327	BS	.002624	.000000	.000000
C	2024	C0000328	LL	.000000	.000000	.000000
C	2025	C0000329	LL	.000000	.000000	.000000
C	2026	C0000330	BS	.000000	.000000	.000000
C	2027	C0000331	BS	9.17932E-04	.000000	.000000
C	2028	C0000332	BS	.000000	.000000	.000000
C	2029	C0000333	BS	.000000	.000000	.000000
C	2030	C0000334	BS	.000000	.000000	.000000
C	2031	C0000335	BS	.000000	.000000	.000000
	Number	Column	At	Value	Input Cost	Reduced Cost
C	2032	C0000336	LL	.000000	.000000	.000000
C	2033	C0000337	BS	.000000	.000000	.000000
C	2034	C0000338	BS	.000000	.000000	.000000
C	2035	C0000339	BS	.000000	.000000	.000000
C	2036	C0000340	BS	.000000	.000000	.000000
C	2037	C0000341	LL	.000000	.000000	.000000
C	2038	C0000342	LL	.000000	.000000	.000000
C	2039	C0000343	BS	.000000	.000000	.000000
C	2040	C0000344	BS	-3.09742E-04	.000000	.000000
C	2041	C0000345	LL	.000000	.000000	.000000
C	2042	C0000346	BS	3.09742E-04	.000000	.000000
C	2043	C0000347	BS	3.09742E-04	.000000	.000000
C	2044	C0000348	BS	3.09742E-04	.000000	.000000
C	2045	C0000349	BS	.000000	.000000	.000000
C	2046	C0000350	BS	.000000	.000000	.000000
C	2047	C0000351	BS	.000000	.000000	.000000
C	2048	C0000352	BS	-6.08190E-04	.000000	.000000
C	2049	C0000353	LL	.000000	.000000	.000000
C	2050	C0000354	BS	6.08190E-04	.000000	.000000
C	2051	C0000355	BS	6.08190E-04	.000000	.000000
C	2052	C0000356	BS	6.08190E-04	.000000	.000000
	Number	Column	At	Value	Input Cost	Reduced Cost
C	2053	C0000357	BS	.000000	.000000	.000000
C	2054	C0000358	BS	.000000	.000000	.000000
C	2055	C0000359	BS	.000000	.000000	.000000
C	2056	C0000360	BS	.000000	.000000	.000000
C	2057	C0000361	BS	.000000	.000000	.000000
C	2058	C0000362	BS	.000000	.000000	.000000
C	2059	C0000363	BS	.000000	.000000	.000000
C	2060	C0000364	BS	.000000	.000000	.000000
C	2061	C0000365	BS	.000000	.000000	.000000
C	2062	C0000366	BS	.000000	.000000	.000000
C	2063	C0000367	BS	.000000	.000000	.000000
C	2064	C0000368	BS	.000000	.000000	.000000
C	2065	C0000369	BS	3.09742E-04	.000000	.000000
C	2066	C0000370	BS	3.09742E-04	.000000	.000000
C	2067	C0000371	BS	.000000	.000000	.000000
C	2068	C0000372	BS	.000000	.000000	.000000
C	2069	C0000373	BS	6.08190E-04	.000000	.000000
C	2070	C0000374	BS	6.08190E-04	.000000	.000000
C	2071	C0000375	BS	.003524	.000000	.000000
C	2072	C0000376	LL	.000000	.000000	.000000
C	2073	C0000377	LL	.000000	.000000	.000000
	Number	Column	At	Value	Input Cost	Reduced Cost
C	2074	C0000378	LL	.000000	.000000	.000000
C	2075	C0000379	BS	9.24819E-05	.000000	.000000
C	2076	C0000380	LL	.000000	.000000	.000000
C	2077	C0000381	BS	4.17000E-04	.000000	.000000
C	2078	C0000382	BS	62.545067	.000000	.000000
C	2079	C0000383	SB	.000000	.000000	.000000
C	2080	C0000384	LL	.000000	.000000	.000000

	Number	Column	At	Value	Input Cost	Reduced Cost
C	2081	C0000385	BS	.000000	.000000	.000000
C	2082	C0000386	BS	.000000	.000000	.000000
C	2083	C0000387	LL	.000000	.000000	.000000
C	2084	C0000388	LL	.000000	.000000	.000000
C	2085	C0000389	SB	.000000	.000000	.000000
C	2086	C0000390	LL	.000000	.000000	.000000
C	2087	C0000391	LL	.000000	.000000	.000000
C	2088	C0000392	BS	.053849	.000000	.000000
C	2089	C0000393	LL	.000000	.000000	.000000
C	2090	C0000394	LL	.000000	.000000	.000000
C	2091	C0000395	SB	.000000	.000000	.000000
C	2092	C0000396	LL	.000000	.000000	.000000
C	2093	C0000397	BS	.000000	.000000	.000000
C	2094	C0000398	LL	.000000	.000000	.000000
	Number	Column	At	Value	Input Cost	Reduced Cost
C	2095	C0000399	LL	.000000	.000000	.000000
C	2096	C0000400	BS	1.135800	.000000	.000000
C	2097	C0000401	LL	.000000	.000000	.000000
C	2098	C0000402	LL	.000000	.000000	.000000
C	2099	C0000403	BS	.000000	.000000	.000000
C	2100	C0000404	BS	.000000	.000000	.000000
C	2101	C0000405	LL	.000000	.000000	.000000
C	2102	C0000406	LL	.000000	.000000	.000000
C	2103	C0000407	BS	.000000	.000000	.000000
C	2104	C0000408	BS	.000000	.000000	.000000
C	2105	C0000409	LL	.000000	.000000	.000000
C	2106	C0000410	BS	.000000	.000000	.000000
C	2107	C0000411	BS	.000000	.000000	.000000
C	2108	C0000412	BS	.000000	.000000	.000000
C	2109	C0000413	BS	.000000	.000000	.000000
C	2110	C0000414	LL	.000000	.000000	.000000
C	2111	C0000415	BS	.000000	.000000	.000000
C	2112	C0000416	BS	.000000	.000000	.000000
C	2113	C0000417	BS	.000000	.000000	.000000
C	2114	C0000418	LL	.000000	.000000	.000000
C	2115	C0000419	LL	.000000	.000000	.000000
	Number	Column	At	Value	Input Cost	Reduced Cost
C	2116	C0000420	LL	.000000	.000000	.000000
C	2117	C0000421	LL	.000000	.000000	.000000
C	2118	C0000422	LL	.000000	.000000	.000000
C	2119	C0000423	LL	.000000	.000000	.000000
C	2120	C0000424	LL	.000000	.000000	.000000
C	2121	C0000425	LL	.000000	.000000	.000000
C	2122	C0000426	LL	.000000	.000000	.000000
C	2123	C0000427	LL	.000000	.000000	.000000
C	2124	C0000428	LL	.000000	.000000	.000000
C	2125	C0000429	BS	.007306	.000000	.000000
C	2126	C0000430	BS	.000000	.000000	.000000
C	2127	C0000431	LL	.000000	.000000	.000000
C	2128	C0000432	LL	.000000	.000000	.000000
C	2129	C0000433	BS	.000000	.000000	.000000
C	2130	C0000434	LL	.000000	.000000	.000000
C	2131	C0000435	LL	.000000	.000000	.000000
C	2132	C0000436	LL	.000000	.000000	.000000
C	2133	C0000437	BS	-.119432	.000000	.000000
C	2134	C0000438	SB	.000000	.000000	.000000
C	2135	C0000439	LL	.000000	.000000	.000000
C	2136	C0000440	LL	.000000	.000000	.000000
	Number	Column	At	Value	Input Cost	Reduced Cost
C	2137	C0000441	LL	.000000	.000000	.000000
C	2138	C0000442	BS	.000000	.000000	.000000
C	2139	C0000443	LL	.000000	.000000	.000000
C	2140	C0000444	BS	.000000	.000000	.000000
C	2141	C0000445	LL	.000000	.000000	.000000
C	2142	C0000446	LL	.000000	.000000	.000000
C	2143	C0000447	BS	1.677691	.000000	.000000
C	2144	C0000448	BS	-1.677691	.000000	.000000
C	2145	C0000449	SB	.000000	.000000	.000000
C	2146	C0000450	BS	.171520	.000000	.000000
C	2147	C0000451	SB	.000000	.000000	.000000
C	2148	C0000452	SB	.000000	.000000	.000000

	Number	Column	At	Value	Input Cost	Reduced Cost
C	2149	C0000453	SB	.000000	.000000	.000000
C	2150	C0000454	BS	.128640	.000000	.000000
C	2151	C0000455	SB	.000000	.000000	.000000
C	2152	C0000456	SB	.000000	.000000	.000000
C	2153	C0000457	SB	.000000	.000000	.000000
C	2154	C0000458	BS	.267557	.000000	.000000
C	2155	C0000459	BS	.196530	.000000	.000000
C	2156	C0000460	SB	.000000	.000000	.000000
C	2157	C0000461	SB	.000000	.000000	.000000
	Number	Column	At	Value	Input Cost	Reduced Cost
C	2158	C0000462	SB	.000000	.000000	.000000
C	2159	C0000463	SB	.000000	.000000	.000000
C	2160	C0000464	BS	.171520	.000000	.000000
C	2161	C0000465	SB	.000000	.000000	.000000
C	2162	C0000466	BS	.042880	.000000	.000000
C	2163	C0000467	SB	.000000	.000000	.000000
C	2164	C0000468	SB	.000000	.000000	.000000
C	2165	C0000469	SB	.000000	.000000	.000000
C	2166	C0000470	LL	.000000	.000000	.000000
C	2167	C0000471	LL	.000000	.000000	.000000
C	2168	C0000472	BS	.000000	.000000	.000000
C	2169	C0000473	LL	.000000	.000000	.000000
C	2170	C0000474	LL	.000000	.000000	.000000
C	2171	C0000475	LL	.000000	.000000	.000000
C	2172	C0000476	LL	.000000	.000000	.000000
C	2173	C0000477	LL	.000000	.000000	.000000
C	2174	C0000478	BS	-2.06000E-04	.000000	.000000
C	2175	C0000479	BS	.000000	.000000	.000000
C	2176	C0000480	LL	.000000	.000000	.000000
C	2177	C0000481	LL	.000000	.000000	.000000
C	2178	C0000482	LL	.000000	.000000	.000000
	Number	Column	At	Value	Input Cost	Reduced Cost
C	2179	C0000483	BS	.000000	.000000	.000000
C	2180	C0000484	LL	.000000	.000000	.000000
C	2181	C0000485	BS	.000000	.000000	.000000
C	2182	C0000486	BS	.000000	.000000	.000000
C	2183	C0000487	LL	.000000	.000000	.000000
C	2184	C0000488	BS	.000000	.000000	.000000
C	2185	C0000489	LL	.000000	.000000	.000000
C	2186	C0000490	LL	.000000	.000000	.000000
C	2187	C0000491	LL	.000000	.000000	.000000
C	2188	C0000492	BS	.000000	.000000	.000000
C	2189	C0000493	LL	.000000	.000000	.000000
C	2190	C0000494	LL	.000000	.000000	.000000
C	2191	C0000495	SB	.000000	.000000	.000000
C	2192	C0000496	SB	.000000	.000000	.000000
C	2193	C0000497	BS	.437432	.000000	.000000
C	2194	C0000498	SB	.000000	.000000	.000000
C	2195	C0000499	BS	.050027	.000000	.000000
C	2196	C0000500	SB	.000000	.000000	.000000
C	2197	C0000501	SB	.000000	.000000	.000000
C	2198	C0000502	LL	.000000	.000000	.000000
C	2199	C0000503	LL	.000000	.000000	.000000
	Number	Column	At	Value	Input Cost	Reduced Cost
C	2200	C0000504	LL	.000000	.000000	.000000
C	2201	C0000505	BS	.000000	.000000	.000000
C	2202	C0000506	BS	-.053860	.000000	.000000
C	2203	C0000507	SB	.000000	.000000	.000000
C	2204	C0000508	SB	.000000	.000000	.000000
C	2205	C0000509	BS	.007306	.000000	.000000
C	2206	C0000510	LL	.000000	.000000	.000000
C	2207	C0000511	BS	.000000	.000000	.000000
C	2208	C0000512	SB	.000000	.000000	.000000
C	2209	C0000513	BS	.000000	.000000	.000000
C	2210	C0000514	LL	.000000	.000000	.000000
C	2211	C0000515	LL	.000000	.000000	.000000
C	2212	C0000516	BS	.000000	.000000	.000000
C	2213	C0000517	BS	.000000	.000000	.000000
C	2214	C0000518	BS	.518520	.000000	.000000
C	2215	C0000519	LL	.000000	.000000	.000000
C	2216	C0000520	LL	.000000	.000000	.000000

C	2217	C0000521	SB	.000000	.000000	.000000
C	2218	C0000522	BS	-.002432	.000000	.000000
C	2219	C0000523	BS	.002849	.000000	.000000
C	2220	C0000524	BS	.007306	.000000	.000000
	Number	Column	At	Value	Input Cost	Reduced Cost
C	2221	C0000525	LL	.000000	.000000	.000000
C	2222	C0000526	BS	.000000	.000000	.000000
C	2223	C0000527	LL	.000000	.000000	.000000
C	2224	C0000528	BS	.000000	.000000	.000000
C	2225	C0000529	BS	.000000	.000000	.000000
C	2226	C0000530	BS	.000000	.000000	.000000
C	2227	C0000531	BS	.000000	.000000	.000000
C	2228	C0000532	BS	.000000	.000000	.000000
C	2229	C0000533	LL	.000000	.000000	.000000
C	2230	C0000534	BS	.000000	.000000	.000000
C	2231	C0000535	SB	.000000	.000000	.000000
C	2232	C0000536	LL	.000000	.000000	.000000
C	2233	C0000537	BS	.000000	.000000	.000000
C	2234	C0000538	BS	.000000	.000000	.000000
C	2235	C0000539	BS	.000000	.000000	.000000
C	2236	C0000540	BS	.000000	.000000	.000000
C	2237	C0000541	BS	.000000	.000000	.000000
C	2238	C0000542	BS	.000000	.000000	.000000
C	2239	C0000543	BS	.000000	.000000	.000000
C	2240	C0000544	BS	.000000	.000000	.000000
C	2241	C0000545	BS	.000000	.000000	.000000
	Number	Column	At	Value	Input Cost	Reduced Cost
C	2242	C0000546	SB	.000000	.000000	.000000
C	2243	C0000547	BS	.000000	.000000	.000000
C	2244	C0000548	BS	.000000	.000000	.000000
C	2245	C0000549	LL	.000000	.000000	.000000
C	2246	C0000550	BS	.000000	.000000	.000000
C	2247	C0000551	BS	.000000	.000000	.000000
C	2248	C0000552	LL	.000000	.000000	.000000
C	2249	C0000553	LL	.000000	.000000	.000000
C	2250	C0000554	LL	.000000	.000000	.000000
C	2251	C0000555	LL	.000000	.000000	.000000
C	2252	C0000556	LL	.000000	.000000	.000000
C	2253	C0000557	LL	.000000	.000000	.000000
C	2254	C0000558	LL	.000000	.000000	.000000
C	2255	C0000559	LL	.000000	.000000	.000000
C	2256	C0000560	LL	.000000	.000000	.000000
C	2257	C0000561	LL	.000000	.000000	.000000
C	2258	C0000562	LL	.000000	.000000	.000000
C	2259	C0000563	LL	.000000	.000000	.000000
C	2260	C0000564	LL	.000000	.000000	.000000
C	2261	C0000565	BS	.000000	.000000	.000000
C	2262	C0000566	LL	.000000	.000000	.000000
	Number	Column	At	Value	Input Cost	Reduced Cost
C	2263	C0000567	LL	.000000	.000000	.000000
C	2264	C0000568	LL	.000000	.000000	.000000
C	2265	C0000569	LL	.000000	.000000	.000000
C	2266	C0000570	LL	.000000	.000000	.000000
C	2267	C0000571	BS	.000000	.000000	.000000
C	2268	C0000572	LL	.000000	.000000	.000000
C	2269	C0000573	LL	.000000	.000000	.000000
C	2270	C0000574	LL	.000000	.000000	.000000
C	2271	C0000575	BS	.000000	.000000	.000000
C	2272	C0000576	LL	.000000	.000000	.000000
C	2273	C0000577	LL	.000000	.000000	.000000
C	2274	C0000578	LL	.000000	.000000	.000000
C	2275	C0000579	LL	.000000	.000000	.000000
C	2276	C0000580	LL	.000000	.000000	.000000
C	2277	C0000581	LL	.000000	.000000	.000000
C	2278	C0000582	LL	.000000	.000000	.000000
C	2279	C0000583	BS	.000000	.000000	.000000
C	2280	C0000584	LL	.000000	.000000	.000000
C	2281	C0000585	LL	.000000	.000000	.000000
C	2282	C0000586	LL	.000000	.000000	.000000
C	2283	C0000587	LL	.000000	.000000	.000000
	Number	Column	At	Value	Input Cost	Reduced Cost

C	2284	C0000588	LL	.000000	.000000	.000000
C	2285	C0000589	LL	.000000	.000000	.000000
C	2286	C0000590	LL	.000000	.000000	.000000
C	2287	C0000591	LL	.000000	.000000	.000000
C	2288	C0000592	LL	.000000	.000000	.000000
C	2289	C0000593	LL	.000000	.000000	.000000
C	2290	C0000594	LL	.000000	.000000	.000000
C	2291	C0000595	LL	.000000	.000000	.000000
C	2292	C0000596	LL	.000000	.000000	.000000
C	2293	C0000597	LL	.000000	.000000	.000000
C	2294	C0000598	LL	.000000	.000000	.000000
C	2295	C0000599	LL	.000000	.000000	.000000
C	2296	C0000600	LL	.000000	.000000	.000000
C	2297	C0000601	LL	.000000	.000000	.000000
C	2298	C0000602	LL	.000000	.000000	.000000
C	2299	C0000603	LL	.000000	.000000	.000000
C	2300	C0000604	BS	.000000	.000000	.000000
C	2301	C0000605	LL	.000000	.000000	.000000
C	2302	C0000606	LL	.000000	.000000	.000000
C	2303	C0000607	LL	.000000	.000000	.000000
C	2304	C0000608	LL	.000000	.000000	.000000
	Number	Column	At	Value	Input Cost	Reduced Cost
C	2305	C0000609	LL	.000000	.000000	.000000
C	2306	C0000610	LL	.000000	.000000	.000000
C	2307	C0000611	LL	.000000	.000000	.000000
C	2308	C0000612	LL	.000000	.000000	.000000
C	2309	C0000613	LL	.000000	.000000	.000000
C	2310	C0000614	LL	.000000	.000000	.000000
C	2311	C0000615	LL	.000000	.000000	.000000
C	2312	C0000616	LL	.000000	.000000	.000000
C	2313	C0000617	LL	.000000	.000000	.000000
C	2314	C0000618	LL	.000000	.000000	.000000
C	2315	C0000619	LL	.000000	.000000	.000000
C	2316	C0000620	LL	.000000	.000000	.000000
C	2317	C0000621	LL	.000000	.000000	.000000
C	2318	C0000622	LL	.000000	.000000	.000000
C	2319	C0000623	LL	.000000	.000000	.000000
C	2320	C0000624	LL	.000000	.000000	.000000
C	2321	C0000625	SB	.000000	.000000	.000000
C	2322	C0000626	LL	.000000	.000000	.000000
C	2323	C0000627	LL	.000000	.000000	.000000
C	2324	C0000628	LL	.000000	.000000	.000000
C	2325	C0000629	BS	.000000	.000000	.000000
	Number	Column	At	Value	Input Cost	Reduced Cost
C	2326	C0000630	LL	.000000	.000000	.000000
C	2327	C0000631	BS	.000000	.000000	.000000
C	2328	C0000632	LL	.000000	.000000	.000000
C	2329	C0000633	LL	.000000	.000000	.000000
C	2330	C0000634	LL	.000000	.000000	.000000
C	2331	C0000635	BS	.000000	.000000	.000000
C	2332	C0000636	LL	.000000	.000000	.000000
C	2333	C0000637	LL	.000000	.000000	.000000
C	2334	C0000638	LL	.000000	.000000	.000000
C	2335	C0000639	BS	.000000	.000000	.000000
C	2336	C0000640	BS	.000000	.000000	.000000
C	2337	C0000641	BS	.000000	.000000	.000000
C	2338	C0000642	BS	.000000	.000000	.000000
C	2339	C0000643	BS	.000000	.000000	.000000
C	2340	C0000644	BS	.000000	.000000	.000000
C	2341	C0000645	BS	.000000	.000000	.000000
C	2342	C0000646	BS	.000000	.000000	.000000
C	2343	C0000647	BS	.000000	.000000	.000000
C	2344	C0000648	BS	.000000	.000000	.000000
C	2345	C0000649	BS	.000000	.000000	.000000
C	2346	C0000650	LL	.000000	.000000	.000000
	Number	Column	At	Value	Input Cost	Reduced Cost
C	2347	C0000651	LL	.000000	.000000	.000000
C	2348	C0000652	BS	.000000	.000000	.000000
C	2349	C0000653	LL	.000000	.000000	.000000
C	2350	C0000654	LL	.000000	.000000	.000000
C	2351	C0000655	LL	.000000	.000000	.000000

C	2352	C0000656	LL	.000000	.000000	.000000
C	2353	C0000657	LL	.000000	.000000	.000000
C	2354	C0000658	LL	.000000	.000000	.000000
C	2355	C0000659	LL	.000000	.000000	.000000
C	2356	C0000660	LL	.000000	.000000	.000000
C	2357	C0000661	LL	.000000	.000000	.000000
C	2358	C0000662	BS	.000000	.000000	.000000
C	2359	C0000663	LL	.000000	.000000	.000000
C	2360	C0000664	LL	.000000	.000000	.000000
C	2361	C0000665	LL	.000000	.000000	.000000
C	2362	C0000666	BS	.000000	.000000	.000000
C	2363	C0000667	BS	.000000	.000000	.000000
C	2364	C0000668	BS	2.652282	.000000	.000000
C	2365	C0000669	BS	6.60000E-05	.000000	.000000
C	2366	C0000670	BS	3.51000E-04	.000000	.000000
C	2367	C0000671	LL	.000000	.000000	.000000
	Number	Column	At	Value	Input Cost	Reduced Cost
C	2368	C0000672	LL	.000000	.000000	.000000
C	2369	C0000673	BS	.000000	.000000	.000000
C	2370	C0000674	LL	.000000	.000000	.000000
C	2371	C0000675	LL	.000000	.000000	.000000
C	2372	C0000676	BS	.000000	.000000	.000000
C	2373	C0000677	LL	.000000	.000000	.000000
C	2374	C0000678	BS	.002365	.000000	.000000
C	2375	C0000679	BS	.000000	.000000	.000000
C	2376	C0000680	BS	.000000	.000000	.000000
C	2377	C0000681	BS	.000000	.000000	.000000
C	2378	C0000682	BS	.000000	.000000	.000000
C	2379	C0000683	BS	.000000	.000000	.000000
C	2380	C0000684	LL	.000000	.000000	.000000
C	2381	C0000685	LL	.000000	.000000	.000000
C	2382	C0000686	LL	.000000	.000000	.000000
C	2383	C0000687	LL	.000000	.000000	.000000
C	2384	C0000688	LL	.000000	.000000	.000000
C	2385	C0000689	LL	.000000	.000000	.000000
C	2386	C0000690	LL	.000000	.000000	.000000
C	2387	C0000691	BS	6.60000E-05	.000000	.000000
C	2388	C0000692	BS	3.51000E-04	.000000	.000000
	Number	Column	At	Value	Input Cost	Reduced Cost
C	2389	C0000693	LL	.000000	.000000	.000000
C	2390	C0000694	BS	4.17000E-04	.000000	.000000
C	2391	C0000695	LL	.000000	.000000	.000000
C	2392	C0000696	LL	.000000	.000000	.000000
C	2393	C0000697	LL	.000000	.000000	.000000
C	2394	C0000698	LL	.000000	.000000	.000000
C	2395	C0000699	LL	.000000	.000000	.000000
C	2396	C0000700	LL	.000000	.000000	.000000
C	2397	C0000701	LL	.000000	.000000	.000000
C	2398	C0000702	BS	.000000	.000000	.000000
C	2399	C0000703	BS	.000000	.000000	.000000
C	2400	C0000704	BS	.000000	.000000	.000000
C	2401	C0000705	LL	.000000	.000000	.000000
C	2402	C0000706	BS	4.17000E-04	.000000	.000000
C	2403	C0000707	BS	.000000	.000000	.000000
C	2404	C0000708	BS	.000000	.000000	.000000
C	2405	C0000709	LL	.000000	.000000	.000000
C	2406	C0000710	LL	.000000	.000000	.000000
C	2407	C0000711	BS	.000000	.000000	.000000
C	2408	C0000712	BS	.000000	.000000	.000000
C	2409	C0000713	BS	.239420	.000000	.000000
	Number	Column	At	Value	Input Cost	Reduced Cost
C	2410	C0000714	BS	.000000	.000000	.000000
C	2411	C0000715	BS	.000000	.000000	.000000
C	2412	C0000716	LL	.000000	.000000	.000000
C	2413	C0000717	LL	.000000	.000000	.000000
C	2414	C0000718	BS	.000000	.000000	.000000
C	2415	C0000719	BS	.000000	.000000	.000000
C	2416	C0000720	LL	.000000	.000000	.000000
C	2417	C0000721	BS	.000000	.000000	.000000
C	2418	C0000722	BS	.000000	.000000	.000000
C	2419	C0000723	LL	.000000	.000000	.000000

C	2420	C0000724	LL	.000000	.000000	.000000
C	2421	C0000725	LL	.000000	.000000	.000000
C	2422	C0000726	SB	.000000	.000000	.000000
C	2423	C0000727	LL	.000000	.000000	.000000
C	2424	C0000728	BS	.000000	.000000	.000000
C	2425	C0000729	BS	.000000	.000000	.000000
C	2426	C0000730	SB	.000000	.000000	.000000
C	2427	C0000731	SB	.000000	.000000	.000000
C	2428	C0000732	BS	-2.637026	.000000	.000000
C	2429	C0000733	SB	.000000	.000000	.000000
C	2430	C0000734	SB	.000000	.000000	.000000
	Number	Column	At	Value	Input Cost	Reduced Cost
C	2431	C0000735	BS	.000000	.000000	.000000
C	2432	C0000736	BS	.000000	.000000	.000000
C	2433	C0000737	SB	.000000	.000000	.000000
C	2434	C0000738	BS	.000000	.000000	.000000
C	2435	C0000739	LL	.000000	.000000	.000000
C	2436	C0000740	BS	.000000	.000000	.000000
C	2437	C0000741	LL	.000000	.000000	.000000
C	2438	C0000742	BS	.000000	.000000	.000000
C	2439	C0000743	BS	.000000	.000000	.000000
C	2440	C0000744	LL	.000000	.000000	.000000
C	2441	C0000745	BS	.000000	.000000	.000000
C	2442	C0000746	BS	.063857	.000000	.000000
C	2443	C0000747	LL	.000000	.000000	.000000
C	2444	C0000748	BS	.000000	.000000	.000000
C	2445	C0000749	SB	.000000	.000000	.000000
C	2446	C0000750	LL	.000000	.000000	.000000
C	2447	C0000751	BS	.000000	.000000	.000000
C	2448	C0000752	BS	.000000	.000000	.000000
C	2449	C0000753	BS	.000000	.000000	.000000
C	2450	C0000754	BS	.000000	.000000	.000000
C	2451	C0000755	LL	.000000	.000000	.000000
	Number	Column	At	Value	Input Cost	Reduced Cost
C	2452	C0000756	BS	.000000	.000000	.000000
C	2453	C0000757	LL	.000000	.000000	.000000
C	2454	C0000758	BS	.000000	.000000	.000000
C	2455	C0000759	BS	.000000	.000000	.000000
C	2456	C0000760	BS	.000000	.000000	.000000
C	2457	C0000761	BS	.000000	.000000	.000000
C	2458	C0000762	BS	.000000	.000000	.000000
C	2459	C0000763	BS	.000000	.000000	.000000
C	2460	C0000764	BS	.000000	.000000	.000000
C	2461	C0000765	LL	.000000	.000000	.000000
C	2462	C0000766	BS	.000000	.000000	.000000
C	2463	C0000767	BS	.000000	.000000	.000000
C	2464	C0000768	BS	.000000	.000000	.000000
C	2465	C0000769	LL	.000000	.000000	.000000
C	2466	C0000770	SB	.000000	.000000	.000000
C	2467	C0000771	SB	.000000	.000000	.000000
C	2468	C0000772	BS	.000000	.000000	.000000
C	2469	C0000773	BS	.000000	.000000	.000000
C	2470	C0000774	BS	.000000	.000000	.000000
C	2471	C0000775	BS	.000000	.000000	.000000
C	2472	C0000776	LL	.000000	.000000	.000000
	Number	Column	At	Value	Input Cost	Reduced Cost
C	2473	C0000777	LL	.000000	.000000	.000000
C	2474	C0000778	LL	.000000	.000000	.000000
C	2475	C0000779	LL	.000000	.000000	.000000
C	2476	C0000780	LL	.000000	.000000	.000000
C	2477	C0000781	BS	.000000	.000000	.000000
C	2478	C0000782	BS	.000000	.000000	.000000
C	2479	C0000783	BS	.000000	.000000	.000000
C	2480	C0000784	BS	.000000	.000000	.000000
C	2481	C0000785	BS	.000000	.000000	.000000
C	2482	C0000786	LL	.000000	.000000	.000000
C	2483	C0000787	LL	.000000	.000000	.000000
C	2484	C0000788	LL	.000000	.000000	.000000
C	2485	C0000789	LL	.000000	.000000	.000000
C	2486	C0000790	LL	.000000	.000000	.000000
C	2487	C0000791	BS	.000000	.000000	.000000

C	2488	C0000792	BS	.000000	.000000	.000000
C	2489	C0000793	BS	.000000	.000000	.000000
C	2490	C0000794	BS	.000000	.000000	.000000
C	2491	C0000795	BS	.000000	.000000	.000000
C	2492	C0000796	BS	.000000	.000000	.000000
C	2493	C0000797	BS	.000000	.000000	.000000
	Number	Column	At	Value	Input Cost	Reduced Cost
C	2494	C0000798	BS	.000000	.000000	.000000
C	2495	C0000799	BS	.000000	.000000	.000000
C	2496	C0000800	SB	.000000	.000000	.000000
C	2497	C0000801	BS	.000000	.000000	.000000
C	2498	C0000802	LL	.000000	.000000	.000000
C	2499	C0000803	LL	.000000	.000000	.000000
C	2500	C0000804	LL	.000000	.000000	.000000
C	2501	C0000805	BS	.003090	.000000	.000000
C	2502	C0000806	BS	.003090	.000000	.000000
C	2503	C0000807	BS	.000000	.000000	.000000
C	2504	C0000808	LL	.000000	.000000	.000000
C	2505	C0000809	LL	.000000	.000000	.000000
C	2506	C0000810	LL	.000000	.000000	.000000
C	2507	C0000811	BS	.053860	.000000	.000000
C	2508	C0000812	BS	.000000	.000000	.000000
C	2509	C0000813	BS	.000000	.000000	.000000
C	2510	C0000814	BS	.000000	.000000	.000000
C	2511	C0000815	BS	.000000	.000000	.000000
C	2512	C0000816	LL	.000000	.000000	.000000
C	2513	C0000817	LL	.000000	.000000	.000000
C	2514	C0000818	LL	.000000	.000000	.000000
	Number	Column	At	Value	Input Cost	Reduced Cost
C	2515	C0000819	BS	.000000	.000000	.000000
C	2516	C0000820	LL	.000000	.000000	.000000
C	2517	C0000821	BS	.000000	.000000	.000000
C	2518	C0000822	BS	.000000	.000000	.000000
C	2519	C0000823	BS	.000000	.000000	.000000
C	2520	C0000824	BS	.000000	.000000	.000000
C	2521	C0000825	BS	.000000	.000000	.000000
C	2522	C0000826	BS	.000000	.000000	.000000
C	2523	C0000827	BS	.000000	.000000	.000000
C	2524	C0000828	BS	.000000	.000000	.000000
C	2525	C0000829	LL	.000000	.000000	.000000
C	2526	C0000830	LL	.000000	.000000	.000000
C	2527	C0000831	LL	.000000	.000000	.000000
C	2528	C0000832	BS	.000000	.000000	.000000
C	2529	C0000833	BS	.000000	.000000	.000000
C	2530	C0000834	BS	.000000	.000000	.000000
C	2531	C0000835	LL	.000000	.000000	.000000
C	2532	C0000836	LL	.000000	.000000	.000000
C	2533	C0000837	BS	-1.701062	.000000	.000000
C	2534	C0000838	SB	.000000	.000000	.000000
C	2535	C0000839	BS	.000000	.000000	.000000
	Number	Column	At	Value	Input Cost	Reduced Cost
C	2536	C0000840	BS	-.053860	.000000	.000000
C	2537	C0000841	SB	.000000	.000000	.000000
C	2538	C0000842	SB	.000000	.000000	.000000
C	2539	C0000843	BS	-.042147	.000000	.000000
C	2540	C0000844	BS	-1.677691	.000000	.000000
C	2541	C0000845	BS	.000000	.000000	.000000
C	2542	C0000846	LL	.000000	.000000	.000000
C	2543	C0000847	BS	.000000	.000000	.000000
C	2544	C0000848	BS	.000000	.000000	.000000
C	2545	C0000849	SB	.000000	.000000	.000000
C	2546	C0000850	BS	-2.559266	.000000	.000000
C	2547	C0000851	SB	.000000	.000000	.000000
C	2548	C0000852	LL	.000000	.000000	.000000
C	2549	C0000853	BS	.002365	.000000	.000000
C	2550	C0000854	BS	1.03000E-04	.000000	.000000
C	2551	C0000855	LL	.000000	.000000	.000000
C	2552	C0000856	LL	.000000	.000000	.000000
C	2553	C0000857	BS	.000000	.000000	.000000
C	2554	C0000858	LL	.000000	.000000	.000000
C	2555	C0000859	LL	.000000	.000000	.000000

C	2556	C0000860	BS	-.821407	.000000	.000000
	Number	Column	At	Value	Input Cost	Reduced Cost
C	2557	C0000861	LL	.000000	.000000	.000000
C	2558	C0000862	LL	.000000	.000000	.000000
C	2559	C0000863	BS	.820990	.000000	.000000
C	2560	C0000864	LL	.000000	.000000	.000000
C	2561	C0000865	LL	.000000	.000000	.000000
C	2562	C0000866	LL	.000000	.000000	.000000
C	2563	C0000867	LL	.000000	.000000	.000000
C	2564	C0000868	LL	.000000	.000000	.000000
C	2565	C0000869	BS	.000000	.000000	.000000
C	2566	C0000870	BS	.000000	.000000	.000000
C	2567	C0000871	BS	-.002432	.000000	.000000
C	2568	C0000872	BS	.000000	.000000	.000000
C	2569	C0000873	BS	.000000	.000000	.000000
C	2570	C0000874	BS	.002432	.000000	.000000
C	2571	C0000875	BS	.000000	.000000	.000000
C	2572	C0000876	BS	.000000	.000000	.000000
C	2573	C0000877	BS	.000000	.000000	.000000
C	2574	C0000878	BS	.000000	.000000	.000000
C	2575	C0000879	BS	.044579	.000000	.000000
C	2576	C0000880	SB	.000000	.000000	.000000
C	2577	C0000881	BS	.000000	.000000	.000000
	Number	Column	At	Value	Input Cost	Reduced Cost
C	2578	C0000882	LL	.000000	.000000	.000000
C	2579	C0000883	BS	.016205	.000000	.000000
C	2580	C0000884	LL	.000000	.000000	.000000
C	2581	C0000885	LL	.000000	.000000	.000000
C	2582	C0000886	LL	.000000	.000000	.000000
C	2583	C0000887	BS	.000000	.000000	.000000
C	2584	C0000888	BS	.000000	.000000	.000000
C	2585	C0000889	LL	.000000	.000000	.000000
C	2586	C0000890	LL	.000000	.000000	.000000
C	2587	C0000891	LL	.000000	.000000	.000000
C	2588	C0000892	LL	.000000	.000000	.000000
C	2589	C0000893	LL	.000000	.000000	.000000
C	2590	C0000894	LL	.000000	.000000	.000000
C	2591	C0000895	LL	.000000	.000000	.000000
C	2592	C0000896	BS	.000000	.000000	.000000
C	2593	C0000897	BS	.000000	.000000	.000000
C	2594	C0000898	LL	.000000	.000000	.000000
C	2595	C0000899	LL	.000000	.000000	.000000
C	2596	C0000900	BS	.000000	.000000	.000000
C	2597	C0000901	BS	.000000	.000000	.000000
C	2598	C0000902	LL	.000000	.000000	.000000
	Number	Column	At	Value	Input Cost	Reduced Cost
C	2599	C0000903	LL	.000000	.000000	.000000
C	2600	C0000904	LL	.000000	.000000	.000000
C	2601	C0000905	LL	.000000	.000000	.000000
C	2602	C0000906	BS	.000000	.000000	.000000
C	2603	C0000907	BS	.000000	.000000	.000000
C	2604	C0000908	LL	.000000	.000000	.000000
C	2605	C0000909	BS	.000000	.000000	.000000
C	2606	C0000910	LL	.000000	.000000	.000000
C	2607	C0000911	BS	.000000	.000000	.000000
C	2608	C0000912	LL	.000000	.000000	.000000
C	2609	C0000913	SB	.000000	.000000	.000000
C	2610	C0000914	SB	.000000	.000000	.000000
C	2611	C0000915	SB	.000000	.000000	.000000
C	2612	C0000916	SB	.000000	.000000	.000000
C	2613	C0000917	BS	.119432	.000000	.000000
C	2614	C0000918	BS	.821407	.000000	.000000
C	2615	C0000919	BS	.000000	.000000	.000000
C	2616	C0000920	BS	.003787	.000000	.000000
C	2617	C0000921	BS	.000000	.000000	.000000
C	2618	C0000922	BS	.000000	.000000	.000000
C	2619	C0000923	BS	.000000	.000000	.000000
	Number	Column	At	Value	Input Cost	Reduced Cost
C	2620	C0000924	LL	.000000	.000000	.000000
C	2621	C0000925	SB	.000000	.000000	.000000
C	2622	C0000926	BS	.000000	.000000	.000000

	Number	Column	At	Value	Input Cost	Reduced Cost
C	2623	C0000927	SB	.000000	.000000	.000000
C	2624	C0000928	BS	.000000	.000000	.000000
C	2625	C0000929	BS	.000000	.000000	.000000
C	2626	C0000930	SB	.000000	.000000	.000000
C	2627	C0000931	BS	.315910	.000000	.000000
C	2628	C0000932	SB	.000000	.000000	.000000
C	2629	C0000933	LL	.000000	.000000	.000000
C	2630	C0000934	LL	.000000	.000000	.000000
C	2631	C0000935	LL	.000000	.000000	.000000
C	2632	C0000936	LL	.000000	.000000	.000000
C	2633	C0000937	BS	.119432	.000000	.000000
C	2634	C0000938	BS	.000000	.000000	.000000
C	2635	C0000939	BS	.000000	.000000	.000000
C	2636	C0000940	BS	.000000	.000000	.000000
C	2637	C0000941	LL	.000000	.000000	.000000
C	2638	C0000942	BS	.000000	.000000	.000000
C	2639	C0000943	LL	.000000	.000000	.000000
C	2640	C0000944	BS	.000000	.000000	.000000
	Number	Column	At	Value	Input Cost	Reduced Cost
C	2641	C0000945	BS	.000000	.000000	.000000
C	2642	C0000946	LL	.000000	.000000	.000000
C	2643	C0000947	LL	.000000	.000000	.000000
C	2644	C0000948	BS	.000000	.000000	.000000
C	2645	C0000949	BS	.000000	.000000	.000000
C	2646	C0000950	BS	.000000	.000000	.000000
C	2647	C0000951	BS	.000000	.000000	.000000
C	2648	C0000952	BS	.000000	.000000	.000000
C	2649	C0000953	BS	.107719	.000000	.000000
C	2650	C0000954	SB	.000000	.000000	.000000
C	2651	C0000955	BS	.003587	.000000	.000000
C	2652	C0000956	BS	.002432	.000000	.000000
C	2653	C0000957	LL	.000000	.000000	.000000
C	2654	C0000958	LL	.000000	.000000	.000000
C	2655	C0000959	LL	.000000	.000000	.000000
C	2656	C0000960	BS	-.107719	.000000	.000000
C	2657	C0000961	LL	.000000	.000000	.000000
C	2658	C0000962	BS	.000000	.000000	.000000
C	2659	C0000963	BS	.000000	.000000	.000000
C	2660	C0000964	BS	.009270	.000000	.000000
C	2661	C0000965	BS	.009270	.000000	.000000
	Number	Column	At	Value	Input Cost	Reduced Cost
C	2662	C0000966	LL	.000000	.000000	.000000
C	2663	C0000967	LL	.000000	.000000	.000000
C	2664	C0000968	LL	.000000	.000000	.000000
C	2665	C0000969	LL	.000000	.000000	.000000
C	2666	C0000970	LL	.000000	.000000	.000000
C	2667	C0000971	LL	.000000	.000000	.000000
C	2668	C0000972	BS	-.119432	.000000	.000000
C	2669	C0000973	BS	-.119432	.000000	.000000
C	2670	C0000974	BS	.821407	.000000	.000000
C	2671	C0000975	BS	.000000	.000000	.000000
C	2672	C0000976	BS	.000000	.000000	.000000
C	2673	C0000977	BS	.000000	.000000	.000000
C	2674	C0000978	BS	.000000	.000000	.000000
C	2675	C0000979	LL	.000000	.000000	.000000
C	2676	C0000980	LL	.000000	.000000	.000000
C	2677	C0000981	LL	.000000	.000000	.000000
C	2678	C0000982	BS	.000000	.000000	.000000
C	2679	C0000983	SB	.000000	.000000	.000000
C	2680	C0000984	LL	.000000	.000000	.000000
C	2681	C0000985	BS	.000000	.000000	.000000
C	2682	C0000986	BS	.009270	.000000	.000000
	Number	Column	At	Value	Input Cost	Reduced Cost
C	2683	C0000987	BS	.007306	.000000	.000000
C	2684	C0000988	LL	.000000	.000000	.000000
C	2685	C0000989	LL	.000000	.000000	.000000
C	2686	C0000990	BS	.000000	.000000	.000000
C	2687	C0000991	LL	.000000	.000000	.000000
C	2688	C0000992	LL	.000000	.000000	.000000
C	2689	C0000993	BS	.000000	.000000	.000000
C	2690	C0000994	BS	.000000	.000000	.000000

	Number	Column	At	Value	Input Cost	Reduced Cost
C	2691	C0000995	BS	.000000	.000000	.000000
C	2692	C0000996	LL	.000000	.000000	.000000
C	2693	C0000997	LL	.000000	.000000	.000000
C	2694	C0000998	BS	1.03000E-04	.000000	.000000
C	2695	C0000999	LL	.000000	.000000	.000000
C	2696	C0001000	BS	.000000	.000000	.000000
C	2697	C0001001	BS	.000000	.000000	.000000
C	2698	C0001002	BS	.000000	.000000	.000000
C	2699	C0001003	LL	.000000	.000000	.000000
C	2700	C0001004	LL	.000000	.000000	.000000
C	2701	C0001005	BS	.000000	.000000	.000000
C	2702	C0001006	LL	.000000	.000000	.000000
C	2703	C0001007	LL	.000000	.000000	.000000
	Number	Column	At	Value	Input Cost	Reduced Cost
C	2704	C0001008	LL	.000000	.000000	.000000
C	2705	C0001009	SB	.000000	.000000	.000000
C	2706	C0001010	SB	.000000	.000000	.000000
C	2707	C0001011	LL	.000000	.000000	.000000
C	2708	C0001012	LL	.000000	.000000	.000000
C	2709	C0001013	LL	.000000	.000000	.000000
C	2710	C0001014	LL	.000000	.000000	.000000
C	2711	C0001015	LL	.000000	.000000	.000000
C	2712	C0001016	BS	3.51000E-04	.000000	.000000
C	2713	C0001017	BS	3.51000E-04	.000000	.000000
C	2714	C0001018	SB	.000000	.000000	.000000
C	2715	C0001019	SB	.000000	.000000	.000000
C	2716	C0001020	SB	.000000	.000000	.000000
C	2717	C0001021	SB	.000000	.000000	.000000
C	2718	C0001022	SB	.000000	.000000	.000000
C	2719	C0001023	SB	.000000	.000000	.000000
C	2720	C0001024	SB	.000000	.000000	.000000
C	2721	C0001025	SB	.000000	.000000	.000000
C	2722	C0001026	SB	.000000	.000000	.000000
C	2723	C0001027	BS	.114350	.000000	.000000
C	2724	C0001028	SB	.000000	.000000	.000000
	Number	Column	At	Value	Input Cost	Reduced Cost
C	2725	C0001029	BS	.096481	.000000	.000000
C	2726	C0001030	SB	.000000	.000000	.000000
C	2727	C0001031	SB	.000000	.000000	.000000
C	2728	C0001032	BS	-.053860	.000000	.000000
C	2729	C0001033	LL	.000000	.000000	.000000
C	2730	C0001034	LL	.000000	.000000	.000000
C	2731	C0001035	LL	.000000	.000000	.000000
C	2732	C0001036	LL	.000000	.000000	.000000
C	2733	C0001037	LL	.000000	.000000	.000000
C	2734	C0001038	LL	.000000	.000000	.000000
C	2735	C0001039	LL	.000000	.000000	.000000
C	2736	C0001040	BS	.000000	.000000	.000000
C	2737	C0001041	BS	.000000	.000000	.000000
C	2738	C0001042	LL	.000000	.000000	.000000
C	2739	C0001043	BS	.000000	.000000	.000000
C	2740	C0001044	LL	.000000	.000000	.000000
C	2741	C0001045	LL	.000000	.000000	.000000
C	2742	C0001046	LL	.000000	.000000	.000000
C	2743	C0001047	BS	.000000	.000000	.000000
C	2744	C0001048	LL	.000000	.000000	.000000
C	2745	C0001049	BS	.000000	.000000	.000000
	Number	Column	At	Value	Input Cost	Reduced Cost
C	2746	C0001050	BS	.000000	.000000	.000000
C	2747	C0001051	BS	.000000	.000000	.000000
C	2748	C0001052	SB	.000000	.000000	.000000
C	2749	C0001053	SB	.000000	.000000	.000000
C	2750	C0001054	BS	-.053860	.000000	.000000
C	2751	C0001055	BS	-.053860	.000000	.000000
C	2752	C0001056	LL	.000000	.000000	.000000
C	2753	C0001057	LL	.000000	.000000	.000000
C	2754	C0001058	BS	.000000	.000000	.000000
C	2755	C0001059	BS	.000000	.000000	.000000
C	2756	C0001060	BS	.000000	.000000	.000000
C	2757	C0001061	BS	.023371	.000000	.000000
C	2758	C0001062	BS	.023371	.000000	.000000

C	2759	C0001063	LL	.000000	.000000	.000000
C	2760	C0001064	BS	.000000	.000000	.000000
C	2761	C0001065	BS	.000000	.000000	.000000
C	2762	C0001066	LL	.000000	.000000	.000000
C	2763	C0001067	LL	.000000	.000000	.000000
C	2764	C0001068	LL	.000000	.000000	.000000
C	2765	C0001069	BS	.000000	.000000	.000000
C	2766	C0001070	BS	.000000	.000000	.000000
	Number	Column	At	Value	Input Cost	Reduced Cost
C	2767	C0001071	LL	.000000	.000000	.000000
C	2768	C0001072	BS	.000000	.000000	.000000
C	2769	C0001073	BS	.006019	.000000	.000000
C	2770	C0001074	BS	.000000	.000000	.000000
C	2771	C0001075	BS	4.17000E-04	.000000	.000000
C	2772	C0001076	LL	.000000	.000000	.000000
C	2773	C0001077	LL	.000000	.000000	.000000
C	2774	C0001078	BS	.000000	.000000	.000000
C	2775	C0001079	BS	.000000	.000000	.000000
C	2776	C0001080	BS	.000000	.000000	.000000
C	2777	C0001081	BS	.000000	.000000	.000000
C	2778	C0001082	BS	.000000	.000000	.000000
C	2779	C0001083	LL	.000000	.000000	.000000
C	2780	C0001084	BS	.000000	.000000	.000000
C	2781	C0001085	BS	.000000	.000000	.000000
C	2782	C0001086	BS	.000000	.000000	.000000
C	2783	C0001087	BS	.000000	.000000	.000000
C	2784	C0001088	LL	.000000	.000000	.000000
C	2785	C0001089	BS	.000000	.000000	.000000
C	2786	C0001090	BS	.053849	.000000	.000000
C	2787	C0001091	BS	-.053849	.000000	.000000
	Number	Column	At	Value	Input Cost	Reduced Cost
C	2788	C0001092	LL	.000000	.000000	.000000
C	2789	C0001093	BS	.000000	.000000	.000000
C	2790	C0001094	LL	.000000	.000000	.000000
C	2791	C0001095	BS	.000000	.000000	.000000
C	2792	C0001096	BS	.000000	.000000	.000000
C	2793	C0001097	BS	.000000	.000000	.000000
C	2794	C0001098	BS	.000000	.000000	.000000
C	2795	C0001099	BS	.000000	.000000	.000000
C	2796	C0001100	BS	.000000	.000000	.000000
C	2797	C0001101	BS	.000000	.000000	.000000
C	2798	C0001102	SB	.000000	.000000	.000000
C	2799	C0001103	BS	.000000	.000000	.000000
C	2800	C0001104	BS	.000000	.000000	.000000
C	2801	C0001105	BS	.000000	.000000	.000000
C	2802	C0001106	BS	.000000	.000000	.000000
C	2803	C0001107	BS	.000000	.000000	.000000
C	2804	C0001108	BS	.000000	.000000	.000000
C	2805	C0001109	BS	.000000	.000000	.000000
C	2806	C0001110	LL	.000000	.000000	.000000
C	2807	C0001111	BS	.000000	.000000	.000000
C	2808	C0001112	BS	.000000	.000000	.000000
	Number	Column	At	Value	Input Cost	Reduced Cost
C	2809	C0001113	BS	.000000	.000000	.000000
C	2810	C0001114	BS	.000000	.000000	.000000
C	2811	C0001115	BS	.000000	.000000	.000000
C	2812	C0001116	LL	.000000	.000000	.000000
C	2813	C0001117	BS	.000000	.000000	.000000
C	2814	C0001118	BS	.000000	.000000	.000000
C	2815	C0001119	BS	.000000	.000000	.000000
C	2816	C0001120	BS	.000000	.000000	.000000
C	2817	C0001121	LL	.000000	.000000	.000000
C	2818	C0001122	BS	.000000	.000000	.000000
C	2819	C0001123	BS	.000000	.000000	.000000
C	2820	C0001124	BS	.000000	.000000	.000000
C	2821	C0001125	BS	.000000	.000000	.000000
C	2822	C0001126	BS	.000000	.000000	.000000
C	2823	C0001127	BS	.000000	.000000	.000000
C	2824	C0001128	BS	.000000	.000000	.000000
C	2825	C0001129	BS	.000000	.000000	.000000
C	2826	C0001130	BS	.000000	.000000	.000000

C	2827	C0001131	BS	.000000	.000000	.000000
C	2828	C0001132	BS	.000000	.000000	.000000
C	2829	C0001133	BS	.000000	.000000	.000000
	Number	Column	At	Value	Input Cost	Reduced Cost
C	2830	C0001134	BS	.000000	.000000	.000000
C	2831	C0001135	BS	.000000	.000000	.000000
C	2832	C0001136	BS	.002432	.000000	.000000
C	2833	C0001137	BS	.000000	.000000	.000000
C	2834	C0001138	BS	.000000	.000000	.000000
C	2835	C0001139	LL	.000000	.000000	.000000
C	2836	C0001140	BS	.000000	.000000	.000000
C	2837	C0001141	BS	.119432	.000000	.000000
C	2838	C0001142	BS	.000000	.000000	.000000
C	2839	C0001143	BS	.000000	.000000	.000000
C	2840	C0001144	BS	.000000	.000000	.000000
C	2841	C0001145	BS	.000000	.000000	.000000
C	2842	C0001146	LL	.000000	.000000	.000000
C	2843	C0001147	BS	.000000	.000000	.000000
C	2844	C0001148	BS	.000000	.000000	.000000
C	2845	C0001149	BS	.000000	.000000	.000000
C	2846	C0001150	BS	.000000	.000000	.000000
C	2847	C0001151	BS	.000000	.000000	.000000
C	2848	C0001152	BS	.000000	.000000	.000000
C	2849	C0001153	LL	.000000	.000000	.000000
C	2850	C0001154	LL	.000000	.000000	.000000
	Number	Column	At	Value	Input Cost	Reduced Cost
C	2851	C0001155	BS	.000000	.000000	.000000
C	2852	C0001156	BS	.000000	.000000	.000000
C	2853	C0001157	LL	.000000	.000000	.000000
C	2854	C0001158	BS	.000000	.000000	.000000
C	2855	C0001159	BS	.000000	.000000	.000000
C	2856	C0001160	LL	.000000	.000000	.000000
C	2857	C0001161	LL	.000000	.000000	.000000
C	2858	C0001162	LL	.000000	.000000	.000000
C	2859	C0001163	LL	.000000	.000000	.000000
C	2860	C0001164	LL	.000000	.000000	.000000
C	2861	C0001165	BS	.002432	.000000	.000000
C	2862	C0001166	BS	-.043475	.000000	.000000
C	2863	C0001167	LL	.000000	.000000	.000000
C	2864	C0001168	LL	.000000	.000000	.000000
C	2865	C0001169	LL	.000000	.000000	.000000
C	2866	C0001170	LL	.000000	.000000	.000000
C	2867	C0001171	LL	.000000	.000000	.000000
C	2868	C0001172	LL	.000000	.000000	.000000
C	2869	C0001173	LL	.000000	.000000	.000000
C	2870	C0001174	LL	.000000	.000000	.000000
C	2871	C0001175	LL	.000000	.000000	.000000
	Number	Column	At	Value	Input Cost	Reduced Cost
C	2872	C0001176	LL	.000000	.000000	.000000
C	2873	C0001177	BS	-.003587	.000000	.000000
C	2874	C0001178	LL	.000000	.000000	.000000
C	2875	C0001179	LL	.000000	.000000	.000000
C	2876	C0001180	LL	.000000	.000000	.000000
C	2877	C0001181	LL	.000000	.000000	.000000
C	2878	C0001182	LL	.000000	.000000	.000000
C	2879	C0001183	LL	.000000	.000000	.000000
C	2880	C0001184	LL	.000000	.000000	.000000
C	2881	C0001185	LL	.000000	.000000	.000000
C	2882	C0001186	LL	.000000	.000000	.000000
C	2883	C0001187	LL	.000000	.000000	.000000
C	2884	C0001188	LL	.000000	.000000	.000000
C	2885	C0001189	LL	.000000	.000000	.000000
C	2886	C0001190	LL	.000000	.000000	.000000
C	2887	C0001191	BS	-.009270	.000000	.000000
C	2888	C0001192	BS	.000000	.000000	.000000
C	2889	C0001193	BS	.000000	.000000	.000000
C	2890	C0001194	BS	.053860	.000000	.000000
C	2891	C0001195	BS	-.119432	.000000	.000000
C	2892	C0001196	BS	.000000	.000000	.000000
	Number	Column	At	Value	Input Cost	Reduced Cost
C	2893	C0001197	BS	.000000	.000000	.000000

C	2894	C0001198	LL	.000000	.000000	.000000
C	2895	C0001199	BS	9.24819E-05	.000000	.000000
C	2896	C0001200	BS	.003524	.000000	.000000
C	2897	C0001201	BS	.053849	.000000	.000000
C	2898	C0001202	BS	.009803	.000000	.000000
C	2899	C0001203	LL	.000000	.000000	.000000
C	2900	C0001204	BS	.002624	.000000	.000000
C	2901	C0001205	BS	.173292	.000000	.000000
C	2902	C0001206	LL	.000000	.000000	.000000
C	2903	C0001207	BS	.000000	.000000	.000000
C	2904	C0001208	BS	-.053860	.000000	.000000
C	2905	C0001209	BS	.000000	.000000	.000000
C	2906	C0001210	BS	.063857	.000000	.000000
C	2907	C0001211	BS	.003587	.000000	.000000
C	2908	C0001212	BS	.002432	.000000	.000000
C	2909	C0001213	BS	-.002432	.000000	.000000
C	2910	C0001214	BS	2.630188	.000000	.000000
C	2911	C0001215	BS	.000000	.000000	.000000
C	2912	C0001216	BS	.000000	.000000	.000000
C	2913	C0001217	LL	.000000	.000000	.000000
	Number	Column	At	Value	Input Cost	Reduced Cost
C	2914	C0001218	BS	.000000	.000000	.000000
C	2915	C0001219	BS	.000000	.000000	.000000
C	2916	C0001220	BS	6.74568E-04	.000000	.000000
C	2917	C0001221	BS	-.058064	.000000	.000000
C	2918	C0001222	BS	.000000	.000000	.000000
C	2919	C0001223	LL	.000000	.000000	.000000
C	2920	C0001224	LL	.000000	.000000	.000000
C	2921	C0001225	LL	.000000	.000000	.000000
C	2922	C0001226	LL	.000000	.000000	.000000
C	2923	C0001227	LL	.000000	.000000	.000000
C	2924	C0001228	LL	.000000	.000000	.000000
C	2925	C0001229	BS	.000000	.000000	.000000
C	2926	C0001230	LL	.000000	.000000	.000000
C	2927	C0001231	LL	.000000	.000000	.000000
C	2928	C0001232	BS	.000000	.000000	.000000
C	2929	C0001233	LL	.000000	.000000	.000000
C	2930	C0001234	BS	.002432	.000000	.000000
C	2931	C0001235	LL	.000000	.000000	.000000
C	2932	C0001236	LL	.000000	.000000	.000000
C	2933	C0001237	LL	.000000	.000000	.000000
C	2934	C0001238	LL	.000000	.000000	.000000
	Number	Column	At	Value	Input Cost	Reduced Cost
C	2935	C0001239	LL	.000000	.000000	.000000
C	2936	C0001240	LL	.000000	.000000	.000000
C	2937	C0001241	LL	.000000	.000000	.000000
C	2938	C0001242	LL	.000000	.000000	.000000
C	2939	C0001243	LL	.000000	.000000	.000000
C	2940	C0001244	BS	2.630188	.000000	.000000
C	2941	C0001245	BS	.000000	.000000	.000000
C	2942	C0001246	LL	.000000	.000000	.000000
C	2943	C0001247	BS	.009270	.000000	.000000
C	2944	C0001248	LL	.000000	.000000	.000000
C	2945	C0001249	BS	.000000	.000000	.000000
C	2946	C0001250	BS	.000000	.000000	.000000
C	2947	C0001251	BS	.000000	.000000	.000000
C	2948	C0001252	LL	.000000	.000000	.000000
C	2949	C0001253	BS	.000000	.000000	.000000
C	2950	C0001254	BS	.000000	.000000	.000000
C	2951	C0001255	BS	.000000	.000000	.000000
C	2952	C0001256	LL	.000000	.000000	.000000
C	2953	C0001257	BS	.000000	.000000	.000000
C	2954	C0001258	BS	.000000	.000000	.000000
C	2955	C0001259	BS	.000000	.000000	.000000
	Number	Column	At	Value	Input Cost	Reduced Cost
C	2956	C0001260	BS	.000000	.000000	.000000
C	2957	C0001261	BS	.000000	.000000	.000000
C	2958	C0001262	BS	.000000	.000000	.000000
C	2959	C0001263	LL	.000000	.000000	.000000
C	2960	C0001264	BS	.000000	.000000	.000000
C	2961	C0001265	LL	.000000	.000000	.000000

C	2962	C0001266	BS	.000000	.000000	.000000
C	2963	C0001267	BS	.000000	.000000	.000000
C	2964	C0001268	BS	.000000	.000000	.000000
C	2965	C0001269	LL	.000000	.000000	.000000
C	2966	C0001270	LL	.000000	.000000	.000000
C	2967	C0001271	BS	.000000	.000000	.000000
C	2968	C0001272	LL	.000000	.000000	.000000
C	2969	C0001273	LL	.000000	.000000	.000000
C	2970	C0001274	LL	.000000	.000000	.000000
C	2971	C0001275	BS	.000000	.000000	.000000
C	2972	C0001276	BS	.000000	.000000	.000000
C	2973	C0001277	LL	.000000	.000000	.000000
C	2974	C0001278	BS	.000000	.000000	.000000
C	2975	C0001279	BS	.000000	.000000	.000000
C	2976	C0001280	LL	.000000	.000000	.000000
	Number	Column	At	Value	Input Cost	Reduced Cost
C	2977	C0001281	BS	.000000	.000000	.000000
C	2978	C0001282	LL	.000000	.000000	.000000
C	2979	C0001283	LL	.000000	.000000	.000000
C	2980	C0001284	LL	.000000	.000000	.000000
C	2981	C0001285	BS	.000000	.000000	.000000
C	2982	C0001286	LL	.000000	.000000	.000000
C	2983	C0001287	BS	.000000	.000000	.000000
C	2984	C0001288	BS	.000000	.000000	.000000
C	2985	C0001289	BS	.000000	.000000	.000000
C	2986	C0001290	LL	.000000	.000000	.000000
C	2987	C0001291	BS	.009270	.000000	.000000
C	2988	C0001292	BS	.000000	.000000	.000000
C	2989	C0001293	BS	.000000	.000000	.000000
C	2990	C0001294	BS	.000000	.000000	.000000
C	2991	C0001295	BS	.000000	.000000	.000000
C	2992	C0001296	LL	.000000	.000000	.000000
C	2993	C0001297	BS	.000000	.000000	.000000
C	2994	C0001298	BS	.000000	.000000	.000000
C	2995	C0001299	BS	.000000	.000000	.000000
C	2996	C0001300	BS	.000000	.000000	.000000
C	2997	C0001301	BS	.000000	.000000	.000000
	Number	Column	At	Value	Input Cost	Reduced Cost
C	2998	C0001302	BS	.000000	.000000	.000000
C	2999	C0001303	BS	.000000	.000000	.000000
C	3000	C0001304	BS	.000000	.000000	.000000
C	3001	C0001305	BS	.000000	.000000	.000000
C	3002	C0001306	BS	.000000	.000000	.000000
C	3003	C0001307	BS	.000000	.000000	.000000
C	3004	C0001308	BS	.000000	.000000	.000000
C	3005	C0001309	BS	1.34339E-04	.000000	.000000
C	3006	C0001310	BS	4.43871E-04	.000000	.000000
C	3007	C0001311	BS	.000000	.000000	.000000
C	3008	C0001312	BS	4.17000E-04	.000000	.000000
C	3009	C0001313	LL	.000000	.000000	.000000
C	3010	C0001314	BS	.000000	.000000	.000000
C	3011	C0001315	LL	.000000	.000000	.000000
C	3012	C0001316	BS	.000000	.000000	.000000
C	3013	C0001317	BS	.000000	.000000	.000000
C	3014	C0001318	BS	.000000	.000000	.000000
C	3015	C0001319	BS	.000000	.000000	.000000
C	3016	C0001320	BS	.000000	.000000	.000000
C	3017	C0001321	LL	.000000	.000000	.000000
C	3018	C0001322	BS	.000000	.000000	.000000
	Number	Column	At	Value	Input Cost	Reduced Cost
C	3019	C0001323	SB	.000000	.000000	.000000
C	3020	C0001324	BS	.000000	.000000	.000000
C	3021	C0001325	SB	.000000	.000000	.000000
C	3022	C0001326	BS	.119432	.000000	.000000
C	3023	C0001327	BS	.119432	.000000	.000000
C	3024	C0001328	BS	.119432	.000000	.000000
C	3025	C0001329	BS	.119432	.000000	.000000
C	3026	C0001330	BS	.119432	.000000	.000000
C	3027	C0001331	BS	.050000	.000000	.000000
C	3028	C0001332	SB	.000000	.000000	.000000
C	3029	C0001333	BS	.000000	.000000	.000000

C	3030	C0001334	BS	.050000	.000000	.000000
C	3031	C0001335	LL	.000000	.000000	.000000
C	3032	C0001336	LL	.000000	.000000	.000000
C	3033	C0001337	LL	.000000	.000000	.000000
C	3034	C0001338	LL	.000000	.000000	.000000
C	3035	C0001339	LL	.000000	.000000	.000000
C	3036	C0001340	LL	.000000	.000000	.000000
C	3037	C0001341	LL	.000000	.000000	.000000
C	3038	C0001342	LL	.000000	.000000	.000000
C	3039	C0001343	BS	.000000	.000000	.000000
	Number	Column	At	Value	Input Cost	Reduced Cost
C	3040	C0001344	LL	.000000	.000000	.000000
C	3041	C0001345	SB	.000000	.000000	.000000
C	3042	C0001346	SB	.000000	.000000	.000000
C	3043	C0001347	SB	.000000	.000000	.000000
C	3044	C0001348	SB	.000000	.000000	.000000
C	3045	C0001349	SB	.000000	.000000	.000000
C	3046	C0001350	SB	.000000	.000000	.000000
C	3047	C0001351	SB	.000000	.000000	.000000
C	3048	C0001352	BS	.000000	.000000	.000000
C	3049	C0001353	BS	.000000	.000000	.000000
C	3050	C0001354	LL	.000000	.000000	.000000
C	3051	C0001355	LL	.000000	.000000	.000000
C	3052	C0001356	BS	.000000	.000000	.000000
C	3053	C0001357	BS	.000000	.000000	.000000
C	3054	C0001358	LL	.000000	.000000	.000000
C	3055	C0001359	BS	.000000	.000000	.000000
C	3056	C0001360	BS	.000000	.000000	.000000
C	3057	C0001361	BS	.000000	.000000	.000000
C	3058	C0001362	LL	.000000	.000000	.000000
C	3059	C0001363	LL	.000000	.000000	.000000
C	3060	C0001364	BS	.000000	.000000	.000000
	Number	Column	At	Value	Input Cost	Reduced Cost
C	3061	C0001365	BS	.000000	.000000	.000000
C	3062	C0001366	BS	.000000	.000000	.000000
C	3063	C0001367	BS	.000000	.000000	.000000
C	3064	C0001368	BS	.000000	.000000	.000000
C	3065	C0001369	BS	.000000	.000000	.000000
C	3066	C0001370	LL	.000000	.000000	.000000
C	3067	C0001371	BS	.000000	.000000	.000000
C	3068	C0001372	LL	.000000	.000000	.000000
C	3069	C0001373	LL	.000000	.000000	.000000
C	3070	C0001374	BS	.000000	.000000	.000000
C	3071	C0001375	SB	.000000	.000000	.000000
C	3072	C0001376	BS	.000000	.000000	.000000
C	3073	C0001377	LL	.000000	.000000	.000000
C	3074	C0001378	BS	.000000	.000000	.000000
C	3075	C0001379	LL	.000000	.000000	.000000
C	3076	C0001380	LL	.000000	.000000	.000000
C	3077	C0001381	LL	.000000	.000000	.000000
C	3078	C0001382	LL	.000000	.000000	.000000
C	3079	C0001383	BS	.000000	.000000	.000000
C	3080	C0001384	LL	.000000	.000000	.000000
C	3081	C0001385	BS	.000000	.000000	.000000
	Number	Column	At	Value	Input Cost	Reduced Cost
C	3082	C0001386	BS	.000000	.000000	.000000
C	3083	C0001387	BS	.000000	.000000	.000000
C	3084	C0001388	BS	.000000	.000000	.000000
C	3085	C0001389	LL	.000000	.000000	.000000
C	3086	C0001390	LL	.000000	.000000	.000000
C	3087	C0001391	SB	.000000	.000000	.000000
C	3088	C0001392	BS	.000000	.000000	.000000
C	3089	C0001393	BS	.000000	.000000	.000000
C	3090	C0001394	BS	.000000	.000000	.000000
C	3091	C0001395	BS	-.053860	.000000	.000000
C	3092	C0001396	BS	.020000	.000000	.000000
C	3093	C0001397	LL	.000000	.000000	.000000
C	3094	C0001398	SB	.000000	.000000	.000000
C	3095	C0001399	BS	.000000	.000000	.000000
C	3096	C0001400	BS	.000000	.000000	.000000
C	3097	C0001401	BS	.000000	.000000	.000000

	Number	Column	At	Value	Input Cost	Reduced Cost
C	3098	C0001402	LL	.000000	.000000	.000000
C	3099	C0001403	LL	.000000	.000000	.000000
C	3100	C0001404	BS	.000000	.000000	.000000
C	3101	C0001405	LL	.000000	.000000	.000000
C	3102	C0001406	SB	.000000	.000000	.000000
	Number	Column	At	Value	Input Cost	Reduced Cost
C	3103	C0001407	LL	.000000	.000000	.000000
C	3104	C0001408	BS	.000000	.000000	.000000
C	3105	C0001409	LL	.000000	.000000	.000000
C	3106	C0001410	BS	.000000	.000000	.000000
C	3107	C0001411	BS	.000000	.000000	.000000
C	3108	C0001412	BS	.000000	.000000	.000000
C	3109	C0001413	BS	.000000	.000000	.000000
C	3110	C0001414	BS	.000000	.000000	.000000
C	3111	C0001415	BS	.000000	.000000	.000000
C	3112	C0001416	BS	.000000	.000000	.000000
C	3113	C0001417	LL	.000000	.000000	.000000
C	3114	C0001418	LL	.000000	.000000	.000000
C	3115	C0001419	BS	1.727691	.000000	.000000
C	3116	C0001420	BS	-.043475	.000000	.000000
C	3117	C0001421	BS	.000000	.000000	.000000
C	3118	C0001422	BS	.000000	.000000	.000000
C	3119	C0001423	BS	.000000	.000000	.000000
C	3120	C0001424	BS	-.003587	.000000	.000000
C	3121	C0001425	BS	.002849	.000000	.000000
C	3122	C0001426	BS	.821407	.000000	.000000
C	3123	C0001427	SB	.000000	.000000	.000000
	Number	Column	At	Value	Input Cost	Reduced Cost
C	3124	C0001428	BS	.000000	.000000	.000000
C	3125	C0001429	LL	.000000	.000000	.000000
C	3126	C0001430	BS	.000000	.000000	.000000
C	3127	C0001431	LL	.000000	.000000	.000000
C	3128	C0001432	LL	.000000	.000000	.000000
C	3129	C0001433	LL	.000000	.000000	.000000
C	3130	C0001434	LL	.000000	.000000	.000000
C	3131	C0001435	BS	.000000	.000000	.000000
C	3132	C0001436	BS	.000000	.000000	.000000
C	3133	C0001437	LL	.000000	.000000	.000000
C	3134	C0001438	BS	.000000	.000000	.000000
C	3135	C0001439	LL	.000000	.000000	.000000
C	3136	C0001440	LL	.000000	.000000	.000000
C	3137	C0001441	BS	9.17932E-04	.000000	.000000
C	3138	C0001442	LL	.000000	.000000	.000000
C	3139	C0001443	LL	.000000	.000000	.000000
C	3140	C0001444	BS	.053860	.000000	.000000
C	3141	C0001445	LL	.000000	.000000	.000000
C	3142	C0001446	BS	.357340	.000000	.000000
C	3143	C0001447	LL	.000000	.000000	.000000
C	3144	C0001448	LL	.000000	.000000	.000000
	Number	Column	At	Value	Input Cost	Reduced Cost
C	3145	C0001449	LL	.000000	.000000	.000000
C	3146	C0001450	BS	.135790	.000000	.000000
C	3147	C0001451	BS	.171520	.000000	.000000
C	3148	C0001452	BS	.344812	.000000	.000000
C	3149	C0001453	LL	.000000	.000000	.000000
C	3150	C0001454	LL	.000000	.000000	.000000
C	3151	C0001455	LL	.000000	.000000	.000000
C	3152	C0001456	LL	.000000	.000000	.000000
C	3153	C0001457	BS	1.34339E-04	.000000	.000000
C	3154	C0001458	BS	4.43871E-04	.000000	.000000
C	3155	C0001459	LL	.000000	.000000	.000000
C	3156	C0001460	BS	9.24819E-05	.000000	.000000
C	3157	C0001461	BS	.003524	.000000	.000000
C	3158	C0001462	BS	.009803	.000000	.000000
C	3159	C0001463	LL	.000000	.000000	.000000
C	3160	C0001464	BS	.002624	.000000	.000000
C	3161	C0001465	LL	.000000	.000000	.000000
C	3162	C0001466	BS	4.17000E-04	.000000	.000000
C	3163	C0001467	LL	.000000	.000000	.000000
C	3164	C0001468	LL	.000000	.000000	.000000
C	3165	C0001469	LL	.000000	.000000	.000000

	Number	Column	At	Value	Input Cost	Reduced Cost
C	3166	C0001470	LL	.000000	.000000	.000000
C	3167	C0001471	BS	.042880	.000000	.000000
C	3168	C0001472	LL	.000000	.000000	.000000
C	3169	C0001473	LL	.000000	.000000	.000000
C	3170	C0001474	LL	.000000	.000000	.000000
C	3171	C0001475	LL	.000000	.000000	.000000
C	3172	C0001476	LL	.000000	.000000	.000000
C	3173	C0001477	LL	.000000	.000000	.000000
C	3174	C0001478	LL	.000000	.000000	.000000
C	3175	C0001479	LL	.000000	.000000	.000000
C	3176	C0001480	BS	.003587	.000000	.000000
C	3177	C0001481	BS	.002432	.000000	.000000
C	3178	C0001482	LL	.000000	.000000	.000000
C	3179	C0001483	BS	9.60000E-05	.000000	.000000
C	3180	C0001484	BS	1.25000E-04	.000000	.000000
C	3181	C0001485	LL	.000000	.000000	.000000
C	3182	C0001486	BS	.006415	.000000	.000000
C	3183	C0001487	BS	.053860	.000000	.000000
C	3184	C0001488	BS	1.14000E-04	.000000	.000000
C	3185	C0001489	LL	.000000	.000000	.000000
C	3186	C0001490	LL	.000000	.000000	.000000
	Number	Column	At	Value	Input Cost	Reduced Cost
C	3187	C0001491	LL	.000000	.000000	.000000
C	3188	C0001492	LL	.000000	.000000	.000000
C	3189	C0001493	BS	2.630188	.000000	.000000
C	3190	C0001494	LL	.000000	.000000	.000000
C	3191	C0001495	LL	.000000	.000000	.000000
C	3192	C0001496	LL	.000000	.000000	.000000
C	3193	C0001497	LL	.000000	.000000	.000000
C	3194	C0001498	LL	.000000	.000000	.000000
C	3195	C0001499	BS	.437432	.000000	.000000
C	3196	C0001500	LL	.000000	.000000	.000000
C	3197	C0001501	BS	.044708	.000000	.000000
C	3198	C0001502	BS	.007306	.000000	.000000
C	3199	C0001503	BS	.315910	.000000	.000000
C	3200	C0001504	BS	.053849	.000000	.000000
C	3201	C0001505	LL	.000000	.000000	.000000
C	3202	C0001506	LL	.000000	.000000	.000000
C	3203	C0001507	LL	.000000	.000000	.000000
C	3204	C0001508	BS	1.227989	.000000	.000000
C	3205	C0001509	BS	.075041	.000000	.000000
C	3206	C0001510	LL	.000000	.000000	.000000
C	3207	C0001511	BS	.171520	.000000	.000000
	Number	Column	At	Value	Input Cost	Reduced Cost
C	3208	C0001512	LL	.000000	.000000	.000000
C	3209	C0001513	LL	.000000	.000000	.000000
C	3210	C0001514	LL	.000000	.000000	.000000
C	3211	C0001515	BS	3.55568E-04	.000000	.000000
C	3212	C0001516	BS	.250140	.000000	.000000
C	3213	C0001517	BS	.239420	.000000	.000000
C	3214	C0001518	BS	.119432	.000000	.000000
C	3215	C0001519	LL	.000000	.000000	.000000
C	3216	C0001520	LL	.000000	.000000	.000000
C	3217	C0001521	BS	.050027	.000000	.000000
C	3218	C0001522	BS	.002365	.000000	.000000
C	3219	C0001523	LL	.000000	.000000	.000000
C	3220	C0001524	LL	.000000	.000000	.000000
C	3221	C0001525	LL	.000000	.000000	.000000
C	3222	C0001526	LL	.000000	.000000	.000000
C	3223	C0001527	LL	.000000	.000000	.000000
C	3224	C0001528	LL	.000000	.000000	.000000
C	3225	C0001529	LL	.000000	.000000	.000000
C	3226	C0001530	LL	.000000	.000000	.000000
C	3227	C0001531	BS	6.74568E-04	.000000	.000000
C	3228	C0001532	LL	.000000	.000000	.000000
	Number	Column	At	Value	Input Cost	Reduced Cost
C	3229	C0001533	LL	.000000	.000000	.000000
C	3230	C0001534	LL	.000000	.000000	.000000
C	3231	C0001535	BS	.063857	.000000	.000000
C	3232	C0001536	LL	.000000	.000000	.000000

C	3233	C0001537	BS	.114350	.000000	.000000
C	3234	C0001538	LL	.000000	.000000	.000000
C	3235	C0001539	LL	.000000	.000000	.000000
C	3236	C0001540	LL	.000000	.000000	.000000
C	3237	C0001541	BS	.128640	.000000	.000000
C	3238	C0001542	LL	.000000	.000000	.000000
C	3239	C0001543	LL	.000000	.000000	.000000
C	3240	C0001544	LL	.000000	.000000	.000000
C	3241	C0001545	LL	.000000	.000000	.000000
C	3242	C0001546	LL	.000000	.000000	.000000
C	3243	C0001547	LL	.000000	.000000	.000000
C	3244	C0001548	BS	.267557	.000000	.000000
C	3245	C0001549	BS	.020000	.000000	.000000
C	3246	C0001550	LL	.000000	.000000	.000000
C	3247	C0001551	LL	.000000	.000000	.000000
C	3248	C0001552	LL	.000000	.000000	.000000
C	3249	C0001553	LL	.000000	.000000	.000000
	Number	Column	At	Value	Input Cost	Reduced Cost
C	3250	C0001554	LL	.000000	.000000	.000000
C	3251	C0001555	LL	.000000	.000000	.000000
C	3252	C0001556	BS	.196530	.000000	.000000
C	3253	C0001557	LL	.000000	.000000	.000000
C	3254	C0001558	LL	.000000	.000000	.000000
C	3255	C0001559	BS	.028000	.000000	.000000
C	3256	C0001560	BS	.096481	.000000	.000000
C	3257	C0001561	LL	.000000	.000000	.000000
C	3258	C0001562	LL	.000000	.000000	.000000
C	3259	C0001563	LL	.000000	.000000	.000000
C	3260	C0001564	BS	.257280	.000000	.000000
C	3261	C0001565	LL	.000000	.000000	.000000
C	3262	C0001566	BS	.000000	.000000	.000000
C	3263	C0001567	LL	.000000	.000000	.000000
C	3264	C0001568	LL	.000000	.000000	.000000
C	3265	C0001569	BS	1.50000E-05	.000000	.000000
C	3266	C0001570	BS	2.67568E-04	.000000	.000000
C	3267	C0001571	BS	.053860	.000000	.000000
C	3268	C0001572	LL	.000000	.000000	.000000
C	3269	C0001573	BS	.000000	.000000	.000000
C	3270	C0001574	BS	.000000	.000000	.000000
	Number	Column	At	Value	Input Cost	Reduced Cost
C	3271	C0001575	LL	.000000	.000000	.000000
C	3272	C0001576	BS	.000000	.000000	.000000
C	3273	C0001577	LL	.000000	.000000	.000000
C	3274	C0001578	BS	.000000	.000000	.000000
C	3275	C0001579	BS	.000000	.000000	.000000
C	3276	C0001580	BS	.000000	.000000	.000000
C	3277	C0001581	LL	.000000	.000000	.000000
C	3278	C0001582	LL	.000000	.000000	.000000
C	3279	C0001583	LL	.000000	.000000	.000000
C	3280	C0001584	BS	.000000	.000000	.000000
C	3281	C0001585	BS	.000000	.000000	.000000
C	3282	C0001586	BS	1.000000	.000000	.000000
C	3283	C0001587	BS	.000000	.000000	.000000
C	3284	C0001588	LL	.000000	.000000	.000000
C	3285	C0001589	LL	.000000	.000000	.000000
C	3286	C0001590	BS	.000000	.000000	.000000
C	3287	C0001591	LL	.000000	.000000	.000000
C	3288	C0001592	LL	.000000	.000000	.000000
C	3289	C0001593	LL	.000000	.000000	.000000
C	3290	C0001594	BS	.000000	.000000	.000000
C	3291	C0001595	LL	.000000	.000000	.000000
	Number	Column	At	Value	Input Cost	Reduced Cost
C	3292	C0001596	BS	.000000	.000000	.000000
C	3293	C0001597	LL	.000000	.000000	.000000
C	3294	C0001598	BS	.000000	.000000	.000000
C	3295	C0001599	BS	.000000	.000000	.000000
C	3296	C0001600	BS	.000000	.000000	.000000
C	3297	C0001601	BS	.058064	.000000	.000000
C	3298	C0001602	LL	.000000	.000000	.000000
C	3299	C0001603	BS	.000000	.000000	.000000
C	3300	C0001604	BS	.000000	.000000	.000000

C	3301	C0001605	BS	.000000	.000000	.000000
C	3302	C0001606	BS	.000000	.000000	.000000
C	3303	C0001607	BS	.000000	.000000	.000000
C	3304	C0001608	BS	.000000	.000000	.000000
C	3305	C0001609	BS	.000000	.000000	.000000
C	3306	C0001610	BS	.000000	.000000	.000000
C	3307	C0001611	LL	.000000	.000000	.000000
C	3308	C0001612	LL	.000000	.000000	.000000
C	3309	C0001613	BS	.000000	.000000	.000000
C	3310	C0001614	LL	.000000	.000000	.000000
C	3311	C0001615	LL	.000000	.000000	.000000
C	3312	C0001616	BS	.000000	.000000	.000000
	Number	Column	At	Value	Input Cost	Reduced Cost
C	3313	C0001617	LL	.000000	.000000	.000000
C	3314	C0001618	LL	.000000	.000000	.000000
C	3315	C0001619	LL	.000000	.000000	.000000
C	3316	C0001620	BS	.000000	.000000	.000000
C	3317	C0001621	BS	.000000	.000000	.000000
C	3318	C0001622	BS	.000000	.000000	.000000
C	3319	C0001623	BS	.000000	.000000	.000000
C	3320	C0001624	LL	.000000	.000000	.000000
C	3321	C0001625	BS	.000000	.000000	.000000
C	3322	C0001626	BS	.000000	.000000	.000000
C	3323	C0001627	BS	.000000	.000000	.000000
C	3324	C0001628	BS	.000000	.000000	.000000
C	3325	C0001629	BS	.000000	.000000	.000000
C	3326	C0001630	LL	.000000	.000000	.000000
C	3327	C0001631	BS	.000000	.000000	.000000
C	3328	C0001632	LL	.000000	.000000	.000000
C	3329	C0001633	LL	.000000	.000000	.000000
C	3330	C0001634	LL	.000000	.000000	.000000
C	3331	C0001635	LL	.000000	.000000	.000000
C	3332	C0001636	BS	2.06000E-04	.000000	.000000
C	3333	C0001637	BS	.000000	.000000	.000000
	Number	Column	At	Value	Input Cost	Reduced Cost
C	3334	C0001638	BS	.000000	.000000	.000000
C	3335	C0001639	LL	.000000	.000000	.000000
C	3336	C0001640	LL	.000000	.000000	.000000
C	3337	C0001641	BS	.009270	.000000	.000000
C	3338	C0001642	LL	.000000	.000000	.000000
C	3339	C0001643	BS	.000000	.000000	.000000
C	3340	C0001644	BS	.000000	.000000	.000000
C	3341	C0001645	BS	.000000	.000000	.000000
C	3342	C0001646	LL	.000000	.000000	.000000
C	3343	C0001647	LL	.000000	.000000	.000000
C	3344	C0001648	LL	.000000	.000000	.000000
C	3345	C0001649	LL	.000000	.000000	.000000
C	3346	C0001650	BS	.000000	.000000	.000000
C	3347	C0001651	BS	.000000	.000000	.000000
C	3348	C0001652	LL	.000000	.000000	.000000
C	3349	C0001653	LL	.000000	.000000	.000000
C	3350	C0001654	BS	.000000	.000000	.000000
C	3351	C0001655	BS	.000000	.000000	.000000
C	3352	C0001656	BS	.000000	.000000	.000000
C	3353	C0001657	BS	.000000	.000000	.000000
C	3354	C0001658	BS	.000000	.000000	.000000
	Number	Column	At	Value	Input Cost	Reduced Cost
C	3355	C0001659	BS	.000000	.000000	.000000
C	3356	C0001660	BS	2.637026	.000000	.000000
C	3357	C0001661	BS	.000000	.000000	.000000
C	3358	C0001662	LL	.000000	.000000	.000000
C	3359	C0001663	BS	.000000	.000000	.000000
C	3360	C0001664	BS	.000000	.000000	.000000
C	3361	C0001665	BS	.000000	.000000	.000000
C	3362	C0001666	LL	.000000	.000000	.000000
C	3363	C0001667	BS	.000000	.000000	.000000
C	3364	C0001668	LL	.000000	.000000	.000000
C	3365	C0001669	BS	.000000	.000000	.000000
C	3366	C0001670	BS	2.559266	.000000	.000000
C	3367	C0001671	BS	.000000	.000000	.000000
C	3368	C0001672	LL	.000000	.000000	.000000

C	3369	C0001673	BS	.000000	.000000	.000000
C	3370	C0001674	BS	.000000	.000000	.000000
C	3371	C0001675	BS	.000000	.000000	.000000
C	3372	C0001676	BS	.000000	.000000	.000000
C	3373	C0001677	BS	.000000	.000000	.000000
C	3374	C0001678	BS	.000000	.000000	.000000
C	3375	C0001679	LL	.000000	.000000	.000000
	Number	Column	At	Value	Input Cost	Reduced Cost
C	3376	C0001680	LL	.000000	.000000	.000000
C	3377	C0001681	BS	.000000	.000000	.000000
C	3378	C0001682	BS	.000000	.000000	.000000
C	3379	C0001683	BS	.000000	.000000	.000000
C	3380	C0001684	BS	.119432	.000000	.000000
C	3381	C0001685	BS	.000000	.000000	.000000
C	3382	C0001686	BS	.000000	.000000	.000000
C	3383	C0001687	LL	.000000	.000000	.000000
C	3384	C0001688	BS	.002432	.000000	.000000
C	3385	C0001689	BS	.000000	.000000	.000000
C	3386	C0001690	LL	.000000	.000000	.000000
C	3387	C0001691	LL	.000000	.000000	.000000
C	3388	C0001692	BS	.000000	.000000	.000000
C	3389	C0001693	BS	.000000	.000000	.000000
C	3390	C0001694	BS	.000000	.000000	.000000
C	3391	C0001695	LL	.000000	.000000	.000000
C	3392	C0001696	BS	.000000	.000000	.000000
C	3393	C0001697	LL	.000000	.000000	.000000
C	3394	C0001698	BS	.000000	.000000	.000000
C	3395	C0001699	BS	.000000	.000000	.000000
C	3396	C0001700	LL	.000000	.000000	.000000
	Number	Column	At	Value	Input Cost	Reduced Cost
C	3397	C0001701	BS	1.000000	.000000	.000000
C	3398	C0001702	BS	1.000000	1.000000	.000000
C	3399	C0001703	LL	.000000	.000000	.000000
C	3400	C0001704	LL	.000000	.000000	.000000
C	3401	C0001705	BS	1.000000	.000000	.000000

##
