# Supplementary material for: Reply to “Do genome-scale models need exact solvers or clearer standards?”
Source: Mol Syst Biol. 2015 Oct 14;11(10):830. doi: 10.15252/msb.20156548 (PMC4631201; doi:10.15252/msb.20156548)
Supplement: Supplementary file 3 — Dataset EV3 [file msb0011-0830-sd3.zip › msb0011-0830-sd3/Dataset3/Example1-NEOSsolvers/NEOS-Gurobi.pdf]

```
*************************************************************

   NEOS Server Version 5.0
   Job#     : 3432330
   Password : knbONyel
   Solver   : lp:Gurobi:MPS
   Start    : 2015-01-01 15:35:04
   End      : 2015-01-01 15:35:09
   Host     : NEOS HTCondor Pool

   Disclaimer:

   This information is provided without any express or
   implied warranty. In particular, there is no warranty
   of any kind concerning the fitness of this
   information  for any particular purpose.
*************************************************************
/opt/gurobi/latest/linux64/lib:/opt/gurobi/latest/linux64/lib:
Load Avg: ( 0.0 , 0.01 , 0.01 )
Read MPS format model from file model.mps
Reading time = 0.00 seconds
SC4cInfeasible: 1694 rows, 1706 columns, 5432 nonzeros
Changed value of parameter Threads to 1
   Prev: 0   Min: 0   Max: 24   Default: 0
Optimize a model with 1694 rows, 1706 columns and 5432 nonzeros
Presolve removed 1691 rows and 1706 columns
Presolve time: 0.01s

Solved in 0 iterations and 0.01 seconds
Infeasible model
The model cannot be solved because it is infeasible or unbounded
```
