# Supplementary material for: Reply to “Do genome-scale models need exact solvers or clearer standards?”
Source: Mol Syst Biol. 2015 Oct 14;11(10):830. doi: 10.15252/msb.20156548 (PMC4631201; doi:10.15252/msb.20156548)
Supplement: Supplementary file 3 — Dataset EV3 [file msb0011-0830-sd3.zip › msb0011-0830-sd3/Dataset3/Example1-NEOSsolvers/NEOS-QSOpt_ex.pdf]

```
*************************************************************

   NEOS Server Version 5.0
   Job#     : 3432333
   Password : naXzGIiQ
   Solver   : milp:qsopt_ex:MPS
   Start    : 2015-01-01 15:40:51
   End      : 2015-01-01 15:41:00
   Host     : thales.la.asu.edu

   Disclaimer:

   This information is provided without any express or
   implied warranty. In particular, there is no warranty
   of any kind concerning the fitness of this
   information  for any particular purpose.
*************************************************************
Host: thales, Current process id: 24616
Reading problem from sample.mps
mpf_ILLlp_add_logicals ...
Time for SOLVER_READ: 0.01 seconds.
starting mpf_ILLsimplex on scaled_lp...
Problem has 1694 rows and 3400 cols and 7126 nonzeros
starting primal phase I
(0): primal infeas = 59.3050000 0.59304999999999999971578291e2
(100): primal infeas =  5.9535560 0.59535559999999999908913655e1
(200): primal infeas =  2.5414380 0.25414379999999999907226782e1
(300): primal infeas =  2.4761717 0.24761716666666665756104347e1
(400): primal infeas =  2.4680383 0.24680383345193324234551O9e1
(500): primal infeas =  0.0107235 0.10723531494000000066563656e-1
completed mpf_ILLsimplex
scaled_lp: Primal Infeasible
time = 0.358, pI = 529, pII = 0, dI = 0, dII = 0, no primal soln
starting mpf_ILLsimplex on SC4cInfeasible...
Problem has 1694 rows and 3400 cols and 7126 nonzeros
starting primal phase I
(0): primal infeas =  0.0000000 0.15999000000265784946744O5e-7
completed mpf_ILLsimplex
SC4cInfeasible: Primal Infeasible
time = 0.020, pI = 1, pII = 0, dI = 0, dII = 0, no primal soln
LP Value: 0.000000, status 2
Time for SOLVER: 0.39 seconds.
FAILURE: mpf_ILLsimplex_solution
        in function mpf_ILLsimplex_solution in file QSopt_alt/mpf_simplex.c line 579.
FAILURE: mpf_ILLlib_solution
        in function mpf_ILLlib_solution in file QSopt_alt/mpf_lib.c line 358.
FAILURE: mpf_ILLlib_get_x
        in function mpf_ILLlib_get_x in file QSopt_alt/mpf_lib.c line 372.
FAILURE: mpf_ILLlib_print_x
        in function mpf_ILLlib_print_x in file QSopt_alt/mpf_lib.c line 3877.


*** You chose the QSopt_EX solver ***
```
