# Supplementary material for: Reply to “Do genome-scale models need exact solvers or clearer standards?”
Source: Mol Syst Biol. 2015 Oct 14;11(10):830. doi: 10.15252/msb.20156548 (PMC4631201; doi:10.15252/msb.20156548)
Supplement: Supplementary file 3 — Dataset EV3 [file msb0011-0830-sd3.zip › msb0011-0830-sd3/Dataset3/Example1-NEOSsolvers/NEOS-SYMPHONY.pdf]

```
*************************************************************

   NEOS Server Version 5.0
   Job#     : 3432326
   Password : bWvRNDko
   Solver   : milp:SYMPHONY:MPS
   Start    : 2015-01-01 15:33:19
   End      : 2015-01-01 15:33:44
   Host     : NEOS HTCondor Pool

   Disclaimer:

   This information is provided without any express or
   implied warranty. In particular, there is no warranty
   of any kind concerning the fitness of this
   information  for any particular purpose.
*************************************************************
Executing on neos-3.neos-server.org

==  Welcome to the SYMPHONY MILP Solver
==  Copyright 2000-2011 Ted Ralphs and others
==  All Rights Reserved.
==  Distributed under the Eclipse Public License 1.0
==  Version: 5.4.7
==  Build Date: Mar  4 2013
==  Revision Number: 2068

Reading input file...


Starting Preprocessing...
Preprocessing finished...
         with no modifications...
Problem has
         1694 constraints
         1706 variables
         5432 nonzero coefficients

Total Presolve Time: 0.001107...

Solving...

solving root lp relaxation
The LP value is: 1.000 [0,90]


****************************************************
* Optimal Solution Found                           *
* Now displaying stats and best solution found...  *
****************************************************

======================= CP Timing ===========================
  Cut Pool                  0.000
====================== LP/CG Timing =========================
  LP Solution Time          0.013
```

```
  LP Setup Time             0.000
  Variable Fixing           0.000
  Pricing                   0.000
  Strong Branching          0.000
  Separation                0.000
  Primal Heuristics         0.000
  Communication             0.000
  Total User Time              0.021
  Total Wallclock Time         0.018

====================== Statistics =========================
Number of created nodes :       1
Number of analyzed nodes:       1
Depth of tree:                  0
Size of the tree:               1
Number of solutions found:      1
Number of solutions in pool:    1
Number of Chains:               1
Number of Diving Halts:         0
Number of cuts in cut pool:     0

======================= LP Solver =========================
Number of times LP solver called:               1
Number of calls from feasibility pump:          0
Number of calls from strong branching:          0
Number of solutions found by LP solve:          1
Number of bounds changed by strong branching:   0
Number of nodes pruned by strong branching:     0

==================== Feasibility Pump =====================
Number of times feasibility pump called:        0
Number of solutions found by feasibility pump:  0
Time spent in feasibility pump:                 0.00

========================== Cuts ==========================
total cuts accepted:                   0
total cuts added to LPs:               0
total cuts deleted from LPs:           0
total gomory cuts generated:           0
total knapsack cuts generated:         0
total oddhole cuts generated:          0
total clique cuts generated:           0
total probing cuts generated:          0
total mir cuts generated:              0
total twomir cuts generated:           0
total flow and cover cuts generated:   0
total rounding cuts generated:         0
total lift and project cuts generated: 0
total landp cuts generated:            0

cuts removed because of bad coeffs:    0
cuts removed because of duplicacy:     0
insufficiently violated cuts:          0

cuts in root:                          0
gomory cuts in root:                   0
knapsack cuts in root:                 0
oddhole cuts in root:                  0
clique cuts in root:                   0
probing cuts in root:                  0
mir cuts in root:                      0
twomir cuts in root:                   0
flow and cover cuts in root:           0
rounding cuts in root:                 0
lift and project cuts in root:         0
landp cuts in root:                    0

time in cut generation: 0.00
time in gomory cuts in 0 calls: 0.00
time in knapsack cuts in 0 calls: 0.00
time in oddhole cuts in 0 calls: 0.00
```

```
time in clique cuts in 0 calls: 0.00
time in probing cuts in 0 calls: 0.00
time in mir cuts in 0 calls: 0.00
time in twomir cuts in 0 calls: 0.00
time in flow and cover cuts in 0 calls: 0.00
time in rounding cuts in 0 calls: 0.00
time in lift and project cuts in 0 calls: 0.00
time in landp cuts in 0 calls: 0.00
time in redsplit cuts in 0 calls: 0.00
time in checking quality and adding: 0.00

Solution Found: Node 0, Level 0
Solution Cost: 1.0000000000
++++++++++++++++++++++++++++++++++++++++++++++++++++
Column names and values of nonzeros in the solution
++++++++++++++++++++++++++++++++++++++++++++++++++++
C0000005 2.8068731988
C0000007 -14.0473997251
C0000010 0.0167416937
C0000016 0.0178011937
C0000018 0.0178011937
C0000020 0.0178011937
C0000021 0.0178011937
C0000024 -0.0178011937
C0000025 2.7092388874
C0000027 2.7092388874
C0000028 0.0178011937
C0000029 0.0178011937
C0000030 0.0178011937
C0000031 0.0178011937
C0000032 0.0178011937
C0000033 5.2733913438
C0000035 5.6137463975
C0000038 -1.6570520000
C0000039 -0.8214070000
C0000044 -8.2873828975
C0000045 2.8068731988
C0000046 -2.8068731988
C0000047 -11.0619779137
C0000048 0.0964810000
C0000049 0.2572800000
C0000050 2.9644358874
C0000051 0.1965300000
C0000052 0.3573400000
C0000053 0.1715200000
C0000054 0.3574513874
C0000055 0.0750410000
C0000056 0.1715200000
C0000057 0.2501400000
C0000058 -2.6236095000
C0000059 0.1143500000
C0000062 14.1034842200
C0000069 5.6137463975
C0000072 0.1357900000
C0000073 0.0360478063
C0000086 0.0008120000
C0000115 0.0000660000
C0000116 0.0003510000
C0000117 0.0064150000
C0000118 0.0000150000
C0000119 0.0000320000
C0000120 0.0000560000
C0000121 0.0001140000
C0000122 0.0000960000
C0000124 0.0001250000
C0000125 0.0071000000
C0000126 0.0428800000
C0000131 0.0280000000
C0000149 -2.6736365000
C0000151 -2.6736365000
C0000165 -0.0035870000
```

C0000166	0.1194320000
C0000169	0.0034350000
C0000171	0.0163246937
C0000172	0.0163246937
C0000173	0.0163246937
C0000174	0.0161920620
C0000175	0.0160596383
C0000176	0.0157538334
C0000177	2.7098935840
C0000180	0.0010700000
C0000189	0.0028840000
C0000195	0.4678121998
C0000198	0.0028840000
C0000201	0.0000660000
C0000202	0.0003510000
C0000203	1.3533514517
C0000223	2.7067028874
C0000230	0.0009870000
C0000232	2.8068731988
C0000233	-14.0343659938
C0000240	-2.3662576937
C0000243	0.9356243996
C0000247	0.8209900000
C0000248	0.0036650000
C0000270	0.0004170000
C0000276	-0.0130337312
C0000308	0.0163246937
C0000309	0.0163246937
C0000310	0.0163246937
C0000311	0.0161920620
C0000312	0.0160596383
C0000313	0.0157538334
C0000314	2.7098935840
C0000328	0.0000913063
C0000331	0.0163246937
C0000332	0.0163246937
C0000333	0.0163246937
C0000340	0.0001326317
C0000344	0.0001324237
C0000348	0.0003058049
C0000352	-2.6941397506
C0000356	2.7098935840
C0000358	0.0163246937
C0000359	0.0163246937
C0000360	0.0163246937
C0000380	0.0004170000
C0000382	59.6364824310
C0000399	1.1358000000
C0000413	2.7067028874
C0000421	2.7067028874
C0000429	-0.0028840000
C0000438	0.0178011937
C0000439	0.0538490000
C0000440	0.1194320000
C0000447	1.6543200000
C0000448	-1.6543200000
C0000454	0.1286400000
C0000461	0.2394200000
C0000473	0.0028840000
C0000478	0.0028840000
C0000506	-0.0130337312
C0000510	0.0071000000
C0000518	0.5185200000
C0000520	0.0538490000
C0000521	0.0028490000
C0000522	-0.0024320000
C0000534	2.8068731988
C0000550	2.6736365000
C0000551	2.6736365000
C0000552	2.6736365000
C0000554	0.0028840000

C0000558 0.0163246937
C0000559 0.0163246937
C0000560 0.0163246937
C0000561 0.0161920620
C0000562 0.0160596383
C0000563 0.0157538334
C0000564 2.7098935840
C0000642 0.0004170000
C0000669 0.0000660000
C0000670 0.0003510000
C0000677 0.0023650000
C0000679 0.9356243996
C0000680 1.8712487992
C0000681 2.8068731988
C0000689 0.0000660000
C0000692 0.0003510000
C0000693 2.8068731988
C0000695 0.0004170000
C0000706 0.0004170000
C0000707 2.8068731988
C0000712 -2.8068731988
C0000726 2.8021057363
C0000728 2.8068731988
C0000729 -11.2274927951
C0000732 -2.5906422688
C0000743 2.6736365000
C0000745 2.6736365000
C0000746 0.0906348063
C0000751 -14.0343659938
C0000791 14.0343659938
C0000793 0.0163246937
C0000794 0.0163246937
C0000795 0.0163246937
C0000796 0.0161920620
C0000797 0.0160596383
C0000798 0.0157538334
C0000799 2.7098935840
C0000803 0.0096786110
C0000804 2.7092931255
C0000811 0.0130337312
C0000814 0.0028840000
C0000815 2.8068731988
C0000837 -1.6543200000
C0000840 -0.0130337312
C0000841 0.1194320000
C0000842 -1.6543200000
C0000849 -2.4733180751
C0000853 0.0023650000
C0000860 -0.8214070000
C0000866 0.8209900000
C0000883 0.0159990000
C0000886 2.7067028874
C0000895 2.7067028874
C0000913 0.1372331937
C0000918 0.8214070000
C0000920 0.0006970000
C0000953 0.0118674625
C0000955 0.0035870000
C0000956 0.0024320000
C0000957 0.0024320000
C0000960 -0.0118674625
C0000974 0.8214070000
C0000986 2.7092388874
C0000987 0.0071000000
C0001001 2.7067028874
C0001013 0.0200000000
C0001017 0.0003510000
C0001018 -0.0059337312
C0001030 -0.0130337312
C0001037 0.0002060000
C0001052 -0.0059337312

C0001053 -0.0059337312
C0001059 -0.0071000000
C0001073 0.0084510000
C0001075 0.0004170000
C0001082 0.0004170000
C0001096 0.4678121998
C0001097 -8.4206195963
C0001098 5.6137463975
C0001109 5.6137463975
C0001119 0.9356243996
C0001120 1.8712487992
C0001141 -14.0473997251
C0001154 0.0233710000
C0001155 -0.0024320000
C0001162 0.1194320000
C0001166 -0.0436810000
C0001177 -0.0035870000
C0001194 10.8871377413
C0001195 -2.8068731988
C0001199 0.0000913063
C0001208 -5.2733913438
C0001210 0.0906348063
C0001211 0.0035870000
C0001212 0.1194320000
C0001214 2.4875944625
C0001216 -2.6736365000
C0001220 4.5404863881
C0001221 -8.2837294351
C0001244 2.4875944625
C0001247 2.6736365000
C0001291 2.6736365000
C0001301 0.0163246937
C0001304 -0.0001326317
C0001305 -0.0001324237
C0001306 -0.0003058049
C0001307 2.6941397506
C0001308 2.7098935840
C0001311 0.0004170000
C0001319 0.0004170000
C0001331 0.0524320000
C0001337 0.0524320000
C0001353 2.6736365000
C0001419 1.7067520000
C0001420 -0.0461130000
C0001424 -0.0035870000
C0001439 0.0000560000
C0001444 14.0473997251
C0001446 0.3573400000
C0001450 0.1357900000
C0001451 0.1715200000
C0001452 14.1034842200
C0001456 2.6736365000
C0001460 0.0000913063
C0001465 0.0004170000
C0001471 0.0428800000
C0001472 0.1194320000
C0001480 0.0035870000
C0001483 0.0000960000
C0001484 0.0001250000
C0001486 0.0064150000
C0001487 10.8871377413
C0001488 0.0001140000
C0001493 2.4875944625
C0001499 0.3574513874
C0001505 0.0028840000
C0001508 6.8870090648
C0001509 0.0750410000
C0001511 0.1715200000
C0001514 0.9356243996
C0001515 0.0000320000
C0001516 0.2501400000

C0001517	0.2394200000
C0001522	0.0023650000
C0001531	4.5404863881
C0001535	0.0906348063
C0001537	0.1143500000
C0001541	0.1286400000
C0001543	1.8712487992
C0001548	2.9644358874
C0001549	0.0200000000
C0001556	0.1965300000
C0001558	0.0233710000
C0001559	0.0280000000
C0001560	0.0964810000
C0001564	0.2572800000
C0001569	0.0000150000
C0001571	5.2733913438
C0001572	8.2873828975
C0001586	1.0000000000
C0001601	8.2837294351
C0001605	2.6736365000
C0001632	11.0619779137
C0001633	0.0028840000
C0001634	2.3662576937
C0001649	11.2452939888
C0001652	2.6236095000
C0001660	2.5906422688
C0001670	2.4733180751
C0001684	2.8068731988
C0001701	1.0000000000
C0001702	1.0000000000
C0001705	1.0000000000

Home
