# Supplementary material for: Reply to “Do genome-scale models need exact solvers or clearer standards?”
Source: Mol Syst Biol. 2015 Oct 14;11(10):830. doi: 10.15252/msb.20156548 (PMC4631201; doi:10.15252/msb.20156548)
Supplement: Supplementary file 3 — Dataset EV3 [file msb0011-0830-sd3.zip › msb0011-0830-sd3/Dataset3/Example1-NEOSsolvers/NEOS-MOSEK.pdf]

```
*************************************************************

   NEOS Server Version 5.0
   Job#     : 3432332
   Password : JgBaoZRc
   Solver   : lp:MOSEK:MPS
   Start    : 2015-01-01 15:36:16
   End      : 2015-01-01 15:36:21
   Host     : NEOS HTCondor Pool

   Disclaimer:

   This information is provided without any express or
   implied warranty. In particular, there is no warranty
   of any kind concerning the fitness of this
   information  for any particular purpose.
*************************************************************
Executing on neos-2.neos-server.org

MOSEK Version 7.1.0.12 (Build date: 2014-12-12 07:25:47)
Copyright (c) 1998-2014 MOSEK ApS, Denmark. WWW: http://mosek.com

Open file 'model.mps'
Using 'OBJ' as objective vector
Read 5433 number of A nonzeros in 0.01 seconds.
Using 'RHS' as rhs vector
Using 'B0000000' as bound vector

Read summary
  Type             : LO (linear optimization problem)
  Objective sense  : min
  Scalar variables : 1706
  Matrix variables : 0
  Constraints      : 1694
  Cones            : 0
  Time             : 0.0

Computer
  Platform               : Linux/64-X86
  Cores                  : 12

Problem
  Name                   : SC4cInfeasible
  Objective sense        : min
  Type                   : LO (linear optimization problem)
  Constraints            : 1694
  Cones                  : 0
  Scalar variables       : 1706
  Matrix variables       : 0
  Integer variables      : 0

Optimizer started.
Interior-point optimizer started.
Presolve started.
Linear dependency checker started.
Linear dependency checker terminated.
Eliminator started.
Total number of eliminations : 271
Eliminator terminated.
Eliminator started.
Total number of eliminations : 297
Eliminator terminated.
Eliminator - tries                  : 2                 time                   : 0.00
Eliminator - elim's                 : 300
Lin. dep.  - tries                  : 1                 time                   : 0.00
Lin. dep.  - number                 : 1
Presolve terminated. Time: 0.01
Optimizer  - threads                : 12
Optimizer  - solved problem         : the primal
Optimizer  - Constraints            : 26
Optimizer  - Cones                  : 0
Optimizer  - Scalar variables       : 56                conic                  : 0
Optimizer  - Semi-definite variables: 0                 scalarized             : 0
Factor     - setup time             : 0.00              dense det. time        : 0.00
Factor     - ML order time          : 0.00              GP order time          : 0.00
Factor     - nonzeros before factor : 219               after factor           : 233
Factor     - dense dim.             : 0                 flops                  : 5.11e+03
ITE PFEAS    DFEAS    GFEAS    PRSTATUS   POBJ              DOBJ              MU       TIME
0   8.0e+00  1.0e+00  1.0e+00  1.00e+00   1.000000000e+00   1.000000000e+00   1.0e+00  0.01
1   3.0e+00  2.2e+00  1.2e+00  0.00e+00   1.000000000e+00   9.347481360e-01   3.2e+00  0.02
2   2.1e-01  1.5e-01  8.3e-02  1.01e+00   1.000000000e+00   9.931603915e-01   2.2e-01  0.02
3   1.3e-02  9.7e-03  5.2e-03  1.13e+00   1.000000000e+00   9.986231372e-01   1.4e-02  0.02
```

```
4   2.8e-03  2.1e-03  1.1e-03  2.39e+00   1.000000000e+00   9.998448655e-01   3.0e-03  0.02
5   2.6e-05  1.9e-05  1.0e-05  1.54e+00   1.000000000e+00   9.999990436e-01   2.7e-05  0.02
6   7.2e-06  5.3e-06  2.9e-06  9.98e-01   1.000000000e+00   9.999997290e-01   7.7e-06  0.02
7   1.7e-06  1.3e-06  6.9e-07  9.51e-01   1.000000000e+00   9.999999316e-01   1.9e-06  0.02
8   1.6e-07  3.3e-08  1.8e-08  7.99e-01   1.000000000e+00   1.000000000e+00   8.6e-08  0.02
9   5.1e-09  1.1e-10  5.7e-11  -6.25e-01  1.000000000e+00   1.000000055e+00   1.8e-09  0.03
10  2.7e-11  5.6e-13  3.0e-13  -9.99e-01  1.000000000e+00   1.000010615e+00   9.6e-12  0.03
11  2.7e-11  5.6e-13  3.0e-13  -1.00e+00  1.000000000e+00   1.000010615e+00   9.6e-12  0.03
12  1.9e-12  6.0e-15  3.2e-15  -1.00e+00  1.000000000e+00   1.000971759e+00   1.0e-13  0.03
13  1.9e-12  6.0e-15  3.2e-15  -1.00e+00  1.000000000e+00   1.000971759e+00   1.0e-13  0.03
14  9.7e-15  8.7e-14  1.7e-17  -1.00e+00  1.000000000e+00   1.188317251e+00   5.4e-16  0.03
15  1.3e-15  3.6e-15  8.5e-20  -1.00e+00  1.000000000e+00   3.792857178e+01   2.7e-18  0.03
Basis identification started.
Primal basis identification phase started.
ITER      TIME
23        0.00
Primal basis identification phase terminated. Time: 0.00
Dual basis identification phase started.
ITER      TIME
9         0.00
Dual basis identification phase terminated. Time: 0.00
Dual simplex reoptimization started.
ITER      DEGITER(%)  PFEAS       DFEAS       POBJ                  DOBJ                  TIME
0         0.00        NA          0.00e+00    NA                    1.000000000000e+00    0.00
7         87.50       NA          0.00e+00    NA                    1.000000000000e+00    0.00
Dual simplex reoptimization terminated. Time: 0.00
Primal simplex reoptimization started.
ITER      DEGITER(%)  PFEAS       DFEAS       POBJ                  DOBJ                  TIME
0         0.00        0.00e+00    NA          1.000000000000e+00    NA                    0.01
2         0.00        0.00e+00    NA          1.000000000000e+00    NA                    0.01
Primal simplex reoptimization terminated. Time: 0.01
Simplex reoptimization terminated. Time: 0.02
Basis identification terminated. Time: 0.02
Interior-point optimizer terminated. Time: 0.05.

Optimizer terminated. Time: 0.07

Interior-point solution summary
  Problem status  : PRIMAL_AND_DUAL_FEASIBLE
  Solution status : OPTIMAL
  Primal.  obj: 1.0000000000e+00    Viol.  con: 2e-04    var: 0e+00
  Dual.    obj: 3.7928571775e+01    Viol.  con: 0e+00    var: 1e-05

Basic solution summary
  Problem status  : PRIMAL_AND_DUAL_FEASIBLE
  Solution status : OPTIMAL
  Primal.  obj: 1.0000000000e+00    Viol.  con: 3e-14    var: 2e-08
  Dual.    obj: 1.0000000000e+00    Viol.  con: 0e+00    var: 2e-36

Optimizer summary
  Optimizer                 -                         time: 0.07
    Interior-point          - iterations : 15         time: 0.05
      Basis identification  -                         time: 0.02
        Primal              - iterations : 23         time: 0.00
        Dual                - iterations : 9          time: 0.00
        Clean primal        - iterations : 2          time: 0.01
        Clean dual          - iterations : 7          time: 0.00
        Clean primal-dual   - iterations : 0          time: 0.00
    Simplex                 -                         time: 0.00
      Primal simplex        - iterations : 0          time: 0.00
      Dual simplex          - iterations : 0          time: 0.00
      Primal-dual simplex   - iterations : 0          time: 0.00
    Mixed integer           - relaxations: 0          time: 0.00

Open file 'model.sol'
Start writing.
done writing. Time: 0.02

Open file 'model.bas'
Start writing.
done writing. Time: 0.02

Return code - 0  [MSK_RES_OK]
********** Begin .sol file *************
NAME                : SC4cInfeasible
PROBLEM STATUS      : PRIMAL_AND_DUAL_FEASIBLE
SOLUTION STATUS     : OPTIMAL
OBJECTIVE NAME      : OBJ
PRIMAL OBJECTIVE    : 1.00000000e+00
DUAL OBJECTIVE      : 3.79285718e+01
```

CONSTRAINTS

INDEX	NAME	AT	ACTIVITY	LOWER LIMIT	UPPER LIMIT	DUAL LOWER	DUAL UPPER
0	R0000000	EQ	0.00000000000000e+00	0.00000000e+00	0.00000000e+00	0.00000000000000e+00	7.59288004087313e+00
1	R0000001	EQ	0.00000000000000e+00	0.00000000e+00	0.00000000e+00	0.00000000000000e+00	0.00000000000000e+00
2	R0000002	EQ	0.00000000000000e+00	0.00000000e+00	0.00000000e+00	0.00000000000000e+00	0.00000000000000e+00
3	R0000003	SB	-0.00000000000000e+00	0.00000000e+00	0.00000000e+00	0.00000000000000e+00	0.00000000000000e+00
4	R0000004	EQ	0.00000000000000e+00	0.00000000e+00	0.00000000e+00	0.00000000000000e+00	0.00000000000000e+00
5	R0000005	EQ	0.00000000000000e+00	0.00000000e+00	0.00000000e+00	0.00000000000000e+00	0.00000000000000e+00
6	R0000006	EQ	0.00000000000000e+00	0.00000000e+00	0.00000000e+00	0.00000000000000e+00	0.00000000000000e+00
7	R0000007	EQ	0.00000000000000e+00	0.00000000e+00	0.00000000e+00	0.00000000000000e+00	0.00000000000000e+00
8	R0000008	EQ	0.00000000000000e+00	0.00000000e+00	0.00000000e+00	0.00000000000000e+00	0.00000000000000e+00
9	R0000009	EQ	0.00000000000000e+00	0.00000000e+00	0.00000000e+00	0.00000000000000e+00	0.00000000000000e+00
10	R0000010	EQ	0.00000000000000e+00	0.00000000e+00	0.00000000e+00	0.00000000000000e+00	0.00000000000000e+00
11	R0000011	EQ	0.00000000000000e+00	0.00000000e+00	0.00000000e+00	0.00000000000000e+00	0.00000000000000e+00
12	R0000012	EQ	0.00000000000000e+00	0.00000000e+00	0.00000000e+00	0.00000000000000e+00	0.00000000000000e+00
13	R0000013	EQ	0.00000000000000e+00	0.00000000e+00	0.00000000e+00	0.00000000000000e+00	0.00000000000000e+00
14	R0000014	EQ	0.00000000000000e+00	0.00000000e+00	0.00000000e+00	0.00000000000000e+00	0.00000000000000e+00

15	R0000015	EQ 0.00000000000000e+00	0.00000000e+00	0.00000000e+00	0.00000000000000e+00	0.00000000000000e+00
16	R0000016	SB 0.00000000000000e+00	0.00000000e+00	0.00000000e+00	0.00000000000000e+00	0.00000000000000e+00
17	R0000017	EQ 0.00000000000000e+00	0.00000000e+00	0.00000000e+00	0.00000000000000e+00	0.00000000000000e+00
18	R0000018	EQ 0.00000000000000e+00	0.00000000e+00	0.00000000e+00	0.00000000000000e+00	0.00000000000000e+00
19	R0000019	SB -0.00000000000000e+00	0.00000000e+00	0.00000000e+00	0.00000000000000e+00	0.00000000000000e+00
20	R0000020	EQ 0.00000000000000e+00	0.00000000e+00	0.00000000e+00	0.00000000000000e+00	0.00000000000000e+00
21	R0000021	EQ 0.00000000000000e+00	0.00000000e+00	0.00000000e+00	0.00000000000000e+00	0.00000000000000e+00
22	R0000022	EQ 0.00000000000000e+00	0.00000000e+00	0.00000000e+00	0.00000000000000e+00	0.00000000000000e+00
23	R0000023	EQ 0.00000000000000e+00	0.00000000e+00	0.00000000e+00	0.00000000000000e+00	2.14748706463151e+10
24	R0000024	EQ 0.00000000000000e+00	0.00000000e+00	0.00000000e+00	0.00000000000000e+00	2.14748706463151e+10
25	R0000025	EQ 0.00000000000000e+00	0.00000000e+00	0.00000000e+00	0.00000000000000e+00	0.00000000000000e+00
26	R0000026	EQ 0.00000000000000e+00	0.00000000e+00	0.00000000e+00	0.00000000000000e+00	0.00000000000000e+00
27	R0000027	EQ 0.00000000000000e+00	0.00000000e+00	0.00000000e+00	0.00000000000000e+00	0.00000000000000e+00
28	R0000028	EQ 0.00000000000000e+00	0.00000000e+00	0.00000000e+00	0.00000000000000e+00	0.00000000000000e+00
29	R0000029	SB 0.00000000000000e+00	0.00000000e+00	0.00000000e+00	0.00000000000000e+00	0.00000000000000e+00
30	R0000030	EQ 0.00000000000000e+00	0.00000000e+00	0.00000000e+00	0.00000000000000e+00	0.00000000000000e+00
31	R0000031	EQ 0.00000000000000e+00	0.00000000e+00	0.00000000e+00	0.00000000000000e+00	0.00000000000000e+00
32	R0000032	EQ 0.00000000000000e+00	0.00000000e+00	0.00000000e+00	0.00000000000000e+00	0.00000000000000e+00
33	R0000033	EQ 0.00000000000000e+00	0.00000000e+00	0.00000000e+00	0.00000000000000e+00	0.00000000000000e+00
34	R0000034	EQ 0.00000000000000e+00	0.00000000e+00	0.00000000e+00	0.00000000000000e+00	0.00000000000000e+00
35	R0000035	EQ 0.00000000000000e+00	0.00000000e+00	0.00000000e+00	0.00000000000000e+00	1.37878179260560e+03
36	R0000036	EQ 0.00000000000000e+00	0.00000000e+00	0.00000000e+00	0.00000000000000e+00	0.00000000000000e+00
37	R0000037	EQ 0.00000000000000e+00	0.00000000e+00	0.00000000e+00	3.64312880567761e+04	0.00000000000000e+00
38	R0000038	EQ 0.00000000000000e+00	0.00000000e+00	0.00000000e+00	3.64312880567761e+04	0.00000000000000e+00
39	R0000039	EQ 0.00000000000000e+00	0.00000000e+00	0.00000000e+00	3.64312880567761e+04	0.00000000000000e+00
40	R0000040	EQ 0.00000000000000e+00	0.00000000e+00	0.00000000e+00	3.64312880567761e+04	0.00000000000000e+00
41	R0000041	EQ 0.00000000000000e+00	0.00000000e+00	0.00000000e+00	3.64312880567761e+04	0.00000000000000e+00
42	R0000042	SB 0.00000000000000e+00	0.00000000e+00	0.00000000e+00	0.00000000000000e+00	0.00000000000000e+00
43	R0000043	EQ 0.00000000000000e+00	0.00000000e+00	0.00000000e+00	0.00000000000000e+00	0.00000000000000e+00
44	R0000044	EQ 0.00000000000000e+00	0.00000000e+00	0.00000000e+00	0.00000000000000e+00	0.00000000000000e+00
45	R0000045	EQ 0.00000000000000e+00	0.00000000e+00	0.00000000e+00	0.00000000000000e+00	1.37878179260560e+03
46	R0000046	EQ 0.00000000000000e+00	0.00000000e+00	0.00000000e+00	0.00000000000000e+00	0.00000000000000e+00
47	R0000047	EQ 0.00000000000000e+00	0.00000000e+00	0.00000000e+00	0.00000000000000e+00	0.00000000000000e+00
48	R0000048	SB -0.00000000000000e+00	0.00000000e+00	0.00000000e+00	0.00000000000000e+00	0.00000000000000e+00
49	R0000049	EQ 0.00000000000000e+00	0.00000000e+00	0.00000000e+00	0.00000000000000e+00	0.00000000000000e+00
50	R0000050	EQ 0.00000000000000e+00	0.00000000e+00	0.00000000e+00	0.00000000000000e+00	0.00000000000000e+00
51	R0000051	EQ 0.00000000000000e+00	0.00000000e+00	0.00000000e+00	0.00000000000000e+00	0.00000000000000e+00
52	R0000052	EQ 0.00000000000000e+00	0.00000000e+00	0.00000000e+00	0.00000000000000e+00	0.00000000000000e+00
53	R0000053	EQ 0.00000000000000e+00	0.00000000e+00	0.00000000e+00	0.00000000000000e+00	0.00000000000000e+00
54	R0000054	EQ 0.00000000000000e+00	0.00000000e+00	0.00000000e+00	0.00000000000000e+00	0.00000000000000e+00
55	R0000055	EQ 0.00000000000000e+00	0.00000000e+00	0.00000000e+00	0.00000000000000e+00	0.00000000000000e+00
56	R0000056	EQ 0.00000000000000e+00	0.00000000e+00	0.00000000e+00	0.00000000000000e+00	0.00000000000000e+00
57	R0000057	EQ 0.00000000000000e+00	0.00000000e+00	0.00000000e+00	0.00000000000000e+00	0.00000000000000e+00
58	R0000058	EQ 0.00000000000000e+00	0.00000000e+00	0.00000000e+00	0.00000000000000e+00	0.00000000000000e+00
59	R0000059	EQ 0.00000000000000e+00	0.00000000e+00	0.00000000e+00	0.00000000000000e+00	0.00000000000000e+00
60	R0000060	EQ 0.00000000000000e+00	0.00000000e+00	0.00000000e+00	0.00000000000000e+00	0.00000000000000e+00
61	R0000061	EQ 0.00000000000000e+00	0.00000000e+00	0.00000000e+00	0.00000000000000e+00	0.00000000000000e+00
62	R0000062	EQ 0.00000000000000e+00	0.00000000e+00	0.00000000e+00	0.00000000000000e+00	0.00000000000000e+00
63	R0000063	EQ 0.00000000000000e+00	0.00000000e+00	0.00000000e+00	0.00000000000000e+00	0.00000000000000e+00
64	R0000064	SB 0.00000000000000e+00	0.00000000e+00	0.00000000e+00	0.00000000000000e+00	0.00000000000000e+00
65	R0000065	EQ 0.00000000000000e+00	0.00000000e+00	0.00000000e+00	0.00000000000000e+00	0.00000000000000e+00
66	R0000066	EQ 0.00000000000000e+00	0.00000000e+00	0.00000000e+00	0.00000000000000e+00	0.00000000000000e+00
67	R0000067	EQ 0.00000000000000e+00	0.00000000e+00	0.00000000e+00	0.00000000000000e+00	0.00000000000000e+00
68	R0000068	EQ 0.00000000000000e+00	0.00000000e+00	0.00000000e+00	0.00000000000000e+00	0.00000000000000e+00
69	R0000069	EQ 0.00000000000000e+00	0.00000000e+00	0.00000000e+00	0.00000000000000e+00	0.00000000000000e+00
70	R0000070	EQ 0.00000000000000e+00	0.00000000e+00	0.00000000e+00	0.00000000000000e+00	0.00000000000000e+00
71	R0000071	EQ 0.00000000000000e+00	0.00000000e+00	0.00000000e+00	0.00000000000000e+00	0.00000000000000e+00
72	R0000072	EQ 0.00000000000000e+00	0.00000000e+00	0.00000000e+00	0.00000000000000e+00	0.00000000000000e+00
73	R0000073	EQ 0.00000000000000e+00	0.00000000e+00	0.00000000e+00	0.00000000000000e+00	0.00000000000000e+00
74	R0000074	SB 0.00000000000000e+00	0.00000000e+00	0.00000000e+00	0.00000000000000e+00	0.00000000000000e+00
75	R0000075	SB -0.00000000000000e+00	0.00000000e+00	0.00000000e+00	0.00000000000000e+00	0.00000000000000e+00
76	R0000076	EQ 0.00000000000000e+00	0.00000000e+00	0.00000000e+00	0.00000000000000e+00	0.00000000000000e+00
77	R0000077	EQ 0.00000000000000e+00	0.00000000e+00	0.00000000e+00	0.00000000000000e+00	0.00000000000000e+00
78	R0000078	EQ 0.00000000000000e+00	0.00000000e+00	0.00000000e+00	0.00000000000000e+00	0.00000000000000e+00
79	R0000079	EQ 0.00000000000000e+00	0.00000000e+00	0.00000000e+00	0.00000000000000e+00	0.00000000000000e+00
80	R0000080	EQ 0.00000000000000e+00	0.00000000e+00	0.00000000e+00	0.00000000000000e+00	0.00000000000000e+00
81	R0000081	EQ 0.00000000000000e+00	0.00000000e+00	0.00000000e+00	0.00000000000000e+00	0.00000000000000e+00
82	R0000082	EQ 0.00000000000000e+00	0.00000000e+00	0.00000000e+00	0.00000000000000e+00	0.00000000000000e+00
83	R0000083	EQ 0.00000000000000e+00	0.00000000e+00	0.00000000e+00	0.00000000000000e+00	0.00000000000000e+00
84	R0000084	SB -0.00000000000000e+00	0.00000000e+00	0.00000000e+00	0.00000000000000e+00	0.00000000000000e+00
85	R0000085	EQ 0.00000000000000e+00	0.00000000e+00	0.00000000e+00	0.00000000000000e+00	0.00000000000000e+00
86	R0000086	EQ 0.00000000000000e+00	0.00000000e+00	0.00000000e+00	0.00000000000000e+00	0.00000000000000e+00
87	R0000087	SB -0.00000000000000e+00	0.00000000e+00	0.00000000e+00	0.00000000000000e+00	0.00000000000000e+00
88	R0000088	SB -0.00000000000000e+00	0.00000000e+00	0.00000000e+00	0.00000000000000e+00	0.00000000000000e+00
89	R0000089	SB -0.00000000000000e+00	0.00000000e+00	0.00000000e+00	0.00000000000000e+00	0.00000000000000e+00
90	R0000090	SB -0.00000000000000e+00	0.00000000e+00	0.00000000e+00	0.00000000000000e+00	0.00000000000000e+00
91	R0000091	EQ 0.00000000000000e+00	0.00000000e+00	0.00000000e+00	0.00000000000000e+00	0.00000000000000e+00
92	R0000092	EQ 0.00000000000000e+00	0.00000000e+00	0.00000000e+00	0.00000000000000e+00	0.00000000000000e+00
93	R0000093	EQ 0.00000000000000e+00	0.00000000e+00	0.00000000e+00	0.00000000000000e+00	0.00000000000000e+00
94	R0000094	EQ 0.00000000000000e+00	0.00000000e+00	0.00000000e+00	0.00000000000000e+00	0.00000000000000e+00
95	R0000095	EQ 0.00000000000000e+00	0.00000000e+00	0.00000000e+00	0.00000000000000e+00	0.00000000000000e+00
96	R0000096	EQ 0.00000000000000e+00	0.00000000e+00	0.00000000e+00	0.00000000000000e+00	6.48881073248197e+04
97	R0000097	EQ 0.00000000000000e+00	0.00000000e+00	0.00000000e+00	0.00000000000000e+00	0.00000000000000e+00
98	R0000098	EQ 0.00000000000000e+00	0.00000000e+00	0.00000000e+00	0.00000000000000e+00	2.14748706463151e+10
99	R0000099	EQ 0.00000000000000e+00	0.00000000e+00	0.00000000e+00	0.00000000000000e+00	2.14748706463151e+10
100	R0000100	EQ 0.00000000000000e+00	0.00000000e+00	0.00000000e+00	0.00000000000000e+00	2.14748706463151e+10
101	R0000101	EQ 0.00000000000000e+00	0.00000000e+00	0.00000000e+00	0.00000000000000e+00	2.14748706463151e+10
102	R0000102	EQ 0.00000000000000e+00	0.00000000e+00	0.00000000e+00	0.00000000000000e+00	2.14748706463151e+10
103	R0000103	EQ 0.00000000000000e+00	0.00000000e+00	0.00000000e+00	0.00000000000000e+00	2.14748706463151e+10
104	R0000104	EQ 0.00000000000000e+00	0.00000000e+00	0.00000000e+00	0.00000000000000e+00	2.14748706463151e+10
105	R0000105	EQ 0.00000000000000e+00	0.00000000e+00	0.00000000e+00	0.00000000000000e+00	2.14748706463151e+10
106	R0000106	EQ 0.00000000000000e+00	0.00000000e+00	0.00000000e+00	0.00000000000000e+00	2.14748706463151e+10
107	R0000107	EQ 0.00000000000000e+00	0.00000000e+00	0.00000000e+00	0.00000000000000e+00	2.14748706463151e+10
108	R0000108	EQ 0.00000000000000e+00	0.00000000e+00	0.00000000e+00	0.00000000000000e+00	2.14748706463151e+10
109	R0000109	EQ 0.00000000000000e+00	0.00000000e+00	0.00000000e+00	0.00000000000000e+00	2.14748706463151e+10
110	R0000110	EQ 0.00000000000000e+00	0.00000000e+00	0.00000000e+00	0.00000000000000e+00	0.00000000000000e+00
111	R0000111	EQ 0.00000000000000e+00	0.00000000e+00	0.00000000e+00	0.00000000000000e+00	0.00000000000000e+00
112	R0000112	SB -0.00000000000000e+00	0.00000000e+00	0.00000000e+00	0.00000000000000e+00	0.00000000000000e+00

113	R0000113	EQ 0.00000000000000e+00	0.00000000e+00	0.00000000e+00	0.00000000000000e+00	0.00000000000000e+00
114	R0000114	EQ 0.00000000000000e+00	0.00000000e+00	0.00000000e+00	0.00000000000000e+00	0.00000000000000e+00
115	R0000115	EQ 0.00000000000000e+00	0.00000000e+00	0.00000000e+00	0.00000000000000e+00	2.14748706463151e+10
116	R0000116	EQ 0.00000000000000e+00	0.00000000e+00	0.00000000e+00	0.00000000000000e+00	2.14748706463151e+10
117	R0000117	EQ 0.00000000000000e+00	0.00000000e+00	0.00000000e+00	0.00000000000000e+00	2.14748706463151e+10
118	R0000118	EQ 0.00000000000000e+00	0.00000000e+00	0.00000000e+00	0.00000000000000e+00	2.14748706463151e+10
119	R0000119	EQ 0.00000000000000e+00	0.00000000e+00	0.00000000e+00	0.00000000000000e+00	2.14748706463151e+10
120	R0000120	EQ 0.00000000000000e+00	0.00000000e+00	0.00000000e+00	0.00000000000000e+00	2.14748706463151e+10
121	R0000121	EQ 0.00000000000000e+00	0.00000000e+00	0.00000000e+00	0.00000000000000e+00	2.14748706463151e+10
122	R0000122	EQ 0.00000000000000e+00	0.00000000e+00	0.00000000e+00	0.00000000000000e+00	2.14748706463151e+10
123	R0000123	EQ 0.00000000000000e+00	0.00000000e+00	0.00000000e+00	0.00000000000000e+00	2.14748706463151e+10
124	R0000124	EQ 0.00000000000000e+00	0.00000000e+00	0.00000000e+00	0.00000000000000e+00	2.14748706463151e+10
125	R0000125	EQ 0.00000000000000e+00	0.00000000e+00	0.00000000e+00	0.00000000000000e+00	2.14748706463151e+10
126	R0000126	EQ 0.00000000000000e+00	0.00000000e+00	0.00000000e+00	0.00000000000000e+00	2.14748706463151e+10
127	R0000127	EQ 0.00000000000000e+00	0.00000000e+00	0.00000000e+00	0.00000000000000e+00	0.00000000000000e+00
128	R0000128	EQ 0.00000000000000e+00	0.00000000e+00	0.00000000e+00	0.00000000000000e+00	0.00000000000000e+00
129	R0000129	EQ 0.00000000000000e+00	0.00000000e+00	0.00000000e+00	0.00000000000000e+00	0.00000000000000e+00
130	R0000130	EQ 0.00000000000000e+00	0.00000000e+00	0.00000000e+00	0.00000000000000e+00	0.00000000000000e+00
131	R0000131	EQ 0.00000000000000e+00	0.00000000e+00	0.00000000e+00	0.00000000000000e+00	0.00000000000000e+00
132	R0000132	EQ 0.00000000000000e+00	0.00000000e+00	0.00000000e+00	1.42049539903395e-07	0.00000000000000e+00
133	R0000133	EQ 0.00000000000000e+00	0.00000000e+00	0.00000000e+00	1.42049539903395e-07	0.00000000000000e+00
134	R0000134	EQ 0.00000000000000e+00	0.00000000e+00	0.00000000e+00	2.74288547865025e-07	0.00000000000000e+00
135	R0000135	EQ 0.00000000000000e+00	0.00000000e+00	0.00000000e+00	2.74288547865025e-07	0.00000000000000e+00
136	R0000136	EQ 0.00000000000000e+00	0.00000000e+00	0.00000000e+00	0.00000000000000e+00	3.81469726562500e-06
137	R0000137	EQ 0.00000000000000e+00	0.00000000e+00	0.00000000e+00	0.00000000000000e+00	0.00000000000000e+00
138	R0000138	EQ 0.00000000000000e+00	0.00000000e+00	0.00000000e+00	7.10247702464503e-08	0.00000000000000e+00
139	R0000139	EQ 0.00000000000000e+00	0.00000000e+00	0.00000000e+00	7.10247702464503e-08	0.00000000000000e+00
140	R0000140	EQ 0.00000000000000e+00	0.00000000e+00	0.00000000e+00	0.00000000000000e+00	0.00000000000000e+00
141	R0000141	EQ 0.00000000000000e+00	0.00000000e+00	0.00000000e+00	0.00000000000000e+00	0.00000000000000e+00
142	R0000142	EQ 0.00000000000000e+00	0.00000000e+00	0.00000000e+00	1.06537154190665e-07	0.00000000000000e+00
143	R0000143	EQ 0.00000000000000e+00	0.00000000e+00	0.00000000e+00	0.00000000000000e+00	0.00000000000000e+00
144	R0000144	EQ 0.00000000000000e+00	0.00000000e+00	0.00000000e+00	0.00000000000000e+00	3.81469726562500e-06
145	R0000145	EQ 0.00000000000000e+00	0.00000000e+00	0.00000000e+00	0.00000000000000e+00	3.81469726562500e-06
146	R0000146	EQ 0.00000000000000e+00	0.00000000e+00	0.00000000e+00	0.00000000000000e+00	0.00000000000000e+00
147	R0000147	EQ 0.00000000000000e+00	0.00000000e+00	0.00000000e+00	0.00000000000000e+00	0.00000000000000e+00
148	R0000148	EQ 0.00000000000000e+00	0.00000000e+00	0.00000000e+00	0.00000000000000e+00	0.00000000000000e+00
149	R0000149	EQ 0.00000000000000e+00	0.00000000e+00	0.00000000e+00	0.00000000000000e+00	0.00000000000000e+00
150	R0000150	EQ 0.00000000000000e+00	0.00000000e+00	0.00000000e+00	0.00000000000000e+00	0.00000000000000e+00
151	R0000151	EQ 0.00000000000000e+00	0.00000000e+00	0.00000000e+00	0.00000000000000e+00	0.00000000000000e+00
152	R0000152	EQ 0.00000000000000e+00	0.00000000e+00	0.00000000e+00	0.00000000000000e+00	0.00000000000000e+00
153	R0000153	EQ 0.00000000000000e+00	0.00000000e+00	0.00000000e+00	0.00000000000000e+00	0.00000000000000e+00
154	R0000154	EQ 0.00000000000000e+00	0.00000000e+00	0.00000000e+00	0.00000000000000e+00	0.00000000000000e+00
155	R0000155	EQ 0.00000000000000e+00	0.00000000e+00	0.00000000e+00	0.00000000000000e+00	0.00000000000000e+00
156	R0000156	EQ 0.00000000000000e+00	0.00000000e+00	0.00000000e+00	0.00000000000000e+00	0.00000000000000e+00
157	R0000157	EQ 0.00000000000000e+00	0.00000000e+00	0.00000000e+00	0.00000000000000e+00	0.00000000000000e+00
158	R0000158	SB 0.00000000000000e+00	0.00000000e+00	0.00000000e+00	0.00000000000000e+00	0.00000000000000e+00
159	R0000159	SB 0.00000000000000e+00	0.00000000e+00	0.00000000e+00	0.00000000000000e+00	0.00000000000000e+00
160	R0000160	EQ 0.00000000000000e+00	0.00000000e+00	0.00000000e+00	0.00000000000000e+00	0.00000000000000e+00
161	R0000161	EQ 0.00000000000000e+00	0.00000000e+00	0.00000000e+00	0.00000000000000e+00	0.00000000000000e+00
162	R0000162	EQ 0.00000000000000e+00	0.00000000e+00	0.00000000e+00	0.00000000000000e+00	0.00000000000000e+00
163	R0000163	EQ 0.00000000000000e+00	0.00000000e+00	0.00000000e+00	0.00000000000000e+00	0.00000000000000e+00
164	R0000164	EQ 0.00000000000000e+00	0.00000000e+00	0.00000000e+00	0.00000000000000e+00	0.00000000000000e+00
165	R0000165	SB 0.00000000000000e+00	0.00000000e+00	0.00000000e+00	0.00000000000000e+00	0.00000000000000e+00
166	R0000166	EQ 0.00000000000000e+00	0.00000000e+00	0.00000000e+00	0.00000000000000e+00	0.00000000000000e+00
167	R0000167	EQ 0.00000000000000e+00	0.00000000e+00	0.00000000e+00	0.00000000000000e+00	0.00000000000000e+00
168	R0000168	EQ 0.00000000000000e+00	0.00000000e+00	0.00000000e+00	0.00000000000000e+00	0.00000000000000e+00
169	R0000169	EQ 0.00000000000000e+00	0.00000000e+00	0.00000000e+00	0.00000000000000e+00	0.00000000000000e+00
170	R0000170	EQ 0.00000000000000e+00	0.00000000e+00	0.00000000e+00	0.00000000000000e+00	0.00000000000000e+00
171	R0000171	EQ 0.00000000000000e+00	0.00000000e+00	0.00000000e+00	0.00000000000000e+00	0.00000000000000e+00
172	R0000172	EQ 0.00000000000000e+00	0.00000000e+00	0.00000000e+00	0.00000000000000e+00	0.00000000000000e+00
173	R0000173	EQ 0.00000000000000e+00	0.00000000e+00	0.00000000e+00	0.00000000000000e+00	0.00000000000000e+00
174	R0000174	EQ 0.00000000000000e+00	0.00000000e+00	0.00000000e+00	0.00000000000000e+00	0.00000000000000e+00
175	R0000175	EQ 0.00000000000000e+00	0.00000000e+00	0.00000000e+00	0.00000000000000e+00	0.00000000000000e+00
176	R0000176	EQ 0.00000000000000e+00	0.00000000e+00	0.00000000e+00	0.00000000000000e+00	0.00000000000000e+00
177	R0000177	EQ 0.00000000000000e+00	0.00000000e+00	0.00000000e+00	0.00000000000000e+00	0.00000000000000e+00
178	R0000178	EQ 0.00000000000000e+00	0.00000000e+00	0.00000000e+00	0.00000000000000e+00	0.00000000000000e+00
179	R0000179	EQ 0.00000000000000e+00	0.00000000e+00	0.00000000e+00	0.00000000000000e+00	0.00000000000000e+00
180	R0000180	EQ 0.00000000000000e+00	0.00000000e+00	0.00000000e+00	0.00000000000000e+00	0.00000000000000e+00
181	R0000181	EQ 0.00000000000000e+00	0.00000000e+00	0.00000000e+00	0.00000000000000e+00	0.00000000000000e+00
182	R0000182	EQ 0.00000000000000e+00	0.00000000e+00	0.00000000e+00	0.00000000000000e+00	0.00000000000000e+00
183	R0000183	EQ 0.00000000000000e+00	0.00000000e+00	0.00000000e+00	0.00000000000000e+00	0.00000000000000e+00
184	R0000184	SB 0.00000000000000e+00	0.00000000e+00	0.00000000e+00	0.00000000000000e+00	0.00000000000000e+00
185	R0000185	EQ 0.00000000000000e+00	0.00000000e+00	0.00000000e+00	0.00000000000000e+00	0.00000000000000e+00
186	R0000186	EQ 0.00000000000000e+00	0.00000000e+00	0.00000000e+00	0.00000000000000e+00	0.00000000000000e+00
187	R0000187	SB -0.00000000000000e+00	0.00000000e+00	0.00000000e+00	0.00000000000000e+00	0.00000000000000e+00
188	R0000188	EQ 0.00000000000000e+00	0.00000000e+00	0.00000000e+00	0.00000000000000e+00	0.00000000000000e+00
189	R0000189	EQ 0.00000000000000e+00	0.00000000e+00	0.00000000e+00	0.00000000000000e+00	0.00000000000000e+00
190	R0000190	EQ 0.00000000000000e+00	0.00000000e+00	0.00000000e+00	0.00000000000000e+00	0.00000000000000e+00
191	R0000191	EQ 0.00000000000000e+00	0.00000000e+00	0.00000000e+00	0.00000000000000e+00	0.00000000000000e+00
192	R0000192	EQ 0.00000000000000e+00	0.00000000e+00	0.00000000e+00	0.00000000000000e+00	0.00000000000000e+00
193	R0000193	EQ 0.00000000000000e+00	0.00000000e+00	0.00000000e+00	0.00000000000000e+00	0.00000000000000e+00
194	R0000194	EQ 0.00000000000000e+00	0.00000000e+00	0.00000000e+00	0.00000000000000e+00	0.00000000000000e+00
195	R0000195	SB 0.00000000000000e+00	0.00000000e+00	0.00000000e+00	0.00000000000000e+00	0.00000000000000e+00
196	R0000196	EQ 0.00000000000000e+00	0.00000000e+00	0.00000000e+00	0.00000000000000e+00	2.14748706463151e+10
197	R0000197	EQ 0.00000000000000e+00	0.00000000e+00	0.00000000e+00	0.00000000000000e+00	0.00000000000000e+00
198	R0000198	EQ 0.00000000000000e+00	0.00000000e+00	0.00000000e+00	0.00000000000000e+00	0.00000000000000e+00
199	R0000199	EQ 0.00000000000000e+00	0.00000000e+00	0.00000000e+00	0.00000000000000e+00	0.00000000000000e+00
200	R0000200	EQ 0.00000000000000e+00	0.00000000e+00	0.00000000e+00	0.00000000000000e+00	0.00000000000000e+00
201	R0000201	EQ 0.00000000000000e+00	0.00000000e+00	0.00000000e+00	0.00000000000000e+00	0.00000000000000e+00
202	R0000202	EQ 0.00000000000000e+00	0.00000000e+00	0.00000000e+00	0.00000000000000e+00	0.00000000000000e+00
203	R0000203	EQ 0.00000000000000e+00	0.00000000e+00	0.00000000e+00	0.00000000000000e+00	0.00000000000000e+00
204	R0000204	EQ 0.00000000000000e+00	0.00000000e+00	0.00000000e+00	0.00000000000000e+00	0.00000000000000e+00
205	R0000205	EQ 0.00000000000000e+00	0.00000000e+00	0.00000000e+00	0.00000000000000e+00	0.00000000000000e+00
206	R0000206	EQ 0.00000000000000e+00	0.00000000e+00	0.00000000e+00	0.00000000000000e+00	0.00000000000000e+00
207	R0000207	EQ 0.00000000000000e+00	0.00000000e+00	0.00000000e+00	0.00000000000000e+00	0.00000000000000e+00
208	R0000208	EQ 0.00000000000000e+00	0.00000000e+00	0.00000000e+00	3.55123851232251e-08	0.00000000000000e+00
209	R0000209	EQ 0.00000000000000e+00	0.00000000e+00	0.00000000e+00	3.55123851232251e-08	0.00000000000000e+00
210	R0000210	EQ 0.00000000000000e+00	0.00000000e+00	0.00000000e+00	0.00000000000000e+00	2.14748706463151e+10

211	R0000211	EQ	0.00000000000000e+00	0.00000000e+00	0.00000000e+00	0.00000000000000e+00	0.00000000000000e+00
212	R0000212	EQ	0.00000000000000e+00	0.00000000e+00	0.00000000e+00	0.00000000000000e+00	0.00000000000000e+00
213	R0000213	EQ	0.00000000000000e+00	0.00000000e+00	0.00000000e+00	0.00000000000000e+00	2.14748706463151e+10
214	R0000214	EQ	0.00000000000000e+00	0.00000000e+00	0.00000000e+00	0.00000000000000e+00	4.08654947594839e+09
215	R0000215	EQ	0.00000000000000e+00	0.00000000e+00	0.00000000e+00	0.00000000000000e+00	0.00000000000000e+00
216	R0000216	EQ	0.00000000000000e+00	0.00000000e+00	0.00000000e+00	0.00000000000000e+00	2.05963350352547e+05
217	R0000217	EQ	3.81469726562500e-06	0.00000000e+00	0.00000000e+00	0.00000000000000e+00	0.00000000000000e+00
218	R0000218	EQ	0.00000000000000e+00	0.00000000e+00	0.00000000e+00	0.00000000000000e+00	0.00000000000000e+00
219	R0000219	EQ	0.00000000000000e+00	0.00000000e+00	0.00000000e+00	1.63351992019513e+04	0.00000000000000e+00
220	R0000220	EQ	0.00000000000000e+00	0.00000000e+00	0.00000000e+00	0.00000000000000e+00	2.14748491714444e+10
221	R0000221	EQ	0.00000000000000e+00	0.00000000e+00	0.00000000e+00	2.14748706474304e+04	0.00000000000000e+00
222	R0000222	EQ	0.00000000000000e+00	0.00000000e+00	0.00000000e+00	0.00000000000000e+00	0.00000000000000e+00
223	R0000223	EQ	0.00000000000000e+00	0.00000000e+00	0.00000000e+00	0.00000000000000e+00	0.00000000000000e+00
224	R0000224	EQ	0.00000000000000e+00	0.00000000e+00	0.00000000e+00	0.00000000000000e+00	0.00000000000000e+00
225	R0000225	EQ	0.00000000000000e+00	0.00000000e+00	0.00000000e+00	0.00000000000000e+00	0.00000000000000e+00
226	R0000226	EQ	0.00000000000000e+00	0.00000000e+00	0.00000000e+00	0.00000000000000e+00	0.00000000000000e+00
227	R0000227	EQ	0.00000000000000e+00	0.00000000e+00	0.00000000e+00	0.00000000000000e+00	0.00000000000000e+00
228	R0000228	EQ	0.00000000000000e+00	0.00000000e+00	0.00000000e+00	0.00000000000000e+00	0.00000000000000e+00
229	R0000229	EQ	0.00000000000000e+00	0.00000000e+00	0.00000000e+00	0.00000000000000e+00	0.00000000000000e+00
230	R0000230	EQ	0.00000000000000e+00	0.00000000e+00	0.00000000e+00	0.00000000000000e+00	4.08654947594839e+09
231	R0000231	SB	0.00000000000000e+00	0.00000000e+00	0.00000000e+00	0.00000000000000e+00	0.00000000000000e+00
232	R0000232	EQ	0.00000000000000e+00	0.00000000e+00	0.00000000e+00	0.00000000000000e+00	4.08654947594839e+09
233	R0000233	EQ	0.00000000000000e+00	0.00000000e+00	0.00000000e+00	0.00000000000000e+00	0.00000000000000e+00
234	R0000234	EQ	0.00000000000000e+00	0.00000000e+00	0.00000000e+00	0.00000000000000e+00	0.00000000000000e+00
235	R0000235	EQ	0.00000000000000e+00	0.00000000e+00	0.00000000e+00	0.00000000000000e+00	0.00000000000000e+00
236	R0000236	EQ	0.00000000000000e+00	0.00000000e+00	0.00000000e+00	0.00000000000000e+00	0.00000000000000e+00
237	R0000237	EQ	0.00000000000000e+00	0.00000000e+00	0.00000000e+00	0.00000000000000e+00	0.00000000000000e+00
238	R0000238	EQ	0.00000000000000e+00	0.00000000e+00	0.00000000e+00	0.00000000000000e+00	0.00000000000000e+00
239	R0000239	EQ	0.00000000000000e+00	0.00000000e+00	0.00000000e+00	0.00000000000000e+00	0.00000000000000e+00
240	R0000240	EQ	0.00000000000000e+00	0.00000000e+00	0.00000000e+00	0.00000000000000e+00	0.00000000000000e+00
241	R0000241	EQ	0.00000000000000e+00	0.00000000e+00	0.00000000e+00	0.00000000000000e+00	0.00000000000000e+00
242	R0000242	SB	-0.00000000000000e+00	0.00000000e+00	0.00000000e+00	0.00000000000000e+00	0.00000000000000e+00
243	R0000243	SB	-0.00000000000000e+00	0.00000000e+00	0.00000000e+00	0.00000000000000e+00	0.00000000000000e+00
244	R0000244	EQ	0.00000000000000e+00	0.00000000e+00	0.00000000e+00	0.00000000000000e+00	0.00000000000000e+00
245	R0000245	EQ	0.00000000000000e+00	0.00000000e+00	0.00000000e+00	0.00000000000000e+00	0.00000000000000e+00
246	R0000246	EQ	0.00000000000000e+00	0.00000000e+00	0.00000000e+00	0.00000000000000e+00	0.00000000000000e+00
247	R0000247	EQ	0.00000000000000e+00	0.00000000e+00	0.00000000e+00	0.00000000000000e+00	0.00000000000000e+00
248	R0000248	EQ	0.00000000000000e+00	0.00000000e+00	0.00000000e+00	0.00000000000000e+00	0.00000000000000e+00
249	R0000249	EQ	0.00000000000000e+00	0.00000000e+00	0.00000000e+00	0.00000000000000e+00	0.00000000000000e+00
250	R0000250	EQ	0.00000000000000e+00	0.00000000e+00	0.00000000e+00	0.00000000000000e+00	0.00000000000000e+00
251	R0000251	EQ	0.00000000000000e+00	0.00000000e+00	0.00000000e+00	0.00000000000000e+00	0.00000000000000e+00
252	R0000252	EQ	0.00000000000000e+00	0.00000000e+00	0.00000000e+00	0.00000000000000e+00	0.00000000000000e+00
253	R0000253	EQ	0.00000000000000e+00	0.00000000e+00	0.00000000e+00	0.00000000000000e+00	4.08654947594839e+09
254	R0000254	EQ	0.00000000000000e+00	0.00000000e+00	0.00000000e+00	0.00000000000000e+00	0.00000000000000e+00
255	R0000255	EQ	0.00000000000000e+00	0.00000000e+00	0.00000000e+00	0.00000000000000e+00	0.00000000000000e+00
256	R0000256	EQ	0.00000000000000e+00	0.00000000e+00	0.00000000e+00	0.00000000000000e+00	0.00000000000000e+00
257	R0000257	EQ	0.00000000000000e+00	0.00000000e+00	0.00000000e+00	0.00000000000000e+00	0.00000000000000e+00
258	R0000258	EQ	0.00000000000000e+00	0.00000000e+00	0.00000000e+00	0.00000000000000e+00	0.00000000000000e+00
259	R0000259	EQ	0.00000000000000e+00	0.00000000e+00	0.00000000e+00	0.00000000000000e+00	0.00000000000000e+00
260	R0000260	EQ	0.00000000000000e+00	0.00000000e+00	0.00000000e+00	0.00000000000000e+00	0.00000000000000e+00
261	R0000261	EQ	0.00000000000000e+00	0.00000000e+00	0.00000000e+00	0.00000000000000e+00	0.00000000000000e+00
262	R0000262	EQ	0.00000000000000e+00	0.00000000e+00	0.00000000e+00	0.00000000000000e+00	4.08654947594839e+09
263	R0000263	EQ	0.00000000000000e+00	0.00000000e+00	0.00000000e+00	0.00000000000000e+00	0.00000000000000e+00
264	R0000264	EQ	0.00000000000000e+00	0.00000000e+00	0.00000000e+00	0.00000000000000e+00	0.00000000000000e+00
265	R0000265	EQ	0.00000000000000e+00	0.00000000e+00	0.00000000e+00	0.00000000000000e+00	0.00000000000000e+00
266	R0000266	EQ	4.44089209850063e-16	0.00000000e+00	0.00000000e+00	0.00000000000000e+00	0.00000000000000e+00
267	R0000267	EQ	0.00000000000000e+00	0.00000000e+00	0.00000000e+00	0.00000000000000e+00	0.00000000000000e+00
268	R0000268	EQ	0.00000000000000e+00	0.00000000e+00	0.00000000e+00	0.00000000000000e+00	0.00000000000000e+00
269	R0000269	EQ	0.00000000000000e+00	0.00000000e+00	0.00000000e+00	0.00000000000000e+00	0.00000000000000e+00
270	R0000270	EQ	0.00000000000000e+00	0.00000000e+00	0.00000000e+00	0.00000000000000e+00	0.00000000000000e+00
271	R0000271	EQ	0.00000000000000e+00	0.00000000e+00	0.00000000e+00	0.00000000000000e+00	0.00000000000000e+00
272	R0000272	EQ	0.00000000000000e+00	0.00000000e+00	0.00000000e+00	0.00000000000000e+00	0.00000000000000e+00
273	R0000273	EQ	0.00000000000000e+00	0.00000000e+00	0.00000000e+00	0.00000000000000e+00	0.00000000000000e+00
274	R0000274	EQ	0.00000000000000e+00	0.00000000e+00	0.00000000e+00	0.00000000000000e+00	0.00000000000000e+00
275	R0000275	EQ	0.00000000000000e+00	0.00000000e+00	0.00000000e+00	0.00000000000000e+00	0.00000000000000e+00
276	R0000276	EQ	0.00000000000000e+00	0.00000000e+00	0.00000000e+00	0.00000000000000e+00	2.14748706463151e+10
277	R0000277	EQ	0.00000000000000e+00	0.00000000e+00	0.00000000e+00	3.55123851232251e-08	0.00000000000000e+00
278	R0000278	EQ	0.00000000000000e+00	0.00000000e+00	0.00000000e+00	3.55123851232251e-08	0.00000000000000e+00
279	R0000279	EQ	0.00000000000000e+00	0.00000000e+00	0.00000000e+00	0.00000000000000e+00	0.00000000000000e+00
280	R0000280	EQ	0.00000000000000e+00	0.00000000e+00	0.00000000e+00	0.00000000000000e+00	0.00000000000000e+00
281	R0000281	EQ	0.00000000000000e+00	0.00000000e+00	0.00000000e+00	0.00000000000000e+00	0.00000000000000e+00
282	R0000282	EQ	0.00000000000000e+00	0.00000000e+00	0.00000000e+00	0.00000000000000e+00	0.00000000000000e+00
283	R0000283	EQ	0.00000000000000e+00	0.00000000e+00	0.00000000e+00	0.00000000000000e+00	6.48881073265076e+04
284	R0000284	EQ	0.00000000000000e+00	0.00000000e+00	0.00000000e+00	0.00000000000000e+00	0.00000000000000e+00
285	R0000285	EQ	0.00000000000000e+00	0.00000000e+00	0.00000000e+00	0.00000000000000e+00	0.00000000000000e+00
286	R0000286	EQ	0.00000000000000e+00	0.00000000e+00	0.00000000e+00	0.00000000000000e+00	0.00000000000000e+00
287	R0000287	EQ	0.00000000000000e+00	0.00000000e+00	0.00000000e+00	0.00000000000000e+00	0.00000000000000e+00
288	R0000288	EQ	0.00000000000000e+00	0.00000000e+00	0.00000000e+00	0.00000000000000e+00	2.14748706463151e+10
289	R0000289	EQ	0.00000000000000e+00	0.00000000e+00	0.00000000e+00	0.00000000000000e+00	2.14748706463151e+10
290	R0000290	EQ	0.00000000000000e+00	0.00000000e+00	0.00000000e+00	0.00000000000000e+00	0.00000000000000e+00
291	R0000291	EQ	0.00000000000000e+00	0.00000000e+00	0.00000000e+00	0.00000000000000e+00	2.14748706463151e+10
292	R0000292	EQ	0.00000000000000e+00	0.00000000e+00	0.00000000e+00	0.00000000000000e+00	0.00000000000000e+00
293	R0000293	EQ	0.00000000000000e+00	0.00000000e+00	0.00000000e+00	0.00000000000000e+00	0.00000000000000e+00
294	R0000294	EQ	0.00000000000000e+00	0.00000000e+00	0.00000000e+00	0.00000000000000e+00	2.14748706463151e+10
295	R0000295	EQ	0.00000000000000e+00	0.00000000e+00	0.00000000e+00	0.00000000000000e+00	0.00000000000000e+00
296	R0000296	EQ	0.00000000000000e+00	0.00000000e+00	0.00000000e+00	0.00000000000000e+00	0.00000000000000e+00
297	R0000297	SB	0.00000000000000e+00	0.00000000e+00	0.00000000e+00	0.00000000000000e+00	0.00000000000000e+00
298	R0000298	EQ	0.00000000000000e+00	0.00000000e+00	0.00000000e+00	0.00000000000000e+00	0.00000000000000e+00
299	R0000299	EQ	0.00000000000000e+00	0.00000000e+00	0.00000000e+00	0.00000000000000e+00	0.00000000000000e+00
300	R0000300	EQ	0.00000000000000e+00	0.00000000e+00	0.00000000e+00	0.00000000000000e+00	0.00000000000000e+00
301	R0000301	EQ	0.00000000000000e+00	0.00000000e+00	0.00000000e+00	0.00000000000000e+00	0.00000000000000e+00
302	R0000302	EQ	0.00000000000000e+00	0.00000000e+00	0.00000000e+00	0.00000000000000e+00	6.30810074355463e+04
303	R0000303	EQ	0.00000000000000e+00	0.00000000e+00	0.00000000e+00	3.78100698493817e+04	0.00000000000000e+00
304	R0000304	EQ	0.00000000000000e+00	0.00000000e+00	0.00000000e+00	0.00000000000000e+00	0.00000000000000e+00
305	R0000305	EQ	0.00000000000000e+00	0.00000000e+00	0.00000000e+00	0.00000000000000e+00	0.00000000000000e+00
306	R0000306	EQ	0.00000000000000e+00	0.00000000e+00	0.00000000e+00	0.00000000000000e+00	0.00000000000000e+00
307	R0000307	EQ	0.00000000000000e+00	0.00000000e+00	0.00000000e+00	0.00000000000000e+00	0.00000000000000e+00
308	R0000308	EQ	0.00000000000000e+00	0.00000000e+00	0.00000000e+00	0.00000000000000e+00	6.30810074355463e+04

309	R0000309	EQ	0.00000000000000e+00	0.00000000e+00	0.00000000e+00	0.00000000000000e+00	6.30810074355463e+04
310	R0000310	EQ	0.00000000000000e+00	0.00000000e+00	0.00000000e+00	0.00000000000000e+00	0.00000000000000e+00
311	R0000311	EQ	0.00000000000000e+00	0.00000000e+00	0.00000000e+00	0.00000000000000e+00	0.00000000000000e+00
312	R0000312	EQ	0.00000000000000e+00	0.00000000e+00	0.00000000e+00	0.00000000000000e+00	0.00000000000000e+00
313	R0000313	EQ	0.00000000000000e+00	0.00000000e+00	0.00000000e+00	0.00000000000000e+00	0.00000000000000e+00
314	R0000314	EQ	0.00000000000000e+00	0.00000000e+00	0.00000000e+00	0.00000000000000e+00	0.00000000000000e+00
315	R0000315	EQ	1.38777878078145e-17	0.00000000e+00	0.00000000e+00	0.00000000000000e+00	0.00000000000000e+00
316	R0000316	EQ	0.00000000000000e+00	0.00000000e+00	0.00000000e+00	0.00000000000000e+00	0.00000000000000e+00
317	R0000317	EQ	0.00000000000000e+00	0.00000000e+00	0.00000000e+00	0.00000000000000e+00	0.00000000000000e+00
318	R0000318	EQ	0.00000000000000e+00	0.00000000e+00	0.00000000e+00	0.00000000000000e+00	0.00000000000000e+00
319	R0000319	EQ	0.00000000000000e+00	0.00000000e+00	0.00000000e+00	0.00000000000000e+00	0.00000000000000e+00
320	R0000320	EQ	0.00000000000000e+00	0.00000000e+00	0.00000000e+00	0.00000000000000e+00	2.14748706463151e+10
321	R0000321	EQ	0.00000000000000e+00	0.00000000e+00	0.00000000e+00	0.00000000000000e+00	4.08654947594839e+09
322	R0000322	EQ	0.00000000000000e+00	0.00000000e+00	0.00000000e+00	0.00000000000000e+00	0.00000000000000e+00
323	R0000323	EQ	0.00000000000000e+00	0.00000000e+00	0.00000000e+00	0.00000000000000e+00	0.00000000000000e+00
324	R0000324	EQ	0.00000000000000e+00	0.00000000e+00	0.00000000e+00	0.00000000000000e+00	3.78100698493817e+04
325	R0000325	SB	0.00000000000000e+00	0.00000000e+00	0.00000000e+00	0.00000000000000e+00	0.00000000000000e+00
326	R0000326	EQ	0.00000000000000e+00	0.00000000e+00	0.00000000e+00	0.00000000000000e+00	0.00000000000000e+00
327	R0000327	EQ	0.00000000000000e+00	0.00000000e+00	0.00000000e+00	0.00000000000000e+00	0.00000000000000e+00
328	R0000328	EQ	0.00000000000000e+00	0.00000000e+00	0.00000000e+00	0.00000000000000e+00	0.00000000000000e+00
329	R0000329	EQ	0.00000000000000e+00	0.00000000e+00	0.00000000e+00	0.00000000000000e+00	0.00000000000000e+00
330	R0000330	EQ	0.00000000000000e+00	0.00000000e+00	0.00000000e+00	0.00000000000000e+00	0.00000000000000e+00
331	R0000331	EQ	0.00000000000000e+00	0.00000000e+00	0.00000000e+00	0.00000000000000e+00	0.00000000000000e+00
332	R0000332	EQ	0.00000000000000e+00	0.00000000e+00	0.00000000e+00	0.00000000000000e+00	0.00000000000000e+00
333	R0000333	EQ	0.00000000000000e+00	0.00000000e+00	0.00000000e+00	0.00000000000000e+00	0.00000000000000e+00
334	R0000334	EQ	0.00000000000000e+00	0.00000000e+00	0.00000000e+00	0.00000000000000e+00	6.48881073257464e+04
335	R0000335	EQ	0.00000000000000e+00	0.00000000e+00	0.00000000e+00	0.00000000000000e+00	0.00000000000000e+00
336	R0000336	EQ	0.00000000000000e+00	0.00000000e+00	0.00000000e+00	0.00000000000000e+00	0.00000000000000e+00
337	R0000337	EQ	0.00000000000000e+00	0.00000000e+00	0.00000000e+00	0.00000000000000e+00	0.00000000000000e+00
338	R0000338	SB	-0.00000000000000e+00	0.00000000e+00	0.00000000e+00	0.00000000000000e+00	0.00000000000000e+00
339	R0000339	EQ	0.00000000000000e+00	0.00000000e+00	0.00000000e+00	0.00000000000000e+00	0.00000000000000e+00
340	R0000340	EQ	0.00000000000000e+00	0.00000000e+00	0.00000000e+00	0.00000000000000e+00	0.00000000000000e+00
341	R0000341	EQ	0.00000000000000e+00	0.00000000e+00	0.00000000e+00	0.00000000000000e+00	0.00000000000000e+00
342	R0000342	SB	-0.00000000000000e+00	0.00000000e+00	0.00000000e+00	0.00000000000000e+00	0.00000000000000e+00
343	R0000343	EQ	0.00000000000000e+00	0.00000000e+00	0.00000000e+00	0.00000000000000e+00	0.00000000000000e+00
344	R0000344	EQ	0.00000000000000e+00	0.00000000e+00	0.00000000e+00	0.00000000000000e+00	0.00000000000000e+00
345	R0000345	EQ	0.00000000000000e+00	0.00000000e+00	0.00000000e+00	0.00000000000000e+00	0.00000000000000e+00
346	R0000346	EQ	0.00000000000000e+00	0.00000000e+00	0.00000000e+00	0.00000000000000e+00	0.00000000000000e+00
347	R0000347	EQ	0.00000000000000e+00	0.00000000e+00	0.00000000e+00	0.00000000000000e+00	0.00000000000000e+00
348	R0000348	SB	0.00000000000000e+00	0.00000000e+00	0.00000000e+00	0.00000000000000e+00	0.00000000000000e+00
349	R0000349	EQ	0.00000000000000e+00	0.00000000e+00	0.00000000e+00	0.00000000000000e+00	0.00000000000000e+00
350	R0000350	EQ	0.00000000000000e+00	0.00000000e+00	0.00000000e+00	0.00000000000000e+00	0.00000000000000e+00
351	R0000351	EQ	0.00000000000000e+00	0.00000000e+00	0.00000000e+00	0.00000000000000e+00	1.37878179260560e+03
352	R0000352	SB	0.00000000000000e+00	0.00000000e+00	0.00000000e+00	0.00000000000000e+00	0.00000000000000e+00
353	R0000353	EQ	0.00000000000000e+00	0.00000000e+00	0.00000000e+00	0.00000000000000e+00	0.00000000000000e+00
354	R0000354	EQ	0.00000000000000e+00	0.00000000e+00	0.00000000e+00	0.00000000000000e+00	0.00000000000000e+00
355	R0000355	EQ	0.00000000000000e+00	0.00000000e+00	0.00000000e+00	0.00000000000000e+00	0.00000000000000e+00
356	R0000356	EQ	0.00000000000000e+00	0.00000000e+00	0.00000000e+00	0.00000000000000e+00	0.00000000000000e+00
357	R0000357	EQ	0.00000000000000e+00	0.00000000e+00	0.00000000e+00	0.00000000000000e+00	0.00000000000000e+00
358	R0000358	SB	0.00000000000000e+00	0.00000000e+00	0.00000000e+00	0.00000000000000e+00	0.00000000000000e+00
359	R0000359	EQ	0.00000000000000e+00	0.00000000e+00	0.00000000e+00	0.00000000000000e+00	0.00000000000000e+00
360	R0000360	EQ	0.00000000000000e+00	0.00000000e+00	0.00000000e+00	0.00000000000000e+00	0.00000000000000e+00
361	R0000361	SB	-0.00000000000000e+00	0.00000000e+00	0.00000000e+00	0.00000000000000e+00	0.00000000000000e+00
362	R0000362	EQ	0.00000000000000e+00	0.00000000e+00	0.00000000e+00	0.00000000000000e+00	0.00000000000000e+00
363	R0000363	EQ	0.00000000000000e+00	0.00000000e+00	0.00000000e+00	0.00000000000000e+00	0.00000000000000e+00
364	R0000364	EQ	0.00000000000000e+00	0.00000000e+00	0.00000000e+00	0.00000000000000e+00	0.00000000000000e+00
365	R0000365	EQ	0.00000000000000e+00	0.00000000e+00	0.00000000e+00	0.00000000000000e+00	0.00000000000000e+00
366	R0000366	EQ	0.00000000000000e+00	0.00000000e+00	0.00000000e+00	0.00000000000000e+00	0.00000000000000e+00
367	R0000367	EQ	0.00000000000000e+00	0.00000000e+00	0.00000000e+00	0.00000000000000e+00	0.00000000000000e+00
368	R0000368	EQ	0.00000000000000e+00	0.00000000e+00	0.00000000e+00	0.00000000000000e+00	0.00000000000000e+00
369	R0000369	EQ	0.00000000000000e+00	0.00000000e+00	0.00000000e+00	0.00000000000000e+00	0.00000000000000e+00
370	R0000370	EQ	0.00000000000000e+00	0.00000000e+00	0.00000000e+00	0.00000000000000e+00	0.00000000000000e+00
371	R0000371	EQ	0.00000000000000e+00	0.00000000e+00	0.00000000e+00	0.00000000000000e+00	0.00000000000000e+00
372	R0000372	EQ	0.00000000000000e+00	0.00000000e+00	0.00000000e+00	0.00000000000000e+00	0.00000000000000e+00
373	R0000373	EQ	0.00000000000000e+00	0.00000000e+00	0.00000000e+00	0.00000000000000e+00	4.08654947594839e+09
374	R0000374	EQ	0.00000000000000e+00	0.00000000e+00	0.00000000e+00	1.42049539903395e-07	0.00000000000000e+00
375	R0000375	EQ	0.00000000000000e+00	0.00000000e+00	0.00000000e+00	1.42049539903395e-07	0.00000000000000e+00
376	R0000376	EQ	0.00000000000000e+00	0.00000000e+00	0.00000000e+00	1.42049539903395e-07	0.00000000000000e+00
377	R0000377	EQ	0.00000000000000e+00	0.00000000e+00	0.00000000e+00	0.00000000000000e+00	2.14748706463151e+10
378	R0000378	EQ	0.00000000000000e+00	0.00000000e+00	0.00000000e+00	0.00000000000000e+00	0.00000000000000e+00
379	R0000379	EQ	0.00000000000000e+00	0.00000000e+00	0.00000000e+00	0.00000000000000e+00	0.00000000000000e+00
380	R0000380	EQ	0.00000000000000e+00	0.00000000e+00	0.00000000e+00	0.00000000000000e+00	0.00000000000000e+00
381	R0000381	EQ	0.00000000000000e+00	0.00000000e+00	0.00000000e+00	0.00000000000000e+00	0.00000000000000e+00
382	R0000382	EQ	0.00000000000000e+00	0.00000000e+00	0.00000000e+00	0.00000000000000e+00	0.00000000000000e+00
383	R0000383	EQ	0.00000000000000e+00	0.00000000e+00	0.00000000e+00	0.00000000000000e+00	0.00000000000000e+00
384	R0000384	EQ	0.00000000000000e+00	0.00000000e+00	0.00000000e+00	0.00000000000000e+00	0.00000000000000e+00
385	R0000385	EQ	0.00000000000000e+00	0.00000000e+00	0.00000000e+00	0.00000000000000e+00	2.14748706463151e+10
386	R0000386	EQ	0.00000000000000e+00	0.00000000e+00	0.00000000e+00	0.00000000000000e+00	4.08654947594839e+09
387	R0000387	EQ	0.00000000000000e+00	0.00000000e+00	0.00000000e+00	0.00000000000000e+00	0.00000000000000e+00
388	R0000388	EQ	0.00000000000000e+00	0.00000000e+00	0.00000000e+00	0.00000000000000e+00	0.00000000000000e+00
389	R0000389	EQ	0.00000000000000e+00	0.00000000e+00	0.00000000e+00	0.00000000000000e+00	0.00000000000000e+00
390	R0000390	EQ	0.00000000000000e+00	0.00000000e+00	0.00000000e+00	0.00000000000000e+00	0.00000000000000e+00
391	R0000391	EQ	0.00000000000000e+00	0.00000000e+00	0.00000000e+00	0.00000000000000e+00	0.00000000000000e+00
392	R0000392	EQ	0.00000000000000e+00	0.00000000e+00	0.00000000e+00	0.00000000000000e+00	0.00000000000000e+00
393	R0000393	EQ	0.00000000000000e+00	0.00000000e+00	0.00000000e+00	3.78100698493817e+04	0.00000000000000e+00
394	R0000394	EQ	-0.00000000000000e+00	0.00000000e+00	0.00000000e+00	3.78100698493817e+04	0.00000000000000e+00
395	R0000395	EQ	0.00000000000000e+00	0.00000000e+00	0.00000000e+00	0.00000000000000e+00	0.00000000000000e+00
396	R0000396	EQ	0.00000000000000e+00	0.00000000e+00	0.00000000e+00	0.00000000000000e+00	0.00000000000000e+00
397	R0000397	EQ	0.00000000000000e+00	0.00000000e+00	0.00000000e+00	0.00000000000000e+00	0.00000000000000e+00
398	R0000398	EQ	0.00000000000000e+00	0.00000000e+00	0.00000000e+00	0.00000000000000e+00	0.00000000000000e+00
399	R0000399	EQ	0.00000000000000e+00	0.00000000e+00	0.00000000e+00	0.00000000000000e+00	0.00000000000000e+00
400	R0000400	EQ	0.00000000000000e+00	0.00000000e+00	0.00000000e+00	0.00000000000000e+00	0.00000000000000e+00
401	R0000401	EQ	0.00000000000000e+00	0.00000000e+00	0.00000000e+00	0.00000000000000e+00	0.00000000000000e+00
402	R0000402	SB	0.00000000000000e+00	0.00000000e+00	0.00000000e+00	0.00000000000000e+00	0.00000000000000e+00
403	R0000403	EQ	0.00000000000000e+00	0.00000000e+00	0.00000000e+00	0.00000000000000e+00	0.00000000000000e+00
404	R0000404	EQ	0.00000000000000e+00	0.00000000e+00	0.00000000e+00	2.74288547865025e-07	0.00000000000000e+00
405	R0000405	EQ	0.00000000000000e+00	0.00000000e+00	0.00000000e+00	2.74288547865025e-07	0.00000000000000e+00
406	R0000406	EQ	0.00000000000000e+00	0.00000000e+00	0.00000000e+00	2.74288547865025e-07	0.00000000000000e+00

407	R0000407	EQ 0.00000000000000e+00	0.00000000e+00	0.00000000e+00	0.00000000000000e+00	2.14748706463151e+10
408	R0000408	EQ -0.00000000000000e+00	0.00000000e+00	0.00000000e+00	3.79644002043657e+00	0.00000000000000e+00
409	R0000409	EQ 0.00000000000000e+00	0.00000000e+00	0.00000000e+00	0.00000000000000e+00	0.00000000000000e+00
410	R0000410	EQ 0.00000000000000e+00	0.00000000e+00	0.00000000e+00	3.79644002043657e+00	0.00000000000000e+00
411	R0000411	EQ 0.00000000000000e+00	0.00000000e+00	0.00000000e+00	0.00000000000000e+00	0.00000000000000e+00
412	R0000412	EQ 0.00000000000000e+00	0.00000000e+00	0.00000000e+00	0.00000000000000e+00	0.00000000000000e+00
413	R0000413	EQ 0.00000000000000e+00	0.00000000e+00	0.00000000e+00	0.00000000000000e+00	0.00000000000000e+00
414	R0000414	EQ 0.00000000000000e+00	0.00000000e+00	0.00000000e+00	0.00000000000000e+00	0.00000000000000e+00
415	R0000415	EQ 0.00000000000000e+00	0.00000000e+00	0.00000000e+00	0.00000000000000e+00	0.00000000000000e+00
416	R0000416	EQ 0.00000000000000e+00	0.00000000e+00	0.00000000e+00	0.00000000000000e+00	0.00000000000000e+00
417	R0000417	EQ 0.00000000000000e+00	0.00000000e+00	0.00000000e+00	0.00000000000000e+00	0.00000000000000e+00
418	R0000418	EQ 0.00000000000000e+00	0.00000000e+00	0.00000000e+00	0.00000000000000e+00	0.00000000000000e+00
419	R0000419	EQ 0.00000000000000e+00	0.00000000e+00	0.00000000e+00	0.00000000000000e+00	0.00000000000000e+00
420	R0000420	EQ 0.00000000000000e+00	0.00000000e+00	0.00000000e+00	0.00000000000000e+00	0.00000000000000e+00
421	R0000421	EQ 0.00000000000000e+00	0.00000000e+00	0.00000000e+00	0.00000000000000e+00	0.00000000000000e+00
422	R0000422	EQ 0.00000000000000e+00	0.00000000e+00	0.00000000e+00	0.00000000000000e+00	0.00000000000000e+00
423	R0000423	EQ 0.00000000000000e+00	0.00000000e+00	0.00000000e+00	0.00000000000000e+00	0.00000000000000e+00
424	R0000424	EQ 0.00000000000000e+00	0.00000000e+00	0.00000000e+00	0.00000000000000e+00	0.00000000000000e+00
425	R0000425	EQ 0.00000000000000e+00	0.00000000e+00	0.00000000e+00	0.00000000000000e+00	0.00000000000000e+00
426	R0000426	EQ 0.00000000000000e+00	0.00000000e+00	0.00000000e+00	0.00000000000000e+00	0.00000000000000e+00
427	R0000427	EQ 0.00000000000000e+00	0.00000000e+00	0.00000000e+00	0.00000000000000e+00	0.00000000000000e+00
428	R0000428	EQ 0.00000000000000e+00	0.00000000e+00	0.00000000e+00	0.00000000000000e+00	0.00000000000000e+00
429	R0000429	EQ 0.00000000000000e+00	0.00000000e+00	0.00000000e+00	2.14748706474304e+04	0.00000000000000e+00
430	R0000430	EQ 0.00000000000000e+00	0.00000000e+00	0.00000000e+00	0.00000000000000e+00	0.00000000000000e+00
431	R0000431	EQ 0.00000000000000e+00	0.00000000e+00	0.00000000e+00	0.00000000000000e+00	0.00000000000000e+00
432	R0000432	SB -0.00000000000000e+00	0.00000000e+00	0.00000000e+00	0.00000000000000e+00	0.00000000000000e+00
433	R0000433	EQ 0.00000000000000e+00	0.00000000e+00	0.00000000e+00	0.00000000000000e+00	0.00000000000000e+00
434	R0000434	EQ 0.00000000000000e+00	0.00000000e+00	0.00000000e+00	0.00000000000000e+00	0.00000000000000e+00
435	R0000435	EQ 0.00000000000000e+00	0.00000000e+00	0.00000000e+00	0.00000000000000e+00	0.00000000000000e+00
436	R0000436	EQ 0.00000000000000e+00	0.00000000e+00	0.00000000e+00	0.00000000000000e+00	4.08654947594839e+09
437	R0000437	EQ 0.00000000000000e+00	0.00000000e+00	0.00000000e+00	0.00000000000000e+00	4.08654947594839e+09
438	R0000438	EQ 0.00000000000000e+00	0.00000000e+00	0.00000000e+00	0.00000000000000e+00	4.08654947594839e+09
439	R0000439	EQ 0.00000000000000e+00	0.00000000e+00	0.00000000e+00	0.00000000000000e+00	0.00000000000000e+00
440	R0000440	EQ 0.00000000000000e+00	0.00000000e+00	0.00000000e+00	0.00000000000000e+00	0.00000000000000e+00
441	R0000441	EQ 0.00000000000000e+00	0.00000000e+00	0.00000000e+00	0.00000000000000e+00	0.00000000000000e+00
442	R0000442	EQ 0.00000000000000e+00	0.00000000e+00	0.00000000e+00	0.00000000000000e+00	0.00000000000000e+00
443	R0000443	SB -0.00000000000000e+00	0.00000000e+00	0.00000000e+00	0.00000000000000e+00	0.00000000000000e+00
444	R0000444	EQ 0.00000000000000e+00	0.00000000e+00	0.00000000e+00	0.00000000000000e+00	0.00000000000000e+00
445	R0000445	EQ 0.00000000000000e+00	0.00000000e+00	0.00000000e+00	0.00000000000000e+00	0.00000000000000e+00
446	R0000446	EQ 0.00000000000000e+00	0.00000000e+00	0.00000000e+00	0.00000000000000e+00	0.00000000000000e+00
447	R0000447	EQ 0.00000000000000e+00	0.00000000e+00	0.00000000e+00	0.00000000000000e+00	0.00000000000000e+00
448	R0000448	SB -0.00000000000000e+00	0.00000000e+00	0.00000000e+00	0.00000000000000e+00	0.00000000000000e+00
449	R0000449	EQ 0.00000000000000e+00	0.00000000e+00	0.00000000e+00	0.00000000000000e+00	0.00000000000000e+00
450	R0000450	EQ 0.00000000000000e+00	0.00000000e+00	0.00000000e+00	0.00000000000000e+00	0.00000000000000e+00
451	R0000451	EQ 0.00000000000000e+00	0.00000000e+00	0.00000000e+00	0.00000000000000e+00	0.00000000000000e+00
452	R0000452	EQ 0.00000000000000e+00	0.00000000e+00	0.00000000e+00	0.00000000000000e+00	0.00000000000000e+00
453	R0000453	EQ 0.00000000000000e+00	0.00000000e+00	0.00000000e+00	0.00000000000000e+00	0.00000000000000e+00
454	R0000454	EQ 0.00000000000000e+00	0.00000000e+00	0.00000000e+00	0.00000000000000e+00	0.00000000000000e+00
455	R0000455	EQ 0.00000000000000e+00	0.00000000e+00	0.00000000e+00	0.00000000000000e+00	0.00000000000000e+00
456	R0000456	EQ 0.00000000000000e+00	0.00000000e+00	0.00000000e+00	0.00000000000000e+00	0.00000000000000e+00
457	R0000457	EQ 0.00000000000000e+00	0.00000000e+00	0.00000000e+00	0.00000000000000e+00	0.00000000000000e+00
458	R0000458	SB -0.00000000000000e+00	0.00000000e+00	0.00000000e+00	0.00000000000000e+00	0.00000000000000e+00
459	R0000459	EQ 0.00000000000000e+00	0.00000000e+00	0.00000000e+00	0.00000000000000e+00	0.00000000000000e+00
460	R0000460	EQ 0.00000000000000e+00	0.00000000e+00	0.00000000e+00	0.00000000000000e+00	0.00000000000000e+00
461	R0000461	EQ 0.00000000000000e+00	0.00000000e+00	0.00000000e+00	0.00000000000000e+00	0.00000000000000e+00
462	R0000462	EQ 0.00000000000000e+00	0.00000000e+00	0.00000000e+00	0.00000000000000e+00	3.79644002043657e+00
463	R0000463	EQ 0.00000000000000e+00	0.00000000e+00	0.00000000e+00	0.00000000000000e+00	0.00000000000000e+00
464	R0000464	EQ 0.00000000000000e+00	0.00000000e+00	0.00000000e+00	0.00000000000000e+00	0.00000000000000e+00
465	R0000465	EQ 0.00000000000000e+00	0.00000000e+00	0.00000000e+00	0.00000000000000e+00	0.00000000000000e+00
466	R0000466	EQ 0.00000000000000e+00	0.00000000e+00	0.00000000e+00	0.00000000000000e+00	0.00000000000000e+00
467	R0000467	EQ 0.00000000000000e+00	0.00000000e+00	0.00000000e+00	0.00000000000000e+00	0.00000000000000e+00
468	R0000468	EQ 0.00000000000000e+00	0.00000000e+00	0.00000000e+00	0.00000000000000e+00	0.00000000000000e+00
469	R0000469	EQ 0.00000000000000e+00	0.00000000e+00	0.00000000e+00	0.00000000000000e+00	0.00000000000000e+00
470	R0000470	EQ 0.00000000000000e+00	0.00000000e+00	0.00000000e+00	0.00000000000000e+00	0.00000000000000e+00
471	R0000471	EQ 0.00000000000000e+00	0.00000000e+00	0.00000000e+00	0.00000000000000e+00	0.00000000000000e+00
472	R0000472	EQ 0.00000000000000e+00	0.00000000e+00	0.00000000e+00	0.00000000000000e+00	0.00000000000000e+00
473	R0000473	EQ 0.00000000000000e+00	0.00000000e+00	0.00000000e+00	0.00000000000000e+00	0.00000000000000e+00
474	R0000474	EQ 0.00000000000000e+00	0.00000000e+00	0.00000000e+00	0.00000000000000e+00	0.00000000000000e+00
475	R0000475	EQ 0.00000000000000e+00	0.00000000e+00	0.00000000e+00	0.00000000000000e+00	0.00000000000000e+00
476	R0000476	EQ 0.00000000000000e+00	0.00000000e+00	0.00000000e+00	0.00000000000000e+00	0.00000000000000e+00
477	R0000477	EQ 0.00000000000000e+00	0.00000000e+00	0.00000000e+00	0.00000000000000e+00	0.00000000000000e+00
478	R0000478	EQ 0.00000000000000e+00	0.00000000e+00	0.00000000e+00	0.00000000000000e+00	0.00000000000000e+00
479	R0000479	SB -0.00000000000000e+00	0.00000000e+00	0.00000000e+00	0.00000000000000e+00	0.00000000000000e+00
480	R0000480	EQ 0.00000000000000e+00	0.00000000e+00	0.00000000e+00	0.00000000000000e+00	0.00000000000000e+00
481	R0000481	EQ 0.00000000000000e+00	0.00000000e+00	0.00000000e+00	0.00000000000000e+00	0.00000000000000e+00
482	R0000482	EQ 0.00000000000000e+00	0.00000000e+00	0.00000000e+00	0.00000000000000e+00	0.00000000000000e+00
483	R0000483	EQ 0.00000000000000e+00	0.00000000e+00	0.00000000e+00	0.00000000000000e+00	0.00000000000000e+00
484	R0000484	EQ 0.00000000000000e+00	0.00000000e+00	0.00000000e+00	0.00000000000000e+00	0.00000000000000e+00
485	R0000485	EQ 0.00000000000000e+00	0.00000000e+00	0.00000000e+00	0.00000000000000e+00	0.00000000000000e+00
486	R0000486	EQ -0.00000000000000e+00	0.00000000e+00	0.00000000e+00	3.81469726562500e-06	0.00000000000000e+00
487	R0000487	EQ 0.00000000000000e+00	0.00000000e+00	0.00000000e+00	0.00000000000000e+00	0.00000000000000e+00
488	R0000488	EQ 0.00000000000000e+00	0.00000000e+00	0.00000000e+00	0.00000000000000e+00	0.00000000000000e+00
489	R0000489	EQ 0.00000000000000e+00	0.00000000e+00	0.00000000e+00	0.00000000000000e+00	0.00000000000000e+00
490	R0000490	EQ 0.00000000000000e+00	0.00000000e+00	0.00000000e+00	0.00000000000000e+00	0.00000000000000e+00
491	R0000491	EQ 0.00000000000000e+00	0.00000000e+00	0.00000000e+00	0.00000000000000e+00	0.00000000000000e+00
492	R0000492	EQ 0.00000000000000e+00	0.00000000e+00	0.00000000e+00	0.00000000000000e+00	0.00000000000000e+00
493	R0000493	EQ 0.00000000000000e+00	0.00000000e+00	0.00000000e+00	0.00000000000000e+00	0.00000000000000e+00
494	R0000494	EQ 0.00000000000000e+00	0.00000000e+00	0.00000000e+00	0.00000000000000e+00	0.00000000000000e+00
495	R0000495	EQ 0.00000000000000e+00	0.00000000e+00	0.00000000e+00	0.00000000000000e+00	0.00000000000000e+00
496	R0000496	EQ 0.00000000000000e+00	0.00000000e+00	0.00000000e+00	0.00000000000000e+00	0.00000000000000e+00
497	R0000497	EQ 0.00000000000000e+00	0.00000000e+00	0.00000000e+00	0.00000000000000e+00	0.00000000000000e+00
498	R0000498	EQ 0.00000000000000e+00	0.00000000e+00	0.00000000e+00	0.00000000000000e+00	0.00000000000000e+00
499	R0000499	EQ 0.00000000000000e+00	0.00000000e+00	0.00000000e+00	0.00000000000000e+00	0.00000000000000e+00
500	R0000500	EQ -2.45636844198316e-15	0.00000000e+00	0.00000000e+00	0.00000000000000e+00	0.00000000000000e+00
501	R0000501	EQ 0.00000000000000e+00	0.00000000e+00	0.00000000e+00	0.00000000000000e+00	0.00000000000000e+00
502	R0000502	EQ 0.00000000000000e+00	0.00000000e+00	0.00000000e+00	0.00000000000000e+00	0.00000000000000e+00
503	R0000503	SB 0.00000000000000e+00	0.00000000e+00	0.00000000e+00	0.00000000000000e+00	0.00000000000000e+00
504	R0000504	EQ 0.00000000000000e+00	0.00000000e+00	0.00000000e+00	0.00000000000000e+00	0.00000000000000e+00

505	R0000505	EQ 0.00000000000000e+00	0.00000000e+00	0.00000000e+00	0.00000000000000e+00	0.00000000000000e+00
506	R0000506	EQ 0.00000000000000e+00	0.00000000e+00	0.00000000e+00	0.00000000000000e+00	2.14748706463151e+10
507	R0000507	EQ 0.00000000000000e+00	0.00000000e+00	0.00000000e+00	0.00000000000000e+00	0.00000000000000e+00
508	R0000508	EQ 0.00000000000000e+00	0.00000000e+00	0.00000000e+00	0.00000000000000e+00	0.00000000000000e+00
509	R0000509	EQ 0.00000000000000e+00	0.00000000e+00	0.00000000e+00	0.00000000000000e+00	2.14748706463151e+10
510	R0000510	EQ 0.00000000000000e+00	0.00000000e+00	0.00000000e+00	7.10247702464503e-08	0.00000000000000e+00
511	R0000511	EQ 0.00000000000000e+00	0.00000000e+00	0.00000000e+00	7.10247702464503e-08	0.00000000000000e+00
512	R0000512	EQ 0.00000000000000e+00	0.00000000e+00	0.00000000e+00	0.00000000000000e+00	2.14748706463151e+10
513	R0000513	EQ 0.00000000000000e+00	0.00000000e+00	0.00000000e+00	0.00000000000000e+00	0.00000000000000e+00
514	R0000514	EQ 0.00000000000000e+00	0.00000000e+00	0.00000000e+00	0.00000000000000e+00	0.00000000000000e+00
515	R0000515	EQ 0.00000000000000e+00	0.00000000e+00	0.00000000e+00	0.00000000000000e+00	0.00000000000000e+00
516	R0000516	EQ 0.00000000000000e+00	0.00000000e+00	0.00000000e+00	0.00000000000000e+00	0.00000000000000e+00
517	R0000517	EQ 0.00000000000000e+00	0.00000000e+00	0.00000000e+00	0.00000000000000e+00	0.00000000000000e+00
518	R0000518	EQ 0.00000000000000e+00	0.00000000e+00	0.00000000e+00	0.00000000000000e+00	0.00000000000000e+00
519	R0000519	EQ 0.00000000000000e+00	0.00000000e+00	0.00000000e+00	0.00000000000000e+00	0.00000000000000e+00
520	R0000520	EQ 0.00000000000000e+00	0.00000000e+00	0.00000000e+00	0.00000000000000e+00	0.00000000000000e+00
521	R0000521	EQ 0.00000000000000e+00	0.00000000e+00	0.00000000e+00	0.00000000000000e+00	0.00000000000000e+00
522	R0000522	EQ 0.00000000000000e+00	0.00000000e+00	0.00000000e+00	0.00000000000000e+00	0.00000000000000e+00
523	R0000523	EQ 0.00000000000000e+00	0.00000000e+00	0.00000000e+00	0.00000000000000e+00	0.00000000000000e+00
524	R0000524	EQ 0.00000000000000e+00	0.00000000e+00	0.00000000e+00	0.00000000000000e+00	0.00000000000000e+00
525	R0000525	EQ 0.00000000000000e+00	0.00000000e+00	0.00000000e+00	0.00000000000000e+00	0.00000000000000e+00
526	R0000526	SB -0.00000000000000e+00	0.00000000e+00	0.00000000e+00	0.00000000000000e+00	0.00000000000000e+00
527	R0000527	EQ 0.00000000000000e+00	0.00000000e+00	0.00000000e+00	0.00000000000000e+00	0.00000000000000e+00
528	R0000528	EQ 0.00000000000000e+00	0.00000000e+00	0.00000000e+00	0.00000000000000e+00	0.00000000000000e+00
529	R0000529	EQ 0.00000000000000e+00	0.00000000e+00	0.00000000e+00	0.00000000000000e+00	0.00000000000000e+00
530	R0000530	EQ 0.00000000000000e+00	0.00000000e+00	0.00000000e+00	0.00000000000000e+00	0.00000000000000e+00
531	R0000531	EQ 0.00000000000000e+00	0.00000000e+00	0.00000000e+00	7.97067234778545e+03	0.00000000000000e+00
532	R0000532	EQ 0.00000000000000e+00	0.00000000e+00	0.00000000e+00	7.97067234871211e+03	0.00000000000000e+00
533	R0000533	EQ 0.00000000000000e+00	0.00000000e+00	0.00000000e+00	0.00000000000000e+00	2.84568192689702e+04
534	R0000534	EQ 0.00000000000000e+00	0.00000000e+00	0.00000000e+00	0.00000000000000e+00	2.84568192680436e+04
535	R0000535	EQ 0.00000000000000e+00	0.00000000e+00	0.00000000e+00	0.00000000000000e+00	0.00000000000000e+00
536	R0000536	EQ 0.00000000000000e+00	0.00000000e+00	0.00000000e+00	0.00000000000000e+00	0.00000000000000e+00
537	R0000537	EQ 0.00000000000000e+00	0.00000000e+00	0.00000000e+00	0.00000000000000e+00	0.00000000000000e+00
538	R0000538	EQ 0.00000000000000e+00	0.00000000e+00	0.00000000e+00	0.00000000000000e+00	0.00000000000000e+00
539	R0000539	EQ 0.00000000000000e+00	0.00000000e+00	0.00000000e+00	0.00000000000000e+00	0.00000000000000e+00
540	R0000540	EQ 0.00000000000000e+00	0.00000000e+00	0.00000000e+00	0.00000000000000e+00	1.37878179260560e+03
541	R0000541	EQ 0.00000000000000e+00	0.00000000e+00	0.00000000e+00	0.00000000000000e+00	0.00000000000000e+00
542	R0000542	EQ 0.00000000000000e+00	0.00000000e+00	0.00000000e+00	0.00000000000000e+00	0.00000000000000e+00
543	R0000543	EQ 0.00000000000000e+00	0.00000000e+00	0.00000000e+00	0.00000000000000e+00	0.00000000000000e+00
544	R0000544	EQ 0.00000000000000e+00	0.00000000e+00	0.00000000e+00	4.08654947594839e+09	0.00000000000000e+00
545	R0000545	EQ 0.00000000000000e+00	0.00000000e+00	0.00000000e+00	0.00000000000000e+00	0.00000000000000e+00
546	R0000546	EQ 0.00000000000000e+00	0.00000000e+00	0.00000000e+00	0.00000000000000e+00	0.00000000000000e+00
547	R0000547	EQ 0.00000000000000e+00	0.00000000e+00	0.00000000e+00	0.00000000000000e+00	0.00000000000000e+00
548	R0000548	EQ 0.00000000000000e+00	0.00000000e+00	0.00000000e+00	0.00000000000000e+00	0.00000000000000e+00
549	R0000549	EQ 0.00000000000000e+00	0.00000000e+00	0.00000000e+00	0.00000000000000e+00	0.00000000000000e+00
550	R0000550	EQ 0.00000000000000e+00	0.00000000e+00	0.00000000e+00	0.00000000000000e+00	0.00000000000000e+00
551	R0000551	EQ 0.00000000000000e+00	0.00000000e+00	0.00000000e+00	0.00000000000000e+00	0.00000000000000e+00
552	R0000552	EQ 0.00000000000000e+00	0.00000000e+00	0.00000000e+00	0.00000000000000e+00	0.00000000000000e+00
553	R0000553	EQ 0.00000000000000e+00	0.00000000e+00	0.00000000e+00	0.00000000000000e+00	0.00000000000000e+00
554	R0000554	EQ 0.00000000000000e+00	0.00000000e+00	0.00000000e+00	0.00000000000000e+00	0.00000000000000e+00
555	R0000555	SB 0.00000000000000e+00	0.00000000e+00	0.00000000e+00	0.00000000000000e+00	0.00000000000000e+00
556	R0000556	EQ 0.00000000000000e+00	0.00000000e+00	0.00000000e+00	0.00000000000000e+00	0.00000000000000e+00
557	R0000557	EQ 0.00000000000000e+00	0.00000000e+00	0.00000000e+00	0.00000000000000e+00	0.00000000000000e+00
558	R0000558	EQ 0.00000000000000e+00	0.00000000e+00	0.00000000e+00	0.00000000000000e+00	0.00000000000000e+00
559	R0000559	SB 0.00000000000000e+00	0.00000000e+00	0.00000000e+00	0.00000000000000e+00	0.00000000000000e+00
560	R0000560	SB 0.00000000000000e+00	0.00000000e+00	0.00000000e+00	0.00000000000000e+00	0.00000000000000e+00
561	R0000561	EQ 0.00000000000000e+00	0.00000000e+00	0.00000000e+00	0.00000000000000e+00	0.00000000000000e+00
562	R0000562	EQ 0.00000000000000e+00	0.00000000e+00	0.00000000e+00	0.00000000000000e+00	0.00000000000000e+00
563	R0000563	EQ 0.00000000000000e+00	0.00000000e+00	0.00000000e+00	0.00000000000000e+00	0.00000000000000e+00
564	R0000564	SB 0.00000000000000e+00	0.00000000e+00	0.00000000e+00	0.00000000000000e+00	0.00000000000000e+00
565	R0000565	SB 0.00000000000000e+00	0.00000000e+00	0.00000000e+00	0.00000000000000e+00	0.00000000000000e+00
566	R0000566	EQ 0.00000000000000e+00	0.00000000e+00	0.00000000e+00	0.00000000000000e+00	0.00000000000000e+00
567	R0000567	EQ 0.00000000000000e+00	0.00000000e+00	0.00000000e+00	0.00000000000000e+00	0.00000000000000e+00
568	R0000568	EQ 0.00000000000000e+00	0.00000000e+00	0.00000000e+00	0.00000000000000e+00	0.00000000000000e+00
569	R0000569	EQ -0.00000000000000e+00	0.00000000e+00	0.00000000e+00	4.08654947594839e+09	0.00000000000000e+00
570	R0000570	SB 0.00000000000000e+00	0.00000000e+00	0.00000000e+00	0.00000000000000e+00	0.00000000000000e+00
571	R0000571	EQ 0.00000000000000e+00	0.00000000e+00	0.00000000e+00	0.00000000000000e+00	0.00000000000000e+00
572	R0000572	EQ 0.00000000000000e+00	0.00000000e+00	0.00000000e+00	0.00000000000000e+00	0.00000000000000e+00
573	R0000573	SB 0.00000000000000e+00	0.00000000e+00	0.00000000e+00	0.00000000000000e+00	0.00000000000000e+00
574	R0000574	EQ 0.00000000000000e+00	0.00000000e+00	0.00000000e+00	0.00000000000000e+00	0.00000000000000e+00
575	R0000575	EQ 0.00000000000000e+00	0.00000000e+00	0.00000000e+00	0.00000000000000e+00	0.00000000000000e+00
576	R0000576	EQ 0.00000000000000e+00	0.00000000e+00	0.00000000e+00	0.00000000000000e+00	0.00000000000000e+00
577	R0000577	EQ 0.00000000000000e+00	0.00000000e+00	0.00000000e+00	0.00000000000000e+00	0.00000000000000e+00
578	R0000578	SB 0.00000000000000e+00	0.00000000e+00	0.00000000e+00	0.00000000000000e+00	0.00000000000000e+00
579	R0000579	SB 0.00000000000000e+00	0.00000000e+00	0.00000000e+00	0.00000000000000e+00	0.00000000000000e+00
580	R0000580	SB 0.00000000000000e+00	0.00000000e+00	0.00000000e+00	0.00000000000000e+00	0.00000000000000e+00
581	R0000581	EQ 0.00000000000000e+00	0.00000000e+00	0.00000000e+00	0.00000000000000e+00	0.00000000000000e+00
582	R0000582	EQ 0.00000000000000e+00	0.00000000e+00	0.00000000e+00	0.00000000000000e+00	0.00000000000000e+00
583	R0000583	EQ 0.00000000000000e+00	0.00000000e+00	0.00000000e+00	0.00000000000000e+00	0.00000000000000e+00
584	R0000584	EQ 0.00000000000000e+00	0.00000000e+00	0.00000000e+00	0.00000000000000e+00	0.00000000000000e+00
585	R0000585	SB 0.00000000000000e+00	0.00000000e+00	0.00000000e+00	0.00000000000000e+00	0.00000000000000e+00
586	R0000586	EQ 0.00000000000000e+00	0.00000000e+00	0.00000000e+00	0.00000000000000e+00	0.00000000000000e+00
587	R0000587	EQ 0.00000000000000e+00	0.00000000e+00	0.00000000e+00	0.00000000000000e+00	0.00000000000000e+00
588	R0000588	EQ 0.00000000000000e+00	0.00000000e+00	0.00000000e+00	0.00000000000000e+00	0.00000000000000e+00
589	R0000589	SB 0.00000000000000e+00	0.00000000e+00	0.00000000e+00	0.00000000000000e+00	0.00000000000000e+00
590	R0000590	SB 0.00000000000000e+00	0.00000000e+00	0.00000000e+00	0.00000000000000e+00	0.00000000000000e+00
591	R0000591	EQ 0.00000000000000e+00	0.00000000e+00	0.00000000e+00	0.00000000000000e+00	0.00000000000000e+00
592	R0000592	EQ 0.00000000000000e+00	0.00000000e+00	0.00000000e+00	0.00000000000000e+00	0.00000000000000e+00
593	R0000593	EQ 0.00000000000000e+00	0.00000000e+00	0.00000000e+00	0.00000000000000e+00	0.00000000000000e+00
594	R0000594	SB 0.00000000000000e+00	0.00000000e+00	0.00000000e+00	0.00000000000000e+00	0.00000000000000e+00
595	R0000595	SB 0.00000000000000e+00	0.00000000e+00	0.00000000e+00	0.00000000000000e+00	0.00000000000000e+00
596	R0000596	EQ 0.00000000000000e+00	0.00000000e+00	0.00000000e+00	0.00000000000000e+00	0.00000000000000e+00
597	R0000597	SB -0.00000000000000e+00	0.00000000e+00	0.00000000e+00	0.00000000000000e+00	0.00000000000000e+00
598	R0000598	SB 0.00000000000000e+00	0.00000000e+00	0.00000000e+00	0.00000000000000e+00	0.00000000000000e+00
599	R0000599	EQ 0.00000000000000e+00	0.00000000e+00	0.00000000e+00	0.00000000000000e+00	0.00000000000000e+00
600	R0000600	EQ 0.00000000000000e+00	0.00000000e+00	0.00000000e+00	0.00000000000000e+00	0.00000000000000e+00
601	R0000601	SB 0.00000000000000e+00	0.00000000e+00	0.00000000e+00	0.00000000000000e+00	0.00000000000000e+00
602	R0000602	EQ 0.00000000000000e+00	0.00000000e+00	0.00000000e+00	0.00000000000000e+00	0.00000000000000e+00

603	R0000603	EQ	0.00000000000000e+00	0.00000000e+00	0.00000000e+00	0.00000000000000e+00	0.00000000000000e+00
604	R0000604	EQ	0.00000000000000e+00	0.00000000e+00	0.00000000e+00	0.00000000000000e+00	0.00000000000000e+00
605	R0000605	EQ	0.00000000000000e+00	0.00000000e+00	0.00000000e+00	0.00000000000000e+00	0.00000000000000e+00
606	R0000606	EQ	0.00000000000000e+00	0.00000000e+00	0.00000000e+00	0.00000000000000e+00	4.08654947594839e+09
607	R0000607	SB	0.00000000000000e+00	0.00000000e+00	0.00000000e+00	0.00000000000000e+00	0.00000000000000e+00
608	R0000608	EQ	0.00000000000000e+00	0.00000000e+00	0.00000000e+00	0.00000000000000e+00	0.00000000000000e+00
609	R0000609	EQ	0.00000000000000e+00	0.00000000e+00	0.00000000e+00	0.00000000000000e+00	0.00000000000000e+00
610	R0000610	SB	0.00000000000000e+00	0.00000000e+00	0.00000000e+00	0.00000000000000e+00	0.00000000000000e+00
611	R0000611	SB	0.00000000000000e+00	0.00000000e+00	0.00000000e+00	0.00000000000000e+00	0.00000000000000e+00
612	R0000612	EQ	0.00000000000000e+00	0.00000000e+00	0.00000000e+00	0.00000000000000e+00	0.00000000000000e+00
613	R0000613	EQ	0.00000000000000e+00	0.00000000e+00	0.00000000e+00	0.00000000000000e+00	0.00000000000000e+00
614	R0000614	EQ	0.00000000000000e+00	0.00000000e+00	0.00000000e+00	0.00000000000000e+00	0.00000000000000e+00
615	R0000615	EQ	0.00000000000000e+00	0.00000000e+00	0.00000000e+00	0.00000000000000e+00	0.00000000000000e+00
616	R0000616	EQ	0.00000000000000e+00	0.00000000e+00	0.00000000e+00	0.00000000000000e+00	0.00000000000000e+00
617	R0000617	SB	0.00000000000000e+00	0.00000000e+00	0.00000000e+00	0.00000000000000e+00	0.00000000000000e+00
618	R0000618	SB	0.00000000000000e+00	0.00000000e+00	0.00000000e+00	0.00000000000000e+00	0.00000000000000e+00
619	R0000619	EQ	0.00000000000000e+00	0.00000000e+00	0.00000000e+00	0.00000000000000e+00	0.00000000000000e+00
620	R0000620	EQ	0.00000000000000e+00	0.00000000e+00	0.00000000e+00	0.00000000000000e+00	0.00000000000000e+00
621	R0000621	SB	0.00000000000000e+00	0.00000000e+00	0.00000000e+00	0.00000000000000e+00	0.00000000000000e+00
622	R0000622	SB	0.00000000000000e+00	0.00000000e+00	0.00000000e+00	0.00000000000000e+00	0.00000000000000e+00
623	R0000623	EQ	0.00000000000000e+00	0.00000000e+00	0.00000000e+00	0.00000000000000e+00	0.00000000000000e+00
624	R0000624	SB	0.00000000000000e+00	0.00000000e+00	0.00000000e+00	0.00000000000000e+00	0.00000000000000e+00
625	R0000625	SB	0.00000000000000e+00	0.00000000e+00	0.00000000e+00	0.00000000000000e+00	0.00000000000000e+00
626	R0000626	EQ	0.00000000000000e+00	0.00000000e+00	0.00000000e+00	0.00000000000000e+00	0.00000000000000e+00
627	R0000627	SB	0.00000000000000e+00	0.00000000e+00	0.00000000e+00	0.00000000000000e+00	0.00000000000000e+00
628	R0000628	EQ	0.00000000000000e+00	0.00000000e+00	0.00000000e+00	0.00000000000000e+00	0.00000000000000e+00
629	R0000629	EQ	0.00000000000000e+00	0.00000000e+00	0.00000000e+00	0.00000000000000e+00	0.00000000000000e+00
630	R0000630	EQ	0.00000000000000e+00	0.00000000e+00	0.00000000e+00	0.00000000000000e+00	0.00000000000000e+00
631	R0000631	EQ	0.00000000000000e+00	0.00000000e+00	0.00000000e+00	0.00000000000000e+00	0.00000000000000e+00
632	R0000632	EQ	0.00000000000000e+00	0.00000000e+00	0.00000000e+00	4.00454110675406e+01	0.00000000000000e+00
633	R0000633	EQ	0.00000000000000e+00	0.00000000e+00	0.00000000e+00	0.00000000000000e+00	0.00000000000000e+00
634	R0000634	EQ	0.00000000000000e+00	0.00000000e+00	0.00000000e+00	0.00000000000000e+00	2.14748706463151e+10
635	R0000635	EQ	0.00000000000000e+00	0.00000000e+00	0.00000000e+00	0.00000000000000e+00	2.05963350352547e+05
636	R0000636	EQ	0.00000000000000e+00	0.00000000e+00	0.00000000e+00	0.00000000000000e+00	0.00000000000000e+00
637	R0000637	EQ	0.00000000000000e+00	0.00000000e+00	0.00000000e+00	0.00000000000000e+00	0.00000000000000e+00
638	R0000638	EQ	0.00000000000000e+00	0.00000000e+00	0.00000000e+00	0.00000000000000e+00	0.00000000000000e+00
639	R0000639	EQ	0.00000000000000e+00	0.00000000e+00	0.00000000e+00	0.00000000000000e+00	0.00000000000000e+00
640	R0000640	EQ	0.00000000000000e+00	0.00000000e+00	0.00000000e+00	0.00000000000000e+00	0.00000000000000e+00
641	R0000641	EQ	0.00000000000000e+00	0.00000000e+00	0.00000000e+00	0.00000000000000e+00	0.00000000000000e+00
642	R0000642	EQ	0.00000000000000e+00	0.00000000e+00	0.00000000e+00	4.13520207058176e-08	0.00000000000000e+00
643	R0000643	EQ	0.00000000000000e+00	0.00000000e+00	0.00000000e+00	0.00000000000000e+00	2.14748706463151e+10
644	R0000644	EQ	0.00000000000000e+00	0.00000000e+00	0.00000000e+00	0.00000000000000e+00	4.08654947594839e+09
645	R0000645	EQ	0.00000000000000e+00	0.00000000e+00	0.00000000e+00	0.00000000000000e+00	0.00000000000000e+00
646	R0000646	EQ	0.00000000000000e+00	0.00000000e+00	0.00000000e+00	0.00000000000000e+00	0.00000000000000e+00
647	R0000647	EQ	0.00000000000000e+00	0.00000000e+00	0.00000000e+00	0.00000000000000e+00	3.79644002043657e+00
648	R0000648	EQ	0.00000000000000e+00	0.00000000e+00	0.00000000e+00	0.00000000000000e+00	2.84606157089907e+04
649	R0000649	EQ	0.00000000000000e+00	0.00000000e+00	0.00000000e+00	0.00000000000000e+00	2.84606157080640e+04
650	R0000650	EQ	0.00000000000000e+00	0.00000000e+00	0.00000000e+00	0.00000000000000e+00	0.00000000000000e+00
651	R0000651	EQ	0.00000000000000e+00	0.00000000e+00	0.00000000e+00	0.00000000000000e+00	0.00000000000000e+00
652	R0000652	EQ	0.00000000000000e+00	0.00000000e+00	0.00000000e+00	0.00000000000000e+00	0.00000000000000e+00
653	R0000653	EQ	0.00000000000000e+00	0.00000000e+00	0.00000000e+00	0.00000000000000e+00	0.00000000000000e+00
654	R0000654	EQ	0.00000000000000e+00	0.00000000e+00	0.00000000e+00	0.00000000000000e+00	0.00000000000000e+00
655	R0000655	EQ	0.00000000000000e+00	0.00000000e+00	0.00000000e+00	0.00000000000000e+00	0.00000000000000e+00
656	R0000656	SB	-0.00000000000000e+00	0.00000000e+00	0.00000000e+00	0.00000000000000e+00	0.00000000000000e+00
657	R0000657	EQ	0.00000000000000e+00	0.00000000e+00	0.00000000e+00	0.00000000000000e+00	0.00000000000000e+00
658	R0000658	EQ	0.00000000000000e+00	0.00000000e+00	0.00000000e+00	0.00000000000000e+00	0.00000000000000e+00
659	R0000659	EQ	0.00000000000000e+00	0.00000000e+00	0.00000000e+00	0.00000000000000e+00	0.00000000000000e+00
660	R0000660	EQ	0.00000000000000e+00	0.00000000e+00	0.00000000e+00	0.00000000000000e+00	0.00000000000000e+00
661	R0000661	EQ	0.00000000000000e+00	0.00000000e+00	0.00000000e+00	0.00000000000000e+00	0.00000000000000e+00
662	R0000662	EQ	0.00000000000000e+00	0.00000000e+00	0.00000000e+00	0.00000000000000e+00	0.00000000000000e+00
663	R0000663	EQ	0.00000000000000e+00	0.00000000e+00	0.00000000e+00	0.00000000000000e+00	0.00000000000000e+00
664	R0000664	EQ	0.00000000000000e+00	0.00000000e+00	0.00000000e+00	0.00000000000000e+00	0.00000000000000e+00
665	R0000665	EQ	0.00000000000000e+00	0.00000000e+00	0.00000000e+00	0.00000000000000e+00	1.37878179260560e+03
666	R0000666	SB	0.00000000000000e+00	0.00000000e+00	0.00000000e+00	0.00000000000000e+00	0.00000000000000e+00
667	R0000667	EQ	0.00000000000000e+00	0.00000000e+00	0.00000000e+00	0.00000000000000e+00	0.00000000000000e+00
668	R0000668	EQ	0.00000000000000e+00	0.00000000e+00	0.00000000e+00	0.00000000000000e+00	0.00000000000000e+00
669	R0000669	EQ	0.00000000000000e+00	0.00000000e+00	0.00000000e+00	0.00000000000000e+00	0.00000000000000e+00
670	R0000670	EQ	0.00000000000000e+00	0.00000000e+00	0.00000000e+00	0.00000000000000e+00	0.00000000000000e+00
671	R0000671	EQ	0.00000000000000e+00	0.00000000e+00	0.00000000e+00	0.00000000000000e+00	0.00000000000000e+00
672	R0000672	EQ	0.00000000000000e+00	0.00000000e+00	0.00000000e+00	0.00000000000000e+00	0.00000000000000e+00
673	R0000673	EQ	0.00000000000000e+00	0.00000000e+00	0.00000000e+00	0.00000000000000e+00	0.00000000000000e+00
674	R0000674	SB	0.00000000000000e+00	0.00000000e+00	0.00000000e+00	0.00000000000000e+00	0.00000000000000e+00
675	R0000675	EQ	0.00000000000000e+00	0.00000000e+00	0.00000000e+00	0.00000000000000e+00	0.00000000000000e+00
676	R0000676	EQ	0.00000000000000e+00	0.00000000e+00	0.00000000e+00	0.00000000000000e+00	0.00000000000000e+00
677	R0000677	SB	0.00000000000000e+00	0.00000000e+00	0.00000000e+00	0.00000000000000e+00	0.00000000000000e+00
678	R0000678	EQ	0.00000000000000e+00	0.00000000e+00	0.00000000e+00	0.00000000000000e+00	0.00000000000000e+00
679	R0000679	EQ	0.00000000000000e+00	0.00000000e+00	0.00000000e+00	0.00000000000000e+00	0.00000000000000e+00
680	R0000680	EQ	0.00000000000000e+00	0.00000000e+00	0.00000000e+00	0.00000000000000e+00	0.00000000000000e+00
681	R0000681	EQ	0.00000000000000e+00	0.00000000e+00	0.00000000e+00	0.00000000000000e+00	0.00000000000000e+00
682	R0000682	EQ	0.00000000000000e+00	0.00000000e+00	0.00000000e+00	0.00000000000000e+00	0.00000000000000e+00
683	R0000683	EQ	0.00000000000000e+00	0.00000000e+00	0.00000000e+00	0.00000000000000e+00	0.00000000000000e+00
684	R0000684	EQ	0.00000000000000e+00	0.00000000e+00	0.00000000e+00	0.00000000000000e+00	0.00000000000000e+00
685	R0000685	EQ	0.00000000000000e+00	0.00000000e+00	0.00000000e+00	0.00000000000000e+00	0.00000000000000e+00
686	R0000686	EQ	0.00000000000000e+00	0.00000000e+00	0.00000000e+00	0.00000000000000e+00	0.00000000000000e+00
687	R0000687	EQ	0.00000000000000e+00	0.00000000e+00	0.00000000e+00	0.00000000000000e+00	4.08654947594839e+09
688	R0000688	EQ	0.00000000000000e+00	0.00000000e+00	0.00000000e+00	0.00000000000000e+00	0.00000000000000e+00
689	R0000689	EQ	0.00000000000000e+00	0.00000000e+00	0.00000000e+00	0.00000000000000e+00	4.08654947594839e+09
690	R0000690	EQ	0.00000000000000e+00	0.00000000e+00	0.00000000e+00	0.00000000000000e+00	0.00000000000000e+00
691	R0000691	EQ	0.00000000000000e+00	0.00000000e+00	0.00000000e+00	0.00000000000000e+00	4.08654947594839e+09
692	R0000692	EQ	0.00000000000000e+00	0.00000000e+00	0.00000000e+00	0.00000000000000e+00	0.00000000000000e+00
693	R0000693	EQ	0.00000000000000e+00	0.00000000e+00	0.00000000e+00	0.00000000000000e+00	4.08654947594839e+09
694	R0000694	EQ	0.00000000000000e+00	0.00000000e+00	0.00000000e+00	0.00000000000000e+00	0.00000000000000e+00
695	R0000695	EQ	0.00000000000000e+00	0.00000000e+00	0.00000000e+00	0.00000000000000e+00	0.00000000000000e+00
696	R0000696	EQ	0.00000000000000e+00	0.00000000e+00	0.00000000e+00	0.00000000000000e+00	0.00000000000000e+00
697	R0000697	EQ	0.00000000000000e+00	0.00000000e+00	0.00000000e+00	0.00000000000000e+00	0.00000000000000e+00
698	R0000698	EQ	0.00000000000000e+00	0.00000000e+00	0.00000000e+00	0.00000000000000e+00	0.00000000000000e+00
699	R0000699	EQ	0.00000000000000e+00	0.00000000e+00	0.00000000e+00	0.00000000000000e+00	0.00000000000000e+00
700	R0000700	EQ	0.00000000000000e+00	0.00000000e+00	0.00000000e+00	0.00000000000000e+00	0.00000000000000e+00

701	R0000701	EQ	0.00000000000000e+00	0.00000000e+00	0.00000000e+00	0.00000000000000e+00	0.00000000000000e+00
702	R0000702	EQ	0.00000000000000e+00	0.00000000e+00	0.00000000e+00	0.00000000000000e+00	0.00000000000000e+00
703	R0000703	EQ	0.00000000000000e+00	0.00000000e+00	0.00000000e+00	0.00000000000000e+00	0.00000000000000e+00
704	R0000704	EQ	0.00000000000000e+00	0.00000000e+00	0.00000000e+00	0.00000000000000e+00	0.00000000000000e+00
705	R0000705	EQ	0.00000000000000e+00	0.00000000e+00	0.00000000e+00	0.00000000000000e+00	0.00000000000000e+00
706	R0000706	SB	0.00000000000000e+00	0.00000000e+00	0.00000000e+00	0.00000000000000e+00	0.00000000000000e+00
707	R0000707	SB	-0.00000000000000e+00	0.00000000e+00	0.00000000e+00	0.00000000000000e+00	0.00000000000000e+00
708	R0000708	EQ	0.00000000000000e+00	0.00000000e+00	0.00000000e+00	0.00000000000000e+00	0.00000000000000e+00
709	R0000709	EQ	0.00000000000000e+00	0.00000000e+00	0.00000000e+00	0.00000000000000e+00	0.00000000000000e+00
710	R0000710	EQ	0.00000000000000e+00	0.00000000e+00	0.00000000e+00	0.00000000000000e+00	0.00000000000000e+00
711	R0000711	EQ	0.00000000000000e+00	0.00000000e+00	0.00000000e+00	0.00000000000000e+00	0.00000000000000e+00
712	R0000712	EQ	0.00000000000000e+00	0.00000000e+00	0.00000000e+00	0.00000000000000e+00	0.00000000000000e+00
713	R0000713	EQ	0.00000000000000e+00	0.00000000e+00	0.00000000e+00	0.00000000000000e+00	0.00000000000000e+00
714	R0000714	EQ	0.00000000000000e+00	0.00000000e+00	0.00000000e+00	0.00000000000000e+00	0.00000000000000e+00
715	R0000715	EQ	0.00000000000000e+00	0.00000000e+00	0.00000000e+00	0.00000000000000e+00	0.00000000000000e+00
716	R0000716	EQ	0.00000000000000e+00	0.00000000e+00	0.00000000e+00	0.00000000000000e+00	0.00000000000000e+00
717	R0000717	EQ	0.00000000000000e+00	0.00000000e+00	0.00000000e+00	0.00000000000000e+00	0.00000000000000e+00
718	R0000718	EQ	0.00000000000000e+00	0.00000000e+00	0.00000000e+00	0.00000000000000e+00	0.00000000000000e+00
719	R0000719	EQ	0.00000000000000e+00	0.00000000e+00	0.00000000e+00	0.00000000000000e+00	2.14748706463151e+10
720	R0000720	EQ	0.00000000000000e+00	0.00000000e+00	0.00000000e+00	0.00000000000000e+00	0.00000000000000e+00
721	R0000721	EQ	0.00000000000000e+00	0.00000000e+00	0.00000000e+00	0.00000000000000e+00	0.00000000000000e+00
722	R0000722	EQ	0.00000000000000e+00	0.00000000e+00	0.00000000e+00	0.00000000000000e+00	2.14748706463151e+10
723	R0000723	EQ	0.00000000000000e+00	0.00000000e+00	0.00000000e+00	1.06537154190665e-07	0.00000000000000e+00
724	R0000724	EQ	0.00000000000000e+00	0.00000000e+00	0.00000000e+00	1.06537154190665e-07	0.00000000000000e+00
725	R0000725	EQ	0.00000000000000e+00	0.00000000e+00	0.00000000e+00	0.00000000000000e+00	2.14748706463151e+10
726	R0000726	EQ	0.00000000000000e+00	0.00000000e+00	0.00000000e+00	0.00000000000000e+00	0.00000000000000e+00
727	R0000727	SB	0.00000000000000e+00	0.00000000e+00	0.00000000e+00	0.00000000000000e+00	0.00000000000000e+00
728	R0000728	EQ	0.00000000000000e+00	0.00000000e+00	0.00000000e+00	0.00000000000000e+00	0.00000000000000e+00
729	R0000729	EQ	0.00000000000000e+00	0.00000000e+00	0.00000000e+00	0.00000000000000e+00	0.00000000000000e+00
730	R0000730	EQ	0.00000000000000e+00	0.00000000e+00	0.00000000e+00	0.00000000000000e+00	0.00000000000000e+00
731	R0000731	EQ	0.00000000000000e+00	0.00000000e+00	0.00000000e+00	0.00000000000000e+00	0.00000000000000e+00
732	R0000732	EQ	0.00000000000000e+00	0.00000000e+00	0.00000000e+00	0.00000000000000e+00	0.00000000000000e+00
733	R0000733	EQ	0.00000000000000e+00	0.00000000e+00	0.00000000e+00	0.00000000000000e+00	0.00000000000000e+00
734	R0000734	EQ	0.00000000000000e+00	0.00000000e+00	0.00000000e+00	0.00000000000000e+00	0.00000000000000e+00
735	R0000735	EQ	0.00000000000000e+00	0.00000000e+00	0.00000000e+00	0.00000000000000e+00	0.00000000000000e+00
736	R0000736	EQ	0.00000000000000e+00	0.00000000e+00	0.00000000e+00	0.00000000000000e+00	0.00000000000000e+00
737	R0000737	EQ	0.00000000000000e+00	0.00000000e+00	0.00000000e+00	0.00000000000000e+00	0.00000000000000e+00
738	R0000738	EQ	0.00000000000000e+00	0.00000000e+00	0.00000000e+00	0.00000000000000e+00	0.00000000000000e+00
739	R0000739	EQ	0.00000000000000e+00	0.00000000e+00	0.00000000e+00	0.00000000000000e+00	0.00000000000000e+00
740	R0000740	EQ	0.00000000000000e+00	0.00000000e+00	0.00000000e+00	0.00000000000000e+00	0.00000000000000e+00
741	R0000741	EQ	0.00000000000000e+00	0.00000000e+00	0.00000000e+00	0.00000000000000e+00	0.00000000000000e+00
742	R0000742	SB	-0.00000000000000e+00	0.00000000e+00	0.00000000e+00	0.00000000000000e+00	0.00000000000000e+00
743	R0000743	EQ	0.00000000000000e+00	0.00000000e+00	0.00000000e+00	0.00000000000000e+00	0.00000000000000e+00
744	R0000744	EQ	0.00000000000000e+00	0.00000000e+00	0.00000000e+00	0.00000000000000e+00	0.00000000000000e+00
745	R0000745	EQ	0.00000000000000e+00	0.00000000e+00	0.00000000e+00	0.00000000000000e+00	6.48881073248197e+04
746	R0000746	EQ	0.00000000000000e+00	0.00000000e+00	0.00000000e+00	0.00000000000000e+00	0.00000000000000e+00
747	R0000747	SB	0.00000000000000e+00	0.00000000e+00	0.00000000e+00	0.00000000000000e+00	0.00000000000000e+00
748	R0000748	EQ	0.00000000000000e+00	0.00000000e+00	0.00000000e+00	0.00000000000000e+00	0.00000000000000e+00
749	R0000749	EQ	0.00000000000000e+00	0.00000000e+00	0.00000000e+00	0.00000000000000e+00	0.00000000000000e+00
750	R0000750	EQ	0.00000000000000e+00	0.00000000e+00	0.00000000e+00	0.00000000000000e+00	0.00000000000000e+00
751	R0000751	EQ	0.00000000000000e+00	0.00000000e+00	0.00000000e+00	0.00000000000000e+00	0.00000000000000e+00
752	R0000752	EQ	0.00000000000000e+00	0.00000000e+00	0.00000000e+00	0.00000000000000e+00	0.00000000000000e+00
753	R0000753	EQ	0.00000000000000e+00	0.00000000e+00	0.00000000e+00	0.00000000000000e+00	0.00000000000000e+00
754	R0000754	EQ	0.00000000000000e+00	0.00000000e+00	0.00000000e+00	0.00000000000000e+00	0.00000000000000e+00
755	R0000755	EQ	0.00000000000000e+00	0.00000000e+00	0.00000000e+00	0.00000000000000e+00	0.00000000000000e+00
756	R0000756	EQ	0.00000000000000e+00	0.00000000e+00	0.00000000e+00	3.78100698493817e+04	0.00000000000000e+00
757	R0000757	EQ	0.00000000000000e+00	0.00000000e+00	0.00000000e+00	3.78100698493817e+04	0.00000000000000e+00
758	R0000758	EQ	0.00000000000000e+00	0.00000000e+00	0.00000000e+00	0.00000000000000e+00	2.52709375861645e+04
759	R0000759	EQ	0.00000000000000e+00	0.00000000e+00	0.00000000e+00	3.78100698493817e+04	0.00000000000000e+00
760	R0000760	EQ	0.00000000000000e+00	0.00000000e+00	0.00000000e+00	0.00000000000000e+00	2.52709375861645e+04
761	R0000761	EQ	0.00000000000000e+00	0.00000000e+00	0.00000000e+00	3.78100698493817e+04	0.00000000000000e+00
762	R0000762	SB	0.00000000000000e+00	0.00000000e+00	0.00000000e+00	0.00000000000000e+00	0.00000000000000e+00
763	R0000763	EQ	0.00000000000000e+00	0.00000000e+00	0.00000000e+00	0.00000000000000e+00	0.00000000000000e+00
764	R0000764	EQ	0.00000000000000e+00	0.00000000e+00	0.00000000e+00	0.00000000000000e+00	0.00000000000000e+00
765	R0000765	EQ	0.00000000000000e+00	0.00000000e+00	0.00000000e+00	3.78100698493817e+04	0.00000000000000e+00
766	R0000766	EQ	0.00000000000000e+00	0.00000000e+00	0.00000000e+00	0.00000000000000e+00	0.00000000000000e+00
767	R0000767	EQ	0.00000000000000e+00	0.00000000e+00	0.00000000e+00	0.00000000000000e+00	0.00000000000000e+00
768	R0000768	EQ	0.00000000000000e+00	0.00000000e+00	0.00000000e+00	0.00000000000000e+00	0.00000000000000e+00
769	R0000769	EQ	0.00000000000000e+00	0.00000000e+00	0.00000000e+00	0.00000000000000e+00	6.48881073265076e+04
770	R0000770	EQ	0.00000000000000e+00	0.00000000e+00	0.00000000e+00	0.00000000000000e+00	6.48881073265076e+04
771	R0000771	SB	0.00000000000000e+00	0.00000000e+00	0.00000000e+00	0.00000000000000e+00	0.00000000000000e+00
772	R0000772	EQ	0.00000000000000e+00	0.00000000e+00	0.00000000e+00	0.00000000000000e+00	0.00000000000000e+00
773	R0000773	EQ	0.00000000000000e+00	0.00000000e+00	0.00000000e+00	0.00000000000000e+00	0.00000000000000e+00
774	R0000774	EQ	0.00000000000000e+00	0.00000000e+00	0.00000000e+00	0.00000000000000e+00	0.00000000000000e+00
775	R0000775	EQ	0.00000000000000e+00	0.00000000e+00	0.00000000e+00	0.00000000000000e+00	0.00000000000000e+00
776	R0000776	EQ	0.00000000000000e+00	0.00000000e+00	0.00000000e+00	0.00000000000000e+00	0.00000000000000e+00
777	R0000777	EQ	0.00000000000000e+00	0.00000000e+00	0.00000000e+00	0.00000000000000e+00	0.00000000000000e+00
778	R0000778	EQ	0.00000000000000e+00	0.00000000e+00	0.00000000e+00	0.00000000000000e+00	0.00000000000000e+00
779	R0000779	EQ	0.00000000000000e+00	0.00000000e+00	0.00000000e+00	0.00000000000000e+00	0.00000000000000e+00
780	R0000780	SB	0.00000000000000e+00	0.00000000e+00	0.00000000e+00	0.00000000000000e+00	0.00000000000000e+00
781	R0000781	SB	-0.00000000000000e+00	0.00000000e+00	0.00000000e+00	0.00000000000000e+00	0.00000000000000e+00
782	R0000782	EQ	0.00000000000000e+00	0.00000000e+00	0.00000000e+00	0.00000000000000e+00	0.00000000000000e+00
783	R0000783	EQ	0.00000000000000e+00	0.00000000e+00	0.00000000e+00	0.00000000000000e+00	0.00000000000000e+00
784	R0000784	EQ	0.00000000000000e+00	0.00000000e+00	0.00000000e+00	0.00000000000000e+00	0.00000000000000e+00
785	R0000785	EQ	0.00000000000000e+00	0.00000000e+00	0.00000000e+00	0.00000000000000e+00	0.00000000000000e+00
786	R0000786	EQ	0.00000000000000e+00	0.00000000e+00	0.00000000e+00	0.00000000000000e+00	0.00000000000000e+00
787	R0000787	EQ	0.00000000000000e+00	0.00000000e+00	0.00000000e+00	0.00000000000000e+00	0.00000000000000e+00
788	R0000788	EQ	0.00000000000000e+00	0.00000000e+00	0.00000000e+00	0.00000000000000e+00	0.00000000000000e+00
789	R0000789	EQ	0.00000000000000e+00	0.00000000e+00	0.00000000e+00	0.00000000000000e+00	0.00000000000000e+00
790	R0000790	EQ	0.00000000000000e+00	0.00000000e+00	0.00000000e+00	0.00000000000000e+00	0.00000000000000e+00
791	R0000791	EQ	0.00000000000000e+00	0.00000000e+00	0.00000000e+00	0.00000000000000e+00	0.00000000000000e+00
792	R0000792	EQ	0.00000000000000e+00	0.00000000e+00	0.00000000e+00	0.00000000000000e+00	0.00000000000000e+00
793	R0000793	EQ	0.00000000000000e+00	0.00000000e+00	0.00000000e+00	0.00000000000000e+00	0.00000000000000e+00
794	R0000794	EQ	-7.39257812520933e-06	0.00000000e+00	0.00000000e+00	0.00000000000000e+00	0.00000000000000e+00
795	R0000795	EQ	0.00000000000000e+00	0.00000000e+00	0.00000000e+00	0.00000000000000e+00	0.00000000000000e+00
796	R0000796	EQ	0.00000000000000e+00	0.00000000e+00	0.00000000e+00	0.00000000000000e+00	0.00000000000000e+00
797	R0000797	EQ	0.00000000000000e+00	0.00000000e+00	0.00000000e+00	0.00000000000000e+00	0.00000000000000e+00
798	R0000798	EQ	0.00000000000000e+00	0.00000000e+00	0.00000000e+00	0.00000000000000e+00	0.00000000000000e+00

799	R0000799	EQ 0.00000000000000e+00	0.00000000e+00	0.00000000e+00	3.55123851232251e-08	0.00000000000000e+00
800	R0000800	EQ 0.00000000000000e+00	0.00000000e+00	0.00000000e+00	3.55123851232251e-08	0.00000000000000e+00
801	R0000801	EQ 0.00000000000000e+00	0.00000000e+00	0.00000000e+00	1.42049539903395e-07	0.00000000000000e+00
802	R0000802	EQ 0.00000000000000e+00	0.00000000e+00	0.00000000e+00	1.42049539903395e-07	0.00000000000000e+00
803	R0000803	EQ 0.00000000000000e+00	0.00000000e+00	0.00000000e+00	2.74288547865025e-07	0.00000000000000e+00
804	R0000804	EQ 0.00000000000000e+00	0.00000000e+00	0.00000000e+00	2.74288547865025e-07	0.00000000000000e+00
805	R0000805	SB -0.00000000000000e+00	0.00000000e+00	0.00000000e+00	0.00000000000000e+00	0.00000000000000e+00
806	R0000806	EQ 0.00000000000000e+00	0.00000000e+00	0.00000000e+00	0.00000000000000e+00	3.81469726562500e-06
807	R0000807	EQ 0.00000000000000e+00	0.00000000e+00	0.00000000e+00	0.00000000000000e+00	0.00000000000000e+00
808	R0000808	EQ 0.00000000000000e+00	0.00000000e+00	0.00000000e+00	7.10247702464503e-08	0.00000000000000e+00
809	R0000809	EQ 0.00000000000000e+00	0.00000000e+00	0.00000000e+00	7.10247702464503e-08	0.00000000000000e+00
810	R0000810	EQ 0.00000000000000e+00	0.00000000e+00	0.00000000e+00	0.00000000000000e+00	0.00000000000000e+00
811	R0000811	EQ 0.00000000000000e+00	0.00000000e+00	0.00000000e+00	0.00000000000000e+00	0.00000000000000e+00
812	R0000812	EQ 0.00000000000000e+00	0.00000000e+00	0.00000000e+00	1.06537154190665e-07	0.00000000000000e+00
813	R0000813	EQ 0.00000000000000e+00	0.00000000e+00	0.00000000e+00	0.00000000000000e+00	0.00000000000000e+00
814	R0000814	EQ 0.00000000000000e+00	0.00000000e+00	0.00000000e+00	0.00000000000000e+00	3.81469726562500e-06
815	R0000815	EQ 0.00000000000000e+00	0.00000000e+00	0.00000000e+00	0.00000000000000e+00	3.81469726562500e-06
816	R0000816	EQ 0.00000000000000e+00	0.00000000e+00	0.00000000e+00	0.00000000000000e+00	0.00000000000000e+00
817	R0000817	SB -0.00000000000000e+00	0.00000000e+00	0.00000000e+00	0.00000000000000e+00	0.00000000000000e+00
818	R0000818	EQ 0.00000000000000e+00	0.00000000e+00	0.00000000e+00	0.00000000000000e+00	0.00000000000000e+00
819	R0000819	EQ 0.00000000000000e+00	0.00000000e+00	0.00000000e+00	0.00000000000000e+00	0.00000000000000e+00
820	R0000820	EQ 0.00000000000000e+00	0.00000000e+00	0.00000000e+00	0.00000000000000e+00	0.00000000000000e+00
821	R0000821	EQ 0.00000000000000e+00	0.00000000e+00	0.00000000e+00	0.00000000000000e+00	0.00000000000000e+00
822	R0000822	EQ 0.00000000000000e+00	0.00000000e+00	0.00000000e+00	0.00000000000000e+00	0.00000000000000e+00
823	R0000823	EQ 0.00000000000000e+00	0.00000000e+00	0.00000000e+00	0.00000000000000e+00	0.00000000000000e+00
824	R0000824	EQ 0.00000000000000e+00	0.00000000e+00	0.00000000e+00	0.00000000000000e+00	0.00000000000000e+00
825	R0000825	EQ 0.00000000000000e+00	0.00000000e+00	0.00000000e+00	0.00000000000000e+00	0.00000000000000e+00
826	R0000826	EQ 0.00000000000000e+00	0.00000000e+00	0.00000000e+00	0.00000000000000e+00	0.00000000000000e+00
827	R0000827	EQ 0.00000000000000e+00	0.00000000e+00	0.00000000e+00	0.00000000000000e+00	0.00000000000000e+00
828	R0000828	EQ 0.00000000000000e+00	0.00000000e+00	0.00000000e+00	0.00000000000000e+00	0.00000000000000e+00
829	R0000829	EQ 0.00000000000000e+00	0.00000000e+00	0.00000000e+00	0.00000000000000e+00	0.00000000000000e+00
830	R0000830	EQ 0.00000000000000e+00	0.00000000e+00	0.00000000e+00	0.00000000000000e+00	0.00000000000000e+00
831	R0000831	EQ 0.00000000000000e+00	0.00000000e+00	0.00000000e+00	0.00000000000000e+00	0.00000000000000e+00
832	R0000832	EQ 0.00000000000000e+00	0.00000000e+00	0.00000000e+00	0.00000000000000e+00	0.00000000000000e+00
833	R0000833	EQ 0.00000000000000e+00	0.00000000e+00	0.00000000e+00	0.00000000000000e+00	0.00000000000000e+00
834	R0000834	EQ 0.00000000000000e+00	0.00000000e+00	0.00000000e+00	0.00000000000000e+00	0.00000000000000e+00
835	R0000835	SB 0.00000000000000e+00	0.00000000e+00	0.00000000e+00	0.00000000000000e+00	0.00000000000000e+00
836	R0000836	EQ 0.00000000000000e+00	0.00000000e+00	0.00000000e+00	0.00000000000000e+00	0.00000000000000e+00
837	R0000837	SB 0.00000000000000e+00	0.00000000e+00	0.00000000e+00	0.00000000000000e+00	0.00000000000000e+00
838	R0000838	EQ 0.00000000000000e+00	0.00000000e+00	0.00000000e+00	0.00000000000000e+00	0.00000000000000e+00
839	R0000839	EQ 0.00000000000000e+00	0.00000000e+00	0.00000000e+00	0.00000000000000e+00	0.00000000000000e+00
840	R0000840	EQ 0.00000000000000e+00	0.00000000e+00	0.00000000e+00	0.00000000000000e+00	0.00000000000000e+00
841	R0000841	EQ 0.00000000000000e+00	0.00000000e+00	0.00000000e+00	0.00000000000000e+00	0.00000000000000e+00
842	R0000842	SB 0.00000000000000e+00	0.00000000e+00	0.00000000e+00	0.00000000000000e+00	0.00000000000000e+00
843	R0000843	EQ 0.00000000000000e+00	0.00000000e+00	0.00000000e+00	0.00000000000000e+00	0.00000000000000e+00
844	R0000844	EQ 0.00000000000000e+00	0.00000000e+00	0.00000000e+00	0.00000000000000e+00	0.00000000000000e+00
845	R0000845	EQ 0.00000000000000e+00	0.00000000e+00	0.00000000e+00	0.00000000000000e+00	0.00000000000000e+00
846	R0000846	EQ 0.00000000000000e+00	0.00000000e+00	0.00000000e+00	0.00000000000000e+00	0.00000000000000e+00
847	R0000847	EQ 0.00000000000000e+00	0.00000000e+00	0.00000000e+00	0.00000000000000e+00	0.00000000000000e+00
848	R0000848	EQ 0.00000000000000e+00	0.00000000e+00	0.00000000e+00	0.00000000000000e+00	0.00000000000000e+00
849	R0000849	EQ 0.00000000000000e+00	0.00000000e+00	0.00000000e+00	0.00000000000000e+00	0.00000000000000e+00
850	R0000850	EQ 0.00000000000000e+00	0.00000000e+00	0.00000000e+00	0.00000000000000e+00	0.00000000000000e+00
851	R0000851	EQ 0.00000000000000e+00	0.00000000e+00	0.00000000e+00	0.00000000000000e+00	6.48881073248197e+04
852	R0000852	EQ 0.00000000000000e+00	0.00000000e+00	0.00000000e+00	0.00000000000000e+00	6.48881073248197e+04
853	R0000853	EQ 0.00000000000000e+00	0.00000000e+00	0.00000000e+00	0.00000000000000e+00	0.00000000000000e+00
854	R0000854	EQ 0.00000000000000e+00	0.00000000e+00	0.00000000e+00	0.00000000000000e+00	0.00000000000000e+00
855	R0000855	EQ 0.00000000000000e+00	0.00000000e+00	0.00000000e+00	0.00000000000000e+00	0.00000000000000e+00
856	R0000856	EQ 0.00000000000000e+00	0.00000000e+00	0.00000000e+00	0.00000000000000e+00	0.00000000000000e+00
857	R0000857	EQ 0.00000000000000e+00	0.00000000e+00	0.00000000e+00	0.00000000000000e+00	0.00000000000000e+00
858	R0000858	EQ 0.00000000000000e+00	0.00000000e+00	0.00000000e+00	0.00000000000000e+00	0.00000000000000e+00
859	R0000859	EQ 0.00000000000000e+00	0.00000000e+00	0.00000000e+00	0.00000000000000e+00	4.08654947594839e+09
860	R0000860	EQ 0.00000000000000e+00	0.00000000e+00	0.00000000e+00	0.00000000000000e+00	0.00000000000000e+00
861	R0000861	EQ 0.00000000000000e+00	0.00000000e+00	0.00000000e+00	0.00000000000000e+00	0.00000000000000e+00
862	R0000862	EQ 0.00000000000000e+00	0.00000000e+00	0.00000000e+00	0.00000000000000e+00	0.00000000000000e+00
863	R0000863	EQ 0.00000000000000e+00	0.00000000e+00	0.00000000e+00	0.00000000000000e+00	0.00000000000000e+00
864	R0000864	SB 0.00000000000000e+00	0.00000000e+00	0.00000000e+00	0.00000000000000e+00	0.00000000000000e+00
865	R0000865	EQ 0.00000000000000e+00	0.00000000e+00	0.00000000e+00	0.00000000000000e+00	0.00000000000000e+00
866	R0000866	EQ 0.00000000000000e+00	0.00000000e+00	0.00000000e+00	0.00000000000000e+00	3.81469726562500e-06
867	R0000867	EQ 0.00000000000000e+00	0.00000000e+00	0.00000000e+00	0.00000000000000e+00	0.00000000000000e+00
868	R0000868	EQ 0.00000000000000e+00	0.00000000e+00	0.00000000e+00	0.00000000000000e+00	2.14748706463151e+10
869	R0000869	EQ 0.00000000000000e+00	0.00000000e+00	0.00000000e+00	0.00000000000000e+00	3.81469726562500e-06
870	R0000870	EQ 0.00000000000000e+00	0.00000000e+00	0.00000000e+00	0.00000000000000e+00	3.81469726562500e-06
871	R0000871	EQ 0.00000000000000e+00	0.00000000e+00	0.00000000e+00	0.00000000000000e+00	2.14748706463151e+10
872	R0000872	EQ 0.00000000000000e+00	0.00000000e+00	0.00000000e+00	0.00000000000000e+00	0.00000000000000e+00
873	R0000873	EQ 0.00000000000000e+00	0.00000000e+00	0.00000000e+00	0.00000000000000e+00	0.00000000000000e+00
874	R0000874	EQ 0.00000000000000e+00	0.00000000e+00	0.00000000e+00	0.00000000000000e+00	0.00000000000000e+00
875	R0000875	EQ 0.00000000000000e+00	0.00000000e+00	0.00000000e+00	0.00000000000000e+00	0.00000000000000e+00
876	R0000876	EQ 0.00000000000000e+00	0.00000000e+00	0.00000000e+00	0.00000000000000e+00	0.00000000000000e+00
877	R0000877	EQ 0.00000000000000e+00	0.00000000e+00	0.00000000e+00	0.00000000000000e+00	0.00000000000000e+00
878	R0000878	SB 0.00000000000000e+00	0.00000000e+00	0.00000000e+00	0.00000000000000e+00	0.00000000000000e+00
879	R0000879	EQ 0.00000000000000e+00	0.00000000e+00	0.00000000e+00	0.00000000000000e+00	0.00000000000000e+00
880	R0000880	SB 0.00000000000000e+00	0.00000000e+00	0.00000000e+00	0.00000000000000e+00	0.00000000000000e+00
881	R0000881	EQ 0.00000000000000e+00	0.00000000e+00	0.00000000e+00	0.00000000000000e+00	0.00000000000000e+00
882	R0000882	EQ 0.00000000000000e+00	0.00000000e+00	0.00000000e+00	0.00000000000000e+00	0.00000000000000e+00
883	R0000883	EQ 0.00000000000000e+00	0.00000000e+00	0.00000000e+00	0.00000000000000e+00	0.00000000000000e+00
884	R0000884	EQ 0.00000000000000e+00	0.00000000e+00	0.00000000e+00	0.00000000000000e+00	0.00000000000000e+00
885	R0000885	EQ 0.00000000000000e+00	0.00000000e+00	0.00000000e+00	0.00000000000000e+00	0.00000000000000e+00
886	R0000886	EQ 0.00000000000000e+00	0.00000000e+00	0.00000000e+00	0.00000000000000e+00	0.00000000000000e+00
887	R0000887	EQ 0.00000000000000e+00	0.00000000e+00	0.00000000e+00	0.00000000000000e+00	2.14748706463151e+10
888	R0000888	EQ 0.00000000000000e+00	0.00000000e+00	0.00000000e+00	0.00000000000000e+00	2.14748706463151e+10
889	R0000889	EQ 0.00000000000000e+00	0.00000000e+00	0.00000000e+00	0.00000000000000e+00	2.14748706463151e+10
890	R0000890	EQ 0.00000000000000e+00	0.00000000e+00	0.00000000e+00	0.00000000000000e+00	2.14748706463151e+10
891	R0000891	EQ 0.00000000000000e+00	0.00000000e+00	0.00000000e+00	0.00000000000000e+00	2.14748706463151e+10
892	R0000892	EQ 0.00000000000000e+00	0.00000000e+00	0.00000000e+00	0.00000000000000e+00	2.14748706463151e+10
893	R0000893	EQ 0.00000000000000e+00	0.00000000e+00	0.00000000e+00	0.00000000000000e+00	2.14748706463151e+10
894	R0000894	EQ 0.00000000000000e+00	0.00000000e+00	0.00000000e+00	0.00000000000000e+00	2.14748706463151e+10
895	R0000895	EQ 0.00000000000000e+00	0.00000000e+00	0.00000000e+00	0.00000000000000e+00	2.14748706463151e+10
896	R0000896	EQ 0.00000000000000e+00	0.00000000e+00	0.00000000e+00	0.00000000000000e+00	2.14748706463151e+10

897	R0000897	EQ 0.00000000000000e+00	0.00000000e+00	0.00000000e+00	0.00000000000000e+00	2.14748706463151e+10
898	R0000898	SB -0.00000000000000e+00	0.00000000e+00	0.00000000e+00	0.00000000000000e+00	0.00000000000000e+00
899	R0000899	SB -0.00000000000000e+00	0.00000000e+00	0.00000000e+00	0.00000000000000e+00	0.00000000000000e+00
900	R0000900	EQ 0.00000000000000e+00	0.00000000e+00	0.00000000e+00	0.00000000000000e+00	2.14748706463151e+10
901	R0000901	EQ 0.00000000000000e+00	0.00000000e+00	0.00000000e+00	0.00000000000000e+00	2.14748706463151e+10
902	R0000902	EQ 0.00000000000000e+00	0.00000000e+00	0.00000000e+00	0.00000000000000e+00	2.14748706463151e+10
903	R0000903	EQ 0.00000000000000e+00	0.00000000e+00	0.00000000e+00	0.00000000000000e+00	2.14748706463151e+10
904	R0000904	EQ 0.00000000000000e+00	0.00000000e+00	0.00000000e+00	0.00000000000000e+00	0.00000000000000e+00
905	R0000905	EQ 0.00000000000000e+00	0.00000000e+00	0.00000000e+00	3.55123851232251e-08	0.00000000000000e+00
906	R0000906	EQ 0.00000000000000e+00	0.00000000e+00	0.00000000e+00	3.55123851232251e-08	0.00000000000000e+00
907	R0000907	EQ 0.00000000000000e+00	0.00000000e+00	0.00000000e+00	1.42049539903395e-07	0.00000000000000e+00
908	R0000908	EQ 0.00000000000000e+00	0.00000000e+00	0.00000000e+00	1.42049539903395e-07	0.00000000000000e+00
909	R0000909	EQ 0.00000000000000e+00	0.00000000e+00	0.00000000e+00	0.00000000000000e+00	2.14748706463151e+10
910	R0000910	EQ 0.00000000000000e+00	0.00000000e+00	0.00000000e+00	0.00000000000000e+00	2.14748706463151e+10
911	R0000911	EQ 0.00000000000000e+00	0.00000000e+00	0.00000000e+00	0.00000000000000e+00	2.14748706463151e+10
912	R0000912	EQ 0.00000000000000e+00	0.00000000e+00	0.00000000e+00	0.00000000000000e+00	2.14748706463151e+10
913	R0000913	EQ 0.00000000000000e+00	0.00000000e+00	0.00000000e+00	0.00000000000000e+00	2.14748706463151e+10
914	R0000914	EQ 0.00000000000000e+00	0.00000000e+00	0.00000000e+00	2.74288547865025e-07	0.00000000000000e+00
915	R0000915	EQ 0.00000000000000e+00	0.00000000e+00	0.00000000e+00	2.74288547865025e-07	0.00000000000000e+00
916	R0000916	EQ 0.00000000000000e+00	0.00000000e+00	0.00000000e+00	7.10247702464503e-08	0.00000000000000e+00
917	R0000917	EQ 0.00000000000000e+00	0.00000000e+00	0.00000000e+00	7.10247702464503e-08	0.00000000000000e+00
918	R0000918	EQ 0.00000000000000e+00	0.00000000e+00	0.00000000e+00	0.00000000000000e+00	0.00000000000000e+00
919	R0000919	EQ 0.00000000000000e+00	0.00000000e+00	0.00000000e+00	0.00000000000000e+00	0.00000000000000e+00
920	R0000920	EQ 0.00000000000000e+00	0.00000000e+00	0.00000000e+00	1.06537154190665e-07	0.00000000000000e+00
921	R0000921	EQ 0.00000000000000e+00	0.00000000e+00	0.00000000e+00	0.00000000000000e+00	0.00000000000000e+00
922	R0000922	EQ 0.00000000000000e+00	0.00000000e+00	0.00000000e+00	0.00000000000000e+00	0.00000000000000e+00
923	R0000923	EQ 0.00000000000000e+00	0.00000000e+00	0.00000000e+00	0.00000000000000e+00	0.00000000000000e+00
924	R0000924	EQ 0.00000000000000e+00	0.00000000e+00	0.00000000e+00	0.00000000000000e+00	3.81469726562500e-06
925	R0000925	EQ 0.00000000000000e+00	0.00000000e+00	0.00000000e+00	0.00000000000000e+00	3.81469726562500e-06
926	R0000926	EQ 0.00000000000000e+00	0.00000000e+00	0.00000000e+00	0.00000000000000e+00	0.00000000000000e+00
927	R0000927	EQ 0.00000000000000e+00	0.00000000e+00	0.00000000e+00	5.92849404983521e+04	0.00000000000000e+00
928	R0000928	EQ 0.00000000000000e+00	0.00000000e+00	0.00000000e+00	0.00000000000000e+00	0.00000000000000e+00
929	R0000929	EQ 0.00000000000000e+00	0.00000000e+00	0.00000000e+00	0.00000000000000e+00	0.00000000000000e+00
930	R0000930	EQ 0.00000000000000e+00	0.00000000e+00	0.00000000e+00	0.00000000000000e+00	0.00000000000000e+00
931	R0000931	EQ 0.00000000000000e+00	0.00000000e+00	0.00000000e+00	0.00000000000000e+00	0.00000000000000e+00
932	R0000932	EQ 0.00000000000000e+00	0.00000000e+00	0.00000000e+00	0.00000000000000e+00	0.00000000000000e+00
933	R0000933	EQ 0.00000000000000e+00	0.00000000e+00	0.00000000e+00	0.00000000000000e+00	0.00000000000000e+00
934	R0000934	EQ 0.00000000000000e+00	0.00000000e+00	0.00000000e+00	0.00000000000000e+00	0.00000000000000e+00
935	R0000935	EQ 0.00000000000000e+00	0.00000000e+00	0.00000000e+00	0.00000000000000e+00	0.00000000000000e+00
936	R0000936	EQ 0.00000000000000e+00	0.00000000e+00	0.00000000e+00	0.00000000000000e+00	0.00000000000000e+00
937	R0000937	EQ 0.00000000000000e+00	0.00000000e+00	0.00000000e+00	0.00000000000000e+00	0.00000000000000e+00
938	R0000938	EQ 0.00000000000000e+00	0.00000000e+00	0.00000000e+00	0.00000000000000e+00	0.00000000000000e+00
939	R0000939	EQ 0.00000000000000e+00	0.00000000e+00	0.00000000e+00	0.00000000000000e+00	0.00000000000000e+00
940	R0000940	EQ 0.00000000000000e+00	0.00000000e+00	0.00000000e+00	0.00000000000000e+00	0.00000000000000e+00
941	R0000941	EQ 0.00000000000000e+00	0.00000000e+00	0.00000000e+00	0.00000000000000e+00	0.00000000000000e+00
942	R0000942	EQ 0.00000000000000e+00	0.00000000e+00	0.00000000e+00	0.00000000000000e+00	0.00000000000000e+00
943	R0000943	EQ 0.00000000000000e+00	0.00000000e+00	0.00000000e+00	0.00000000000000e+00	0.00000000000000e+00
944	R0000944	SB -0.00000000000000e+00	0.00000000e+00	0.00000000e+00	0.00000000000000e+00	0.00000000000000e+00
945	R0000945	EQ 0.00000000000000e+00	0.00000000e+00	0.00000000e+00	0.00000000000000e+00	0.00000000000000e+00
946	R0000946	EQ 0.00000000000000e+00	0.00000000e+00	0.00000000e+00	0.00000000000000e+00	0.00000000000000e+00
947	R0000947	EQ 0.00000000000000e+00	0.00000000e+00	0.00000000e+00	0.00000000000000e+00	0.00000000000000e+00
948	R0000948	EQ 0.00000000000000e+00	0.00000000e+00	0.00000000e+00	0.00000000000000e+00	0.00000000000000e+00
949	R0000949	EQ 0.00000000000000e+00	0.00000000e+00	0.00000000e+00	0.00000000000000e+00	0.00000000000000e+00
950	R0000950	EQ 0.00000000000000e+00	0.00000000e+00	0.00000000e+00	0.00000000000000e+00	0.00000000000000e+00
951	R0000951	EQ 0.00000000000000e+00	0.00000000e+00	0.00000000e+00	0.00000000000000e+00	0.00000000000000e+00
952	R0000952	EQ 0.00000000000000e+00	0.00000000e+00	0.00000000e+00	0.00000000000000e+00	0.00000000000000e+00
953	R0000953	EQ 0.00000000000000e+00	0.00000000e+00	0.00000000e+00	0.00000000000000e+00	0.00000000000000e+00
954	R0000954	EQ 0.00000000000000e+00	0.00000000e+00	0.00000000e+00	0.00000000000000e+00	0.00000000000000e+00
955	R0000955	EQ 0.00000000000000e+00	0.00000000e+00	0.00000000e+00	0.00000000000000e+00	0.00000000000000e+00
956	R0000956	EQ 0.00000000000000e+00	0.00000000e+00	0.00000000e+00	0.00000000000000e+00	0.00000000000000e+00
957	R0000957	EQ 0.00000000000000e+00	0.00000000e+00	0.00000000e+00	0.00000000000000e+00	0.00000000000000e+00
958	R0000958	EQ 0.00000000000000e+00	0.00000000e+00	0.00000000e+00	0.00000000000000e+00	0.00000000000000e+00
959	R0000959	EQ 0.00000000000000e+00	0.00000000e+00	0.00000000e+00	0.00000000000000e+00	0.00000000000000e+00
960	R0000960	EQ 0.00000000000000e+00	0.00000000e+00	0.00000000e+00	0.00000000000000e+00	0.00000000000000e+00
961	R0000961	EQ 5.55111512312578e-17	0.00000000e+00	0.00000000e+00	0.00000000000000e+00	0.00000000000000e+00
962	R0000962	EQ 0.00000000000000e+00	0.00000000e+00	0.00000000e+00	0.00000000000000e+00	0.00000000000000e+00
963	R0000963	EQ 0.00000000000000e+00	0.00000000e+00	0.00000000e+00	0.00000000000000e+00	0.00000000000000e+00
964	R0000964	EQ -1.11022302462516e-16	0.00000000e+00	0.00000000e+00	0.00000000000000e+00	0.00000000000000e+00
965	R0000965	EQ 0.00000000000000e+00	0.00000000e+00	0.00000000e+00	0.00000000000000e+00	0.00000000000000e+00
966	R0000966	EQ 0.00000000000000e+00	0.00000000e+00	0.00000000e+00	0.00000000000000e+00	0.00000000000000e+00
967	R0000967	EQ 0.00000000000000e+00	0.00000000e+00	0.00000000e+00	0.00000000000000e+00	0.00000000000000e+00
968	R0000968	EQ 0.00000000000000e+00	0.00000000e+00	0.00000000e+00	0.00000000000000e+00	0.00000000000000e+00
969	R0000969	EQ 0.00000000000000e+00	0.00000000e+00	0.00000000e+00	0.00000000000000e+00	0.00000000000000e+00
970	R0000970	EQ 0.00000000000000e+00	0.00000000e+00	0.00000000e+00	0.00000000000000e+00	0.00000000000000e+00
971	R0000971	EQ 0.00000000000000e+00	0.00000000e+00	0.00000000e+00	0.00000000000000e+00	0.00000000000000e+00
972	R0000972	EQ 0.00000000000000e+00	0.00000000e+00	0.00000000e+00	0.00000000000000e+00	0.00000000000000e+00
973	R0000973	EQ 0.00000000000000e+00	0.00000000e+00	0.00000000e+00	0.00000000000000e+00	0.00000000000000e+00
974	R0000974	EQ 0.00000000000000e+00	0.00000000e+00	0.00000000e+00	0.00000000000000e+00	0.00000000000000e+00
975	R0000975	EQ 0.00000000000000e+00	0.00000000e+00	0.00000000e+00	0.00000000000000e+00	0.00000000000000e+00
976	R0000976	EQ 0.00000000000000e+00	0.00000000e+00	0.00000000e+00	0.00000000000000e+00	0.00000000000000e+00
977	R0000977	EQ 0.00000000000000e+00	0.00000000e+00	0.00000000e+00	0.00000000000000e+00	0.00000000000000e+00
978	R0000978	EQ 0.00000000000000e+00	0.00000000e+00	0.00000000e+00	0.00000000000000e+00	0.00000000000000e+00
979	R0000979	EQ 0.00000000000000e+00	0.00000000e+00	0.00000000e+00	0.00000000000000e+00	0.00000000000000e+00
980	R0000980	EQ 0.00000000000000e+00	0.00000000e+00	0.00000000e+00	0.00000000000000e+00	0.00000000000000e+00
981	R0000981	EQ 0.00000000000000e+00	0.00000000e+00	0.00000000e+00	0.00000000000000e+00	0.00000000000000e+00
982	R0000982	EQ 0.00000000000000e+00	0.00000000e+00	0.00000000e+00	0.00000000000000e+00	0.00000000000000e+00
983	R0000983	EQ 0.00000000000000e+00	0.00000000e+00	0.00000000e+00	0.00000000000000e+00	0.00000000000000e+00
984	R0000984	EQ 0.00000000000000e+00	0.00000000e+00	0.00000000e+00	0.00000000000000e+00	0.00000000000000e+00
985	R0000985	EQ 0.00000000000000e+00	0.00000000e+00	0.00000000e+00	0.00000000000000e+00	0.00000000000000e+00
986	R0000986	EQ 0.00000000000000e+00	0.00000000e+00	0.00000000e+00	0.00000000000000e+00	0.00000000000000e+00
987	R0000987	EQ 0.00000000000000e+00	0.00000000e+00	0.00000000e+00	0.00000000000000e+00	0.00000000000000e+00
988	R0000988	EQ 0.00000000000000e+00	0.00000000e+00	0.00000000e+00	0.00000000000000e+00	0.00000000000000e+00
989	R0000989	EQ 0.00000000000000e+00	0.00000000e+00	0.00000000e+00	0.00000000000000e+00	0.00000000000000e+00
990	R0000990	EQ 0.00000000000000e+00	0.00000000e+00	0.00000000e+00	0.00000000000000e+00	0.00000000000000e+00
991	R0000991	EQ 0.00000000000000e+00	0.00000000e+00	0.00000000e+00	0.00000000000000e+00	0.00000000000000e+00
992	R0000992	EQ 0.00000000000000e+00	0.00000000e+00	0.00000000e+00	0.00000000000000e+00	0.00000000000000e+00
993	R0000993	EQ 0.00000000000000e+00	0.00000000e+00	0.00000000e+00	0.00000000000000e+00	0.00000000000000e+00
994	R0000994	EQ 0.00000000000000e+00	0.00000000e+00	0.00000000e+00	0.00000000000000e+00	0.00000000000000e+00

995	R0000995	EQ 0.00000000000000e+00	0.00000000e+00	0.00000000e+00	0.00000000000000e+00	0.00000000000000e+00
996	R0000996	EQ 0.00000000000000e+00	0.00000000e+00	0.00000000e+00	0.00000000000000e+00	0.00000000000000e+00
997	R0000997	EQ 0.00000000000000e+00	0.00000000e+00	0.00000000e+00	0.00000000000000e+00	0.00000000000000e+00
998	R0000998	EQ 0.00000000000000e+00	0.00000000e+00	0.00000000e+00	0.00000000000000e+00	0.00000000000000e+00
999	R0000999	EQ 0.00000000000000e+00	0.00000000e+00	0.00000000e+00	0.00000000000000e+00	0.00000000000000e+00
1000	R0001000	EQ 0.00000000000000e+00	0.00000000e+00	0.00000000e+00	0.00000000000000e+00	0.00000000000000e+00
1001	R0001001	EQ 0.00000000000000e+00	0.00000000e+00	0.00000000e+00	0.00000000000000e+00	0.00000000000000e+00
1002	R0001002	EQ 0.00000000000000e+00	0.00000000e+00	0.00000000e+00	0.00000000000000e+00	0.00000000000000e+00
1003	R0001003	EQ 0.00000000000000e+00	0.00000000e+00	0.00000000e+00	0.00000000000000e+00	0.00000000000000e+00
1004	R0001004	EQ 0.00000000000000e+00	0.00000000e+00	0.00000000e+00	0.00000000000000e+00	0.00000000000000e+00
1005	R0001005	EQ 0.00000000000000e+00	0.00000000e+00	0.00000000e+00	0.00000000000000e+00	0.00000000000000e+00
1006	R0001006	EQ 0.00000000000000e+00	0.00000000e+00	0.00000000e+00	0.00000000000000e+00	0.00000000000000e+00
1007	R0001007	EQ 0.00000000000000e+00	0.00000000e+00	0.00000000e+00	0.00000000000000e+00	0.00000000000000e+00
1008	R0001008	EQ 0.00000000000000e+00	0.00000000e+00	0.00000000e+00	0.00000000000000e+00	0.00000000000000e+00
1009	R0001009	EQ 0.00000000000000e+00	0.00000000e+00	0.00000000e+00	0.00000000000000e+00	0.00000000000000e+00
1010	R0001010	EQ 0.00000000000000e+00	0.00000000e+00	0.00000000e+00	0.00000000000000e+00	0.00000000000000e+00
1011	R0001011	EQ 0.00000000000000e+00	0.00000000e+00	0.00000000e+00	0.00000000000000e+00	0.00000000000000e+00
1012	R0001012	EQ 0.00000000000000e+00	0.00000000e+00	0.00000000e+00	0.00000000000000e+00	0.00000000000000e+00
1013	R0001013	EQ 0.00000000000000e+00	0.00000000e+00	0.00000000e+00	0.00000000000000e+00	0.00000000000000e+00
1014	R0001014	EQ 0.00000000000000e+00	0.00000000e+00	0.00000000e+00	0.00000000000000e+00	0.00000000000000e+00
1015	R0001015	EQ 0.00000000000000e+00	0.00000000e+00	0.00000000e+00	0.00000000000000e+00	0.00000000000000e+00
1016	R0001016	EQ 0.00000000000000e+00	0.00000000e+00	0.00000000e+00	0.00000000000000e+00	0.00000000000000e+00
1017	R0001017	EQ 0.00000000000000e+00	0.00000000e+00	0.00000000e+00	0.00000000000000e+00	0.00000000000000e+00
1018	R0001018	EQ 0.00000000000000e+00	0.00000000e+00	0.00000000e+00	0.00000000000000e+00	0.00000000000000e+00
1019	R0001019	EQ 0.00000000000000e+00	0.00000000e+00	0.00000000e+00	0.00000000000000e+00	0.00000000000000e+00
1020	R0001020	EQ 0.00000000000000e+00	0.00000000e+00	0.00000000e+00	0.00000000000000e+00	0.00000000000000e+00
1021	R0001021	EQ 0.00000000000000e+00	0.00000000e+00	0.00000000e+00	0.00000000000000e+00	0.00000000000000e+00
1022	R0001022	EQ 0.00000000000000e+00	0.00000000e+00	0.00000000e+00	0.00000000000000e+00	0.00000000000000e+00
1023	R0001023	EQ 0.00000000000000e+00	0.00000000e+00	0.00000000e+00	0.00000000000000e+00	0.00000000000000e+00
1024	R0001024	EQ 0.00000000000000e+00	0.00000000e+00	0.00000000e+00	0.00000000000000e+00	0.00000000000000e+00
1025	R0001025	EQ 0.00000000000000e+00	0.00000000e+00	0.00000000e+00	0.00000000000000e+00	0.00000000000000e+00
1026	R0001026	EQ 0.00000000000000e+00	0.00000000e+00	0.00000000e+00	0.00000000000000e+00	0.00000000000000e+00
1027	R0001027	EQ 0.00000000000000e+00	0.00000000e+00	0.00000000e+00	0.00000000000000e+00	0.00000000000000e+00
1028	R0001028	EQ 0.00000000000000e+00	0.00000000e+00	0.00000000e+00	0.00000000000000e+00	0.00000000000000e+00
1029	R0001029	EQ 0.00000000000000e+00	0.00000000e+00	0.00000000e+00	0.00000000000000e+00	0.00000000000000e+00
1030	R0001030	EQ 0.00000000000000e+00	0.00000000e+00	0.00000000e+00	0.00000000000000e+00	0.00000000000000e+00
1031	R0001031	EQ 0.00000000000000e+00	0.00000000e+00	0.00000000e+00	0.00000000000000e+00	0.00000000000000e+00
1032	R0001032	EQ 0.00000000000000e+00	0.00000000e+00	0.00000000e+00	0.00000000000000e+00	0.00000000000000e+00
1033	R0001033	EQ 0.00000000000000e+00	0.00000000e+00	0.00000000e+00	0.00000000000000e+00	0.00000000000000e+00
1034	R0001034	EQ 0.00000000000000e+00	0.00000000e+00	0.00000000e+00	0.00000000000000e+00	0.00000000000000e+00
1035	R0001035	EQ 0.00000000000000e+00	0.00000000e+00	0.00000000e+00	0.00000000000000e+00	0.00000000000000e+00
1036	R0001036	EQ 0.00000000000000e+00	0.00000000e+00	0.00000000e+00	0.00000000000000e+00	0.00000000000000e+00
1037	R0001037	EQ 0.00000000000000e+00	0.00000000e+00	0.00000000e+00	0.00000000000000e+00	0.00000000000000e+00
1038	R0001038	EQ 0.00000000000000e+00	0.00000000e+00	0.00000000e+00	0.00000000000000e+00	0.00000000000000e+00
1039	R0001039	EQ 0.00000000000000e+00	0.00000000e+00	0.00000000e+00	0.00000000000000e+00	0.00000000000000e+00
1040	R0001040	EQ 0.00000000000000e+00	0.00000000e+00	0.00000000e+00	0.00000000000000e+00	0.00000000000000e+00
1041	R0001041	EQ 0.00000000000000e+00	0.00000000e+00	0.00000000e+00	0.00000000000000e+00	0.00000000000000e+00
1042	R0001042	EQ 0.00000000000000e+00	0.00000000e+00	0.00000000e+00	0.00000000000000e+00	0.00000000000000e+00
1043	R0001043	EQ 0.00000000000000e+00	0.00000000e+00	0.00000000e+00	0.00000000000000e+00	0.00000000000000e+00
1044	R0001044	EQ 0.00000000000000e+00	0.00000000e+00	0.00000000e+00	0.00000000000000e+00	0.00000000000000e+00
1045	R0001045	EQ 0.00000000000000e+00	0.00000000e+00	0.00000000e+00	0.00000000000000e+00	0.00000000000000e+00
1046	R0001046	EQ 0.00000000000000e+00	0.00000000e+00	0.00000000e+00	0.00000000000000e+00	0.00000000000000e+00
1047	R0001047	EQ 0.00000000000000e+00	0.00000000e+00	0.00000000e+00	0.00000000000000e+00	0.00000000000000e+00
1048	R0001048	EQ 0.00000000000000e+00	0.00000000e+00	0.00000000e+00	0.00000000000000e+00	0.00000000000000e+00
1049	R0001049	EQ 0.00000000000000e+00	0.00000000e+00	0.00000000e+00	0.00000000000000e+00	0.00000000000000e+00
1050	R0001050	EQ 0.00000000000000e+00	0.00000000e+00	0.00000000e+00	0.00000000000000e+00	0.00000000000000e+00
1051	R0001051	EQ 0.00000000000000e+00	0.00000000e+00	0.00000000e+00	0.00000000000000e+00	0.00000000000000e+00
1052	R0001052	EQ 0.00000000000000e+00	0.00000000e+00	0.00000000e+00	0.00000000000000e+00	0.00000000000000e+00
1053	R0001053	EQ 0.00000000000000e+00	0.00000000e+00	0.00000000e+00	0.00000000000000e+00	0.00000000000000e+00
1054	R0001054	EQ 0.00000000000000e+00	0.00000000e+00	0.00000000e+00	0.00000000000000e+00	0.00000000000000e+00
1055	R0001055	EQ 0.00000000000000e+00	0.00000000e+00	0.00000000e+00	0.00000000000000e+00	0.00000000000000e+00
1056	R0001056	EQ 0.00000000000000e+00	0.00000000e+00	0.00000000e+00	0.00000000000000e+00	0.00000000000000e+00
1057	R0001057	EQ 0.00000000000000e+00	0.00000000e+00	0.00000000e+00	0.00000000000000e+00	0.00000000000000e+00
1058	R0001058	EQ 0.00000000000000e+00	0.00000000e+00	0.00000000e+00	0.00000000000000e+00	0.00000000000000e+00
1059	R0001059	EQ 0.00000000000000e+00	0.00000000e+00	0.00000000e+00	0.00000000000000e+00	0.00000000000000e+00
1060	R0001060	EQ 0.00000000000000e+00	0.00000000e+00	0.00000000e+00	0.00000000000000e+00	0.00000000000000e+00
1061	R0001061	EQ 0.00000000000000e+00	0.00000000e+00	0.00000000e+00	0.00000000000000e+00	0.00000000000000e+00
1062	R0001062	EQ 0.00000000000000e+00	0.00000000e+00	0.00000000e+00	0.00000000000000e+00	0.00000000000000e+00
1063	R0001063	EQ 0.00000000000000e+00	0.00000000e+00	0.00000000e+00	0.00000000000000e+00	0.00000000000000e+00
1064	R0001064	EQ 0.00000000000000e+00	0.00000000e+00	0.00000000e+00	0.00000000000000e+00	0.00000000000000e+00
1065	R0001065	EQ 0.00000000000000e+00	0.00000000e+00	0.00000000e+00	0.00000000000000e+00	0.00000000000000e+00
1066	R0001066	EQ 0.00000000000000e+00	0.00000000e+00	0.00000000e+00	0.00000000000000e+00	0.00000000000000e+00
1067	R0001067	EQ 0.00000000000000e+00	0.00000000e+00	0.00000000e+00	0.00000000000000e+00	0.00000000000000e+00
1068	R0001068	EQ 0.00000000000000e+00	0.00000000e+00	0.00000000e+00	0.00000000000000e+00	0.00000000000000e+00
1069	R0001069	EQ 0.00000000000000e+00	0.00000000e+00	0.00000000e+00	0.00000000000000e+00	0.00000000000000e+00
1070	R0001070	EQ 0.00000000000000e+00	0.00000000e+00	0.00000000e+00	0.00000000000000e+00	0.00000000000000e+00
1071	R0001071	EQ 0.00000000000000e+00	0.00000000e+00	0.00000000e+00	0.00000000000000e+00	0.00000000000000e+00
1072	R0001072	EQ 0.00000000000000e+00	0.00000000e+00	0.00000000e+00	0.00000000000000e+00	0.00000000000000e+00
1073	R0001073	EQ 0.00000000000000e+00	0.00000000e+00	0.00000000e+00	0.00000000000000e+00	0.00000000000000e+00
1074	R0001074	EQ 0.00000000000000e+00	0.00000000e+00	0.00000000e+00	0.00000000000000e+00	0.00000000000000e+00
1075	R0001075	EQ 0.00000000000000e+00	0.00000000e+00	0.00000000e+00	0.00000000000000e+00	0.00000000000000e+00
1076	R0001076	EQ 0.00000000000000e+00	0.00000000e+00	0.00000000e+00	0.00000000000000e+00	0.00000000000000e+00
1077	R0001077	EQ 0.00000000000000e+00	0.00000000e+00	0.00000000e+00	0.00000000000000e+00	0.00000000000000e+00
1078	R0001078	EQ 0.00000000000000e+00	0.00000000e+00	0.00000000e+00	0.00000000000000e+00	0.00000000000000e+00
1079	R0001079	EQ 0.00000000000000e+00	0.00000000e+00	0.00000000e+00	0.00000000000000e+00	0.00000000000000e+00
1080	R0001080	EQ 0.00000000000000e+00	0.00000000e+00	0.00000000e+00	0.00000000000000e+00	0.00000000000000e+00
1081	R0001081	EQ 0.00000000000000e+00	0.00000000e+00	0.00000000e+00	0.00000000000000e+00	0.00000000000000e+00
1082	R0001082	EQ 0.00000000000000e+00	0.00000000e+00	0.00000000e+00	0.00000000000000e+00	0.00000000000000e+00
1083	R0001083	EQ 0.00000000000000e+00	0.00000000e+00	0.00000000e+00	0.00000000000000e+00	0.00000000000000e+00
1084	R0001084	EQ 0.00000000000000e+00	0.00000000e+00	0.00000000e+00	0.00000000000000e+00	0.00000000000000e+00
1085	R0001085	EQ 0.00000000000000e+00	0.00000000e+00	0.00000000e+00	0.00000000000000e+00	0.00000000000000e+00
1086	R0001086	EQ 0.00000000000000e+00	0.00000000e+00	0.00000000e+00	0.00000000000000e+00	0.00000000000000e+00
1087	R0001087	EQ 0.00000000000000e+00	0.00000000e+00	0.00000000e+00	0.00000000000000e+00	0.00000000000000e+00
1088	R0001088	EQ 0.00000000000000e+00	0.00000000e+00	0.00000000e+00	0.00000000000000e+00	0.00000000000000e+00
1089	R0001089	EQ 0.00000000000000e+00	0.00000000e+00	0.00000000e+00	0.00000000000000e+00	0.00000000000000e+00
1090	R0001090	EQ 0.00000000000000e+00	0.00000000e+00	0.00000000e+00	0.00000000000000e+00	0.00000000000000e+00
1091	R0001091	EQ 0.00000000000000e+00	0.00000000e+00	0.00000000e+00	0.00000000000000e+00	0.00000000000000e+00
1092	R0001092	EQ 0.00000000000000e+00	0.00000000e+00	0.00000000e+00	0.00000000000000e+00	0.00000000000000e+00

1093	R0001093	EQ	0.00000000000000e+00	0.00000000e+00	0.00000000e+00	0.00000000000000e+00	0.00000000000000e+00
1094	R0001094	EQ	0.00000000000000e+00	0.00000000e+00	0.00000000e+00	0.00000000000000e+00	0.00000000000000e+00
1095	R0001095	EQ	0.00000000000000e+00	0.00000000e+00	0.00000000e+00	0.00000000000000e+00	0.00000000000000e+00
1096	R0001096	EQ	0.00000000000000e+00	0.00000000e+00	0.00000000e+00	0.00000000000000e+00	0.00000000000000e+00
1097	R0001097	EQ	0.00000000000000e+00	0.00000000e+00	0.00000000e+00	0.00000000000000e+00	0.00000000000000e+00
1098	R0001098	EQ	0.00000000000000e+00	0.00000000e+00	0.00000000e+00	0.00000000000000e+00	0.00000000000000e+00
1099	R0001099	EQ	0.00000000000000e+00	0.00000000e+00	0.00000000e+00	0.00000000000000e+00	0.00000000000000e+00
1100	R0001100	EQ	0.00000000000000e+00	0.00000000e+00	0.00000000e+00	0.00000000000000e+00	0.00000000000000e+00
1101	R0001101	EQ	0.00000000000000e+00	0.00000000e+00	0.00000000e+00	0.00000000000000e+00	0.00000000000000e+00
1102	R0001102	EQ	0.00000000000000e+00	0.00000000e+00	0.00000000e+00	0.00000000000000e+00	0.00000000000000e+00
1103	R0001103	EQ	0.00000000000000e+00	0.00000000e+00	0.00000000e+00	0.00000000000000e+00	0.00000000000000e+00
1104	R0001104	EQ	0.00000000000000e+00	0.00000000e+00	0.00000000e+00	0.00000000000000e+00	0.00000000000000e+00
1105	R0001105	EQ	0.00000000000000e+00	0.00000000e+00	0.00000000e+00	0.00000000000000e+00	0.00000000000000e+00
1106	R0001106	EQ	0.00000000000000e+00	0.00000000e+00	0.00000000e+00	0.00000000000000e+00	0.00000000000000e+00
1107	R0001107	EQ	0.00000000000000e+00	0.00000000e+00	0.00000000e+00	0.00000000000000e+00	0.00000000000000e+00
1108	R0001108	EQ	0.00000000000000e+00	0.00000000e+00	0.00000000e+00	0.00000000000000e+00	0.00000000000000e+00
1109	R0001109	EQ	0.00000000000000e+00	0.00000000e+00	0.00000000e+00	0.00000000000000e+00	0.00000000000000e+00
1110	R0001110	EQ	0.00000000000000e+00	0.00000000e+00	0.00000000e+00	0.00000000000000e+00	0.00000000000000e+00
1111	R0001111	EQ	0.00000000000000e+00	0.00000000e+00	0.00000000e+00	0.00000000000000e+00	0.00000000000000e+00
1112	R0001112	EQ	0.00000000000000e+00	0.00000000e+00	0.00000000e+00	0.00000000000000e+00	0.00000000000000e+00
1113	R0001113	EQ	0.00000000000000e+00	0.00000000e+00	0.00000000e+00	0.00000000000000e+00	0.00000000000000e+00
1114	R0001114	EQ	0.00000000000000e+00	0.00000000e+00	0.00000000e+00	0.00000000000000e+00	0.00000000000000e+00
1115	R0001115	EQ	0.00000000000000e+00	0.00000000e+00	0.00000000e+00	0.00000000000000e+00	0.00000000000000e+00
1116	R0001116	EQ	0.00000000000000e+00	0.00000000e+00	0.00000000e+00	0.00000000000000e+00	0.00000000000000e+00
1117	R0001117	EQ	0.00000000000000e+00	0.00000000e+00	0.00000000e+00	0.00000000000000e+00	0.00000000000000e+00
1118	R0001118	EQ	0.00000000000000e+00	0.00000000e+00	0.00000000e+00	0.00000000000000e+00	0.00000000000000e+00
1119	R0001119	EQ	0.00000000000000e+00	0.00000000e+00	0.00000000e+00	0.00000000000000e+00	0.00000000000000e+00
1120	R0001120	EQ	0.00000000000000e+00	0.00000000e+00	0.00000000e+00	0.00000000000000e+00	0.00000000000000e+00
1121	R0001121	EQ	0.00000000000000e+00	0.00000000e+00	0.00000000e+00	0.00000000000000e+00	0.00000000000000e+00
1122	R0001122	EQ	0.00000000000000e+00	0.00000000e+00	0.00000000e+00	0.00000000000000e+00	0.00000000000000e+00
1123	R0001123	EQ	0.00000000000000e+00	0.00000000e+00	0.00000000e+00	0.00000000000000e+00	0.00000000000000e+00
1124	R0001124	EQ	0.00000000000000e+00	0.00000000e+00	0.00000000e+00	0.00000000000000e+00	0.00000000000000e+00
1125	R0001125	EQ	0.00000000000000e+00	0.00000000e+00	0.00000000e+00	0.00000000000000e+00	0.00000000000000e+00
1126	R0001126	EQ	0.00000000000000e+00	0.00000000e+00	0.00000000e+00	0.00000000000000e+00	0.00000000000000e+00
1127	R0001127	EQ	0.00000000000000e+00	0.00000000e+00	0.00000000e+00	0.00000000000000e+00	0.00000000000000e+00
1128	R0001128	EQ	0.00000000000000e+00	0.00000000e+00	0.00000000e+00	0.00000000000000e+00	0.00000000000000e+00
1129	R0001129	EQ	0.00000000000000e+00	0.00000000e+00	0.00000000e+00	0.00000000000000e+00	0.00000000000000e+00
1130	R0001130	EQ	0.00000000000000e+00	0.00000000e+00	0.00000000e+00	0.00000000000000e+00	0.00000000000000e+00
1131	R0001131	EQ	0.00000000000000e+00	0.00000000e+00	0.00000000e+00	0.00000000000000e+00	0.00000000000000e+00
1132	R0001132	EQ	0.00000000000000e+00	0.00000000e+00	0.00000000e+00	0.00000000000000e+00	0.00000000000000e+00
1133	R0001133	EQ	0.00000000000000e+00	0.00000000e+00	0.00000000e+00	0.00000000000000e+00	0.00000000000000e+00
1134	R0001134	EQ	0.00000000000000e+00	0.00000000e+00	0.00000000e+00	0.00000000000000e+00	0.00000000000000e+00
1135	R0001135	EQ	0.00000000000000e+00	0.00000000e+00	0.00000000e+00	0.00000000000000e+00	0.00000000000000e+00
1136	R0001136	EQ	0.00000000000000e+00	0.00000000e+00	0.00000000e+00	0.00000000000000e+00	0.00000000000000e+00
1137	R0001137	EQ	0.00000000000000e+00	0.00000000e+00	0.00000000e+00	0.00000000000000e+00	0.00000000000000e+00
1138	R0001138	EQ	0.00000000000000e+00	0.00000000e+00	0.00000000e+00	0.00000000000000e+00	0.00000000000000e+00
1139	R0001139	EQ	0.00000000000000e+00	0.00000000e+00	0.00000000e+00	0.00000000000000e+00	0.00000000000000e+00
1140	R0001140	EQ	0.00000000000000e+00	0.00000000e+00	0.00000000e+00	0.00000000000000e+00	0.00000000000000e+00
1141	R0001141	EQ	0.00000000000000e+00	0.00000000e+00	0.00000000e+00	0.00000000000000e+00	0.00000000000000e+00
1142	R0001142	EQ	0.00000000000000e+00	0.00000000e+00	0.00000000e+00	0.00000000000000e+00	0.00000000000000e+00
1143	R0001143	EQ	0.00000000000000e+00	0.00000000e+00	0.00000000e+00	0.00000000000000e+00	0.00000000000000e+00
1144	R0001144	EQ	0.00000000000000e+00	0.00000000e+00	0.00000000e+00	0.00000000000000e+00	0.00000000000000e+00
1145	R0001145	EQ	0.00000000000000e+00	0.00000000e+00	0.00000000e+00	0.00000000000000e+00	0.00000000000000e+00
1146	R0001146	EQ	0.00000000000000e+00	0.00000000e+00	0.00000000e+00	0.00000000000000e+00	0.00000000000000e+00
1147	R0001147	EQ	0.00000000000000e+00	0.00000000e+00	0.00000000e+00	0.00000000000000e+00	0.00000000000000e+00
1148	R0001148	EQ	0.00000000000000e+00	0.00000000e+00	0.00000000e+00	0.00000000000000e+00	0.00000000000000e+00
1149	R0001149	EQ	0.00000000000000e+00	0.00000000e+00	0.00000000e+00	0.00000000000000e+00	0.00000000000000e+00
1150	R0001150	EQ	0.00000000000000e+00	0.00000000e+00	0.00000000e+00	0.00000000000000e+00	0.00000000000000e+00
1151	R0001151	EQ	0.00000000000000e+00	0.00000000e+00	0.00000000e+00	0.00000000000000e+00	0.00000000000000e+00
1152	R0001152	EQ	0.00000000000000e+00	0.00000000e+00	0.00000000e+00	0.00000000000000e+00	0.00000000000000e+00
1153	R0001153	EQ	0.00000000000000e+00	0.00000000e+00	0.00000000e+00	0.00000000000000e+00	0.00000000000000e+00
1154	R0001154	EQ	0.00000000000000e+00	0.00000000e+00	0.00000000e+00	0.00000000000000e+00	0.00000000000000e+00
1155	R0001155	EQ	0.00000000000000e+00	0.00000000e+00	0.00000000e+00	0.00000000000000e+00	0.00000000000000e+00
1156	R0001156	EQ	0.00000000000000e+00	0.00000000e+00	0.00000000e+00	0.00000000000000e+00	0.00000000000000e+00
1157	R0001157	EQ	0.00000000000000e+00	0.00000000e+00	0.00000000e+00	0.00000000000000e+00	0.00000000000000e+00
1158	R0001158	EQ	0.00000000000000e+00	0.00000000e+00	0.00000000e+00	0.00000000000000e+00	0.00000000000000e+00
1159	R0001159	EQ	0.00000000000000e+00	0.00000000e+00	0.00000000e+00	0.00000000000000e+00	0.00000000000000e+00
1160	R0001160	EQ	0.00000000000000e+00	0.00000000e+00	0.00000000e+00	0.00000000000000e+00	0.00000000000000e+00
1161	R0001161	EQ	0.00000000000000e+00	0.00000000e+00	0.00000000e+00	0.00000000000000e+00	0.00000000000000e+00
1162	R0001162	EQ	0.00000000000000e+00	0.00000000e+00	0.00000000e+00	0.00000000000000e+00	0.00000000000000e+00
1163	R0001163	EQ	0.00000000000000e+00	0.00000000e+00	0.00000000e+00	0.00000000000000e+00	0.00000000000000e+00
1164	R0001164	EQ	0.00000000000000e+00	0.00000000e+00	0.00000000e+00	0.00000000000000e+00	0.00000000000000e+00
1165	R0001165	EQ	0.00000000000000e+00	0.00000000e+00	0.00000000e+00	0.00000000000000e+00	0.00000000000000e+00
1166	R0001166	EQ	0.00000000000000e+00	0.00000000e+00	0.00000000e+00	0.00000000000000e+00	0.00000000000000e+00
1167	R0001167	EQ	0.00000000000000e+00	0.00000000e+00	0.00000000e+00	0.00000000000000e+00	0.00000000000000e+00
1168	R0001168	EQ	0.00000000000000e+00	0.00000000e+00	0.00000000e+00	0.00000000000000e+00	0.00000000000000e+00
1169	R0001169	EQ	0.00000000000000e+00	0.00000000e+00	0.00000000e+00	0.00000000000000e+00	0.00000000000000e+00
1170	R0001170	EQ	0.00000000000000e+00	0.00000000e+00	0.00000000e+00	0.00000000000000e+00	0.00000000000000e+00
1171	R0001171	EQ	0.00000000000000e+00	0.00000000e+00	0.00000000e+00	0.00000000000000e+00	0.00000000000000e+00
1172	R0001172	EQ	0.00000000000000e+00	0.00000000e+00	0.00000000e+00	0.00000000000000e+00	0.00000000000000e+00
1173	R0001173	EQ	0.00000000000000e+00	0.00000000e+00	0.00000000e+00	0.00000000000000e+00	0.00000000000000e+00
1174	R0001174	EQ	0.00000000000000e+00	0.00000000e+00	0.00000000e+00	0.00000000000000e+00	0.00000000000000e+00
1175	R0001175	EQ	0.00000000000000e+00	0.00000000e+00	0.00000000e+00	0.00000000000000e+00	0.00000000000000e+00
1176	R0001176	EQ	0.00000000000000e+00	0.00000000e+00	0.00000000e+00	0.00000000000000e+00	0.00000000000000e+00
1177	R0001177	EQ	0.00000000000000e+00	0.00000000e+00	0.00000000e+00	0.00000000000000e+00	0.00000000000000e+00
1178	R0001178	EQ	0.00000000000000e+00	0.00000000e+00	0.00000000e+00	0.00000000000000e+00	0.00000000000000e+00
1179	R0001179	EQ	0.00000000000000e+00	0.00000000e+00	0.00000000e+00	0.00000000000000e+00	0.00000000000000e+00
1180	R0001180	EQ	0.00000000000000e+00	0.00000000e+00	0.00000000e+00	0.00000000000000e+00	0.00000000000000e+00
1181	R0001181	EQ	0.00000000000000e+00	0.00000000e+00	0.00000000e+00	0.00000000000000e+00	0.00000000000000e+00
1182	R0001182	EQ	0.00000000000000e+00	0.00000000e+00	0.00000000e+00	0.00000000000000e+00	0.00000000000000e+00
1183	R0001183	EQ	0.00000000000000e+00	0.00000000e+00	0.00000000e+00	0.00000000000000e+00	0.00000000000000e+00
1184	R0001184	EQ	0.00000000000000e+00	0.00000000e+00	0.00000000e+00	0.00000000000000e+00	0.00000000000000e+00
1185	R0001185	EQ	0.00000000000000e+00	0.00000000e+00	0.00000000e+00	0.00000000000000e+00	0.00000000000000e+00
1186	R0001186	EQ	0.00000000000000e+00	0.00000000e+00	0.00000000e+00	0.00000000000000e+00	0.00000000000000e+00
1187	R0001187	EQ	0.00000000000000e+00	0.00000000e+00	0.00000000e+00	0.00000000000000e+00	0.00000000000000e+00
1188	R0001188	EQ	0.00000000000000e+00	0.00000000e+00	0.00000000e+00	0.00000000000000e+00	0.00000000000000e+00
1189	R0001189	EQ	0.00000000000000e+00	0.00000000e+00	0.00000000e+00	0.00000000000000e+00	0.00000000000000e+00
1190	R0001190	EQ	0.00000000000000e+00	0.00000000e+00	0.00000000e+00	0.00000000000000e+00	0.00000000000000e+00

1191	R0001191	EQ 0.00000000000000e+00	0.00000000e+00	0.00000000e+00	0.00000000000000e+00	0.00000000000000e+00
1192	R0001192	EQ 0.00000000000000e+00	0.00000000e+00	0.00000000e+00	0.00000000000000e+00	0.00000000000000e+00
1193	R0001193	EQ 0.00000000000000e+00	0.00000000e+00	0.00000000e+00	0.00000000000000e+00	0.00000000000000e+00
1194	R0001194	EQ 0.00000000000000e+00	0.00000000e+00	0.00000000e+00	0.00000000000000e+00	0.00000000000000e+00
1195	R0001195	EQ 0.00000000000000e+00	0.00000000e+00	0.00000000e+00	0.00000000000000e+00	0.00000000000000e+00
1196	R0001196	EQ 0.00000000000000e+00	0.00000000e+00	0.00000000e+00	0.00000000000000e+00	0.00000000000000e+00
1197	R0001197	EQ 0.00000000000000e+00	0.00000000e+00	0.00000000e+00	0.00000000000000e+00	0.00000000000000e+00
1198	R0001198	EQ 0.00000000000000e+00	0.00000000e+00	0.00000000e+00	0.00000000000000e+00	0.00000000000000e+00
1199	R0001199	EQ 0.00000000000000e+00	0.00000000e+00	0.00000000e+00	0.00000000000000e+00	0.00000000000000e+00
1200	R0001200	EQ 0.00000000000000e+00	0.00000000e+00	0.00000000e+00	0.00000000000000e+00	0.00000000000000e+00
1201	R0001201	EQ 0.00000000000000e+00	0.00000000e+00	0.00000000e+00	0.00000000000000e+00	0.00000000000000e+00
1202	R0001202	EQ 0.00000000000000e+00	0.00000000e+00	0.00000000e+00	0.00000000000000e+00	0.00000000000000e+00
1203	R0001203	EQ 0.00000000000000e+00	0.00000000e+00	0.00000000e+00	0.00000000000000e+00	0.00000000000000e+00
1204	R0001204	EQ 0.00000000000000e+00	0.00000000e+00	0.00000000e+00	0.00000000000000e+00	0.00000000000000e+00
1205	R0001205	EQ 0.00000000000000e+00	0.00000000e+00	0.00000000e+00	0.00000000000000e+00	0.00000000000000e+00
1206	R0001206	EQ 0.00000000000000e+00	0.00000000e+00	0.00000000e+00	0.00000000000000e+00	0.00000000000000e+00
1207	R0001207	EQ 0.00000000000000e+00	0.00000000e+00	0.00000000e+00	0.00000000000000e+00	0.00000000000000e+00
1208	R0001208	EQ 0.00000000000000e+00	0.00000000e+00	0.00000000e+00	0.00000000000000e+00	0.00000000000000e+00
1209	R0001209	EQ 0.00000000000000e+00	0.00000000e+00	0.00000000e+00	0.00000000000000e+00	0.00000000000000e+00
1210	R0001210	EQ 0.00000000000000e+00	0.00000000e+00	0.00000000e+00	0.00000000000000e+00	0.00000000000000e+00
1211	R0001211	EQ 0.00000000000000e+00	0.00000000e+00	0.00000000e+00	0.00000000000000e+00	0.00000000000000e+00
1212	R0001212	EQ 0.00000000000000e+00	0.00000000e+00	0.00000000e+00	0.00000000000000e+00	0.00000000000000e+00
1213	R0001213	EQ 0.00000000000000e+00	0.00000000e+00	0.00000000e+00	0.00000000000000e+00	0.00000000000000e+00
1214	R0001214	EQ 0.00000000000000e+00	0.00000000e+00	0.00000000e+00	0.00000000000000e+00	0.00000000000000e+00
1215	R0001215	EQ 0.00000000000000e+00	0.00000000e+00	0.00000000e+00	0.00000000000000e+00	0.00000000000000e+00
1216	R0001216	EQ 0.00000000000000e+00	0.00000000e+00	0.00000000e+00	0.00000000000000e+00	0.00000000000000e+00
1217	R0001217	EQ 0.00000000000000e+00	0.00000000e+00	0.00000000e+00	0.00000000000000e+00	0.00000000000000e+00
1218	R0001218	EQ 0.00000000000000e+00	0.00000000e+00	0.00000000e+00	0.00000000000000e+00	0.00000000000000e+00
1219	R0001219	EQ 0.00000000000000e+00	0.00000000e+00	0.00000000e+00	0.00000000000000e+00	0.00000000000000e+00
1220	R0001220	EQ 0.00000000000000e+00	0.00000000e+00	0.00000000e+00	0.00000000000000e+00	0.00000000000000e+00
1221	R0001221	EQ 0.00000000000000e+00	0.00000000e+00	0.00000000e+00	0.00000000000000e+00	0.00000000000000e+00
1222	R0001222	EQ 0.00000000000000e+00	0.00000000e+00	0.00000000e+00	0.00000000000000e+00	0.00000000000000e+00
1223	R0001223	EQ 0.00000000000000e+00	0.00000000e+00	0.00000000e+00	0.00000000000000e+00	0.00000000000000e+00
1224	R0001224	EQ 0.00000000000000e+00	0.00000000e+00	0.00000000e+00	0.00000000000000e+00	0.00000000000000e+00
1225	R0001225	EQ 0.00000000000000e+00	0.00000000e+00	0.00000000e+00	0.00000000000000e+00	0.00000000000000e+00
1226	R0001226	EQ 0.00000000000000e+00	0.00000000e+00	0.00000000e+00	0.00000000000000e+00	0.00000000000000e+00
1227	R0001227	EQ 0.00000000000000e+00	0.00000000e+00	0.00000000e+00	0.00000000000000e+00	0.00000000000000e+00
1228	R0001228	EQ 0.00000000000000e+00	0.00000000e+00	0.00000000e+00	0.00000000000000e+00	0.00000000000000e+00
1229	R0001229	EQ 0.00000000000000e+00	0.00000000e+00	0.00000000e+00	0.00000000000000e+00	0.00000000000000e+00
1230	R0001230	EQ 0.00000000000000e+00	0.00000000e+00	0.00000000e+00	0.00000000000000e+00	0.00000000000000e+00
1231	R0001231	EQ 0.00000000000000e+00	0.00000000e+00	0.00000000e+00	0.00000000000000e+00	0.00000000000000e+00
1232	R0001232	EQ 0.00000000000000e+00	0.00000000e+00	0.00000000e+00	0.00000000000000e+00	0.00000000000000e+00
1233	R0001233	EQ 0.00000000000000e+00	0.00000000e+00	0.00000000e+00	0.00000000000000e+00	0.00000000000000e+00
1234	R0001234	EQ 0.00000000000000e+00	0.00000000e+00	0.00000000e+00	0.00000000000000e+00	0.00000000000000e+00
1235	R0001235	EQ 0.00000000000000e+00	0.00000000e+00	0.00000000e+00	0.00000000000000e+00	0.00000000000000e+00
1236	R0001236	EQ 0.00000000000000e+00	0.00000000e+00	0.00000000e+00	0.00000000000000e+00	0.00000000000000e+00
1237	R0001237	EQ 0.00000000000000e+00	0.00000000e+00	0.00000000e+00	0.00000000000000e+00	0.00000000000000e+00
1238	R0001238	EQ 0.00000000000000e+00	0.00000000e+00	0.00000000e+00	0.00000000000000e+00	0.00000000000000e+00
1239	R0001239	EQ 0.00000000000000e+00	0.00000000e+00	0.00000000e+00	0.00000000000000e+00	0.00000000000000e+00
1240	R0001240	EQ 0.00000000000000e+00	0.00000000e+00	0.00000000e+00	0.00000000000000e+00	0.00000000000000e+00
1241	R0001241	EQ 0.00000000000000e+00	0.00000000e+00	0.00000000e+00	0.00000000000000e+00	0.00000000000000e+00
1242	R0001242	EQ 0.00000000000000e+00	0.00000000e+00	0.00000000e+00	0.00000000000000e+00	0.00000000000000e+00
1243	R0001243	EQ 0.00000000000000e+00	0.00000000e+00	0.00000000e+00	0.00000000000000e+00	0.00000000000000e+00
1244	R0001244	EQ 0.00000000000000e+00	0.00000000e+00	0.00000000e+00	0.00000000000000e+00	0.00000000000000e+00
1245	R0001245	EQ 0.00000000000000e+00	0.00000000e+00	0.00000000e+00	0.00000000000000e+00	0.00000000000000e+00
1246	R0001246	EQ 0.00000000000000e+00	0.00000000e+00	0.00000000e+00	0.00000000000000e+00	0.00000000000000e+00
1247	R0001247	EQ 0.00000000000000e+00	0.00000000e+00	0.00000000e+00	0.00000000000000e+00	0.00000000000000e+00
1248	R0001248	EQ 0.00000000000000e+00	0.00000000e+00	0.00000000e+00	0.00000000000000e+00	0.00000000000000e+00
1249	R0001249	EQ 0.00000000000000e+00	0.00000000e+00	0.00000000e+00	0.00000000000000e+00	0.00000000000000e+00
1250	R0001250	EQ 0.00000000000000e+00	0.00000000e+00	0.00000000e+00	0.00000000000000e+00	0.00000000000000e+00
1251	R0001251	EQ 0.00000000000000e+00	0.00000000e+00	0.00000000e+00	0.00000000000000e+00	0.00000000000000e+00
1252	R0001252	EQ 0.00000000000000e+00	0.00000000e+00	0.00000000e+00	0.00000000000000e+00	0.00000000000000e+00
1253	R0001253	EQ 0.00000000000000e+00	0.00000000e+00	0.00000000e+00	0.00000000000000e+00	0.00000000000000e+00
1254	R0001254	EQ 0.00000000000000e+00	0.00000000e+00	0.00000000e+00	0.00000000000000e+00	0.00000000000000e+00
1255	R0001255	EQ 0.00000000000000e+00	0.00000000e+00	0.00000000e+00	0.00000000000000e+00	0.00000000000000e+00
1256	R0001256	EQ 0.00000000000000e+00	0.00000000e+00	0.00000000e+00	0.00000000000000e+00	0.00000000000000e+00
1257	R0001257	EQ 0.00000000000000e+00	0.00000000e+00	0.00000000e+00	0.00000000000000e+00	0.00000000000000e+00
1258	R0001258	EQ 0.00000000000000e+00	0.00000000e+00	0.00000000e+00	0.00000000000000e+00	0.00000000000000e+00
1259	R0001259	EQ 0.00000000000000e+00	0.00000000e+00	0.00000000e+00	0.00000000000000e+00	0.00000000000000e+00
1260	R0001260	EQ 0.00000000000000e+00	0.00000000e+00	0.00000000e+00	0.00000000000000e+00	0.00000000000000e+00
1261	R0001261	EQ 0.00000000000000e+00	0.00000000e+00	0.00000000e+00	0.00000000000000e+00	0.00000000000000e+00
1262	R0001262	EQ 0.00000000000000e+00	0.00000000e+00	0.00000000e+00	0.00000000000000e+00	0.00000000000000e+00
1263	R0001263	EQ 0.00000000000000e+00	0.00000000e+00	0.00000000e+00	0.00000000000000e+00	0.00000000000000e+00
1264	R0001264	EQ 0.00000000000000e+00	0.00000000e+00	0.00000000e+00	0.00000000000000e+00	0.00000000000000e+00
1265	R0001265	EQ 0.00000000000000e+00	0.00000000e+00	0.00000000e+00	0.00000000000000e+00	0.00000000000000e+00
1266	R0001266	EQ 0.00000000000000e+00	0.00000000e+00	0.00000000e+00	0.00000000000000e+00	0.00000000000000e+00
1267	R0001267	EQ 0.00000000000000e+00	0.00000000e+00	0.00000000e+00	0.00000000000000e+00	0.00000000000000e+00
1268	R0001268	EQ 0.00000000000000e+00	0.00000000e+00	0.00000000e+00	0.00000000000000e+00	0.00000000000000e+00
1269	R0001269	EQ 0.00000000000000e+00	0.00000000e+00	0.00000000e+00	0.00000000000000e+00	0.00000000000000e+00
1270	R0001270	EQ 0.00000000000000e+00	0.00000000e+00	0.00000000e+00	0.00000000000000e+00	0.00000000000000e+00
1271	R0001271	EQ 0.00000000000000e+00	0.00000000e+00	0.00000000e+00	0.00000000000000e+00	0.00000000000000e+00
1272	R0001272	EQ 0.00000000000000e+00	0.00000000e+00	0.00000000e+00	0.00000000000000e+00	0.00000000000000e+00
1273	R0001273	EQ 0.00000000000000e+00	0.00000000e+00	0.00000000e+00	0.00000000000000e+00	0.00000000000000e+00
1274	R0001274	EQ 0.00000000000000e+00	0.00000000e+00	0.00000000e+00	0.00000000000000e+00	0.00000000000000e+00
1275	R0001275	EQ 0.00000000000000e+00	0.00000000e+00	0.00000000e+00	0.00000000000000e+00	0.00000000000000e+00
1276	R0001276	EQ 0.00000000000000e+00	0.00000000e+00	0.00000000e+00	0.00000000000000e+00	0.00000000000000e+00
1277	R0001277	EQ 0.00000000000000e+00	0.00000000e+00	0.00000000e+00	0.00000000000000e+00	0.00000000000000e+00
1278	R0001278	EQ 0.00000000000000e+00	0.00000000e+00	0.00000000e+00	0.00000000000000e+00	0.00000000000000e+00
1279	R0001279	EQ 0.00000000000000e+00	0.00000000e+00	0.00000000e+00	0.00000000000000e+00	0.00000000000000e+00
1280	R0001280	EQ 0.00000000000000e+00	0.00000000e+00	0.00000000e+00	0.00000000000000e+00	0.00000000000000e+00
1281	R0001281	EQ 0.00000000000000e+00	0.00000000e+00	0.00000000e+00	0.00000000000000e+00	0.00000000000000e+00
1282	R0001282	EQ 0.00000000000000e+00	0.00000000e+00	0.00000000e+00	0.00000000000000e+00	0.00000000000000e+00
1283	R0001283	EQ 0.00000000000000e+00	0.00000000e+00	0.00000000e+00	0.00000000000000e+00	0.00000000000000e+00
1284	R0001284	EQ 0.00000000000000e+00	0.00000000e+00	0.00000000e+00	0.00000000000000e+00	0.00000000000000e+00
1285	R0001285	EQ 0.00000000000000e+00	0.00000000e+00	0.00000000e+00	0.00000000000000e+00	0.00000000000000e+00
1286	R0001286	EQ 0.00000000000000e+00	0.00000000e+00	0.00000000e+00	0.00000000000000e+00	0.00000000000000e+00
1287	R0001287	EQ 0.00000000000000e+00	0.00000000e+00	0.00000000e+00	0.00000000000000e+00	0.00000000000000e+00
1288	R0001288	EQ 0.00000000000000e+00	0.00000000e+00	0.00000000e+00	0.00000000000000e+00	0.00000000000000e+00

1289	R0001289	EQ	0.00000000000000e+00	0.00000000e+00	0.00000000e+00	0.00000000000000e+00	0.00000000000000e+00
1290	R0001290	EQ	0.00000000000000e+00	0.00000000e+00	0.00000000e+00	0.00000000000000e+00	0.00000000000000e+00
1291	R0001291	EQ	0.00000000000000e+00	0.00000000e+00	0.00000000e+00	0.00000000000000e+00	0.00000000000000e+00
1292	R0001292	EQ	0.00000000000000e+00	0.00000000e+00	0.00000000e+00	0.00000000000000e+00	0.00000000000000e+00
1293	R0001293	EQ	0.00000000000000e+00	0.00000000e+00	0.00000000e+00	0.00000000000000e+00	0.00000000000000e+00
1294	R0001294	EQ	0.00000000000000e+00	0.00000000e+00	0.00000000e+00	0.00000000000000e+00	0.00000000000000e+00
1295	R0001295	EQ	0.00000000000000e+00	0.00000000e+00	0.00000000e+00	0.00000000000000e+00	0.00000000000000e+00
1296	R0001296	EQ	0.00000000000000e+00	0.00000000e+00	0.00000000e+00	0.00000000000000e+00	0.00000000000000e+00
1297	R0001297	EQ	0.00000000000000e+00	0.00000000e+00	0.00000000e+00	0.00000000000000e+00	0.00000000000000e+00
1298	R0001298	EQ	0.00000000000000e+00	0.00000000e+00	0.00000000e+00	0.00000000000000e+00	0.00000000000000e+00
1299	R0001299	EQ	0.00000000000000e+00	0.00000000e+00	0.00000000e+00	0.00000000000000e+00	0.00000000000000e+00
1300	R0001300	EQ	0.00000000000000e+00	0.00000000e+00	0.00000000e+00	0.00000000000000e+00	0.00000000000000e+00
1301	R0001301	EQ	0.00000000000000e+00	0.00000000e+00	0.00000000e+00	0.00000000000000e+00	0.00000000000000e+00
1302	R0001302	EQ	0.00000000000000e+00	0.00000000e+00	0.00000000e+00	0.00000000000000e+00	0.00000000000000e+00
1303	R0001303	EQ	0.00000000000000e+00	0.00000000e+00	0.00000000e+00	0.00000000000000e+00	0.00000000000000e+00
1304	R0001304	EQ	0.00000000000000e+00	0.00000000e+00	0.00000000e+00	0.00000000000000e+00	0.00000000000000e+00
1305	R0001305	EQ	0.00000000000000e+00	0.00000000e+00	0.00000000e+00	0.00000000000000e+00	0.00000000000000e+00
1306	R0001306	EQ	0.00000000000000e+00	0.00000000e+00	0.00000000e+00	0.00000000000000e+00	0.00000000000000e+00
1307	R0001307	EQ	0.00000000000000e+00	0.00000000e+00	0.00000000e+00	0.00000000000000e+00	0.00000000000000e+00
1308	R0001308	EQ	0.00000000000000e+00	0.00000000e+00	0.00000000e+00	0.00000000000000e+00	0.00000000000000e+00
1309	R0001309	EQ	0.00000000000000e+00	0.00000000e+00	0.00000000e+00	0.00000000000000e+00	0.00000000000000e+00
1310	R0001310	EQ	0.00000000000000e+00	0.00000000e+00	0.00000000e+00	0.00000000000000e+00	0.00000000000000e+00
1311	R0001311	EQ	0.00000000000000e+00	0.00000000e+00	0.00000000e+00	0.00000000000000e+00	0.00000000000000e+00
1312	R0001312	EQ	0.00000000000000e+00	0.00000000e+00	0.00000000e+00	0.00000000000000e+00	0.00000000000000e+00
1313	R0001313	EQ	0.00000000000000e+00	0.00000000e+00	0.00000000e+00	0.00000000000000e+00	0.00000000000000e+00
1314	R0001314	EQ	0.00000000000000e+00	0.00000000e+00	0.00000000e+00	0.00000000000000e+00	0.00000000000000e+00
1315	R0001315	EQ	0.00000000000000e+00	0.00000000e+00	0.00000000e+00	0.00000000000000e+00	0.00000000000000e+00
1316	R0001316	EQ	0.00000000000000e+00	0.00000000e+00	0.00000000e+00	0.00000000000000e+00	0.00000000000000e+00
1317	R0001317	EQ	0.00000000000000e+00	0.00000000e+00	0.00000000e+00	0.00000000000000e+00	0.00000000000000e+00
1318	R0001318	EQ	0.00000000000000e+00	0.00000000e+00	0.00000000e+00	0.00000000000000e+00	0.00000000000000e+00
1319	R0001319	EQ	0.00000000000000e+00	0.00000000e+00	0.00000000e+00	0.00000000000000e+00	0.00000000000000e+00
1320	R0001320	EQ	0.00000000000000e+00	0.00000000e+00	0.00000000e+00	0.00000000000000e+00	0.00000000000000e+00
1321	R0001321	EQ	0.00000000000000e+00	0.00000000e+00	0.00000000e+00	0.00000000000000e+00	0.00000000000000e+00
1322	R0001322	EQ	0.00000000000000e+00	0.00000000e+00	0.00000000e+00	0.00000000000000e+00	0.00000000000000e+00
1323	R0001323	EQ	0.00000000000000e+00	0.00000000e+00	0.00000000e+00	0.00000000000000e+00	0.00000000000000e+00
1324	R0001324	EQ	0.00000000000000e+00	0.00000000e+00	0.00000000e+00	0.00000000000000e+00	0.00000000000000e+00
1325	R0001325	EQ	0.00000000000000e+00	0.00000000e+00	0.00000000e+00	0.00000000000000e+00	0.00000000000000e+00
1326	R0001326	EQ	0.00000000000000e+00	0.00000000e+00	0.00000000e+00	0.00000000000000e+00	0.00000000000000e+00
1327	R0001327	EQ	0.00000000000000e+00	0.00000000e+00	0.00000000e+00	0.00000000000000e+00	0.00000000000000e+00
1328	R0001328	EQ	0.00000000000000e+00	0.00000000e+00	0.00000000e+00	0.00000000000000e+00	0.00000000000000e+00
1329	R0001329	EQ	0.00000000000000e+00	0.00000000e+00	0.00000000e+00	0.00000000000000e+00	0.00000000000000e+00
1330	R0001330	EQ	0.00000000000000e+00	0.00000000e+00	0.00000000e+00	0.00000000000000e+00	0.00000000000000e+00
1331	R0001331	EQ	0.00000000000000e+00	0.00000000e+00	0.00000000e+00	0.00000000000000e+00	0.00000000000000e+00
1332	R0001332	EQ	0.00000000000000e+00	0.00000000e+00	0.00000000e+00	0.00000000000000e+00	0.00000000000000e+00
1333	R0001333	EQ	0.00000000000000e+00	0.00000000e+00	0.00000000e+00	0.00000000000000e+00	0.00000000000000e+00
1334	R0001334	EQ	0.00000000000000e+00	0.00000000e+00	0.00000000e+00	0.00000000000000e+00	0.00000000000000e+00
1335	R0001335	EQ	0.00000000000000e+00	0.00000000e+00	0.00000000e+00	0.00000000000000e+00	0.00000000000000e+00
1336	R0001336	EQ	0.00000000000000e+00	0.00000000e+00	0.00000000e+00	0.00000000000000e+00	0.00000000000000e+00
1337	R0001337	EQ	0.00000000000000e+00	0.00000000e+00	0.00000000e+00	0.00000000000000e+00	0.00000000000000e+00
1338	R0001338	EQ	0.00000000000000e+00	0.00000000e+00	0.00000000e+00	0.00000000000000e+00	0.00000000000000e+00
1339	R0001339	EQ	0.00000000000000e+00	0.00000000e+00	0.00000000e+00	0.00000000000000e+00	0.00000000000000e+00
1340	R0001340	EQ	0.00000000000000e+00	0.00000000e+00	0.00000000e+00	0.00000000000000e+00	0.00000000000000e+00
1341	R0001341	EQ	0.00000000000000e+00	0.00000000e+00	0.00000000e+00	0.00000000000000e+00	0.00000000000000e+00
1342	R0001342	EQ	0.00000000000000e+00	0.00000000e+00	0.00000000e+00	0.00000000000000e+00	0.00000000000000e+00
1343	R0001343	EQ	0.00000000000000e+00	0.00000000e+00	0.00000000e+00	0.00000000000000e+00	0.00000000000000e+00
1344	R0001344	EQ	0.00000000000000e+00	0.00000000e+00	0.00000000e+00	0.00000000000000e+00	0.00000000000000e+00
1345	R0001345	EQ	0.00000000000000e+00	0.00000000e+00	0.00000000e+00	0.00000000000000e+00	0.00000000000000e+00
1346	R0001346	EQ	0.00000000000000e+00	0.00000000e+00	0.00000000e+00	0.00000000000000e+00	0.00000000000000e+00
1347	R0001347	EQ	0.00000000000000e+00	0.00000000e+00	0.00000000e+00	0.00000000000000e+00	0.00000000000000e+00
1348	R0001348	EQ	0.00000000000000e+00	0.00000000e+00	0.00000000e+00	0.00000000000000e+00	0.00000000000000e+00
1349	R0001349	EQ	0.00000000000000e+00	0.00000000e+00	0.00000000e+00	0.00000000000000e+00	0.00000000000000e+00
1350	R0001350	EQ	0.00000000000000e+00	0.00000000e+00	0.00000000e+00	0.00000000000000e+00	0.00000000000000e+00
1351	R0001351	EQ	0.00000000000000e+00	0.00000000e+00	0.00000000e+00	0.00000000000000e+00	0.00000000000000e+00
1352	R0001352	EQ	0.00000000000000e+00	0.00000000e+00	0.00000000e+00	0.00000000000000e+00	0.00000000000000e+00
1353	R0001353	EQ	0.00000000000000e+00	0.00000000e+00	0.00000000e+00	0.00000000000000e+00	0.00000000000000e+00
1354	R0001354	EQ	0.00000000000000e+00	0.00000000e+00	0.00000000e+00	0.00000000000000e+00	0.00000000000000e+00
1355	R0001355	EQ	0.00000000000000e+00	0.00000000e+00	0.00000000e+00	0.00000000000000e+00	0.00000000000000e+00
1356	R0001356	EQ	0.00000000000000e+00	0.00000000e+00	0.00000000e+00	0.00000000000000e+00	0.00000000000000e+00
1357	R0001357	EQ	0.00000000000000e+00	0.00000000e+00	0.00000000e+00	0.00000000000000e+00	0.00000000000000e+00
1358	R0001358	EQ	0.00000000000000e+00	0.00000000e+00	0.00000000e+00	0.00000000000000e+00	0.00000000000000e+00
1359	R0001359	EQ	0.00000000000000e+00	0.00000000e+00	0.00000000e+00	0.00000000000000e+00	0.00000000000000e+00
1360	R0001360	EQ	0.00000000000000e+00	0.00000000e+00	0.00000000e+00	0.00000000000000e+00	0.00000000000000e+00
1361	R0001361	EQ	0.00000000000000e+00	0.00000000e+00	0.00000000e+00	0.00000000000000e+00	0.00000000000000e+00
1362	R0001362	EQ	0.00000000000000e+00	0.00000000e+00	0.00000000e+00	0.00000000000000e+00	0.00000000000000e+00
1363	R0001363	EQ	0.00000000000000e+00	0.00000000e+00	0.00000000e+00	0.00000000000000e+00	0.00000000000000e+00
1364	R0001364	EQ	0.00000000000000e+00	0.00000000e+00	0.00000000e+00	0.00000000000000e+00	0.00000000000000e+00
1365	R0001365	EQ	0.00000000000000e+00	0.00000000e+00	0.00000000e+00	0.00000000000000e+00	0.00000000000000e+00
1366	R0001366	EQ	0.00000000000000e+00	0.00000000e+00	0.00000000e+00	0.00000000000000e+00	0.00000000000000e+00
1367	R0001367	EQ	0.00000000000000e+00	0.00000000e+00	0.00000000e+00	0.00000000000000e+00	0.00000000000000e+00
1368	R0001368	EQ	0.00000000000000e+00	0.00000000e+00	0.00000000e+00	0.00000000000000e+00	0.00000000000000e+00
1369	R0001369	EQ	0.00000000000000e+00	0.00000000e+00	0.00000000e+00	0.00000000000000e+00	0.00000000000000e+00
1370	R0001370	EQ	0.00000000000000e+00	0.00000000e+00	0.00000000e+00	0.00000000000000e+00	0.00000000000000e+00
1371	R0001371	EQ	0.00000000000000e+00	0.00000000e+00	0.00000000e+00	0.00000000000000e+00	0.00000000000000e+00
1372	R0001372	EQ	0.00000000000000e+00	0.00000000e+00	0.00000000e+00	0.00000000000000e+00	0.00000000000000e+00
1373	R0001373	EQ	0.00000000000000e+00	0.00000000e+00	0.00000000e+00	0.00000000000000e+00	0.00000000000000e+00
1374	R0001374	EQ	0.00000000000000e+00	0.00000000e+00	0.00000000e+00	0.00000000000000e+00	0.00000000000000e+00
1375	R0001375	EQ	0.00000000000000e+00	0.00000000e+00	0.00000000e+00	0.00000000000000e+00	0.00000000000000e+00
1376	R0001376	EQ	0.00000000000000e+00	0.00000000e+00	0.00000000e+00	0.00000000000000e+00	0.00000000000000e+00
1377	R0001377	EQ	0.00000000000000e+00	0.00000000e+00	0.00000000e+00	0.00000000000000e+00	0.00000000000000e+00
1378	R0001378	EQ	0.00000000000000e+00	0.00000000e+00	0.00000000e+00	0.00000000000000e+00	0.00000000000000e+00
1379	R0001379	EQ	0.00000000000000e+00	0.00000000e+00	0.00000000e+00	0.00000000000000e+00	0.00000000000000e+00
1380	R0001380	EQ	0.00000000000000e+00	0.00000000e+00	0.00000000e+00	0.00000000000000e+00	0.00000000000000e+00
1381	R0001381	EQ	0.00000000000000e+00	0.00000000e+00	0.00000000e+00	0.00000000000000e+00	0.00000000000000e+00
1382	R0001382	EQ	0.00000000000000e+00	0.00000000e+00	0.00000000e+00	0.00000000000000e+00	0.00000000000000e+00
1383	R0001383	EQ	0.00000000000000e+00	0.00000000e+00	0.00000000e+00	0.00000000000000e+00	0.00000000000000e+00
1384	R0001384	EQ	0.00000000000000e+00	0.00000000e+00	0.00000000e+00	0.00000000000000e+00	0.00000000000000e+00
1385	R0001385	EQ	0.00000000000000e+00	0.00000000e+00	0.00000000e+00	0.00000000000000e+00	0.00000000000000e+00
1386	R0001386	EQ	0.00000000000000e+00	0.00000000e+00	0.00000000e+00	0.00000000000000e+00	0.00000000000000e+00

1387	R0001387	EQ	0.00000000000000e+00	0.00000000e+00	0.00000000e+00	0.00000000000000e+00	0.00000000000000e+00
1388	R0001388	EQ	0.00000000000000e+00	0.00000000e+00	0.00000000e+00	0.00000000000000e+00	0.00000000000000e+00
1389	R0001389	EQ	0.00000000000000e+00	0.00000000e+00	0.00000000e+00	0.00000000000000e+00	0.00000000000000e+00
1390	R0001390	EQ	0.00000000000000e+00	0.00000000e+00	0.00000000e+00	0.00000000000000e+00	0.00000000000000e+00
1391	R0001391	EQ	0.00000000000000e+00	0.00000000e+00	0.00000000e+00	0.00000000000000e+00	0.00000000000000e+00
1392	R0001392	EQ	0.00000000000000e+00	0.00000000e+00	0.00000000e+00	0.00000000000000e+00	0.00000000000000e+00
1393	R0001393	EQ	0.00000000000000e+00	0.00000000e+00	0.00000000e+00	0.00000000000000e+00	0.00000000000000e+00
1394	R0001394	EQ	0.00000000000000e+00	0.00000000e+00	0.00000000e+00	0.00000000000000e+00	0.00000000000000e+00
1395	R0001395	EQ	0.00000000000000e+00	0.00000000e+00	0.00000000e+00	0.00000000000000e+00	0.00000000000000e+00
1396	R0001396	EQ	0.00000000000000e+00	0.00000000e+00	0.00000000e+00	0.00000000000000e+00	0.00000000000000e+00
1397	R0001397	EQ	0.00000000000000e+00	0.00000000e+00	0.00000000e+00	0.00000000000000e+00	0.00000000000000e+00
1398	R0001398	EQ	0.00000000000000e+00	0.00000000e+00	0.00000000e+00	0.00000000000000e+00	0.00000000000000e+00
1399	R0001399	EQ	0.00000000000000e+00	0.00000000e+00	0.00000000e+00	0.00000000000000e+00	0.00000000000000e+00
1400	R0001400	EQ	0.00000000000000e+00	0.00000000e+00	0.00000000e+00	0.00000000000000e+00	0.00000000000000e+00
1401	R0001401	EQ	0.00000000000000e+00	0.00000000e+00	0.00000000e+00	0.00000000000000e+00	0.00000000000000e+00
1402	R0001402	EQ	0.00000000000000e+00	0.00000000e+00	0.00000000e+00	0.00000000000000e+00	0.00000000000000e+00
1403	R0001403	EQ	0.00000000000000e+00	0.00000000e+00	0.00000000e+00	0.00000000000000e+00	0.00000000000000e+00
1404	R0001404	EQ	0.00000000000000e+00	0.00000000e+00	0.00000000e+00	0.00000000000000e+00	0.00000000000000e+00
1405	R0001405	EQ	0.00000000000000e+00	0.00000000e+00	0.00000000e+00	0.00000000000000e+00	0.00000000000000e+00
1406	R0001406	EQ	0.00000000000000e+00	0.00000000e+00	0.00000000e+00	0.00000000000000e+00	0.00000000000000e+00
1407	R0001407	EQ	0.00000000000000e+00	0.00000000e+00	0.00000000e+00	0.00000000000000e+00	0.00000000000000e+00
1408	R0001408	EQ	0.00000000000000e+00	0.00000000e+00	0.00000000e+00	0.00000000000000e+00	0.00000000000000e+00
1409	R0001409	EQ	0.00000000000000e+00	0.00000000e+00	0.00000000e+00	0.00000000000000e+00	0.00000000000000e+00
1410	R0001410	EQ	0.00000000000000e+00	0.00000000e+00	0.00000000e+00	0.00000000000000e+00	0.00000000000000e+00
1411	R0001411	EQ	0.00000000000000e+00	0.00000000e+00	0.00000000e+00	0.00000000000000e+00	0.00000000000000e+00
1412	R0001412	EQ	0.00000000000000e+00	0.00000000e+00	0.00000000e+00	0.00000000000000e+00	0.00000000000000e+00
1413	R0001413	EQ	0.00000000000000e+00	0.00000000e+00	0.00000000e+00	0.00000000000000e+00	0.00000000000000e+00
1414	R0001414	EQ	0.00000000000000e+00	0.00000000e+00	0.00000000e+00	0.00000000000000e+00	0.00000000000000e+00
1415	R0001415	EQ	0.00000000000000e+00	0.00000000e+00	0.00000000e+00	0.00000000000000e+00	0.00000000000000e+00
1416	R0001416	EQ	0.00000000000000e+00	0.00000000e+00	0.00000000e+00	0.00000000000000e+00	0.00000000000000e+00
1417	R0001417	EQ	0.00000000000000e+00	0.00000000e+00	0.00000000e+00	0.00000000000000e+00	0.00000000000000e+00
1418	R0001418	EQ	0.00000000000000e+00	0.00000000e+00	0.00000000e+00	0.00000000000000e+00	0.00000000000000e+00
1419	R0001419	EQ	0.00000000000000e+00	0.00000000e+00	0.00000000e+00	0.00000000000000e+00	0.00000000000000e+00
1420	R0001420	EQ	0.00000000000000e+00	0.00000000e+00	0.00000000e+00	0.00000000000000e+00	0.00000000000000e+00
1421	R0001421	EQ	0.00000000000000e+00	0.00000000e+00	0.00000000e+00	0.00000000000000e+00	0.00000000000000e+00
1422	R0001422	EQ	0.00000000000000e+00	0.00000000e+00	0.00000000e+00	0.00000000000000e+00	0.00000000000000e+00
1423	R0001423	EQ	0.00000000000000e+00	0.00000000e+00	0.00000000e+00	0.00000000000000e+00	0.00000000000000e+00
1424	R0001424	EQ	0.00000000000000e+00	0.00000000e+00	0.00000000e+00	0.00000000000000e+00	0.00000000000000e+00
1425	R0001425	EQ	0.00000000000000e+00	0.00000000e+00	0.00000000e+00	0.00000000000000e+00	0.00000000000000e+00
1426	R0001426	EQ	0.00000000000000e+00	0.00000000e+00	0.00000000e+00	0.00000000000000e+00	0.00000000000000e+00
1427	R0001427	EQ	0.00000000000000e+00	0.00000000e+00	0.00000000e+00	0.00000000000000e+00	0.00000000000000e+00
1428	R0001428	EQ	0.00000000000000e+00	0.00000000e+00	0.00000000e+00	0.00000000000000e+00	0.00000000000000e+00
1429	R0001429	EQ	0.00000000000000e+00	0.00000000e+00	0.00000000e+00	0.00000000000000e+00	0.00000000000000e+00
1430	R0001430	EQ	0.00000000000000e+00	0.00000000e+00	0.00000000e+00	0.00000000000000e+00	0.00000000000000e+00
1431	R0001431	EQ	0.00000000000000e+00	0.00000000e+00	0.00000000e+00	0.00000000000000e+00	0.00000000000000e+00
1432	R0001432	EQ	0.00000000000000e+00	0.00000000e+00	0.00000000e+00	0.00000000000000e+00	0.00000000000000e+00
1433	R0001433	EQ	0.00000000000000e+00	0.00000000e+00	0.00000000e+00	0.00000000000000e+00	0.00000000000000e+00
1434	R0001434	EQ	0.00000000000000e+00	0.00000000e+00	0.00000000e+00	0.00000000000000e+00	0.00000000000000e+00
1435	R0001435	EQ	0.00000000000000e+00	0.00000000e+00	0.00000000e+00	0.00000000000000e+00	0.00000000000000e+00
1436	R0001436	EQ	0.00000000000000e+00	0.00000000e+00	0.00000000e+00	0.00000000000000e+00	0.00000000000000e+00
1437	R0001437	EQ	0.00000000000000e+00	0.00000000e+00	0.00000000e+00	0.00000000000000e+00	0.00000000000000e+00
1438	R0001438	EQ	0.00000000000000e+00	0.00000000e+00	0.00000000e+00	0.00000000000000e+00	0.00000000000000e+00
1439	R0001439	EQ	0.00000000000000e+00	0.00000000e+00	0.00000000e+00	0.00000000000000e+00	0.00000000000000e+00
1440	R0001440	EQ	0.00000000000000e+00	0.00000000e+00	0.00000000e+00	0.00000000000000e+00	0.00000000000000e+00
1441	R0001441	EQ	0.00000000000000e+00	0.00000000e+00	0.00000000e+00	0.00000000000000e+00	0.00000000000000e+00
1442	R0001442	EQ	0.00000000000000e+00	0.00000000e+00	0.00000000e+00	0.00000000000000e+00	0.00000000000000e+00
1443	R0001443	EQ	0.00000000000000e+00	0.00000000e+00	0.00000000e+00	0.00000000000000e+00	0.00000000000000e+00
1444	R0001444	EQ	0.00000000000000e+00	0.00000000e+00	0.00000000e+00	0.00000000000000e+00	0.00000000000000e+00
1445	R0001445	EQ	0.00000000000000e+00	0.00000000e+00	0.00000000e+00	0.00000000000000e+00	0.00000000000000e+00
1446	R0001446	EQ	0.00000000000000e+00	0.00000000e+00	0.00000000e+00	0.00000000000000e+00	0.00000000000000e+00
1447	R0001447	EQ	0.00000000000000e+00	0.00000000e+00	0.00000000e+00	0.00000000000000e+00	0.00000000000000e+00
1448	R0001448	EQ	0.00000000000000e+00	0.00000000e+00	0.00000000e+00	0.00000000000000e+00	0.00000000000000e+00
1449	R0001449	EQ	0.00000000000000e+00	0.00000000e+00	0.00000000e+00	0.00000000000000e+00	0.00000000000000e+00
1450	R0001450	EQ	0.00000000000000e+00	0.00000000e+00	0.00000000e+00	0.00000000000000e+00	0.00000000000000e+00
1451	R0001451	EQ	0.00000000000000e+00	0.00000000e+00	0.00000000e+00	0.00000000000000e+00	0.00000000000000e+00
1452	R0001452	EQ	0.00000000000000e+00	0.00000000e+00	0.00000000e+00	0.00000000000000e+00	0.00000000000000e+00
1453	R0001453	EQ	0.00000000000000e+00	0.00000000e+00	0.00000000e+00	0.00000000000000e+00	0.00000000000000e+00
1454	R0001454	EQ	0.00000000000000e+00	0.00000000e+00	0.00000000e+00	0.00000000000000e+00	0.00000000000000e+00
1455	R0001455	EQ	0.00000000000000e+00	0.00000000e+00	0.00000000e+00	0.00000000000000e+00	0.00000000000000e+00
1456	R0001456	EQ	0.00000000000000e+00	0.00000000e+00	0.00000000e+00	0.00000000000000e+00	0.00000000000000e+00
1457	R0001457	EQ	0.00000000000000e+00	0.00000000e+00	0.00000000e+00	0.00000000000000e+00	0.00000000000000e+00
1458	R0001458	EQ	0.00000000000000e+00	0.00000000e+00	0.00000000e+00	0.00000000000000e+00	0.00000000000000e+00
1459	R0001459	EQ	0.00000000000000e+00	0.00000000e+00	0.00000000e+00	0.00000000000000e+00	0.00000000000000e+00
1460	R0001460	EQ	0.00000000000000e+00	0.00000000e+00	0.00000000e+00	0.00000000000000e+00	0.00000000000000e+00
1461	R0001461	EQ	0.00000000000000e+00	0.00000000e+00	0.00000000e+00	0.00000000000000e+00	0.00000000000000e+00
1462	R0001462	EQ	0.00000000000000e+00	0.00000000e+00	0.00000000e+00	0.00000000000000e+00	0.00000000000000e+00
1463	R0001463	EQ	0.00000000000000e+00	0.00000000e+00	0.00000000e+00	0.00000000000000e+00	0.00000000000000e+00
1464	R0001464	EQ	0.00000000000000e+00	0.00000000e+00	0.00000000e+00	0.00000000000000e+00	0.00000000000000e+00
1465	R0001465	EQ	0.00000000000000e+00	0.00000000e+00	0.00000000e+00	0.00000000000000e+00	0.00000000000000e+00
1466	R0001466	EQ	0.00000000000000e+00	0.00000000e+00	0.00000000e+00	0.00000000000000e+00	0.00000000000000e+00
1467	R0001467	EQ	0.00000000000000e+00	0.00000000e+00	0.00000000e+00	0.00000000000000e+00	0.00000000000000e+00
1468	R0001468	EQ	0.00000000000000e+00	0.00000000e+00	0.00000000e+00	0.00000000000000e+00	0.00000000000000e+00
1469	R0001469	EQ	0.00000000000000e+00	0.00000000e+00	0.00000000e+00	0.00000000000000e+00	0.00000000000000e+00
1470	R0001470	EQ	0.00000000000000e+00	0.00000000e+00	0.00000000e+00	0.00000000000000e+00	0.00000000000000e+00
1471	R0001471	EQ	0.00000000000000e+00	0.00000000e+00	0.00000000e+00	0.00000000000000e+00	0.00000000000000e+00
1472	R0001472	EQ	0.00000000000000e+00	0.00000000e+00	0.00000000e+00	0.00000000000000e+00	0.00000000000000e+00
1473	R0001473	EQ	0.00000000000000e+00	0.00000000e+00	0.00000000e+00	0.00000000000000e+00	0.00000000000000e+00
1474	R0001474	EQ	0.00000000000000e+00	0.00000000e+00	0.00000000e+00	0.00000000000000e+00	0.00000000000000e+00
1475	R0001475	EQ	0.00000000000000e+00	0.00000000e+00	0.00000000e+00	0.00000000000000e+00	0.00000000000000e+00
1476	R0001476	EQ	0.00000000000000e+00	0.00000000e+00	0.00000000e+00	0.00000000000000e+00	0.00000000000000e+00
1477	R0001477	EQ	0.00000000000000e+00	0.00000000e+00	0.00000000e+00	0.00000000000000e+00	0.00000000000000e+00
1478	R0001478	EQ	0.00000000000000e+00	0.00000000e+00	0.00000000e+00	0.00000000000000e+00	0.00000000000000e+00
1479	R0001479	EQ	0.00000000000000e+00	0.00000000e+00	0.00000000e+00	0.00000000000000e+00	0.00000000000000e+00
1480	R0001480	EQ	0.00000000000000e+00	0.00000000e+00	0.00000000e+00	0.00000000000000e+00	0.00000000000000e+00
1481	R0001481	EQ	0.00000000000000e+00	0.00000000e+00	0.00000000e+00	0.00000000000000e+00	0.00000000000000e+00
1482	R0001482	EQ	0.00000000000000e+00	0.00000000e+00	0.00000000e+00	0.00000000000000e+00	0.00000000000000e+00
1483	R0001483	EQ	0.00000000000000e+00	0.00000000e+00	0.00000000e+00	0.00000000000000e+00	0.00000000000000e+00
1484	R0001484	EQ	0.00000000000000e+00	0.00000000e+00	0.00000000e+00	0.00000000000000e+00	0.00000000000000e+00

1485	R0001485	EQ	0.00000000000000e+00	0.00000000e+00	0.00000000e+00	0.00000000000000e+00	0.00000000000000e+00
1486	R0001486	EQ	0.00000000000000e+00	0.00000000e+00	0.00000000e+00	0.00000000000000e+00	0.00000000000000e+00
1487	R0001487	EQ	0.00000000000000e+00	0.00000000e+00	0.00000000e+00	0.00000000000000e+00	0.00000000000000e+00
1488	R0001488	EQ	0.00000000000000e+00	0.00000000e+00	0.00000000e+00	0.00000000000000e+00	0.00000000000000e+00
1489	R0001489	EQ	0.00000000000000e+00	0.00000000e+00	0.00000000e+00	0.00000000000000e+00	0.00000000000000e+00
1490	R0001490	EQ	0.00000000000000e+00	0.00000000e+00	0.00000000e+00	0.00000000000000e+00	0.00000000000000e+00
1491	R0001491	EQ	0.00000000000000e+00	0.00000000e+00	0.00000000e+00	0.00000000000000e+00	0.00000000000000e+00
1492	R0001492	EQ	0.00000000000000e+00	0.00000000e+00	0.00000000e+00	0.00000000000000e+00	0.00000000000000e+00
1493	R0001493	EQ	0.00000000000000e+00	0.00000000e+00	0.00000000e+00	0.00000000000000e+00	0.00000000000000e+00
1494	R0001494	EQ	0.00000000000000e+00	0.00000000e+00	0.00000000e+00	0.00000000000000e+00	0.00000000000000e+00
1495	R0001495	EQ	0.00000000000000e+00	0.00000000e+00	0.00000000e+00	0.00000000000000e+00	0.00000000000000e+00
1496	R0001496	EQ	0.00000000000000e+00	0.00000000e+00	0.00000000e+00	0.00000000000000e+00	0.00000000000000e+00
1497	R0001497	EQ	0.00000000000000e+00	0.00000000e+00	0.00000000e+00	0.00000000000000e+00	0.00000000000000e+00
1498	R0001498	EQ	0.00000000000000e+00	0.00000000e+00	0.00000000e+00	0.00000000000000e+00	0.00000000000000e+00
1499	R0001499	EQ	0.00000000000000e+00	0.00000000e+00	0.00000000e+00	0.00000000000000e+00	0.00000000000000e+00
1500	R0001500	EQ	0.00000000000000e+00	0.00000000e+00	0.00000000e+00	0.00000000000000e+00	0.00000000000000e+00
1501	R0001501	EQ	0.00000000000000e+00	0.00000000e+00	0.00000000e+00	0.00000000000000e+00	0.00000000000000e+00
1502	R0001502	EQ	0.00000000000000e+00	0.00000000e+00	0.00000000e+00	0.00000000000000e+00	0.00000000000000e+00
1503	R0001503	EQ	0.00000000000000e+00	0.00000000e+00	0.00000000e+00	0.00000000000000e+00	0.00000000000000e+00
1504	R0001504	EQ	0.00000000000000e+00	0.00000000e+00	0.00000000e+00	0.00000000000000e+00	0.00000000000000e+00
1505	R0001505	EQ	0.00000000000000e+00	0.00000000e+00	0.00000000e+00	0.00000000000000e+00	0.00000000000000e+00
1506	R0001506	EQ	0.00000000000000e+00	0.00000000e+00	0.00000000e+00	0.00000000000000e+00	0.00000000000000e+00
1507	R0001507	EQ	0.00000000000000e+00	0.00000000e+00	0.00000000e+00	0.00000000000000e+00	0.00000000000000e+00
1508	R0001508	EQ	0.00000000000000e+00	0.00000000e+00	0.00000000e+00	0.00000000000000e+00	0.00000000000000e+00
1509	R0001509	EQ	0.00000000000000e+00	0.00000000e+00	0.00000000e+00	0.00000000000000e+00	0.00000000000000e+00
1510	R0001510	EQ	0.00000000000000e+00	0.00000000e+00	0.00000000e+00	0.00000000000000e+00	0.00000000000000e+00
1511	R0001511	EQ	0.00000000000000e+00	0.00000000e+00	0.00000000e+00	0.00000000000000e+00	0.00000000000000e+00
1512	R0001512	EQ	0.00000000000000e+00	0.00000000e+00	0.00000000e+00	0.00000000000000e+00	0.00000000000000e+00
1513	R0001513	EQ	0.00000000000000e+00	0.00000000e+00	0.00000000e+00	0.00000000000000e+00	0.00000000000000e+00
1514	R0001514	EQ	0.00000000000000e+00	0.00000000e+00	0.00000000e+00	0.00000000000000e+00	0.00000000000000e+00
1515	R0001515	EQ	0.00000000000000e+00	0.00000000e+00	0.00000000e+00	0.00000000000000e+00	0.00000000000000e+00
1516	R0001516	EQ	0.00000000000000e+00	0.00000000e+00	0.00000000e+00	0.00000000000000e+00	0.00000000000000e+00
1517	R0001517	EQ	0.00000000000000e+00	0.00000000e+00	0.00000000e+00	0.00000000000000e+00	0.00000000000000e+00
1518	R0001518	EQ	0.00000000000000e+00	0.00000000e+00	0.00000000e+00	0.00000000000000e+00	0.00000000000000e+00
1519	R0001519	EQ	0.00000000000000e+00	0.00000000e+00	0.00000000e+00	0.00000000000000e+00	0.00000000000000e+00
1520	R0001520	EQ	0.00000000000000e+00	0.00000000e+00	0.00000000e+00	0.00000000000000e+00	0.00000000000000e+00
1521	R0001521	EQ	0.00000000000000e+00	0.00000000e+00	0.00000000e+00	0.00000000000000e+00	0.00000000000000e+00
1522	R0001522	EQ	0.00000000000000e+00	0.00000000e+00	0.00000000e+00	0.00000000000000e+00	0.00000000000000e+00
1523	R0001523	EQ	0.00000000000000e+00	0.00000000e+00	0.00000000e+00	0.00000000000000e+00	0.00000000000000e+00
1524	R0001524	EQ	0.00000000000000e+00	0.00000000e+00	0.00000000e+00	0.00000000000000e+00	0.00000000000000e+00
1525	R0001525	EQ	0.00000000000000e+00	0.00000000e+00	0.00000000e+00	0.00000000000000e+00	0.00000000000000e+00
1526	R0001526	EQ	0.00000000000000e+00	0.00000000e+00	0.00000000e+00	0.00000000000000e+00	0.00000000000000e+00
1527	R0001527	EQ	0.00000000000000e+00	0.00000000e+00	0.00000000e+00	0.00000000000000e+00	0.00000000000000e+00
1528	R0001528	EQ	0.00000000000000e+00	0.00000000e+00	0.00000000e+00	0.00000000000000e+00	0.00000000000000e+00
1529	R0001529	EQ	0.00000000000000e+00	0.00000000e+00	0.00000000e+00	0.00000000000000e+00	0.00000000000000e+00
1530	R0001530	EQ	0.00000000000000e+00	0.00000000e+00	0.00000000e+00	0.00000000000000e+00	0.00000000000000e+00
1531	R0001531	EQ	0.00000000000000e+00	0.00000000e+00	0.00000000e+00	0.00000000000000e+00	0.00000000000000e+00
1532	R0001532	EQ	0.00000000000000e+00	0.00000000e+00	0.00000000e+00	0.00000000000000e+00	0.00000000000000e+00
1533	R0001533	EQ	0.00000000000000e+00	0.00000000e+00	0.00000000e+00	0.00000000000000e+00	0.00000000000000e+00
1534	R0001534	EQ	0.00000000000000e+00	0.00000000e+00	0.00000000e+00	0.00000000000000e+00	0.00000000000000e+00
1535	R0001535	EQ	0.00000000000000e+00	0.00000000e+00	0.00000000e+00	0.00000000000000e+00	0.00000000000000e+00
1536	R0001536	EQ	0.00000000000000e+00	0.00000000e+00	0.00000000e+00	0.00000000000000e+00	0.00000000000000e+00
1537	R0001537	EQ	0.00000000000000e+00	0.00000000e+00	0.00000000e+00	0.00000000000000e+00	0.00000000000000e+00
1538	R0001538	EQ	0.00000000000000e+00	0.00000000e+00	0.00000000e+00	0.00000000000000e+00	0.00000000000000e+00
1539	R0001539	EQ	0.00000000000000e+00	0.00000000e+00	0.00000000e+00	0.00000000000000e+00	0.00000000000000e+00
1540	R0001540	EQ	0.00000000000000e+00	0.00000000e+00	0.00000000e+00	0.00000000000000e+00	0.00000000000000e+00
1541	R0001541	EQ	0.00000000000000e+00	0.00000000e+00	0.00000000e+00	0.00000000000000e+00	0.00000000000000e+00
1542	R0001542	EQ	0.00000000000000e+00	0.00000000e+00	0.00000000e+00	0.00000000000000e+00	0.00000000000000e+00
1543	R0001543	EQ	0.00000000000000e+00	0.00000000e+00	0.00000000e+00	0.00000000000000e+00	0.00000000000000e+00
1544	R0001544	EQ	0.00000000000000e+00	0.00000000e+00	0.00000000e+00	0.00000000000000e+00	0.00000000000000e+00
1545	R0001545	EQ	0.00000000000000e+00	0.00000000e+00	0.00000000e+00	0.00000000000000e+00	0.00000000000000e+00
1546	R0001546	EQ	0.00000000000000e+00	0.00000000e+00	0.00000000e+00	0.00000000000000e+00	0.00000000000000e+00
1547	R0001547	EQ	0.00000000000000e+00	0.00000000e+00	0.00000000e+00	0.00000000000000e+00	0.00000000000000e+00
1548	R0001548	EQ	0.00000000000000e+00	0.00000000e+00	0.00000000e+00	0.00000000000000e+00	0.00000000000000e+00
1549	R0001549	EQ	0.00000000000000e+00	0.00000000e+00	0.00000000e+00	0.00000000000000e+00	0.00000000000000e+00
1550	R0001550	EQ	0.00000000000000e+00	0.00000000e+00	0.00000000e+00	0.00000000000000e+00	0.00000000000000e+00
1551	R0001551	EQ	0.00000000000000e+00	0.00000000e+00	0.00000000e+00	0.00000000000000e+00	0.00000000000000e+00
1552	R0001552	EQ	0.00000000000000e+00	0.00000000e+00	0.00000000e+00	0.00000000000000e+00	0.00000000000000e+00
1553	R0001553	EQ	0.00000000000000e+00	0.00000000e+00	0.00000000e+00	0.00000000000000e+00	0.00000000000000e+00
1554	R0001554	EQ	0.00000000000000e+00	0.00000000e+00	0.00000000e+00	0.00000000000000e+00	0.00000000000000e+00
1555	R0001555	EQ	0.00000000000000e+00	0.00000000e+00	0.00000000e+00	0.00000000000000e+00	0.00000000000000e+00
1556	R0001556	EQ	0.00000000000000e+00	0.00000000e+00	0.00000000e+00	0.00000000000000e+00	0.00000000000000e+00
1557	R0001557	EQ	0.00000000000000e+00	0.00000000e+00	0.00000000e+00	0.00000000000000e+00	0.00000000000000e+00
1558	R0001558	EQ	0.00000000000000e+00	0.00000000e+00	0.00000000e+00	0.00000000000000e+00	0.00000000000000e+00
1559	R0001559	EQ	0.00000000000000e+00	0.00000000e+00	0.00000000e+00	0.00000000000000e+00	0.00000000000000e+00
1560	R0001560	EQ	0.00000000000000e+00	0.00000000e+00	0.00000000e+00	0.00000000000000e+00	0.00000000000000e+00
1561	R0001561	EQ	0.00000000000000e+00	0.00000000e+00	0.00000000e+00	0.00000000000000e+00	0.00000000000000e+00
1562	R0001562	EQ	0.00000000000000e+00	0.00000000e+00	0.00000000e+00	0.00000000000000e+00	0.00000000000000e+00
1563	R0001563	EQ	0.00000000000000e+00	0.00000000e+00	0.00000000e+00	0.00000000000000e+00	0.00000000000000e+00
1564	R0001564	EQ	0.00000000000000e+00	0.00000000e+00	0.00000000e+00	0.00000000000000e+00	0.00000000000000e+00
1565	R0001565	EQ	0.00000000000000e+00	0.00000000e+00	0.00000000e+00	0.00000000000000e+00	0.00000000000000e+00
1566	R0001566	EQ	0.00000000000000e+00	0.00000000e+00	0.00000000e+00	0.00000000000000e+00	0.00000000000000e+00
1567	R0001567	EQ	0.00000000000000e+00	0.00000000e+00	0.00000000e+00	0.00000000000000e+00	0.00000000000000e+00
1568	R0001568	EQ	0.00000000000000e+00	0.00000000e+00	0.00000000e+00	0.00000000000000e+00	0.00000000000000e+00
1569	R0001569	EQ	0.00000000000000e+00	0.00000000e+00	0.00000000e+00	0.00000000000000e+00	0.00000000000000e+00
1570	R0001570	EQ	0.00000000000000e+00	0.00000000e+00	0.00000000e+00	0.00000000000000e+00	0.00000000000000e+00
1571	R0001571	EQ	0.00000000000000e+00	0.00000000e+00	0.00000000e+00	0.00000000000000e+00	0.00000000000000e+00
1572	R0001572	EQ	0.00000000000000e+00	0.00000000e+00	0.00000000e+00	0.00000000000000e+00	0.00000000000000e+00
1573	R0001573	EQ	0.00000000000000e+00	0.00000000e+00	0.00000000e+00	0.00000000000000e+00	0.00000000000000e+00
1574	R0001574	EQ	0.00000000000000e+00	0.00000000e+00	0.00000000e+00	0.00000000000000e+00	0.00000000000000e+00
1575	R0001575	EQ	0.00000000000000e+00	0.00000000e+00	0.00000000e+00	0.00000000000000e+00	0.00000000000000e+00
1576	R0001576	EQ	0.00000000000000e+00	0.00000000e+00	0.00000000e+00	0.00000000000000e+00	0.00000000000000e+00
1577	R0001577	EQ	0.00000000000000e+00	0.00000000e+00	0.00000000e+00	0.00000000000000e+00	0.00000000000000e+00
1578	R0001578	EQ	0.00000000000000e+00	0.00000000e+00	0.00000000e+00	0.00000000000000e+00	0.00000000000000e+00
1579	R0001579	EQ	0.00000000000000e+00	0.00000000e+00	0.00000000e+00	0.00000000000000e+00	0.00000000000000e+00
1580	R0001580	EQ	0.00000000000000e+00	0.00000000e+00	0.00000000e+00	0.00000000000000e+00	0.00000000000000e+00
1581	R0001581	EQ	0.00000000000000e+00	0.00000000e+00	0.00000000e+00	0.00000000000000e+00	0.00000000000000e+00
1582	R0001582	EQ	0.00000000000000e+00	0.00000000e+00	0.00000000e+00	0.00000000000000e+00	0.00000000000000e+00

1583	R0001583	EQ	0.00000000000000e+00	0.00000000e+00	0.00000000e+00	0.00000000000000e+00	0.00000000000000e+00
1584	R0001584	EQ	0.00000000000000e+00	0.00000000e+00	0.00000000e+00	0.00000000000000e+00	0.00000000000000e+00
1585	R0001585	EQ	0.00000000000000e+00	0.00000000e+00	0.00000000e+00	0.00000000000000e+00	0.00000000000000e+00
1586	R0001586	EQ	0.00000000000000e+00	0.00000000e+00	0.00000000e+00	0.00000000000000e+00	0.00000000000000e+00
1587	R0001587	EQ	0.00000000000000e+00	0.00000000e+00	0.00000000e+00	0.00000000000000e+00	0.00000000000000e+00
1588	R0001588	EQ	0.00000000000000e+00	0.00000000e+00	0.00000000e+00	0.00000000000000e+00	0.00000000000000e+00
1589	R0001589	EQ	0.00000000000000e+00	0.00000000e+00	0.00000000e+00	0.00000000000000e+00	0.00000000000000e+00
1590	R0001590	EQ	0.00000000000000e+00	0.00000000e+00	0.00000000e+00	0.00000000000000e+00	0.00000000000000e+00
1591	R0001591	EQ	0.00000000000000e+00	0.00000000e+00	0.00000000e+00	0.00000000000000e+00	0.00000000000000e+00
1592	R0001592	EQ	0.00000000000000e+00	0.00000000e+00	0.00000000e+00	0.00000000000000e+00	0.00000000000000e+00
1593	R0001593	EQ	0.00000000000000e+00	0.00000000e+00	0.00000000e+00	0.00000000000000e+00	0.00000000000000e+00
1594	R0001594	EQ	0.00000000000000e+00	0.00000000e+00	0.00000000e+00	0.00000000000000e+00	0.00000000000000e+00
1595	R0001595	EQ	0.00000000000000e+00	0.00000000e+00	0.00000000e+00	0.00000000000000e+00	0.00000000000000e+00
1596	R0001596	EQ	0.00000000000000e+00	0.00000000e+00	0.00000000e+00	0.00000000000000e+00	0.00000000000000e+00
1597	R0001597	EQ	0.00000000000000e+00	0.00000000e+00	0.00000000e+00	0.00000000000000e+00	0.00000000000000e+00
1598	R0001598	EQ	0.00000000000000e+00	0.00000000e+00	0.00000000e+00	0.00000000000000e+00	0.00000000000000e+00
1599	R0001599	EQ	0.00000000000000e+00	0.00000000e+00	0.00000000e+00	0.00000000000000e+00	0.00000000000000e+00
1600	R0001600	EQ	0.00000000000000e+00	0.00000000e+00	0.00000000e+00	0.00000000000000e+00	0.00000000000000e+00
1601	R0001601	EQ	0.00000000000000e+00	0.00000000e+00	0.00000000e+00	0.00000000000000e+00	0.00000000000000e+00
1602	R0001602	EQ	0.00000000000000e+00	0.00000000e+00	0.00000000e+00	0.00000000000000e+00	0.00000000000000e+00
1603	R0001603	EQ	0.00000000000000e+00	0.00000000e+00	0.00000000e+00	0.00000000000000e+00	0.00000000000000e+00
1604	R0001604	EQ	0.00000000000000e+00	0.00000000e+00	0.00000000e+00	0.00000000000000e+00	0.00000000000000e+00
1605	R0001605	EQ	0.00000000000000e+00	0.00000000e+00	0.00000000e+00	0.00000000000000e+00	0.00000000000000e+00
1606	R0001606	EQ	0.00000000000000e+00	0.00000000e+00	0.00000000e+00	0.00000000000000e+00	0.00000000000000e+00
1607	R0001607	EQ	0.00000000000000e+00	0.00000000e+00	0.00000000e+00	0.00000000000000e+00	0.00000000000000e+00
1608	R0001608	EQ	0.00000000000000e+00	0.00000000e+00	0.00000000e+00	0.00000000000000e+00	0.00000000000000e+00
1609	R0001609	EQ	0.00000000000000e+00	0.00000000e+00	0.00000000e+00	0.00000000000000e+00	0.00000000000000e+00
1610	R0001610	EQ	0.00000000000000e+00	0.00000000e+00	0.00000000e+00	0.00000000000000e+00	0.00000000000000e+00
1611	R0001611	EQ	0.00000000000000e+00	0.00000000e+00	0.00000000e+00	0.00000000000000e+00	0.00000000000000e+00
1612	R0001612	EQ	0.00000000000000e+00	0.00000000e+00	0.00000000e+00	0.00000000000000e+00	0.00000000000000e+00
1613	R0001613	EQ	0.00000000000000e+00	0.00000000e+00	0.00000000e+00	0.00000000000000e+00	0.00000000000000e+00
1614	R0001614	EQ	0.00000000000000e+00	0.00000000e+00	0.00000000e+00	0.00000000000000e+00	0.00000000000000e+00
1615	R0001615	EQ	0.00000000000000e+00	0.00000000e+00	0.00000000e+00	0.00000000000000e+00	0.00000000000000e+00
1616	R0001616	EQ	0.00000000000000e+00	0.00000000e+00	0.00000000e+00	0.00000000000000e+00	0.00000000000000e+00
1617	R0001617	EQ	0.00000000000000e+00	0.00000000e+00	0.00000000e+00	0.00000000000000e+00	0.00000000000000e+00
1618	R0001618	EQ	0.00000000000000e+00	0.00000000e+00	0.00000000e+00	0.00000000000000e+00	0.00000000000000e+00
1619	R0001619	EQ	0.00000000000000e+00	0.00000000e+00	0.00000000e+00	0.00000000000000e+00	0.00000000000000e+00
1620	R0001620	EQ	0.00000000000000e+00	0.00000000e+00	0.00000000e+00	0.00000000000000e+00	0.00000000000000e+00
1621	R0001621	EQ	0.00000000000000e+00	0.00000000e+00	0.00000000e+00	0.00000000000000e+00	0.00000000000000e+00
1622	R0001622	EQ	0.00000000000000e+00	0.00000000e+00	0.00000000e+00	0.00000000000000e+00	0.00000000000000e+00
1623	R0001623	EQ	0.00000000000000e+00	0.00000000e+00	0.00000000e+00	0.00000000000000e+00	0.00000000000000e+00
1624	R0001624	EQ	0.00000000000000e+00	0.00000000e+00	0.00000000e+00	0.00000000000000e+00	0.00000000000000e+00
1625	R0001625	EQ	0.00000000000000e+00	0.00000000e+00	0.00000000e+00	0.00000000000000e+00	0.00000000000000e+00
1626	R0001626	EQ	0.00000000000000e+00	0.00000000e+00	0.00000000e+00	0.00000000000000e+00	0.00000000000000e+00
1627	R0001627	EQ	0.00000000000000e+00	0.00000000e+00	0.00000000e+00	0.00000000000000e+00	0.00000000000000e+00
1628	R0001628	EQ	0.00000000000000e+00	0.00000000e+00	0.00000000e+00	0.00000000000000e+00	0.00000000000000e+00
1629	R0001629	EQ	0.00000000000000e+00	0.00000000e+00	0.00000000e+00	0.00000000000000e+00	0.00000000000000e+00
1630	R0001630	EQ	0.00000000000000e+00	0.00000000e+00	0.00000000e+00	0.00000000000000e+00	0.00000000000000e+00
1631	R0001631	EQ	0.00000000000000e+00	0.00000000e+00	0.00000000e+00	0.00000000000000e+00	0.00000000000000e+00
1632	R0001632	EQ	0.00000000000000e+00	0.00000000e+00	0.00000000e+00	0.00000000000000e+00	0.00000000000000e+00
1633	R0001633	EQ	0.00000000000000e+00	0.00000000e+00	0.00000000e+00	0.00000000000000e+00	0.00000000000000e+00
1634	R0001634	EQ	0.00000000000000e+00	0.00000000e+00	0.00000000e+00	0.00000000000000e+00	0.00000000000000e+00
1635	R0001635	EQ	0.00000000000000e+00	0.00000000e+00	0.00000000e+00	0.00000000000000e+00	0.00000000000000e+00
1636	R0001636	EQ	0.00000000000000e+00	0.00000000e+00	0.00000000e+00	0.00000000000000e+00	0.00000000000000e+00
1637	R0001637	EQ	0.00000000000000e+00	0.00000000e+00	0.00000000e+00	0.00000000000000e+00	0.00000000000000e+00
1638	R0001638	EQ	0.00000000000000e+00	0.00000000e+00	0.00000000e+00	0.00000000000000e+00	0.00000000000000e+00
1639	R0001639	EQ	0.00000000000000e+00	0.00000000e+00	0.00000000e+00	0.00000000000000e+00	0.00000000000000e+00
1640	R0001640	EQ	0.00000000000000e+00	0.00000000e+00	0.00000000e+00	0.00000000000000e+00	0.00000000000000e+00
1641	R0001641	EQ	0.00000000000000e+00	0.00000000e+00	0.00000000e+00	0.00000000000000e+00	0.00000000000000e+00
1642	R0001642	EQ	0.00000000000000e+00	0.00000000e+00	0.00000000e+00	0.00000000000000e+00	0.00000000000000e+00
1643	R0001643	EQ	0.00000000000000e+00	0.00000000e+00	0.00000000e+00	0.00000000000000e+00	0.00000000000000e+00
1644	R0001644	EQ	0.00000000000000e+00	0.00000000e+00	0.00000000e+00	0.00000000000000e+00	0.00000000000000e+00
1645	R0001645	EQ	0.00000000000000e+00	0.00000000e+00	0.00000000e+00	0.00000000000000e+00	0.00000000000000e+00
1646	R0001646	EQ	0.00000000000000e+00	0.00000000e+00	0.00000000e+00	0.00000000000000e+00	0.00000000000000e+00
1647	R0001647	EQ	0.00000000000000e+00	0.00000000e+00	0.00000000e+00	0.00000000000000e+00	0.00000000000000e+00
1648	R0001648	EQ	0.00000000000000e+00	0.00000000e+00	0.00000000e+00	0.00000000000000e+00	0.00000000000000e+00
1649	R0001649	EQ	0.00000000000000e+00	0.00000000e+00	0.00000000e+00	0.00000000000000e+00	0.00000000000000e+00
1650	R0001650	EQ	0.00000000000000e+00	0.00000000e+00	0.00000000e+00	0.00000000000000e+00	0.00000000000000e+00
1651	R0001651	EQ	0.00000000000000e+00	0.00000000e+00	0.00000000e+00	0.00000000000000e+00	0.00000000000000e+00
1652	R0001652	EQ	0.00000000000000e+00	0.00000000e+00	0.00000000e+00	0.00000000000000e+00	0.00000000000000e+00
1653	R0001653	EQ	0.00000000000000e+00	0.00000000e+00	0.00000000e+00	0.00000000000000e+00	0.00000000000000e+00
1654	R0001654	EQ	0.00000000000000e+00	0.00000000e+00	0.00000000e+00	0.00000000000000e+00	0.00000000000000e+00
1655	R0001655	EQ	0.00000000000000e+00	0.00000000e+00	0.00000000e+00	0.00000000000000e+00	0.00000000000000e+00
1656	R0001656	EQ	0.00000000000000e+00	0.00000000e+00	0.00000000e+00	0.00000000000000e+00	0.00000000000000e+00
1657	R0001657	EQ	0.00000000000000e+00	0.00000000e+00	0.00000000e+00	0.00000000000000e+00	0.00000000000000e+00
1658	R0001658	EQ	0.00000000000000e+00	0.00000000e+00	0.00000000e+00	0.00000000000000e+00	0.00000000000000e+00
1659	R0001659	EQ	0.00000000000000e+00	0.00000000e+00	0.00000000e+00	0.00000000000000e+00	0.00000000000000e+00
1660	R0001660	EQ	0.00000000000000e+00	0.00000000e+00	0.00000000e+00	0.00000000000000e+00	0.00000000000000e+00
1661	R0001661	EQ	0.00000000000000e+00	0.00000000e+00	0.00000000e+00	0.00000000000000e+00	0.00000000000000e+00
1662	R0001662	EQ	0.00000000000000e+00	0.00000000e+00	0.00000000e+00	0.00000000000000e+00	0.00000000000000e+00
1663	R0001663	EQ	0.00000000000000e+00	0.00000000e+00	0.00000000e+00	0.00000000000000e+00	0.00000000000000e+00
1664	R0001664	EQ	0.00000000000000e+00	0.00000000e+00	0.00000000e+00	0.00000000000000e+00	0.00000000000000e+00
1665	R0001665	EQ	0.00000000000000e+00	0.00000000e+00	0.00000000e+00	0.00000000000000e+00	0.00000000000000e+00
1666	R0001666	EQ	0.00000000000000e+00	0.00000000e+00	0.00000000e+00	0.00000000000000e+00	0.00000000000000e+00
1667	R0001667	EQ	0.00000000000000e+00	0.00000000e+00	0.00000000e+00	0.00000000000000e+00	0.00000000000000e+00
1668	R0001668	EQ	0.00000000000000e+00	0.00000000e+00	0.00000000e+00	0.00000000000000e+00	0.00000000000000e+00
1669	R0001669	EQ	0.00000000000000e+00	0.00000000e+00	0.00000000e+00	0.00000000000000e+00	0.00000000000000e+00
1670	R0001670	EQ	0.00000000000000e+00	0.00000000e+00	0.00000000e+00	0.00000000000000e+00	0.00000000000000e+00
1671	R0001671	EQ	0.00000000000000e+00	0.00000000e+00	0.00000000e+00	0.00000000000000e+00	0.00000000000000e+00
1672	R0001672	EQ	0.00000000000000e+00	0.00000000e+00	0.00000000e+00	0.00000000000000e+00	0.00000000000000e+00
1673	R0001673	EQ	0.00000000000000e+00	0.00000000e+00	0.00000000e+00	0.00000000000000e+00	0.00000000000000e+00
1674	R0001674	EQ	0.00000000000000e+00	0.00000000e+00	0.00000000e+00	0.00000000000000e+00	0.00000000000000e+00
1675	R0001675	EQ	0.00000000000000e+00	0.00000000e+00	0.00000000e+00	0.00000000000000e+00	0.00000000000000e+00
1676	R0001676	EQ	0.00000000000000e+00	0.00000000e+00	0.00000000e+00	0.00000000000000e+00	0.00000000000000e+00
1677	R0001677	EQ	0.00000000000000e+00	0.00000000e+00	0.00000000e+00	0.00000000000000e+00	0.00000000000000e+00
1678	R0001678	EQ	0.00000000000000e+00	0.00000000e+00	0.00000000e+00	0.00000000000000e+00	0.00000000000000e+00
1679	R0001679	EQ	0.00000000000000e+00	0.00000000e+00	0.00000000e+00	0.00000000000000e+00	0.00000000000000e+00
1680	R0001680	EQ	0.00000000000000e+00	0.00000000e+00	0.00000000e+00	0.00000000000000e+00	0.00000000000000e+00

1681	R0001681	EQ	0.00000000000000e+00	0.00000000e+00	0.00000000e+00	0.00000000000000e+00	0.00000000000000e+00
1682	R0001682	EQ	0.00000000000000e+00	0.00000000e+00	0.00000000e+00	0.00000000000000e+00	0.00000000000000e+00
1683	R0001683	EQ	0.00000000000000e+00	0.00000000e+00	0.00000000e+00	0.00000000000000e+00	0.00000000000000e+00
1684	R0001684	EQ	0.00000000000000e+00	0.00000000e+00	0.00000000e+00	0.00000000000000e+00	0.00000000000000e+00
1685	R0001685	EQ	0.00000000000000e+00	0.00000000e+00	0.00000000e+00	0.00000000000000e+00	0.00000000000000e+00
1686	R0001686	EQ	0.00000000000000e+00	0.00000000e+00	0.00000000e+00	0.00000000000000e+00	0.00000000000000e+00
1687	R0001687	EQ	0.00000000000000e+00	0.00000000e+00	0.00000000e+00	0.00000000000000e+00	0.00000000000000e+00
1688	R0001688	EQ	0.00000000000000e+00	0.00000000e+00	0.00000000e+00	0.00000000000000e+00	0.00000000000000e+00
1689	R0001689	EQ	0.00000000000000e+00	0.00000000e+00	0.00000000e+00	0.00000000000000e+00	0.00000000000000e+00
1690	R0001690	EQ	0.00000000000000e+00	0.00000000e+00	0.00000000e+00	0.00000000000000e+00	0.00000000000000e+00
1691	R0001691	EQ	0.00000000000000e+00	0.00000000e+00	0.00000000e+00	0.00000000000000e+00	0.00000000000000e+00
1692	R0001692	EQ	0.00000000000000e+00	0.00000000e+00	0.00000000e+00	0.00000000000000e+00	0.00000000000000e+00
1693	R0001693	EQ	1.00000000000000e+00	1.00000000e+00	1.00000000e+00	3.79285717751623e+01	0.00000000000000e+00

VARIABLES

INDEX	NAME	AT	ACTIVITY	LOWER LIMIT	UPPER LIMIT	DUAL LOWER	DUAL UPPER
0	C0000000	SB	0.00000000000000e+00	0.00000000e+00	NONE	0.00000000000000e+00	0.00000000000000e+00
1	C0000001	SB	0.00000000000000e+00	0.00000000e+00	NONE	0.00000000000000e+00	0.00000000000000e+00
2	C0000002	SB	-0.00000000000000e+00	0.00000000e+00	NONE	0.00000000000000e+00	0.00000000000000e+00
3	C0000003	LL	0.00000000000000e+00	0.00000000e+00	NONE	0.00000000000000e+00	0.00000000000000e+00
4	C0000004	LL	0.00000000000000e+00	0.00000000e+00	NONE	0.00000000000000e+00	0.00000000000000e+00
5	C0000005	SB	2.99338197122179e+10	NONE	NONE	0.00000000000000e+00	0.00000000000000e+00
6	C0000006	SB	1.18207846309196e+00	NONE	NONE	0.00000000000000e+00	0.00000000000000e+00
7	C0000007	SB	-1.39269785360470e+11	NONE	NONE	0.00000000000000e+00	0.00000000000000e+00
8	C0000008	SB	0.00000000000000e+00	NONE	NONE	0.00000000000000e+00	0.00000000000000e+00
9	C0000009	SB	0.00000000000000e+00	0.00000000e+00	NONE	0.00000000000000e+00	0.00000000000000e+00
10	C0000010	SB	1.34110805171553e+11	NONE	NONE	0.00000000000000e+00	0.00000000000000e+00
11	C0000011	LL	0.00000000000000e+00	0.00000000e+00	NONE	0.00000000000000e+00	0.00000000000000e+00
12	C0000012	SB	0.00000000000000e+00	NONE	NONE	0.00000000000000e+00	0.00000000000000e+00
13	C0000013	LL	0.00000000000000e+00	0.00000000e+00	NONE	0.00000000000000e+00	0.00000000000000e+00
14	C0000014	SB	1.44200546058582e+11	0.00000000e+00	NONE	0.00000000000000e+00	0.00000000000000e+00
15	C0000015	SB	0.00000000000000e+00	0.00000000e+00	NONE	0.00000000000000e+00	0.00000000000000e+00
16	C0000016	SB	-0.00000000000000e+00	NONE	NONE	0.00000000000000e+00	0.00000000000000e+00
17	C0000017	SB	0.00000000000000e+00	0.00000000e+00	NONE	0.00000000000000e+00	0.00000000000000e+00
18	C0000018	SB	-0.00000000000000e+00	NONE	NONE	0.00000000000000e+00	0.00000000000000e+00
19	C0000019	SB	-0.00000000000000e+00	NONE	NONE	0.00000000000000e+00	0.00000000000000e+00
20	C0000020	SB	-0.00000000000000e+00	NONE	NONE	0.00000000000000e+00	0.00000000000000e+00
21	C0000021	SB	-0.00000000000000e+00	NONE	NONE	0.00000000000000e+00	0.00000000000000e+00
22	C0000022	SB	0.00000000000000e+00	NONE	NONE	0.00000000000000e+00	0.00000000000000e+00
23	C0000023	SB	0.00000000000000e+00	NONE	NONE	0.00000000000000e+00	0.00000000000000e+00
24	C0000024	SB	-0.00000000000000e+00	NONE	NONE	0.00000000000000e+00	0.00000000000000e+00
25	C0000025	SB	0.00000000000000e+00	NONE	NONE	0.00000000000000e+00	0.00000000000000e+00
26	C0000026	LL	0.00000000000000e+00	0.00000000e+00	NONE	0.00000000000000e+00	0.00000000000000e+00
27	C0000027	SB	-0.00000000000000e+00	NONE	NONE	0.00000000000000e+00	0.00000000000000e+00
28	C0000028	SB	0.00000000000000e+00	0.00000000e+00	NONE	0.00000000000000e+00	0.00000000000000e+00
29	C0000029	SB	0.00000000000000e+00	NONE	NONE	0.00000000000000e+00	0.00000000000000e+00
30	C0000030	SB	-0.00000000000000e+00	0.00000000e+00	NONE	0.00000000000000e+00	0.00000000000000e+00
31	C0000031	LL	0.00000000000000e+00	0.00000000e+00	NONE	0.00000000000000e+00	0.00000000000000e+00
32	C0000032	SB	0.00000000000000e+00	0.00000000e+00	NONE	0.00000000000000e+00	0.00000000000000e+00
33	C0000033	SB	0.00000000000000e+00	NONE	NONE	0.00000000000000e+00	0.00000000000000e+00
34	C0000034	SB	0.00000000000000e+00	NONE	NONE	0.00000000000000e+00	0.00000000000000e+00
35	C0000035	SB	0.00000000000000e+00	NONE	NONE	0.00000000000000e+00	0.00000000000000e+00
36	C0000036	SB	0.00000000000000e+00	NONE	NONE	0.00000000000000e+00	0.00000000000000e+00
37	C0000037	SB	-1.05402848635147e+10	NONE	NONE	0.00000000000000e+00	0.00000000000000e+00
38	C0000038	SB	0.00000000000000e+00	NONE	NONE	0.00000000000000e+00	0.00000000000000e+00
39	C0000039	SB	1.66781884893276e+11	NONE	NONE	0.00000000000000e+00	0.00000000000000e+00
40	C0000040	SB	0.00000000000000e+00	NONE	NONE	0.00000000000000e+00	0.00000000000000e+00
41	C0000041	SB	-0.00000000000000e+00	NONE	NONE	0.00000000000000e+00	0.00000000000000e+00
42	C0000042	SB	0.00000000000000e+00	NONE	NONE	0.00000000000000e+00	0.00000000000000e+00
43	C0000043	SB	0.00000000000000e+00	NONE	NONE	0.00000000000000e+00	0.00000000000000e+00
44	C0000044	SB	1.44200546058582e+11	NONE	NONE	0.00000000000000e+00	0.00000000000000e+00
45	C0000045	SB	-2.60684352755897e+10	NONE	NONE	0.00000000000000e+00	0.00000000000000e+00
46	C0000046	SB	-2.99338197122179e+10	NONE	NONE	0.00000000000000e+00	0.00000000000000e+00
47	C0000047	SB	0.00000000000000e+00	NONE	NONE	0.00000000000000e+00	0.00000000000000e+00
48	C0000048	SB	-9.95869639116647e-01	NONE	NONE	0.00000000000000e+00	0.00000000000000e+00
49	C0000049	SB	-1.22678687280035e+10	NONE	NONE	0.00000000000000e+00	0.00000000000000e+00
50	C0000050	SB	5.53892196433869e-01	NONE	NONE	0.00000000000000e+00	0.00000000000000e+00
51	C0000051	SB	4.07654291706615e-01	NONE	NONE	0.00000000000000e+00	0.00000000000000e+00
52	C0000052	SB	-4.15628453506229e+11	NONE	NONE	0.00000000000000e+00	0.00000000000000e+00
53	C0000053	SB	3.73407603944451e-01	NONE	NONE	0.00000000000000e+00	0.00000000000000e+00
54	C0000054	SB	5.72810696151426e-01	NONE	NONE	0.00000000000000e+00	0.00000000000000e+00
55	C0000055	SB	1.68727404444811e-01	NONE	NONE	0.00000000000000e+00	0.00000000000000e+00
56	C0000056	SB	3.58892808889621e-01	NONE	NONE	0.00000000000000e+00	0.00000000000000e+00
57	C0000057	SB	5.23282052395434e-01	NONE	NONE	0.00000000000000e+00	0.00000000000000e+00
58	C0000058	SB	1.08086180219319e-01	NONE	NONE	0.00000000000000e+00	0.00000000000000e+00
59	C0000059	SB	2.35746467731304e-01	NONE	NONE	0.00000000000000e+00	0.00000000000000e+00
60	C0000060	SB	0.00000000000000e+00	NONE	NONE	0.00000000000000e+00	0.00000000000000e+00
61	C0000061	SB	0.00000000000000e+00	NONE	NONE	0.00000000000000e+00	0.00000000000000e+00
62	C0000062	SB	1.09335965648625e+11	NONE	NONE	0.00000000000000e+00	0.00000000000000e+00
63	C0000063	SB	0.00000000000000e+00	NONE	NONE	0.00000000000000e+00	0.00000000000000e+00
64	C0000064	LL	0.00000000000000e+00	0.00000000e+00	NONE	0.00000000000000e+00	0.00000000000000e+00
65	C0000065	LL	0.00000000000000e+00	0.00000000e+00	NONE	0.00000000000000e+00	0.00000000000000e+00
66	C0000066	LL	0.00000000000000e+00	0.00000000e+00	NONE	0.00000000000000e+00	0.00000000000000e+00
67	C0000067	LL	0.00000000000000e+00	0.00000000e+00	NONE	0.00000000000000e+00	0.00000000000000e+00
68	C0000068	SB	0.00000000000000e+00	0.00000000e+00	NONE	0.00000000000000e+00	0.00000000000000e+00
69	C0000069	SB	0.00000000000000e+00	0.00000000e+00	NONE	0.00000000000000e+00	0.00000000000000e+00
70	C0000070	SB	-0.00000000000000e+00	NONE	NONE	0.00000000000000e+00	0.00000000000000e+00
71	C0000071	SB	-0.00000000000000e+00	0.00000000e+00	NONE	0.00000000000000e+00	0.00000000000000e+00
72	C0000072	SB	2.65103628670302e-01	NONE	NONE	0.00000000000000e+00	0.00000000000000e+00
73	C0000073	LL	0.00000000000000e+00	0.00000000e+00	NONE	0.00000000000000e+00	0.00000000000000e+00
74	C0000074	LL	0.00000000000000e+00	0.00000000e+00	NONE	0.00000000000000e+00	0.00000000000000e+00
75	C0000075	LL	0.00000000000000e+00	0.00000000e+00	NONE	0.00000000000000e+00	0.00000000000000e+00
76	C0000076	LL	0.00000000000000e+00	0.00000000e+00	NONE	0.00000000000000e+00	0.00000000000000e+00
77	C0000077	LL	0.00000000000000e+00	0.00000000e+00	NONE	0.00000000000000e+00	0.00000000000000e+00
78	C0000078	LL	0.00000000000000e+00	0.00000000e+00	NONE	0.00000000000000e+00	0.00000000000000e+00
79	C0000079	LL	0.00000000000000e+00	0.00000000e+00	NONE	0.00000000000000e+00	0.00000000000000e+00
80	C0000080	SB	0.00000000000000e+00	NONE	NONE	0.00000000000000e+00	0.00000000000000e+00
81	C0000081	SB	0.00000000000000e+00	0.00000000e+00	NONE	0.00000000000000e+00	0.00000000000000e+00

82	C0000082	SB 0.00000000000000e+00	0.00000000e+00	NONE	0.00000000000000e+00	0.00000000000000e+00
83	C0000083	SB 0.00000000000000e+00	0.00000000e+00	NONE	0.00000000000000e+00	0.00000000000000e+00
84	C0000084	LL 0.00000000000000e+00	0.00000000e+00	NONE	0.00000000000000e+00	0.00000000000000e+00
85	C0000085	SB 0.00000000000000e+00	0.00000000e+00	NONE	0.00000000000000e+00	0.00000000000000e+00
86	C0000086	SB 2.48502845990322e-03	0.00000000e+00	NONE	0.00000000000000e+00	0.00000000000000e+00
87	C0000087	SB 0.00000000000000e+00	0.00000000e+00	NONE	0.00000000000000e+00	0.00000000000000e+00
88	C0000088	SB -0.00000000000000e+00	NONE	NONE	0.00000000000000e+00	0.00000000000000e+00
89	C0000089	LL 0.00000000000000e+00	0.00000000e+00	NONE	0.00000000000000e+00	0.00000000000000e+00
90	C0000090	SB -0.00000000000000e+00	NONE	NONE	0.00000000000000e+00	0.00000000000000e+00
91	C0000091	SB 0.00000000000000e+00	0.00000000e+00	NONE	0.00000000000000e+00	0.00000000000000e+00
92	C0000092	SB 0.00000000000000e+00	0.00000000e+00	NONE	0.00000000000000e+00	0.00000000000000e+00
93	C0000093	SB -0.00000000000000e+00	0.00000000e+00	NONE	0.00000000000000e+00	0.00000000000000e+00
94	C0000094	SB 1.18207846309196e+00	0.00000000e+00	NONE	0.00000000000000e+00	0.00000000000000e+00
95	C0000095	SB 1.18207846309196e+00	0.00000000e+00	NONE	0.00000000000000e+00	0.00000000000000e+00
96	C0000096	SB 1.18207846309196e+00	0.00000000e+00	NONE	0.00000000000000e+00	0.00000000000000e+00
97	C0000097	SB 1.18207846309196e+00	0.00000000e+00	NONE	0.00000000000000e+00	0.00000000000000e+00
98	C0000098	SB 1.18207846309196e+00	0.00000000e+00	NONE	0.00000000000000e+00	0.00000000000000e+00
99	C0000099	SB 1.18207846309196e+00	0.00000000e+00	NONE	0.00000000000000e+00	0.00000000000000e+00
100	C0000100	SB 1.18207846309196e+00	0.00000000e+00	NONE	0.00000000000000e+00	0.00000000000000e+00
101	C0000101	SB 0.00000000000000e+00	0.00000000e+00	NONE	0.00000000000000e+00	0.00000000000000e+00
102	C0000102	SB 1.18207846309196e+00	0.00000000e+00	NONE	0.00000000000000e+00	0.00000000000000e+00
103	C0000103	LL 0.00000000000000e+00	0.00000000e+00	NONE	0.00000000000000e+00	0.00000000000000e+00
104	C0000104	SB 0.00000000000000e+00	NONE	NONE	0.00000000000000e+00	0.00000000000000e+00
105	C0000105	LL 0.00000000000000e+00	0.00000000e+00	NONE	0.00000000000000e+00	0.00000000000000e+00
106	C0000106	LL 0.00000000000000e+00	0.00000000e+00	NONE	0.00000000000000e+00	0.00000000000000e+00
107	C0000107	LL 0.00000000000000e+00	0.00000000e+00	NONE	0.00000000000000e+00	0.00000000000000e+00
108	C0000108	LL 0.00000000000000e+00	0.00000000e+00	NONE	0.00000000000000e+00	0.00000000000000e+00
109	C0000109	LL 0.00000000000000e+00	0.00000000e+00	NONE	0.00000000000000e+00	0.00000000000000e+00
110	C0000110	LL 0.00000000000000e+00	0.00000000e+00	NONE	0.00000000000000e+00	0.00000000000000e+00
111	C0000111	LL 0.00000000000000e+00	0.00000000e+00	NONE	0.00000000000000e+00	0.00000000000000e+00
112	C0000112	LL 0.00000000000000e+00	0.00000000e+00	NONE	0.00000000000000e+00	0.00000000000000e+00
113	C0000113	LL 0.00000000000000e+00	0.00000000e+00	NONE	0.00000000000000e+00	0.00000000000000e+00
114	C0000114	SB -0.00000000000000e+00	0.00000000e+00	NONE	0.00000000000000e+00	0.00000000000000e+00
115	C0000115	SB 3.13328408055491e-04	0.00000000e+00	NONE	0.00000000000000e+00	0.00000000000000e+00
116	C0000116	SB 7.70658076413308e-04	0.00000000e+00	NONE	0.00000000000000e+00	0.00000000000000e+00
117	C0000117	SB 1.54107863180578e-02	NONE	NONE	0.00000000000000e+00	0.00000000000000e+00
118	C0000118	SB 3.47929023474919e-05	NONE	NONE	0.00000000000000e+00	0.00000000000000e+00
119	C0000119	SB 8.08390082205510e-05	NONE	NONE	0.00000000000000e+00	0.00000000000000e+00
120	C0000120	SB 1.28318704507009e-04	NONE	NONE	0.00000000000000e+00	0.00000000000000e+00
121	C0000121	SB 2.57181988925586e-04	NONE	NONE	0.00000000000000e+00	0.00000000000000e+00
122	C0000122	SB 2.17320263216948e-04	NONE	NONE	0.00000000000000e+00	0.00000000000000e+00
123	C0000123	SB 0.00000000000000e+00	NONE	NONE	0.00000000000000e+00	0.00000000000000e+00
124	C0000124	SB 2.96555002775001e-04	NONE	NONE	0.00000000000000e+00	0.00000000000000e+00
125	C0000125	LL 1.68357142837536e-02	0.00000000e+00	NONE	5.13967144641609e+03	0.00000000000000e+00
126	C0000126	SB 1.00939180221817e-01	NONE	NONE	0.00000000000000e+00	0.00000000000000e+00
127	C0000127	SB 0.00000000000000e+00	NONE	NONE	0.00000000000000e+00	0.00000000000000e+00
128	C0000128	SB 0.00000000000000e+00	NONE	NONE	0.00000000000000e+00	0.00000000000000e+00
129	C0000129	SB 0.00000000000000e+00	NONE	NONE	0.00000000000000e+00	0.00000000000000e+00
130	C0000130	SB 0.00000000000000e+00	NONE	NONE	0.00000000000000e+00	0.00000000000000e+00
131	C0000131	SB 6.49467510486577e-02	NONE	NONE	0.00000000000000e+00	0.00000000000000e+00
132	C0000132	SB 0.00000000000000e+00	NONE	NONE	0.00000000000000e+00	0.00000000000000e+00
133	C0000133	SB 0.00000000000000e+00	NONE	NONE	0.00000000000000e+00	0.00000000000000e+00
134	C0000134	SB 0.00000000000000e+00	NONE	NONE	0.00000000000000e+00	0.00000000000000e+00
135	C0000135	SB 0.00000000000000e+00	NONE	NONE	0.00000000000000e+00	0.00000000000000e+00
136	C0000136	SB 0.00000000000000e+00	NONE	NONE	0.00000000000000e+00	0.00000000000000e+00
137	C0000137	SB 0.00000000000000e+00	NONE	NONE	0.00000000000000e+00	0.00000000000000e+00
138	C0000138	SB 0.00000000000000e+00	NONE	NONE	0.00000000000000e+00	0.00000000000000e+00
139	C0000139	SB 0.00000000000000e+00	NONE	NONE	0.00000000000000e+00	0.00000000000000e+00
140	C0000140	SB 0.00000000000000e+00	NONE	NONE	0.00000000000000e+00	0.00000000000000e+00
141	C0000141	SB 0.00000000000000e+00	NONE	NONE	0.00000000000000e+00	0.00000000000000e+00
142	C0000142	SB 0.00000000000000e+00	NONE	NONE	0.00000000000000e+00	0.00000000000000e+00
143	C0000143	SB 0.00000000000000e+00	NONE	NONE	0.00000000000000e+00	0.00000000000000e+00
144	C0000144	SB -0.00000000000000e+00	NONE	NONE	0.00000000000000e+00	0.00000000000000e+00
145	C0000145	SB 1.22678687285069e+10	NONE	NONE	0.00000000000000e+00	0.00000000000000e+00
146	C0000146	SB -0.00000000000000e+00	NONE	NONE	0.00000000000000e+00	0.00000000000000e+00
147	C0000147	LL 0.00000000000000e+00	0.00000000e+00	NONE	0.00000000000000e+00	0.00000000000000e+00
148	C0000148	LL 0.00000000000000e+00	0.00000000e+00	NONE	0.00000000000000e+00	0.00000000000000e+00
149	C0000149	SB -0.00000000000000e+00	NONE	NONE	0.00000000000000e+00	0.00000000000000e+00
150	C0000150	SB 0.00000000000000e+00	NONE	NONE	0.00000000000000e+00	0.00000000000000e+00
151	C0000151	SB 0.00000000000000e+00	NONE	NONE	0.00000000000000e+00	0.00000000000000e+00
152	C0000152	LL 0.00000000000000e+00	0.00000000e+00	NONE	0.00000000000000e+00	0.00000000000000e+00
153	C0000153	SB 0.00000000000000e+00	0.00000000e+00	NONE	0.00000000000000e+00	0.00000000000000e+00
154	C0000154	SB 0.00000000000000e+00	NONE	NONE	0.00000000000000e+00	0.00000000000000e+00
155	C0000155	SB 0.00000000000000e+00	NONE	NONE	0.00000000000000e+00	0.00000000000000e+00
156	C0000156	SB 0.00000000000000e+00	NONE	NONE	0.00000000000000e+00	0.00000000000000e+00
157	C0000157	LL 0.00000000000000e+00	0.00000000e+00	NONE	0.00000000000000e+00	0.00000000000000e+00
158	C0000158	SB 0.00000000000000e+00	0.00000000e+00	NONE	0.00000000000000e+00	0.00000000000000e+00
159	C0000159	SB 6.53392876399171e+09	0.00000000e+00	NONE	0.00000000000000e+00	0.00000000000000e+00
160	C0000160	LL 0.00000000000000e+00	0.00000000e+00	NONE	0.00000000000000e+00	0.00000000000000e+00
161	C0000161	SB 0.00000000000000e+00	0.00000000e+00	NONE	0.00000000000000e+00	0.00000000000000e+00
162	C0000162	LL 0.00000000000000e+00	0.00000000e+00	NONE	0.00000000000000e+00	0.00000000000000e+00
163	C0000163	SB -0.00000000000000e+00	0.00000000e+00	NONE	0.00000000000000e+00	0.00000000000000e+00
164	C0000164	LL 0.00000000000000e+00	0.00000000e+00	NONE	0.00000000000000e+00	0.00000000000000e+00
165	C0000165	SB -8.86510729266539e-03	NONE	NONE	0.00000000000000e+00	0.00000000000000e+00
166	C0000166	LL 0.00000000000000e+00	0.00000000e+00	NONE	0.00000000000000e+00	0.00000000000000e+00
167	C0000167	SB -0.00000000000000e+00	0.00000000e+00	NONE	0.00000000000000e+00	0.00000000000000e+00
168	C0000168	SB -0.00000000000000e+00	NONE	NONE	0.00000000000000e+00	0.00000000000000e+00
169	C0000169	SB 7.77458070280346e-03	NONE	NONE	0.00000000000000e+00	0.00000000000000e+00
170	C0000170	SB 2.03645884315475e+09	0.00000000e+00	NONE	6.13108455367028e-10	0.00000000000000e+00
171	C0000171	SB 2.03645884315475e+09	0.00000000e+00	NONE	6.13108455367060e-10	0.00000000000000e+00
172	C0000172	SB 2.03645884315474e+09	0.00000000e+00	NONE	6.13108455366996e-10	0.00000000000000e+00
173	C0000173	SB 2.03645884315475e+09	0.00000000e+00	NONE	6.13108455367060e-10	0.00000000000000e+00
174	C0000174	SB 2.10700166620784e+09	0.00000000e+00	NONE	5.47496828854092e-10	0.00000000000000e+00
175	C0000175	SB 2.13362476705130e+09	0.00000000e+00	NONE	3.59302095116215e-10	0.00000000000000e+00
176	C0000176	SB 6.64376745765031e+09	0.00000000e+00	NONE	1.30999703009286e-10	0.00000000000000e+00
177	C0000177	SB 2.83696386612192e+09	0.00000000e+00	NONE	3.23928194109471e-10	0.00000000000000e+00
178	C0000178	SB 0.00000000000000e+00	0.00000000e+00	NONE	0.00000000000000e+00	0.00000000000000e+00
179	C0000179	LL 0.00000000000000e+00	0.00000000e+00	NONE	0.00000000000000e+00	0.00000000000000e+00

180	C0000180	SB	2.37402006585261e-03	NONE	NONE	0.00000000000000e+00	0.00000000000000e+00
181	C0000181	SB	-0.00000000000000e+00	NONE	NONE	0.00000000000000e+00	0.00000000000000e+00
182	C0000182	SB	0.00000000000000e+00	0.00000000e+00	NONE	0.00000000000000e+00	0.00000000000000e+00
183	C0000183	LL	0.00000000000000e+00	0.00000000e+00	NONE	0.00000000000000e+00	0.00000000000000e+00
184	C0000184	LL	0.00000000000000e+00	0.00000000e+00	NONE	0.00000000000000e+00	0.00000000000000e+00
185	C0000185	LL	0.00000000000000e+00	0.00000000e+00	NONE	0.00000000000000e+00	0.00000000000000e+00
186	C0000186	SB	0.00000000000000e+00	0.00000000e+00	NONE	0.00000000000000e+00	0.00000000000000e+00
187	C0000187	LL	0.00000000000000e+00	0.00000000e+00	NONE	0.00000000000000e+00	0.00000000000000e+00
188	C0000188	SB	0.00000000000000e+00	0.00000000e+00	NONE	0.00000000000000e+00	0.00000000000000e+00
189	C0000189	SB	6.63359725366489e-03	0.00000000e+00	NONE	0.00000000000000e+00	0.00000000000000e+00
190	C0000190	LL	0.00000000000000e+00	0.00000000e+00	NONE	0.00000000000000e+00	0.00000000000000e+00
191	C0000191	LL	0.00000000000000e+00	0.00000000e+00	NONE	0.00000000000000e+00	0.00000000000000e+00
192	C0000192	SB	-0.00000000000000e+00	0.00000000e+00	NONE	0.00000000000000e+00	0.00000000000000e+00
193	C0000193	SB	-0.00000000000000e+00	0.00000000e+00	NONE	0.00000000000000e+00	0.00000000000000e+00
194	C0000194	SB	-0.00000000000000e+00	0.00000000e+00	NONE	0.00000000000000e+00	0.00000000000000e+00
195	C0000195	SB	-0.00000000000000e+00	0.00000000e+00	NONE	0.00000000000000e+00	0.00000000000000e+00
196	C0000196	LL	0.00000000000000e+00	0.00000000e+00	NONE	0.00000000000000e+00	0.00000000000000e+00
197	C0000197	SB	0.00000000000000e+00	0.00000000e+00	NONE	0.00000000000000e+00	0.00000000000000e+00
198	C0000198	SB	6.63359725366489e-03	0.00000000e+00	NONE	0.00000000000000e+00	0.00000000000000e+00
199	C0000199	SB	6.53392876399171e+09	0.00000000e+00	NONE	0.00000000000000e+00	0.00000000000000e+00
200	C0000200	LL	0.00000000000000e+00	0.00000000e+00	NONE	0.00000000000000e+00	0.00000000000000e+00
201	C0000201	SB	1.44112424064288e-04	0.00000000e+00	NONE	0.00000000000000e+00	0.00000000000000e+00
202	C0000202	SB	7.70658076413308e-04	0.00000000e+00	NONE	0.00000000000000e+00	0.00000000000000e+00
203	C0000203	LL	0.00000000000000e+00	0.00000000e+00	NONE	0.00000000000000e+00	0.00000000000000e+00
204	C0000204	SB	0.00000000000000e+00	NONE	NONE	0.00000000000000e+00	0.00000000000000e+00
205	C0000205	SB	-0.00000000000000e+00	NONE	NONE	0.00000000000000e+00	0.00000000000000e+00
206	C0000206	SB	0.00000000000000e+00	NONE	NONE	0.00000000000000e+00	0.00000000000000e+00
207	C0000207	LL	0.00000000000000e+00	0.00000000e+00	NONE	0.00000000000000e+00	0.00000000000000e+00
208	C0000208	SB	-0.00000000000000e+00	0.00000000e+00	NONE	0.00000000000000e+00	0.00000000000000e+00
209	C0000209	LL	0.00000000000000e+00	0.00000000e+00	NONE	0.00000000000000e+00	0.00000000000000e+00
210	C0000210	LL	0.00000000000000e+00	0.00000000e+00	NONE	0.00000000000000e+00	0.00000000000000e+00
211	C0000211	SB	0.00000000000000e+00	0.00000000e+00	NONE	0.00000000000000e+00	0.00000000000000e+00
212	C0000212	LL	0.00000000000000e+00	0.00000000e+00	NONE	0.00000000000000e+00	0.00000000000000e+00
213	C0000213	SB	-0.00000000000000e+00	0.00000000e+00	NONE	0.00000000000000e+00	0.00000000000000e+00
214	C0000214	SB	-0.00000000000000e+00	NONE	NONE	0.00000000000000e+00	0.00000000000000e+00
215	C0000215	SB	0.00000000000000e+00	NONE	NONE	0.00000000000000e+00	0.00000000000000e+00
216	C0000216	SB	-0.00000000000000e+00	NONE	NONE	0.00000000000000e+00	0.00000000000000e+00
217	C0000217	LL	0.00000000000000e+00	0.00000000e+00	NONE	0.00000000000000e+00	0.00000000000000e+00
218	C0000218	LL	0.00000000000000e+00	0.00000000e+00	NONE	0.00000000000000e+00	0.00000000000000e+00
219	C0000219	LL	0.00000000000000e+00	0.00000000e+00	NONE	0.00000000000000e+00	0.00000000000000e+00
220	C0000220	SB	-6.39058046949904e-03	NONE	NONE	0.00000000000000e+00	0.00000000000000e+00
221	C0000221	LL	0.00000000000000e+00	0.00000000e+00	NONE	0.00000000000000e+00	0.00000000000000e+00
222	C0000222	SB	7.82846095735579e+09	0.00000000e+00	NONE	0.00000000000000e+00	0.00000000000000e+00
223	C0000223	SB	7.81357630476114e+09	0.00000000e+00	NONE	0.00000000000000e+00	0.00000000000000e+00
224	C0000224	SB	7.23654754949763e+09	0.00000000e+00	NONE	0.00000000000000e+00	0.00000000000000e+00
225	C0000225	SB	-0.00000000000000e+00	0.00000000e+00	NONE	0.00000000000000e+00	0.00000000000000e+00
226	C0000226	LL	0.00000000000000e+00	0.00000000e+00	NONE	0.00000000000000e+00	0.00000000000000e+00
227	C0000227	SB	0.00000000000000e+00	NONE	NONE	0.00000000000000e+00	0.00000000000000e+00
228	C0000228	SB	0.00000000000000e+00	0.00000000e+00	NONE	0.00000000000000e+00	0.00000000000000e+00
229	C0000229	SB	-0.00000000000000e+00	0.00000000e+00	NONE	0.00000000000000e+00	0.00000000000000e+00
230	C0000230	SB	2.25832052525916e-03	0.00000000e+00	NONE	0.00000000000000e+00	0.00000000000000e+00
231	C0000231	LL	0.00000000000000e+00	0.00000000e+00	NONE	0.00000000000000e+00	0.00000000000000e+00
232	C0000232	SB	4.47457779412649e+10	NONE	NONE	0.00000000000000e+00	0.00000000000000e+00
233	C0000233	SB	-1.31203158777902e+11	NONE	NONE	0.00000000000000e+00	0.00000000000000e+00
234	C0000234	LL	0.00000000000000e+00	0.00000000e+00	NONE	0.00000000000000e+00	0.00000000000000e+00
235	C0000235	LL	0.00000000000000e+00	0.00000000e+00	NONE	0.00000000000000e+00	0.00000000000000e+00
236	C0000236	SB	0.00000000000000e+00	NONE	NONE	0.00000000000000e+00	0.00000000000000e+00
237	C0000237	SB	0.00000000000000e+00	NONE	NONE	0.00000000000000e+00	0.00000000000000e+00
238	C0000238	SB	0.00000000000000e+00	NONE	NONE	0.00000000000000e+00	0.00000000000000e+00
239	C0000239	SB	0.00000000000000e+00	NONE	NONE	0.00000000000000e+00	0.00000000000000e+00
240	C0000240	SB	6.92008456840244e-01	NONE	NONE	0.00000000000000e+00	0.00000000000000e+00
241	C0000241	SB	0.00000000000000e+00	NONE	NONE	0.00000000000000e+00	0.00000000000000e+00
242	C0000242	SB	0.00000000000000e+00	NONE	NONE	0.00000000000000e+00	0.00000000000000e+00
243	C0000243	SB	-0.00000000000000e+00	0.00000000e+00	NONE	0.00000000000000e+00	0.00000000000000e+00
244	C0000244	LL	0.00000000000000e+00	0.00000000e+00	NONE	0.00000000000000e+00	0.00000000000000e+00
245	C0000245	LL	0.00000000000000e+00	0.00000000e+00	NONE	1.68784026755020e-06	0.00000000000000e+00
246	C0000246	SB	0.00000000000000e+00	0.00000000e+00	NONE	0.00000000000000e+00	0.00000000000000e+00
247	C0000247	SB	2.13259966222735e+00	0.00000000e+00	NONE	0.00000000000000e+00	0.00000000000000e+00
248	C0000248	SB	9.06113376573011e-03	0.00000000e+00	NONE	0.00000000000000e+00	0.00000000000000e+00
249	C0000249	LL	0.00000000000000e+00	0.00000000e+00	NONE	0.00000000000000e+00	0.00000000000000e+00
250	C0000250	SB	0.00000000000000e+00	NONE	NONE	0.00000000000000e+00	0.00000000000000e+00
251	C0000251	LL	-0.00000000000000e+00	0.00000000e+00	NONE	0.00000000000000e+00	0.00000000000000e+00
252	C0000252	SB	0.00000000000000e+00	NONE	NONE	0.00000000000000e+00	0.00000000000000e+00
253	C0000253	LL	0.00000000000000e+00	0.00000000e+00	NONE	0.00000000000000e+00	0.00000000000000e+00
254	C0000254	LL	0.00000000000000e+00	0.00000000e+00	NONE	0.00000000000000e+00	0.00000000000000e+00
255	C0000255	SB	0.00000000000000e+00	0.00000000e+00	NONE	0.00000000000000e+00	0.00000000000000e+00
256	C0000256	SB	0.00000000000000e+00	0.00000000e+00	NONE	0.00000000000000e+00	0.00000000000000e+00
257	C0000257	SB	0.00000000000000e+00	0.00000000e+00	NONE	0.00000000000000e+00	0.00000000000000e+00
258	C0000258	SB	-0.00000000000000e+00	NONE	NONE	0.00000000000000e+00	0.00000000000000e+00
259	C0000259	SB	0.00000000000000e+00	0.00000000e+00	NONE	0.00000000000000e+00	0.00000000000000e+00
260	C0000260	LL	0.00000000000000e+00	0.00000000e+00	NONE	0.00000000000000e+00	0.00000000000000e+00
261	C0000261	SB	0.00000000000000e+00	0.00000000e+00	NONE	0.00000000000000e+00	0.00000000000000e+00
262	C0000262	SB	-0.00000000000000e+00	0.00000000e+00	NONE	0.00000000000000e+00	0.00000000000000e+00
263	C0000263	LL	0.00000000000000e+00	0.00000000e+00	NONE	0.00000000000000e+00	0.00000000000000e+00
264	C0000264	SB	0.00000000000000e+00	0.00000000e+00	NONE	0.00000000000000e+00	0.00000000000000e+00
265	C0000265	SB	5.61651238016001e+09	0.00000000e+00	NONE	0.00000000000000e+00	0.00000000000000e+00
266	C0000266	SB	1.90109348583852e+09	0.00000000e+00	NONE	0.00000000000000e+00	0.00000000000000e+00
267	C0000267	SB	2.05441826536973e+10	0.00000000e+00	NONE	0.00000000000000e+00	0.00000000000000e+00
268	C0000268	SB	2.05441826536973e+10	0.00000000e+00	NONE	0.00000000000000e+00	0.00000000000000e+00
269	C0000269	SB	2.05441826536973e+10	0.00000000e+00	NONE	0.00000000000000e+00	0.00000000000000e+00
270	C0000270	SB	2.05441826536973e+10	0.00000000e+00	NONE	0.00000000000000e+00	0.00000000000000e+00
271	C0000271	SB	0.00000000000000e+00	0.00000000e+00	NONE	0.00000000000000e+00	0.00000000000000e+00
272	C0000272	SB	0.00000000000000e+00	0.00000000e+00	NONE	0.00000000000000e+00	0.00000000000000e+00
273	C0000273	SB	0.00000000000000e+00	0.00000000e+00	NONE	0.00000000000000e+00	0.00000000000000e+00
274	C0000274	SB	0.00000000000000e+00	0.00000000e+00	NONE	0.00000000000000e+00	0.00000000000000e+00
275	C0000275	SB	0.00000000000000e+00	0.00000000e+00	NONE	0.00000000000000e+00	0.00000000000000e+00
276	C0000276	SB	0.00000000000000e+00	NONE	NONE	0.00000000000000e+00	0.00000000000000e+00
277	C0000277	SB	0.00000000000000e+00	NONE	NONE	0.00000000000000e+00	0.00000000000000e+00

278	C0000278	SB -0.00000000000000e+00	NONE	NONE	0.00000000000000e+00	0.00000000000000e+00
279	C0000279	SB -0.00000000000000e+00	0.00000000e+00	NONE	0.00000000000000e+00	0.00000000000000e+00
280	C0000280	SB 0.00000000000000e+00	NONE	NONE	0.00000000000000e+00	0.00000000000000e+00
281	C0000281	SB 0.00000000000000e+00	0.00000000e+00	NONE	0.00000000000000e+00	0.00000000000000e+00
282	C0000282	LL 0.00000000000000e+00	0.00000000e+00	NONE	0.00000000000000e+00	0.00000000000000e+00
283	C0000283	LL 0.00000000000000e+00	0.00000000e+00	NONE	0.00000000000000e+00	0.00000000000000e+00
284	C0000284	LL 0.00000000000000e+00	0.00000000e+00	NONE	0.00000000000000e+00	0.00000000000000e+00
285	C0000285	LL 0.00000000000000e+00	0.00000000e+00	NONE	0.00000000000000e+00	0.00000000000000e+00
286	C0000286	LL 0.00000000000000e+00	0.00000000e+00	NONE	0.00000000000000e+00	0.00000000000000e+00
287	C0000287	SB 0.00000000000000e+00	0.00000000e+00	NONE	0.00000000000000e+00	0.00000000000000e+00
288	C0000288	SB 0.00000000000000e+00	0.00000000e+00	NONE	0.00000000000000e+00	0.00000000000000e+00
289	C0000289	SB -0.00000000000000e+00	0.00000000e+00	NONE	0.00000000000000e+00	0.00000000000000e+00
290	C0000290	SB 0.00000000000000e+00	0.00000000e+00	NONE	0.00000000000000e+00	0.00000000000000e+00
291	C0000291	SB -0.00000000000000e+00	0.00000000e+00	NONE	0.00000000000000e+00	0.00000000000000e+00
292	C0000292	SB 0.00000000000000e+00	0.00000000e+00	NONE	0.00000000000000e+00	0.00000000000000e+00
293	C0000293	SB -0.00000000000000e+00	0.00000000e+00	NONE	0.00000000000000e+00	0.00000000000000e+00
294	C0000294	SB 0.00000000000000e+00	0.00000000e+00	NONE	0.00000000000000e+00	0.00000000000000e+00
295	C0000295	SB -0.00000000000000e+00	0.00000000e+00	NONE	0.00000000000000e+00	0.00000000000000e+00
296	C0000296	SB 0.00000000000000e+00	0.00000000e+00	NONE	0.00000000000000e+00	0.00000000000000e+00
297	C0000297	SB -0.00000000000000e+00	0.00000000e+00	NONE	0.00000000000000e+00	0.00000000000000e+00
298	C0000298	SB -0.00000000000000e+00	0.00000000e+00	NONE	0.00000000000000e+00	0.00000000000000e+00
299	C0000299	LL 0.00000000000000e+00	0.00000000e+00	NONE	0.00000000000000e+00	0.00000000000000e+00
300	C0000300	SB -0.00000000000000e+00	0.00000000e+00	NONE	0.00000000000000e+00	0.00000000000000e+00
301	C0000301	SB 0.00000000000000e+00	0.00000000e+00	NONE	0.00000000000000e+00	0.00000000000000e+00
302	C0000302	SB 0.00000000000000e+00	0.00000000e+00	NONE	0.00000000000000e+00	0.00000000000000e+00
303	C0000303	SB 0.00000000000000e+00	0.00000000e+00	NONE	0.00000000000000e+00	0.00000000000000e+00
304	C0000304	SB 0.00000000000000e+00	NONE	NONE	0.00000000000000e+00	0.00000000000000e+00
305	C0000305	SB 2.36415692618392e+00	NONE	NONE	0.00000000000000e+00	0.00000000000000e+00
306	C0000306	SB 0.00000000000000e+00	NONE	NONE	0.00000000000000e+00	0.00000000000000e+00
307	C0000307	SB 2.03645884315475e+09	NONE	NONE	0.00000000000000e+00	0.00000000000000e+00
308	C0000308	SB 2.03645884315475e+09	NONE	NONE	0.00000000000000e+00	0.00000000000000e+00
309	C0000309	SB 2.03645884315474e+09	NONE	NONE	0.00000000000000e+00	0.00000000000000e+00
310	C0000310	SB 2.03645884315475e+09	NONE	NONE	0.00000000000000e+00	0.00000000000000e+00
311	C0000311	SB 2.10700166620784e+09	NONE	NONE	0.00000000000000e+00	0.00000000000000e+00
312	C0000312	SB 2.13362476705130e+09	NONE	NONE	0.00000000000000e+00	0.00000000000000e+00
313	C0000313	SB 6.64376745765031e+09	NONE	NONE	0.00000000000000e+00	0.00000000000000e+00
314	C0000314	SB 2.83696386612192e+09	NONE	NONE	0.00000000000000e+00	0.00000000000000e+00
315	C0000315	LL 0.00000000000000e+00	0.00000000e+00	NONE	0.00000000000000e+00	0.00000000000000e+00
316	C0000316	LL 0.00000000000000e+00	0.00000000e+00	NONE	0.00000000000000e+00	0.00000000000000e+00
317	C0000317	SB 1.57518001955659e+09	0.00000000e+00	NONE	0.00000000000000e+00	0.00000000000000e+00
318	C0000318	SB 1.47239793211233e+09	0.00000000e+00	NONE	0.00000000000000e+00	0.00000000000000e+00
319	C0000319	SB 0.00000000000000e+00	0.00000000e+00	NONE	0.00000000000000e+00	0.00000000000000e+00
320	C0000320	SB 1.56003400387094e+09	0.00000000e+00	NONE	0.00000000000000e+00	0.00000000000000e+00
321	C0000321	SB 0.00000000000000e+00	0.00000000e+00	NONE	0.00000000000000e+00	0.00000000000000e+00
322	C0000322	SB 0.00000000000000e+00	0.00000000e+00	NONE	0.00000000000000e+00	0.00000000000000e+00
323	C0000323	SB 0.00000000000000e+00	0.00000000e+00	NONE	0.00000000000000e+00	0.00000000000000e+00
324	C0000324	SB 2.79615745992000e+09	0.00000000e+00	NONE	0.00000000000000e+00	0.00000000000000e+00
325	C0000325	SB 0.00000000000000e+00	0.00000000e+00	NONE	0.00000000000000e+00	0.00000000000000e+00
326	C0000326	SB 3.13882183530324e+09	0.00000000e+00	NONE	5.74229282990675e-10	0.00000000000000e+00
327	C0000327	SB 2.34892352070190e+09	0.00000000e+00	NONE	4.20469080077508e-10	0.00000000000000e+00
328	C0000328	SB 1.62319748065204e+09	0.00000000e+00	NONE	8.05156781406439e-10	0.00000000000000e+00
329	C0000329	LL 0.00000000000000e+00	0.00000000e+00	NONE	0.00000000000000e+00	0.00000000000000e+00
330	C0000330	LL 0.00000000000000e+00	0.00000000e+00	NONE	4.08654947594839e+09	0.00000000000000e+00
331	C0000331	SB 4.44164686907656e+10	NONE	NONE	0.00000000000000e+00	0.00000000000000e+00
332	C0000332	SB 3.99301155846531e+09	0.00000000e+00	NONE	0.00000000000000e+00	0.00000000000000e+00
333	C0000333	SB 3.99301155846531e+09	NONE	NONE	0.00000000000000e+00	0.00000000000000e+00
334	C0000334	SB 3.99301155846531e+09	0.00000000e+00	NONE	0.00000000000000e+00	0.00000000000000e+00
335	C0000335	SB 3.99301155846531e+09	NONE	NONE	0.00000000000000e+00	0.00000000000000e+00
336	C0000336	SB 3.99301155846531e+09	0.00000000e+00	NONE	0.00000000000000e+00	0.00000000000000e+00
337	C0000337	SB 3.99301155846531e+09	NONE	NONE	0.00000000000000e+00	0.00000000000000e+00
338	C0000338	SB 3.99301155846531e+09	0.00000000e+00	NONE	0.00000000000000e+00	0.00000000000000e+00
339	C0000339	SB 3.99301155846531e+09	NONE	NONE	0.00000000000000e+00	0.00000000000000e+00
340	C0000340	SB -1.56003400387061e+09	NONE	NONE	0.00000000000000e+00	0.00000000000000e+00
341	C0000341	LL 0.00000000000000e+00	0.00000000e+00	NONE	0.00000000000000e+00	0.00000000000000e+00
342	C0000342	SB 5.48250273928282e+09	0.00000000e+00	NONE	0.00000000000000e+00	0.00000000000000e+00
343	C0000343	SB 5.48250273928282e+09	NONE	NONE	0.00000000000000e+00	0.00000000000000e+00
344	C0000344	SB -4.04883829813941e+09	NONE	NONE	0.00000000000000e+00	0.00000000000000e+00
345	C0000345	LL 0.00000000000000e+00	0.00000000e+00	NONE	0.00000000000000e+00	0.00000000000000e+00
346	C0000346	SB 9.50471793657878e+09	0.00000000e+00	NONE	0.00000000000000e+00	0.00000000000000e+00
347	C0000347	SB 9.50471793657878e+09	NONE	NONE	0.00000000000000e+00	0.00000000000000e+00
348	C0000348	SB 4.99457524597976e+09	NONE	NONE	0.00000000000000e+00	0.00000000000000e+00
349	C0000349	LL 0.00000000000000e+00	0.00000000e+00	NONE	0.00000000000000e+00	0.00000000000000e+00
350	C0000350	LL 0.00000000000000e+00	0.00000000e+00	NONE	2.14748706463151e+10	0.00000000000000e+00
351	C0000351	SB -0.00000000000000e+00	NONE	NONE	0.00000000000000e+00	0.00000000000000e+00
352	C0000352	SB -9.65039818951435e+09	NONE	NONE	0.00000000000000e+00	0.00000000000000e+00
353	C0000353	LL 0.00000000000000e+00	0.00000000e+00	NONE	0.00000000000000e+00	0.00000000000000e+00
354	C0000354	SB 1.34572017810427e+10	0.00000000e+00	NONE	0.00000000000000e+00	0.00000000000000e+00
355	C0000355	SB 1.34572017810427e+10	NONE	NONE	0.00000000000000e+00	0.00000000000000e+00
356	C0000356	SB 1.62941656471647e+10	NONE	NONE	0.00000000000000e+00	0.00000000000000e+00
357	C0000357	LL 0.00000000000000e+00	0.00000000e+00	NONE	0.00000000000000e+00	0.00000000000000e+00
358	C0000358	SB 6.02947040162006e+09	NONE	NONE	0.00000000000000e+00	0.00000000000000e+00
359	C0000359	SB 3.99301155846531e+09	0.00000000e+00	NONE	2.48584079719028e-10	0.00000000000000e+00
360	C0000360	SB 3.99301155846531e+09	NONE	NONE	0.00000000000000e+00	0.00000000000000e+00
361	C0000361	SB 3.99301155846531e+09	0.00000000e+00	NONE	2.48584079719036e-10	0.00000000000000e+00
362	C0000362	SB 3.99301155846531e+09	NONE	NONE	0.00000000000000e+00	0.00000000000000e+00
363	C0000363	SB 3.99301155846531e+09	0.00000000e+00	NONE	2.48584079719028e-10	0.00000000000000e+00
364	C0000364	SB 3.99301155846531e+09	NONE	NONE	0.00000000000000e+00	0.00000000000000e+00
365	C0000365	SB 3.99301155846531e+09	0.00000000e+00	NONE	2.48584079719036e-10	0.00000000000000e+00
366	C0000366	SB 3.99301155846531e+09	NONE	NONE	0.00000000000000e+00	0.00000000000000e+00
367	C0000367	SB 5.48250273928282e+09	0.00000000e+00	NONE	2.27127548657035e-10	0.00000000000000e+00
368	C0000368	SB 5.48250273928282e+09	NONE	NONE	0.00000000000000e+00	0.00000000000000e+00
369	C0000369	SB 9.50471793657878e+09	0.00000000e+00	NONE	2.25175000148846e-10	0.00000000000000e+00
370	C0000370	SB 9.50471793657878e+09	NONE	NONE	0.00000000000000e+00	0.00000000000000e+00
371	C0000371	SB 0.00000000000000e+00	0.00000000e+00	NONE	0.00000000000000e+00	0.00000000000000e+00
372	C0000372	SB 0.00000000000000e+00	NONE	NONE	0.00000000000000e+00	0.00000000000000e+00
373	C0000373	SB 1.34572017810427e+10	0.00000000e+00	NONE	1.05518084403271e-10	0.00000000000000e+00
374	C0000374	SB 1.34572017810427e+10	NONE	NONE	0.00000000000000e+00	0.00000000000000e+00
375	C0000375	SB 0.00000000000000e+00	0.00000000e+00	NONE	0.00000000000000e+00	0.00000000000000e+00

376	C0000376	SB -0.00000000000000e+00	0.00000000e+00	NONE	0.00000000000000e+00	0.00000000000000e+00
377	C0000377	SB 0.00000000000000e+00	0.00000000e+00	NONE	5.74229282990675e-10	0.00000000000000e+00
378	C0000378	SB 0.00000000000000e+00	0.00000000e+00	NONE	4.20469080077508e-10	0.00000000000000e+00
379	C0000379	SB 0.00000000000000e+00	0.00000000e+00	NONE	8.05156781406439e-10	0.00000000000000e+00
380	C0000380	LL 0.00000000000000e+00	0.00000000e+00	NONE	0.00000000000000e+00	0.00000000000000e+00
381	C0000381	SB 0.00000000000000e+00	0.00000000e+00	NONE	0.00000000000000e+00	0.00000000000000e+00
382	C0000382	SB 4.67674575110463e+11	0.00000000e+00	NONE	0.00000000000000e+00	0.00000000000000e+00
383	C0000383	SB -0.00000000000000e+00	NONE	NONE	0.00000000000000e+00	0.00000000000000e+00
384	C0000384	LL 0.00000000000000e+00	0.00000000e+00	NONE	0.00000000000000e+00	0.00000000000000e+00
385	C0000385	SB -0.00000000000000e+00	0.00000000e+00	NONE	0.00000000000000e+00	0.00000000000000e+00
386	C0000386	SB 1.42005975274149e-01	0.00000000e+00	NONE	0.00000000000000e+00	0.00000000000000e+00
387	C0000387	LL 0.00000000000000e+00	0.00000000e+00	NONE	0.00000000000000e+00	0.00000000000000e+00
388	C0000388	LL 0.00000000000000e+00	0.00000000e+00	NONE	0.00000000000000e+00	0.00000000000000e+00
389	C0000389	SB 1.11086453283902e-01	NONE	NONE	0.00000000000000e+00	0.00000000000000e+00
390	C0000390	LL 0.00000000000000e+00	0.00000000e+00	NONE	0.00000000000000e+00	0.00000000000000e+00
391	C0000391	LL 0.00000000000000e+00	0.00000000e+00	NONE	0.00000000000000e+00	0.00000000000000e+00
392	C0000392	SB 0.00000000000000e+00	NONE	NONE	0.00000000000000e+00	0.00000000000000e+00
393	C0000393	LL 0.00000000000000e+00	0.00000000e+00	NONE	0.00000000000000e+00	0.00000000000000e+00
394	C0000394	LL 0.00000000000000e+00	0.00000000e+00	NONE	0.00000000000000e+00	0.00000000000000e+00
395	C0000395	SB 0.00000000000000e+00	NONE	NONE	0.00000000000000e+00	0.00000000000000e+00
396	C0000396	LL 0.00000000000000e+00	0.00000000e+00	NONE	0.00000000000000e+00	0.00000000000000e+00
397	C0000397	SB 2.49769249969460e-12	NONE	NONE	0.00000000000000e+00	0.00000000000000e+00
398	C0000398	SB 0.00000000000000e+00	0.00000000e+00	NONE	0.00000000000000e+00	0.00000000000000e+00
399	C0000399	SB 2.40650433070919e+00	0.00000000e+00	NONE	0.00000000000000e+00	0.00000000000000e+00
400	C0000400	SB 0.00000000000000e+00	0.00000000e+00	NONE	0.00000000000000e+00	0.00000000000000e+00
401	C0000401	SB -0.00000000000000e+00	0.00000000e+00	NONE	0.00000000000000e+00	0.00000000000000e+00
402	C0000402	LL 0.00000000000000e+00	0.00000000e+00	NONE	0.00000000000000e+00	0.00000000000000e+00
403	C0000403	SB 0.00000000000000e+00	0.00000000e+00	NONE	0.00000000000000e+00	0.00000000000000e+00
404	C0000404	LL 0.00000000000000e+00	0.00000000e+00	NONE	0.00000000000000e+00	0.00000000000000e+00
405	C0000405	LL 0.00000000000000e+00	0.00000000e+00	NONE	0.00000000000000e+00	0.00000000000000e+00
406	C0000406	SB 0.00000000000000e+00	0.00000000e+00	NONE	0.00000000000000e+00	0.00000000000000e+00
407	C0000407	SB 0.00000000000000e+00	0.00000000e+00	NONE	0.00000000000000e+00	0.00000000000000e+00
408	C0000408	SB 0.00000000000000e+00	0.00000000e+00	NONE	0.00000000000000e+00	0.00000000000000e+00
409	C0000409	SB -0.00000000000000e+00	0.00000000e+00	NONE	0.00000000000000e+00	0.00000000000000e+00
410	C0000410	SB 0.00000000000000e+00	0.00000000e+00	NONE	0.00000000000000e+00	0.00000000000000e+00
411	C0000411	SB 0.00000000000000e+00	0.00000000e+00	NONE	0.00000000000000e+00	0.00000000000000e+00
412	C0000412	SB 0.00000000000000e+00	NONE	NONE	0.00000000000000e+00	0.00000000000000e+00
413	C0000413	SB 0.00000000000000e+00	0.00000000e+00	NONE	0.00000000000000e+00	0.00000000000000e+00
414	C0000414	SB 0.00000000000000e+00	0.00000000e+00	NONE	0.00000000000000e+00	0.00000000000000e+00
415	C0000415	SB 2.47365827858315e+09	0.00000000e+00	NONE	0.00000000000000e+00	0.00000000000000e+00
416	C0000416	LL 0.00000000000000e+00	0.00000000e+00	NONE	2.14748706463151e+10	0.00000000000000e+00
417	C0000417	LL 0.00000000000000e+00	0.00000000e+00	NONE	2.14748706463151e+10	0.00000000000000e+00
418	C0000418	SB 0.00000000000000e+00	0.00000000e+00	NONE	0.00000000000000e+00	0.00000000000000e+00
419	C0000419	SB 0.00000000000000e+00	0.00000000e+00	NONE	0.00000000000000e+00	0.00000000000000e+00
420	C0000420	SB 0.00000000000000e+00	0.00000000e+00	NONE	0.00000000000000e+00	0.00000000000000e+00
421	C0000421	SB 0.00000000000000e+00	0.00000000e+00	NONE	0.00000000000000e+00	0.00000000000000e+00
422	C0000422	SB 0.00000000000000e+00	0.00000000e+00	NONE	0.00000000000000e+00	0.00000000000000e+00
423	C0000423	SB 2.47365827858315e+09	0.00000000e+00	NONE	0.00000000000000e+00	0.00000000000000e+00
424	C0000424	SB -0.00000000000000e+00	0.00000000e+00	NONE	0.00000000000000e+00	0.00000000000000e+00
425	C0000425	SB 0.00000000000000e+00	0.00000000e+00	NONE	0.00000000000000e+00	0.00000000000000e+00
426	C0000426	SB 0.00000000000000e+00	0.00000000e+00	NONE	0.00000000000000e+00	0.00000000000000e+00
427	C0000427	SB 0.00000000000000e+00	0.00000000e+00	NONE	0.00000000000000e+00	0.00000000000000e+00
428	C0000428	SB -0.00000000000000e+00	0.00000000e+00	NONE	0.00000000000000e+00	0.00000000000000e+00
429	C0000429	SB 0.00000000000000e+00	NONE	NONE	0.00000000000000e+00	0.00000000000000e+00
430	C0000430	LL 0.00000000000000e+00	0.00000000e+00	NONE	0.00000000000000e+00	0.00000000000000e+00
431	C0000431	SB 0.00000000000000e+00	0.00000000e+00	NONE	0.00000000000000e+00	0.00000000000000e+00
432	C0000432	SB 0.00000000000000e+00	0.00000000e+00	NONE	0.00000000000000e+00	0.00000000000000e+00
433	C0000433	LL 0.00000000000000e+00	0.00000000e+00	NONE	0.00000000000000e+00	0.00000000000000e+00
434	C0000434	LL 0.00000000000000e+00	0.00000000e+00	NONE	0.00000000000000e+00	0.00000000000000e+00
435	C0000435	LL 0.00000000000000e+00	0.00000000e+00	NONE	0.00000000000000e+00	0.00000000000000e+00
436	C0000436	LL 0.00000000000000e+00	0.00000000e+00	NONE	0.00000000000000e+00	0.00000000000000e+00
437	C0000437	SB -2.28785848116146e+10	NONE	NONE	0.00000000000000e+00	0.00000000000000e+00
438	C0000438	SB -8.64573808366372e+10	NONE	NONE	0.00000000000000e+00	0.00000000000000e+00
439	C0000439	SB 0.00000000000000e+00	0.00000000e+00	NONE	0.00000000000000e+00	0.00000000000000e+00
440	C0000440	LL 0.00000000000000e+00	0.00000000e+00	NONE	0.00000000000000e+00	0.00000000000000e+00
441	C0000441	LL 0.00000000000000e+00	0.00000000e+00	NONE	0.00000000000000e+00	0.00000000000000e+00
442	C0000442	SB -0.00000000000000e+00	0.00000000e+00	NONE	0.00000000000000e+00	0.00000000000000e+00
443	C0000443	LL 0.00000000000000e+00	0.00000000e+00	NONE	0.00000000000000e+00	0.00000000000000e+00
444	C0000444	SB -0.00000000000000e+00	NONE	NONE	0.00000000000000e+00	0.00000000000000e+00
445	C0000445	LL 0.00000000000000e+00	0.00000000e+00	NONE	0.00000000000000e+00	0.00000000000000e+00
446	C0000446	SB 0.00000000000000e+00	0.00000000e+00	NONE	0.00000000000000e+00	0.00000000000000e+00
447	C0000447	SB 3.80514872176114e+00	NONE	NONE	0.00000000000000e+00	0.00000000000000e+00
448	C0000448	SB -3.80514872176114e+00	NONE	NONE	0.00000000000000e+00	0.00000000000000e+00
449	C0000449	SB 0.00000000000000e+00	NONE	NONE	0.00000000000000e+00	0.00000000000000e+00
450	C0000450	SB 0.00000000000000e+00	NONE	NONE	0.00000000000000e+00	0.00000000000000e+00
451	C0000451	SB 0.00000000000000e+00	NONE	NONE	0.00000000000000e+00	0.00000000000000e+00
452	C0000452	SB 0.00000000000000e+00	NONE	NONE	0.00000000000000e+00	0.00000000000000e+00
453	C0000453	SB 0.00000000000000e+00	NONE	NONE	0.00000000000000e+00	0.00000000000000e+00
454	C0000454	SB 5.60022549880920e+10	NONE	NONE	0.00000000000000e+00	0.00000000000000e+00
455	C0000455	SB 0.00000000000000e+00	NONE	NONE	0.00000000000000e+00	0.00000000000000e+00
456	C0000456	SB 0.00000000000000e+00	NONE	NONE	0.00000000000000e+00	0.00000000000000e+00
457	C0000457	SB 0.00000000000000e+00	NONE	NONE	0.00000000000000e+00	0.00000000000000e+00
458	C0000458	SB 0.00000000000000e+00	NONE	NONE	0.00000000000000e+00	0.00000000000000e+00
459	C0000459	SB 0.00000000000000e+00	NONE	NONE	0.00000000000000e+00	0.00000000000000e+00
460	C0000460	SB 0.00000000000000e+00	NONE	NONE	0.00000000000000e+00	0.00000000000000e+00
461	C0000461	SB -8.06662658205896e+09	NONE	NONE	0.00000000000000e+00	0.00000000000000e+00
462	C0000462	SB 0.00000000000000e+00	NONE	NONE	0.00000000000000e+00	0.00000000000000e+00
463	C0000463	SB 0.00000000000000e+00	NONE	NONE	0.00000000000000e+00	0.00000000000000e+00
464	C0000464	SB 0.00000000000000e+00	NONE	NONE	0.00000000000000e+00	0.00000000000000e+00
465	C0000465	SB 0.00000000000000e+00	NONE	NONE	0.00000000000000e+00	0.00000000000000e+00
466	C0000466	SB 0.00000000000000e+00	NONE	NONE	0.00000000000000e+00	0.00000000000000e+00
467	C0000467	SB 0.00000000000000e+00	NONE	NONE	0.00000000000000e+00	0.00000000000000e+00
468	C0000468	SB 0.00000000000000e+00	NONE	NONE	0.00000000000000e+00	0.00000000000000e+00
469	C0000469	SB 0.00000000000000e+00	NONE	NONE	0.00000000000000e+00	0.00000000000000e+00
470	C0000470	LL 0.00000000000000e+00	0.00000000e+00	NONE	0.00000000000000e+00	0.00000000000000e+00
471	C0000471	LL 0.00000000000000e+00	0.00000000e+00	NONE	0.00000000000000e+00	0.00000000000000e+00
472	C0000472	SB 0.00000000000000e+00	NONE	NONE	0.00000000000000e+00	0.00000000000000e+00
473	C0000473	LL 0.00000000000000e+00	0.00000000e+00	NONE	6.30810074355463e+04	0.00000000000000e+00

474	C0000474	SB 0.00000000000000e+00	0.00000000e+00	NONE	0.00000000000000e+00	0.00000000000000e+00
475	C0000475	LL 0.00000000000000e+00	0.00000000e+00	NONE	0.00000000000000e+00	0.00000000000000e+00
476	C0000476	SB 0.00000000000000e+00	0.00000000e+00	NONE	0.00000000000000e+00	0.00000000000000e+00
477	C0000477	SB -0.00000000000000e+00	0.00000000e+00	NONE	0.00000000000000e+00	0.00000000000000e+00
478	C0000478	SB -1.36837220879242e-04	NONE	NONE	0.00000000000000e+00	0.00000000000000e+00
479	C0000479	SB 0.00000000000000e+00	NONE	NONE	0.00000000000000e+00	0.00000000000000e+00
480	C0000480	LL 0.00000000000000e+00	0.00000000e+00	NONE	0.00000000000000e+00	0.00000000000000e+00
481	C0000481	SB 0.00000000000000e+00	0.00000000e+00	NONE	0.00000000000000e+00	0.00000000000000e+00
482	C0000482	LL 0.00000000000000e+00	0.00000000e+00	NONE	0.00000000000000e+00	0.00000000000000e+00
483	C0000483	LL 0.00000000000000e+00	0.00000000e+00	NONE	0.00000000000000e+00	0.00000000000000e+00
484	C0000484	LL 0.00000000000000e+00	0.00000000e+00	NONE	0.00000000000000e+00	0.00000000000000e+00
485	C0000485	LL 0.00000000000000e+00	0.00000000e+00	NONE	0.00000000000000e+00	0.00000000000000e+00
486	C0000486	SB -0.00000000000000e+00	NONE	NONE	0.00000000000000e+00	0.00000000000000e+00
487	C0000487	SB -0.00000000000000e+00	0.00000000e+00	NONE	0.00000000000000e+00	0.00000000000000e+00
488	C0000488	LL 0.00000000000000e+00	0.00000000e+00	NONE	0.00000000000000e+00	0.00000000000000e+00
489	C0000489	SB -0.00000000000000e+00	0.00000000e+00	NONE	0.00000000000000e+00	0.00000000000000e+00
490	C0000490	LL 0.00000000000000e+00	0.00000000e+00	NONE	0.00000000000000e+00	0.00000000000000e+00
491	C0000491	LL 0.00000000000000e+00	0.00000000e+00	NONE	0.00000000000000e+00	0.00000000000000e+00
492	C0000492	SB 0.00000000000000e+00	NONE	NONE	0.00000000000000e+00	0.00000000000000e+00
493	C0000493	SB 0.00000000000000e+00	0.00000000e+00	NONE	0.00000000000000e+00	0.00000000000000e+00
494	C0000494	SB 0.00000000000000e+00	0.00000000e+00	NONE	0.00000000000000e+00	0.00000000000000e+00
495	C0000495	SB 0.00000000000000e+00	NONE	NONE	0.00000000000000e+00	0.00000000000000e+00
496	C0000496	SB 0.00000000000000e+00	NONE	NONE	0.00000000000000e+00	0.00000000000000e+00
497	C0000497	SB 0.00000000000000e+00	NONE	NONE	0.00000000000000e+00	0.00000000000000e+00
498	C0000498	SB 0.00000000000000e+00	NONE	NONE	0.00000000000000e+00	0.00000000000000e+00
499	C0000499	SB 0.00000000000000e+00	NONE	NONE	0.00000000000000e+00	0.00000000000000e+00
500	C0000500	SB 0.00000000000000e+00	NONE	NONE	0.00000000000000e+00	0.00000000000000e+00
501	C0000501	SB 0.00000000000000e+00	NONE	NONE	0.00000000000000e+00	0.00000000000000e+00
502	C0000502	SB 0.00000000000000e+00	0.00000000e+00	NONE	0.00000000000000e+00	0.00000000000000e+00
503	C0000503	SB 0.00000000000000e+00	0.00000000e+00	NONE	0.00000000000000e+00	0.00000000000000e+00
504	C0000504	LL 0.00000000000000e+00	0.00000000e+00	NONE	0.00000000000000e+00	0.00000000000000e+00
505	C0000505	SB 0.00000000000000e+00	NONE	NONE	0.00000000000000e+00	0.00000000000000e+00
506	C0000506	SB 0.00000000000000e+00	NONE	NONE	0.00000000000000e+00	0.00000000000000e+00
507	C0000507	SB 2.36415692618392e+00	NONE	NONE	0.00000000000000e+00	0.00000000000000e+00
508	C0000508	SB 0.00000000000000e+00	NONE	NONE	0.00000000000000e+00	0.00000000000000e+00
509	C0000509	LL 0.00000000000000e+00	0.00000000e+00	NONE	5.13967144641609e+03	0.00000000000000e+00
510	C0000510	SB 1.68357142837536e-02	0.00000000e+00	NONE	0.00000000000000e+00	0.00000000000000e+00
511	C0000511	SB -0.00000000000000e+00	NONE	NONE	0.00000000000000e+00	0.00000000000000e+00
512	C0000512	SB 0.00000000000000e+00	NONE	NONE	0.00000000000000e+00	0.00000000000000e+00
513	C0000513	LL 0.00000000000000e+00	0.00000000e+00	NONE	0.00000000000000e+00	0.00000000000000e+00
514	C0000514	SB 0.00000000000000e+00	0.00000000e+00	NONE	0.00000000000000e+00	0.00000000000000e+00
515	C0000515	SB 0.00000000000000e+00	0.00000000e+00	NONE	0.00000000000000e+00	0.00000000000000e+00
516	C0000516	LL 0.00000000000000e+00	0.00000000e+00	NONE	0.00000000000000e+00	0.00000000000000e+00
517	C0000517	SB -0.00000000000000e+00	0.00000000e+00	NONE	0.00000000000000e+00	0.00000000000000e+00
518	C0000518	SB 1.39864439105195e+00	0.00000000e+00	NONE	0.00000000000000e+00	0.00000000000000e+00
519	C0000519	SB 0.00000000000000e+00	0.00000000e+00	NONE	0.00000000000000e+00	0.00000000000000e+00
520	C0000520	LL 0.00000000000000e+00	0.00000000e+00	NONE	0.00000000000000e+00	0.00000000000000e+00
521	C0000521	SB 7.30538035724813e-03	NONE	NONE	0.00000000000000e+00	0.00000000000000e+00
522	C0000522	SB -6.39058046949904e-03	NONE	NONE	0.00000000000000e+00	0.00000000000000e+00
523	C0000523	SB -0.00000000000000e+00	NONE	NONE	0.00000000000000e+00	0.00000000000000e+00
524	C0000524	SB -0.00000000000000e+00	0.00000000e+00	NONE	0.00000000000000e+00	0.00000000000000e+00
525	C0000525	SB 0.00000000000000e+00	0.00000000e+00	NONE	0.00000000000000e+00	0.00000000000000e+00
526	C0000526	LL 0.00000000000000e+00	0.00000000e+00	NONE	0.00000000000000e+00	0.00000000000000e+00
527	C0000527	SB -0.00000000000000e+00	0.00000000e+00	NONE	0.00000000000000e+00	0.00000000000000e+00
528	C0000528	LL 0.00000000000000e+00	0.00000000e+00	NONE	0.00000000000000e+00	0.00000000000000e+00
529	C0000529	LL 0.00000000000000e+00	0.00000000e+00	NONE	0.00000000000000e+00	0.00000000000000e+00
530	C0000530	LL 0.00000000000000e+00	0.00000000e+00	NONE	0.00000000000000e+00	0.00000000000000e+00
531	C0000531	SB -0.00000000000000e+00	0.00000000e+00	NONE	0.00000000000000e+00	0.00000000000000e+00
532	C0000532	SB -0.00000000000000e+00	0.00000000e+00	NONE	0.00000000000000e+00	0.00000000000000e+00
533	C0000533	SB -0.00000000000000e+00	0.00000000e+00	NONE	0.00000000000000e+00	0.00000000000000e+00
534	C0000534	SB 2.18671931296504e+10	NONE	NONE	0.00000000000000e+00	0.00000000000000e+00
535	C0000535	SB 0.00000000000000e+00	NONE	NONE	0.00000000000000e+00	0.00000000000000e+00
536	C0000536	SB 0.00000000000000e+00	0.00000000e+00	NONE	0.00000000000000e+00	0.00000000000000e+00
537	C0000537	SB -0.00000000000000e+00	0.00000000e+00	NONE	0.00000000000000e+00	0.00000000000000e+00
538	C0000538	SB -0.00000000000000e+00	0.00000000e+00	NONE	0.00000000000000e+00	0.00000000000000e+00
539	C0000539	SB 0.00000000000000e+00	0.00000000e+00	NONE	0.00000000000000e+00	0.00000000000000e+00
540	C0000540	SB 0.00000000000000e+00	0.00000000e+00	NONE	0.00000000000000e+00	0.00000000000000e+00
541	C0000541	SB -0.00000000000000e+00	0.00000000e+00	NONE	0.00000000000000e+00	0.00000000000000e+00
542	C0000542	SB -0.00000000000000e+00	0.00000000e+00	NONE	0.00000000000000e+00	0.00000000000000e+00
543	C0000543	SB 0.00000000000000e+00	0.00000000e+00	NONE	0.00000000000000e+00	0.00000000000000e+00
544	C0000544	LL 0.00000000000000e+00	0.00000000e+00	NONE	0.00000000000000e+00	0.00000000000000e+00
545	C0000545	SB -0.00000000000000e+00	NONE	NONE	0.00000000000000e+00	0.00000000000000e+00
546	C0000546	SB 0.00000000000000e+00	NONE	NONE	0.00000000000000e+00	0.00000000000000e+00
547	C0000547	SB -0.00000000000000e+00	NONE	NONE	0.00000000000000e+00	0.00000000000000e+00
548	C0000548	LL 0.00000000000000e+00	0.00000000e+00	NONE	0.00000000000000e+00	0.00000000000000e+00
549	C0000549	LL 6.63359725366489e-03	0.00000000e+00	NONE	6.30810074355463e+04	0.00000000000000e+00
550	C0000550	SB 0.00000000000000e+00	0.00000000e+00	NONE	0.00000000000000e+00	0.00000000000000e+00
551	C0000551	SB 0.00000000000000e+00	0.00000000e+00	NONE	0.00000000000000e+00	0.00000000000000e+00
552	C0000552	SB 0.00000000000000e+00	0.00000000e+00	NONE	0.00000000000000e+00	0.00000000000000e+00
553	C0000553	LL 0.00000000000000e+00	0.00000000e+00	NONE	0.00000000000000e+00	0.00000000000000e+00
554	C0000554	SB 0.00000000000000e+00	0.00000000e+00	NONE	0.00000000000000e+00	0.00000000000000e+00
555	C0000555	SB -0.00000000000000e+00	0.00000000e+00	NONE	0.00000000000000e+00	0.00000000000000e+00
556	C0000556	LL 0.00000000000000e+00	0.00000000e+00	NONE	0.00000000000000e+00	0.00000000000000e+00
557	C0000557	SB 2.03645884315475e+09	0.00000000e+00	NONE	0.00000000000000e+00	0.00000000000000e+00
558	C0000558	SB 2.03645884315475e+09	0.00000000e+00	NONE	0.00000000000000e+00	0.00000000000000e+00
559	C0000559	SB 2.03645884315474e+09	0.00000000e+00	NONE	0.00000000000000e+00	0.00000000000000e+00
560	C0000560	SB 2.03645884315475e+09	0.00000000e+00	NONE	0.00000000000000e+00	0.00000000000000e+00
561	C0000561	SB 2.10700166620784e+09	0.00000000e+00	NONE	0.00000000000000e+00	0.00000000000000e+00
562	C0000562	SB 2.13362476705130e+09	0.00000000e+00	NONE	0.00000000000000e+00	0.00000000000000e+00
563	C0000563	SB 6.64376745765031e+09	0.00000000e+00	NONE	0.00000000000000e+00	0.00000000000000e+00
564	C0000564	SB 2.83696386612192e+09	0.00000000e+00	NONE	0.00000000000000e+00	0.00000000000000e+00
565	C0000565	LL 0.00000000000000e+00	0.00000000e+00	NONE	0.00000000000000e+00	0.00000000000000e+00
566	C0000566	LL 0.00000000000000e+00	0.00000000e+00	NONE	0.00000000000000e+00	0.00000000000000e+00
567	C0000567	SB 0.00000000000000e+00	0.00000000e+00	NONE	0.00000000000000e+00	0.00000000000000e+00
568	C0000568	LL 0.00000000000000e+00	0.00000000e+00	NONE	0.00000000000000e+00	0.00000000000000e+00
569	C0000569	LL 0.00000000000000e+00	0.00000000e+00	NONE	0.00000000000000e+00	0.00000000000000e+00
570	C0000570	LL 0.00000000000000e+00	0.00000000e+00	NONE	0.00000000000000e+00	0.00000000000000e+00
571	C0000571	SB -0.00000000000000e+00	0.00000000e+00	NONE	0.00000000000000e+00	0.00000000000000e+00

572	C0000572	LL 0.00000000000000e+00	0.00000000e+00	NONE	0.00000000000000e+00	0.00000000000000e+00
573	C0000573	LL 0.00000000000000e+00	0.00000000e+00	NONE	0.00000000000000e+00	0.00000000000000e+00
574	C0000574	LL 0.00000000000000e+00	0.00000000e+00	NONE	0.00000000000000e+00	0.00000000000000e+00
575	C0000575	SB 0.00000000000000e+00	0.00000000e+00	NONE	0.00000000000000e+00	0.00000000000000e+00
576	C0000576	SB 0.00000000000000e+00	0.00000000e+00	NONE	0.00000000000000e+00	0.00000000000000e+00
577	C0000577	LL 0.00000000000000e+00	0.00000000e+00	NONE	0.00000000000000e+00	0.00000000000000e+00
578	C0000578	LL 0.00000000000000e+00	0.00000000e+00	NONE	0.00000000000000e+00	0.00000000000000e+00
579	C0000579	LL 0.00000000000000e+00	0.00000000e+00	NONE	0.00000000000000e+00	0.00000000000000e+00
580	C0000580	LL 0.00000000000000e+00	0.00000000e+00	NONE	0.00000000000000e+00	0.00000000000000e+00
581	C0000581	LL 0.00000000000000e+00	0.00000000e+00	NONE	0.00000000000000e+00	0.00000000000000e+00
582	C0000582	LL 0.00000000000000e+00	0.00000000e+00	NONE	0.00000000000000e+00	0.00000000000000e+00
583	C0000583	LL 0.00000000000000e+00	0.00000000e+00	NONE	0.00000000000000e+00	0.00000000000000e+00
584	C0000584	LL 0.00000000000000e+00	0.00000000e+00	NONE	0.00000000000000e+00	0.00000000000000e+00
585	C0000585	LL 0.00000000000000e+00	0.00000000e+00	NONE	0.00000000000000e+00	0.00000000000000e+00
586	C0000586	LL 0.00000000000000e+00	0.00000000e+00	NONE	0.00000000000000e+00	0.00000000000000e+00
587	C0000587	LL 0.00000000000000e+00	0.00000000e+00	NONE	0.00000000000000e+00	0.00000000000000e+00
588	C0000588	LL 0.00000000000000e+00	0.00000000e+00	NONE	0.00000000000000e+00	0.00000000000000e+00
589	C0000589	LL 0.00000000000000e+00	0.00000000e+00	NONE	0.00000000000000e+00	0.00000000000000e+00
590	C0000590	LL 0.00000000000000e+00	0.00000000e+00	NONE	0.00000000000000e+00	0.00000000000000e+00
591	C0000591	LL 0.00000000000000e+00	0.00000000e+00	NONE	0.00000000000000e+00	0.00000000000000e+00
592	C0000592	LL 0.00000000000000e+00	0.00000000e+00	NONE	0.00000000000000e+00	0.00000000000000e+00
593	C0000593	LL 0.00000000000000e+00	0.00000000e+00	NONE	0.00000000000000e+00	0.00000000000000e+00
594	C0000594	LL 0.00000000000000e+00	0.00000000e+00	NONE	0.00000000000000e+00	0.00000000000000e+00
595	C0000595	LL 0.00000000000000e+00	0.00000000e+00	NONE	0.00000000000000e+00	0.00000000000000e+00
596	C0000596	LL 0.00000000000000e+00	0.00000000e+00	NONE	0.00000000000000e+00	0.00000000000000e+00
597	C0000597	LL 0.00000000000000e+00	0.00000000e+00	NONE	0.00000000000000e+00	0.00000000000000e+00
598	C0000598	LL 0.00000000000000e+00	0.00000000e+00	NONE	0.00000000000000e+00	0.00000000000000e+00
599	C0000599	LL 0.00000000000000e+00	0.00000000e+00	NONE	0.00000000000000e+00	0.00000000000000e+00
600	C0000600	LL 0.00000000000000e+00	0.00000000e+00	NONE	0.00000000000000e+00	0.00000000000000e+00
601	C0000601	LL 0.00000000000000e+00	0.00000000e+00	NONE	0.00000000000000e+00	0.00000000000000e+00
602	C0000602	LL 0.00000000000000e+00	0.00000000e+00	NONE	0.00000000000000e+00	0.00000000000000e+00
603	C0000603	LL 0.00000000000000e+00	0.00000000e+00	NONE	0.00000000000000e+00	0.00000000000000e+00
604	C0000604	LL 0.00000000000000e+00	0.00000000e+00	NONE	0.00000000000000e+00	0.00000000000000e+00
605	C0000605	LL 0.00000000000000e+00	0.00000000e+00	NONE	0.00000000000000e+00	0.00000000000000e+00
606	C0000606	LL 0.00000000000000e+00	0.00000000e+00	NONE	0.00000000000000e+00	0.00000000000000e+00
607	C0000607	LL 0.00000000000000e+00	0.00000000e+00	NONE	0.00000000000000e+00	0.00000000000000e+00
608	C0000608	LL 0.00000000000000e+00	0.00000000e+00	NONE	0.00000000000000e+00	0.00000000000000e+00
609	C0000609	LL 0.00000000000000e+00	0.00000000e+00	NONE	0.00000000000000e+00	0.00000000000000e+00
610	C0000610	LL 0.00000000000000e+00	0.00000000e+00	NONE	0.00000000000000e+00	0.00000000000000e+00
611	C0000611	LL 0.00000000000000e+00	0.00000000e+00	NONE	0.00000000000000e+00	0.00000000000000e+00
612	C0000612	LL 0.00000000000000e+00	0.00000000e+00	NONE	0.00000000000000e+00	0.00000000000000e+00
613	C0000613	LL 0.00000000000000e+00	0.00000000e+00	NONE	0.00000000000000e+00	0.00000000000000e+00
614	C0000614	LL 0.00000000000000e+00	0.00000000e+00	NONE	0.00000000000000e+00	0.00000000000000e+00
615	C0000615	LL 0.00000000000000e+00	0.00000000e+00	NONE	0.00000000000000e+00	0.00000000000000e+00
616	C0000616	LL 0.00000000000000e+00	0.00000000e+00	NONE	0.00000000000000e+00	0.00000000000000e+00
617	C0000617	LL 0.00000000000000e+00	0.00000000e+00	NONE	0.00000000000000e+00	0.00000000000000e+00
618	C0000618	LL 0.00000000000000e+00	0.00000000e+00	NONE	0.00000000000000e+00	0.00000000000000e+00
619	C0000619	LL 0.00000000000000e+00	0.00000000e+00	NONE	0.00000000000000e+00	0.00000000000000e+00
620	C0000620	LL 0.00000000000000e+00	0.00000000e+00	NONE	0.00000000000000e+00	0.00000000000000e+00
621	C0000621	LL 0.00000000000000e+00	0.00000000e+00	NONE	0.00000000000000e+00	0.00000000000000e+00
622	C0000622	LL 0.00000000000000e+00	0.00000000e+00	NONE	0.00000000000000e+00	0.00000000000000e+00
623	C0000623	LL 0.00000000000000e+00	0.00000000e+00	NONE	0.00000000000000e+00	0.00000000000000e+00
624	C0000624	LL 0.00000000000000e+00	0.00000000e+00	NONE	0.00000000000000e+00	0.00000000000000e+00
625	C0000625	SB 0.00000000000000e+00	NONE	NONE	0.00000000000000e+00	0.00000000000000e+00
626	C0000626	LL 0.00000000000000e+00	0.00000000e+00	NONE	0.00000000000000e+00	0.00000000000000e+00
627	C0000627	LL 0.00000000000000e+00	0.00000000e+00	NONE	0.00000000000000e+00	0.00000000000000e+00
628	C0000628	LL 0.00000000000000e+00	0.00000000e+00	NONE	0.00000000000000e+00	0.00000000000000e+00
629	C0000629	SB 0.00000000000000e+00	NONE	NONE	0.00000000000000e+00	0.00000000000000e+00
630	C0000630	SB 0.00000000000000e+00	0.00000000e+00	NONE	0.00000000000000e+00	0.00000000000000e+00
631	C0000631	SB -0.00000000000000e+00	0.00000000e+00	NONE	0.00000000000000e+00	0.00000000000000e+00
632	C0000632	SB 0.00000000000000e+00	0.00000000e+00	NONE	0.00000000000000e+00	0.00000000000000e+00
633	C0000633	SB 0.00000000000000e+00	0.00000000e+00	NONE	0.00000000000000e+00	0.00000000000000e+00
634	C0000634	SB -0.00000000000000e+00	0.00000000e+00	NONE	0.00000000000000e+00	0.00000000000000e+00
635	C0000635	SB -0.00000000000000e+00	0.00000000e+00	NONE	0.00000000000000e+00	0.00000000000000e+00
636	C0000636	SB -0.00000000000000e+00	0.00000000e+00	NONE	0.00000000000000e+00	0.00000000000000e+00
637	C0000637	SB 5.61651238016001e+09	0.00000000e+00	NONE	0.00000000000000e+00	0.00000000000000e+00
638	C0000638	SB 1.90109348583852e+09	0.00000000e+00	NONE	0.00000000000000e+00	0.00000000000000e+00
639	C0000639	SB 2.05441826536973e+10	0.00000000e+00	NONE	0.00000000000000e+00	0.00000000000000e+00
640	C0000640	SB 2.05441826536973e+10	0.00000000e+00	NONE	0.00000000000000e+00	0.00000000000000e+00
641	C0000641	SB 2.05441826536973e+10	0.00000000e+00	NONE	0.00000000000000e+00	0.00000000000000e+00
642	C0000642	SB 2.05441826536973e+10	0.00000000e+00	NONE	0.00000000000000e+00	0.00000000000000e+00
643	C0000643	LL 0.00000000000000e+00	0.00000000e+00	NONE	0.00000000000000e+00	0.00000000000000e+00
644	C0000644	SB 0.00000000000000e+00	0.00000000e+00	NONE	0.00000000000000e+00	0.00000000000000e+00
645	C0000645	SB 0.00000000000000e+00	0.00000000e+00	NONE	0.00000000000000e+00	0.00000000000000e+00
646	C0000646	SB 1.22678687285069e+10	0.00000000e+00	NONE	0.00000000000000e+00	0.00000000000000e+00
647	C0000647	SB 1.22678687285069e+10	0.00000000e+00	NONE	0.00000000000000e+00	0.00000000000000e+00
648	C0000648	LL 0.00000000000000e+00	0.00000000e+00	NONE	0.00000000000000e+00	0.00000000000000e+00
649	C0000649	SB -0.00000000000000e+00	0.00000000e+00	NONE	0.00000000000000e+00	0.00000000000000e+00
650	C0000650	SB 1.22678687285069e+10	0.00000000e+00	NONE	0.00000000000000e+00	0.00000000000000e+00
651	C0000651	SB 0.00000000000000e+00	0.00000000e+00	NONE	0.00000000000000e+00	0.00000000000000e+00
652	C0000652	LL 0.00000000000000e+00	0.00000000e+00	NONE	0.00000000000000e+00	0.00000000000000e+00
653	C0000653	SB 0.00000000000000e+00	0.00000000e+00	NONE	0.00000000000000e+00	0.00000000000000e+00
654	C0000654	SB 0.00000000000000e+00	0.00000000e+00	NONE	0.00000000000000e+00	0.00000000000000e+00
655	C0000655	SB 5.40056063695085e-03	0.00000000e+00	NONE	0.00000000000000e+00	0.00000000000000e+00
656	C0000656	LL 5.40056063695085e-03	0.00000000e+00	NONE	1.37878179260560e+03	0.00000000000000e+00
657	C0000657	SB 0.00000000000000e+00	0.00000000e+00	NONE	0.00000000000000e+00	0.00000000000000e+00
658	C0000658	SB 0.00000000000000e+00	0.00000000e+00	NONE	0.00000000000000e+00	0.00000000000000e+00
659	C0000659	SB 0.00000000000000e+00	0.00000000e+00	NONE	0.00000000000000e+00	0.00000000000000e+00
660	C0000660	SB -0.00000000000000e+00	0.00000000e+00	NONE	0.00000000000000e+00	0.00000000000000e+00
661	C0000661	SB -0.00000000000000e+00	0.00000000e+00	NONE	0.00000000000000e+00	0.00000000000000e+00
662	C0000662	SB -0.00000000000000e+00	0.00000000e+00	NONE	0.00000000000000e+00	0.00000000000000e+00
663	C0000663	SB -0.00000000000000e+00	0.00000000e+00	NONE	0.00000000000000e+00	0.00000000000000e+00
664	C0000664	SB 0.00000000000000e+00	0.00000000e+00	NONE	0.00000000000000e+00	0.00000000000000e+00
665	C0000665	SB 0.00000000000000e+00	0.00000000e+00	NONE	0.00000000000000e+00	0.00000000000000e+00
666	C0000666	SB 0.00000000000000e+00	0.00000000e+00	NONE	0.00000000000000e+00	0.00000000000000e+00
667	C0000667	SB 0.00000000000000e+00	0.00000000e+00	NONE	0.00000000000000e+00	0.00000000000000e+00
668	C0000668	SB 1.66781884899220e+11	0.00000000e+00	NONE	0.00000000000000e+00	0.00000000000000e+00
669	C0000669	SB 1.44112424064288e-04	0.00000000e+00	NONE	0.00000000000000e+00	0.00000000000000e+00

670	C0000670	SB 7.70658076413308e-04	0.00000000e+00	NONE	0.00000000000000e+00	0.00000000000000e+00
671	C0000671	LL 0.00000000000000e+00	0.00000000e+00	NONE	0.00000000000000e+00	0.00000000000000e+00
672	C0000672	LL 1.69215983991203e-04	0.00000000e+00	NONE	3.78100698493820e+04	0.00000000000000e+00
673	C0000673	LL 0.00000000000000e+00	0.00000000e+00	NONE	7.42413579061579e+04	0.00000000000000e+00
674	C0000674	LL 0.00000000000000e+00	0.00000000e+00	NONE	3.78100698493817e+04	0.00000000000000e+00
675	C0000675	LL 0.00000000000000e+00	0.00000000e+00	NONE	3.78100698493817e+04	0.00000000000000e+00
676	C0000676	LL 0.00000000000000e+00	0.00000000e+00	NONE	0.00000000000000e+00	0.00000000000000e+00
677	C0000677	LL 0.00000000000000e+00	0.00000000e+00	NONE	1.37878179260560e+03	0.00000000000000e+00
678	C0000678	LL 0.00000000000000e+00	0.00000000e+00	NONE	1.37878179260560e+03	0.00000000000000e+00
679	C0000679	SB 0.00000000000000e+00	NONE	NONE	0.00000000000000e+00	0.00000000000000e+00
680	C0000680	SB 4.63564081912159e+11	NONE	NONE	0.00000000000000e+00	0.00000000000000e+00
681	C0000681	SB 2.18671931296504e+10	0.00000000e+00	NONE	0.00000000000000e+00	0.00000000000000e+00
682	C0000682	LL 0.00000000000000e+00	0.00000000e+00	NONE	0.00000000000000e+00	0.00000000000000e+00
683	C0000683	LL 0.00000000000000e+00	0.00000000e+00	NONE	0.00000000000000e+00	0.00000000000000e+00
684	C0000684	LL 0.00000000000000e+00	0.00000000e+00	NONE	0.00000000000000e+00	0.00000000000000e+00
685	C0000685	LL 0.00000000000000e+00	0.00000000e+00	NONE	0.00000000000000e+00	0.00000000000000e+00
686	C0000686	LL 0.00000000000000e+00	0.00000000e+00	NONE	0.00000000000000e+00	0.00000000000000e+00
687	C0000687	LL 0.00000000000000e+00	0.00000000e+00	NONE	0.00000000000000e+00	0.00000000000000e+00
688	C0000688	LL 0.00000000000000e+00	0.00000000e+00	NONE	0.00000000000000e+00	0.00000000000000e+00
689	C0000689	SB 0.00000000000000e+00	0.00000000e+00	NONE	0.00000000000000e+00	0.00000000000000e+00
690	C0000690	SB 2.05441826536973e+10	0.00000000e+00	NONE	0.00000000000000e+00	0.00000000000000e+00
691	C0000691	SB 0.00000000000000e+00	0.00000000e+00	NONE	0.00000000000000e+00	0.00000000000000e+00
692	C0000692	SB 0.00000000000000e+00	0.00000000e+00	NONE	0.00000000000000e+00	0.00000000000000e+00
693	C0000693	SB 2.33998909482262e+10	0.00000000e+00	NONE	1.59204806935896e-10	0.00000000000000e+00
694	C0000694	LL 9.14641155588388e-04	0.00000000e+00	NONE	6.48881073228616e+04	0.00000000000000e+00
695	C0000695	LL 0.00000000000000e+00	0.00000000e+00	NONE	6.48881073228616e+04	0.00000000000000e+00
696	C0000696	SB 0.00000000000000e+00	0.00000000e+00	NONE	0.00000000000000e+00	0.00000000000000e+00
697	C0000697	SB 0.00000000000000e+00	0.00000000e+00	NONE	0.00000000000000e+00	0.00000000000000e+00
698	C0000698	LL 0.00000000000000e+00	0.00000000e+00	NONE	0.00000000000000e+00	0.00000000000000e+00
699	C0000699	LL 0.00000000000000e+00	0.00000000e+00	NONE	0.00000000000000e+00	0.00000000000000e+00
700	C0000700	LL 0.00000000000000e+00	0.00000000e+00	NONE	0.00000000000000e+00	0.00000000000000e+00
701	C0000701	LL 0.00000000000000e+00	0.00000000e+00	NONE	0.00000000000000e+00	0.00000000000000e+00
702	C0000702	SB -0.00000000000000e+00	NONE	NONE	0.00000000000000e+00	0.00000000000000e+00
703	C0000703	SB -0.00000000000000e+00	NONE	NONE	0.00000000000000e+00	0.00000000000000e+00
704	C0000704	SB 0.00000000000000e+00	0.00000000e+00	NONE	0.00000000000000e+00	0.00000000000000e+00
705	C0000705	SB 0.00000000000000e+00	0.00000000e+00	NONE	0.00000000000000e+00	0.00000000000000e+00
706	C0000706	SB 2.05441826536973e+10	0.00000000e+00	NONE	0.00000000000000e+00	0.00000000000000e+00
707	C0000707	SB 2.33998909482262e+10	0.00000000e+00	NONE	0.00000000000000e+00	0.00000000000000e+00
708	C0000708	SB 0.00000000000000e+00	0.00000000e+00	NONE	0.00000000000000e+00	0.00000000000000e+00
709	C0000709	LL 2.81485806428314e-12	0.00000000e+00	NONE	2.14748831854473e+10	0.00000000000000e+00
710	C0000710	LL 0.00000000000000e+00	0.00000000e+00	NONE	0.00000000000000e+00	0.00000000000000e+00
711	C0000711	SB -0.00000000000000e+00	NONE	NONE	0.00000000000000e+00	0.00000000000000e+00
712	C0000712	SB -2.18671931296504e+10	NONE	NONE	0.00000000000000e+00	0.00000000000000e+00
713	C0000713	SB 0.00000000000000e+00	NONE	NONE	0.00000000000000e+00	0.00000000000000e+00
714	C0000714	SB 8.06662658256757e+09	NONE	NONE	0.00000000000000e+00	0.00000000000000e+00
715	C0000715	SB 8.06662658256757e+09	NONE	NONE	0.00000000000000e+00	0.00000000000000e+00
716	C0000716	SB 8.06662658256757e+09	0.00000000e+00	NONE	0.00000000000000e+00	0.00000000000000e+00
717	C0000717	LL 0.00000000000000e+00	0.00000000e+00	NONE	0.00000000000000e+00	0.00000000000000e+00
718	C0000718	LL 0.00000000000000e+00	0.00000000e+00	NONE	0.00000000000000e+00	0.00000000000000e+00
719	C0000719	SB 8.06662658256757e+09	0.00000000e+00	NONE	1.87442143606701e-10	0.00000000000000e+00
720	C0000720	SB 0.00000000000000e+00	0.00000000e+00	NONE	0.00000000000000e+00	0.00000000000000e+00
721	C0000721	SB -8.06662658256757e+09	NONE	NONE	0.00000000000000e+00	0.00000000000000e+00
722	C0000722	SB 8.06662658256757e+09	NONE	NONE	0.00000000000000e+00	0.00000000000000e+00
723	C0000723	SB 0.00000000000000e+00	0.00000000e+00	NONE	0.00000000000000e+00	0.00000000000000e+00
724	C0000724	LL 0.00000000000000e+00	0.00000000e+00	NONE	0.00000000000000e+00	0.00000000000000e+00
725	C0000725	SB 0.00000000000000e+00	0.00000000e+00	NONE	0.00000000000000e+00	0.00000000000000e+00
726	C0000726	SB 0.00000000000000e+00	NONE	NONE	0.00000000000000e+00	0.00000000000000e+00
727	C0000727	SB 0.00000000000000e+00	0.00000000e+00	NONE	0.00000000000000e+00	0.00000000000000e+00
728	C0000728	SB 2.18671931296504e+10	NONE	NONE	0.00000000000000e+00	0.00000000000000e+00
729	C0000729	SB -1.09335965648252e+11	NONE	NONE	0.00000000000000e+00	0.00000000000000e+00
730	C0000730	SB 0.00000000000000e+00	NONE	NONE	0.00000000000000e+00	0.00000000000000e+00
731	C0000731	SB 0.00000000000000e+00	NONE	NONE	0.00000000000000e+00	0.00000000000000e+00
732	C0000732	SB 0.00000000000000e+00	NONE	NONE	0.00000000000000e+00	0.00000000000000e+00
733	C0000733	SB -1.35615394561704e-01	NONE	NONE	0.00000000000000e+00	0.00000000000000e+00
734	C0000734	SB 0.00000000000000e+00	NONE	NONE	0.00000000000000e+00	0.00000000000000e+00
735	C0000735	LL 0.00000000000000e+00	0.00000000e+00	NONE	0.00000000000000e+00	0.00000000000000e+00
736	C0000736	LL 0.00000000000000e+00	0.00000000e+00	NONE	0.00000000000000e+00	0.00000000000000e+00
737	C0000737	SB 0.00000000000000e+00	NONE	NONE	0.00000000000000e+00	0.00000000000000e+00
738	C0000738	LL 0.00000000000000e+00	0.00000000e+00	NONE	0.00000000000000e+00	0.00000000000000e+00
739	C0000739	LL 0.00000000000000e+00	0.00000000e+00	NONE	0.00000000000000e+00	0.00000000000000e+00
740	C0000740	SB 0.00000000000000e+00	0.00000000e+00	NONE	0.00000000000000e+00	0.00000000000000e+00
741	C0000741	LL 0.00000000000000e+00	0.00000000e+00	NONE	0.00000000000000e+00	0.00000000000000e+00
742	C0000742	LL 0.00000000000000e+00	0.00000000e+00	NONE	0.00000000000000e+00	0.00000000000000e+00
743	C0000743	LL 0.00000000000000e+00	0.00000000e+00	NONE	0.00000000000000e+00	0.00000000000000e+00
744	C0000744	LL 0.00000000000000e+00	0.00000000e+00	NONE	0.00000000000000e+00	0.00000000000000e+00
745	C0000745	SB 0.00000000000000e+00	NONE	NONE	0.00000000000000e+00	0.00000000000000e+00
746	C0000746	SB 1.12646180464762e-01	0.00000000e+00	NONE	0.00000000000000e+00	0.00000000000000e+00
747	C0000747	LL 0.00000000000000e+00	0.00000000e+00	NONE	0.00000000000000e+00	0.00000000000000e+00
748	C0000748	LL 0.00000000000000e+00	0.00000000e+00	NONE	0.00000000000000e+00	0.00000000000000e+00
749	C0000749	SB 0.00000000000000e+00	NONE	NONE	0.00000000000000e+00	0.00000000000000e+00
750	C0000750	LL 0.00000000000000e+00	0.00000000e+00	NONE	0.00000000000000e+00	0.00000000000000e+00
751	C0000751	SB -1.31203158777902e+11	NONE	NONE	0.00000000000000e+00	0.00000000000000e+00
752	C0000752	SB 0.00000000000000e+00	NONE	NONE	0.00000000000000e+00	0.00000000000000e+00
753	C0000753	LL 0.00000000000000e+00	0.00000000e+00	NONE	0.00000000000000e+00	0.00000000000000e+00
754	C0000754	SB 0.00000000000000e+00	NONE	NONE	0.00000000000000e+00	0.00000000000000e+00
755	C0000755	LL 0.00000000000000e+00	0.00000000e+00	NONE	0.00000000000000e+00	0.00000000000000e+00
756	C0000756	LL 0.00000000000000e+00	0.00000000e+00	NONE	0.00000000000000e+00	0.00000000000000e+00
757	C0000757	LL 0.00000000000000e+00	0.00000000e+00	NONE	0.00000000000000e+00	0.00000000000000e+00
758	C0000758	LL 0.00000000000000e+00	0.00000000e+00	NONE	0.00000000000000e+00	0.00000000000000e+00
759	C0000759	LL 0.00000000000000e+00	0.00000000e+00	NONE	0.00000000000000e+00	0.00000000000000e+00
760	C0000760	LL 0.00000000000000e+00	0.00000000e+00	NONE	0.00000000000000e+00	0.00000000000000e+00
761	C0000761	LL 0.00000000000000e+00	0.00000000e+00	NONE	0.00000000000000e+00	0.00000000000000e+00
762	C0000762	LL 0.00000000000000e+00	0.00000000e+00	NONE	0.00000000000000e+00	0.00000000000000e+00
763	C0000763	LL 0.00000000000000e+00	0.00000000e+00	NONE	0.00000000000000e+00	0.00000000000000e+00
764	C0000764	LL 0.00000000000000e+00	0.00000000e+00	NONE	0.00000000000000e+00	0.00000000000000e+00
765	C0000765	LL 0.00000000000000e+00	0.00000000e+00	NONE	0.00000000000000e+00	0.00000000000000e+00
766	C0000766	LL 0.00000000000000e+00	0.00000000e+00	NONE	0.00000000000000e+00	0.00000000000000e+00
767	C0000767	LL 0.00000000000000e+00	0.00000000e+00	NONE	0.00000000000000e+00	0.00000000000000e+00

768	C0000768	LL 0.00000000000000e+00	0.00000000e+00	NONE	0.00000000000000e+00	0.00000000000000e+00
769	C0000769	SB 0.00000000000000e+00	0.00000000e+00	NONE	0.00000000000000e+00	0.00000000000000e+00
770	C0000770	SB 0.00000000000000e+00	NONE	NONE	0.00000000000000e+00	0.00000000000000e+00
771	C0000771	SB 0.00000000000000e+00	NONE	NONE	0.00000000000000e+00	0.00000000000000e+00
772	C0000772	SB 0.00000000000000e+00	0.00000000e+00	NONE	0.00000000000000e+00	0.00000000000000e+00
773	C0000773	SB -0.00000000000000e+00	0.00000000e+00	NONE	0.00000000000000e+00	0.00000000000000e+00
774	C0000774	LL 0.00000000000000e+00	0.00000000e+00	NONE	0.00000000000000e+00	0.00000000000000e+00
775	C0000775	LL 0.00000000000000e+00	0.00000000e+00	NONE	0.00000000000000e+00	0.00000000000000e+00
776	C0000776	SB 0.00000000000000e+00	0.00000000e+00	NONE	0.00000000000000e+00	0.00000000000000e+00
777	C0000777	SB -0.00000000000000e+00	0.00000000e+00	NONE	0.00000000000000e+00	0.00000000000000e+00
778	C0000778	LL 0.00000000000000e+00	0.00000000e+00	NONE	0.00000000000000e+00	0.00000000000000e+00
779	C0000779	LL 0.00000000000000e+00	0.00000000e+00	NONE	0.00000000000000e+00	0.00000000000000e+00
780	C0000780	LL 0.00000000000000e+00	0.00000000e+00	NONE	0.00000000000000e+00	0.00000000000000e+00
781	C0000781	SB 0.00000000000000e+00	NONE	NONE	0.00000000000000e+00	0.00000000000000e+00
782	C0000782	LL 0.00000000000000e+00	0.00000000e+00	NONE	0.00000000000000e+00	0.00000000000000e+00
783	C0000783	LL 0.00000000000000e+00	0.00000000e+00	NONE	0.00000000000000e+00	0.00000000000000e+00
784	C0000784	LL 0.00000000000000e+00	0.00000000e+00	NONE	0.00000000000000e+00	0.00000000000000e+00
785	C0000785	SB 0.00000000000000e+00	0.00000000e+00	NONE	0.00000000000000e+00	0.00000000000000e+00
786	C0000786	SB -0.00000000000000e+00	0.00000000e+00	NONE	0.00000000000000e+00	0.00000000000000e+00
787	C0000787	SB -0.00000000000000e+00	0.00000000e+00	NONE	0.00000000000000e+00	0.00000000000000e+00
788	C0000788	LL 1.36837218064384e-04	0.00000000e+00	NONE	1.80692412766383e+05	0.00000000000000e+00
789	C0000789	LL 0.00000000000000e+00	0.00000000e+00	NONE	0.00000000000000e+00	0.00000000000000e+00
790	C0000790	LL 0.00000000000000e+00	0.00000000e+00	NONE	0.00000000000000e+00	0.00000000000000e+00
791	C0000791	SB 1.39269785360470e+11	NONE	NONE	0.00000000000000e+00	0.00000000000000e+00
792	C0000792	SB 2.03645884315475e+09	NONE	NONE	0.00000000000000e+00	0.00000000000000e+00
793	C0000793	SB 2.03645884315475e+09	NONE	NONE	0.00000000000000e+00	0.00000000000000e+00
794	C0000794	SB 2.03645884315474e+09	NONE	NONE	0.00000000000000e+00	0.00000000000000e+00
795	C0000795	SB 2.03645884315475e+09	NONE	NONE	0.00000000000000e+00	0.00000000000000e+00
796	C0000796	SB 2.10700166620784e+09	NONE	NONE	0.00000000000000e+00	0.00000000000000e+00
797	C0000797	SB 2.13362476705130e+09	NONE	NONE	0.00000000000000e+00	0.00000000000000e+00
798	C0000798	SB 6.64376745765031e+09	NONE	NONE	0.00000000000000e+00	0.00000000000000e+00
799	C0000799	SB 2.83696386612192e+09	NONE	NONE	0.00000000000000e+00	0.00000000000000e+00
800	C0000800	SB 0.00000000000000e+00	NONE	NONE	0.00000000000000e+00	0.00000000000000e+00
801	C0000801	SB -0.00000000000000e+00	NONE	NONE	0.00000000000000e+00	0.00000000000000e+00
802	C0000802	SB 5.61335006884581e+09	0.00000000e+00	NONE	1.93015273590069e-10	0.00000000000000e+00
803	C0000803	SB 4.68963912207602e+09	0.00000000e+00	NONE	1.96676558240179e-10	0.00000000000000e+00
804	C0000804	SB 5.46465278406552e+09	0.00000000e+00	NONE	2.56427552712569e-10	0.00000000000000e+00
805	C0000805	LL 0.00000000000000e+00	0.00000000e+00	NONE	6.30810074355463e+04	0.00000000000000e+00
806	C0000806	SB -0.00000000000000e+00	0.00000000e+00	NONE	0.00000000000000e+00	0.00000000000000e+00
807	C0000807	SB 0.00000000000000e+00	NONE	NONE	0.00000000000000e+00	0.00000000000000e+00
808	C0000808	LL 0.00000000000000e+00	0.00000000e+00	NONE	0.00000000000000e+00	0.00000000000000e+00
809	C0000809	SB 0.00000000000000e+00	0.00000000e+00	NONE	0.00000000000000e+00	0.00000000000000e+00
810	C0000810	SB 0.00000000000000e+00	0.00000000e+00	NONE	0.00000000000000e+00	0.00000000000000e+00
811	C0000811	LL 0.00000000000000e+00	0.00000000e+00	NONE	0.00000000000000e+00	0.00000000000000e+00
812	C0000812	SB -0.00000000000000e+00	NONE	NONE	0.00000000000000e+00	0.00000000000000e+00
813	C0000813	SB 0.00000000000000e+00	NONE	NONE	0.00000000000000e+00	0.00000000000000e+00
814	C0000814	SB 6.63359725366489e-03	0.00000000e+00	NONE	0.00000000000000e+00	0.00000000000000e+00
815	C0000815	SB 2.33998909482262e+10	0.00000000e+00	NONE	0.00000000000000e+00	0.00000000000000e+00
816	C0000816	LL 0.00000000000000e+00	0.00000000e+00	NONE	0.00000000000000e+00	0.00000000000000e+00
817	C0000817	LL 0.00000000000000e+00	0.00000000e+00	NONE	0.00000000000000e+00	0.00000000000000e+00
818	C0000818	LL 0.00000000000000e+00	0.00000000e+00	NONE	0.00000000000000e+00	0.00000000000000e+00
819	C0000819	SB 0.00000000000000e+00	0.00000000e+00	NONE	0.00000000000000e+00	0.00000000000000e+00
820	C0000820	SB -0.00000000000000e+00	0.00000000e+00	NONE	0.00000000000000e+00	0.00000000000000e+00
821	C0000821	LL 0.00000000000000e+00	0.00000000e+00	NONE	0.00000000000000e+00	0.00000000000000e+00
822	C0000822	LL 0.00000000000000e+00	0.00000000e+00	NONE	0.00000000000000e+00	0.00000000000000e+00
823	C0000823	LL 0.00000000000000e+00	0.00000000e+00	NONE	0.00000000000000e+00	0.00000000000000e+00
824	C0000824	LL 0.00000000000000e+00	0.00000000e+00	NONE	0.00000000000000e+00	0.00000000000000e+00
825	C0000825	LL 0.00000000000000e+00	0.00000000e+00	NONE	0.00000000000000e+00	0.00000000000000e+00
826	C0000826	SB -0.00000000000000e+00	NONE	NONE	0.00000000000000e+00	0.00000000000000e+00
827	C0000827	SB 0.00000000000000e+00	NONE	NONE	0.00000000000000e+00	0.00000000000000e+00
828	C0000828	SB 0.00000000000000e+00	NONE	NONE	0.00000000000000e+00	0.00000000000000e+00
829	C0000829	LL 0.00000000000000e+00	0.00000000e+00	NONE	0.00000000000000e+00	0.00000000000000e+00
830	C0000830	SB 0.00000000000000e+00	0.00000000e+00	NONE	0.00000000000000e+00	0.00000000000000e+00
831	C0000831	LL 0.00000000000000e+00	0.00000000e+00	NONE	0.00000000000000e+00	0.00000000000000e+00
832	C0000832	LL 0.00000000000000e+00	0.00000000e+00	NONE	0.00000000000000e+00	0.00000000000000e+00
833	C0000833	LL 0.00000000000000e+00	0.00000000e+00	NONE	0.00000000000000e+00	0.00000000000000e+00
834	C0000834	LL 0.00000000000000e+00	0.00000000e+00	NONE	0.00000000000000e+00	0.00000000000000e+00
835	C0000835	LL 0.00000000000000e+00	0.00000000e+00	NONE	0.00000000000000e+00	0.00000000000000e+00
836	C0000836	LL 0.00000000000000e+00	0.00000000e+00	NONE	0.00000000000000e+00	0.00000000000000e+00
837	C0000837	SB -3.81054928239810e+00	NONE	NONE	0.00000000000000e+00	0.00000000000000e+00
838	C0000838	SB 0.00000000000000e+00	NONE	NONE	0.00000000000000e+00	0.00000000000000e+00
839	C0000839	SB -0.00000000000000e+00	NONE	NONE	0.00000000000000e+00	0.00000000000000e+00
840	C0000840	SB 2.36415692618392e+00	NONE	NONE	0.00000000000000e+00	0.00000000000000e+00
841	C0000841	SB -2.46701848088552e-01	NONE	NONE	0.00000000000000e+00	0.00000000000000e+00
842	C0000842	SB -3.80514872176114e+00	NONE	NONE	0.00000000000000e+00	0.00000000000000e+00
843	C0000843	SB 0.00000000000000e+00	NONE	NONE	0.00000000000000e+00	0.00000000000000e+00
844	C0000844	SB 0.00000000000000e+00	NONE	NONE	0.00000000000000e+00	0.00000000000000e+00
845	C0000845	SB -0.00000000000000e+00	NONE	NONE	0.00000000000000e+00	0.00000000000000e+00
846	C0000846	SB 0.00000000000000e+00	0.00000000e+00	NONE	0.00000000000000e+00	0.00000000000000e+00
847	C0000847	SB -0.00000000000000e+00	0.00000000e+00	NONE	0.00000000000000e+00	0.00000000000000e+00
848	C0000848	SB 0.00000000000000e+00	0.00000000e+00	NONE	0.00000000000000e+00	0.00000000000000e+00
849	C0000849	SB 1.72305317596191e-01	NONE	NONE	0.00000000000000e+00	0.00000000000000e+00
850	C0000850	SB 0.00000000000000e+00	NONE	NONE	0.00000000000000e+00	0.00000000000000e+00
851	C0000851	SB 0.00000000000000e+00	NONE	NONE	0.00000000000000e+00	0.00000000000000e+00
852	C0000852	LL 0.00000000000000e+00	0.00000000e+00	NONE	0.00000000000000e+00	0.00000000000000e+00
853	C0000853	SB 5.40056063695085e-03	0.00000000e+00	NONE	0.00000000000000e+00	0.00000000000000e+00
854	C0000854	SB 0.00000000000000e+00	0.00000000e+00	NONE	0.00000000000000e+00	0.00000000000000e+00
855	C0000855	SB -0.00000000000000e+00	0.00000000e+00	NONE	0.00000000000000e+00	0.00000000000000e+00
856	C0000856	SB 0.00000000000000e+00	0.00000000e+00	NONE	0.00000000000000e+00	0.00000000000000e+00
857	C0000857	SB 0.00000000000000e+00	0.00000000e+00	NONE	0.00000000000000e+00	0.00000000000000e+00
858	C0000858	LL 0.00000000000000e+00	0.00000000e+00	NONE	0.00000000000000e+00	0.00000000000000e+00
859	C0000859	LL 0.00000000000000e+00	0.00000000e+00	NONE	0.00000000000000e+00	0.00000000000000e+00
860	C0000860	SB -2.13351446211510e+00	NONE	NONE	0.00000000000000e+00	0.00000000000000e+00
861	C0000861	LL 0.00000000000000e+00	0.00000000e+00	NONE	0.00000000000000e+00	0.00000000000000e+00
862	C0000862	LL 0.00000000000000e+00	0.00000000e+00	NONE	0.00000000000000e+00	0.00000000000000e+00
863	C0000863	SB 0.00000000000000e+00	0.00000000e+00	NONE	0.00000000000000e+00	0.00000000000000e+00
864	C0000864	SB 0.00000000000000e+00	0.00000000e+00	NONE	0.00000000000000e+00	0.00000000000000e+00
865	C0000865	SB 0.00000000000000e+00	0.00000000e+00	NONE	0.00000000000000e+00	0.00000000000000e+00

866	C0000866	SB 0.00000000000000e+00	0.00000000e+00	NONE	0.00000000000000e+00	0.00000000000000e+00
867	C0000867	SB 2.13259966222735e+00	0.00000000e+00	NONE	0.00000000000000e+00	0.00000000000000e+00
868	C0000868	SB 0.00000000000000e+00	0.00000000e+00	NONE	0.00000000000000e+00	0.00000000000000e+00
869	C0000869	SB 0.00000000000000e+00	NONE	NONE	0.00000000000000e+00	0.00000000000000e+00
870	C0000870	SB 0.00000000000000e+00	NONE	NONE	0.00000000000000e+00	0.00000000000000e+00
871	C0000871	SB -6.39058046949904e-03	NONE	NONE	0.00000000000000e+00	0.00000000000000e+00
872	C0000872	SB -0.00000000000000e+00	NONE	NONE	0.00000000000000e+00	0.00000000000000e+00
873	C0000873	SB 0.00000000000000e+00	NONE	NONE	0.00000000000000e+00	0.00000000000000e+00
874	C0000874	SB -0.00000000000000e+00	NONE	NONE	0.00000000000000e+00	0.00000000000000e+00
875	C0000875	SB 6.39058046949904e-03	NONE	NONE	0.00000000000000e+00	0.00000000000000e+00
876	C0000876	SB -0.00000000000000e+00	NONE	NONE	0.00000000000000e+00	0.00000000000000e+00
877	C0000877	SB -0.00000000000000e+00	NONE	NONE	0.00000000000000e+00	0.00000000000000e+00
878	C0000878	SB 0.00000000000000e+00	NONE	NONE	0.00000000000000e+00	0.00000000000000e+00
879	C0000879	SB 0.00000000000000e+00	NONE	NONE	0.00000000000000e+00	0.00000000000000e+00
880	C0000880	SB 1.11086453283902e-01	NONE	NONE	0.00000000000000e+00	0.00000000000000e+00
881	C0000881	SB 0.00000000000000e+00	NONE	NONE	0.00000000000000e+00	0.00000000000000e+00
882	C0000882	LL 1.79096324915912e-04	0.00000000e+00	NONE	2.70387962291657e+05	0.00000000000000e+00
883	C0000883	SB 3.86577429323317e-02	0.00000000e+00	NONE	0.00000000000000e+00	0.00000000000000e+00
884	C0000884	SB 0.00000000000000e+00	0.00000000e+00	NONE	0.00000000000000e+00	0.00000000000000e+00
885	C0000885	SB 0.00000000000000e+00	0.00000000e+00	NONE	0.00000000000000e+00	0.00000000000000e+00
886	C0000886	SB -0.00000000000000e+00	0.00000000e+00	NONE	0.00000000000000e+00	0.00000000000000e+00
887	C0000887	SB -0.00000000000000e+00	0.00000000e+00	NONE	0.00000000000000e+00	0.00000000000000e+00
888	C0000888	SB 2.47365827858315e+09	0.00000000e+00	NONE	0.00000000000000e+00	0.00000000000000e+00
889	C0000889	SB -0.00000000000000e+00	0.00000000e+00	NONE	0.00000000000000e+00	0.00000000000000e+00
890	C0000890	SB 0.00000000000000e+00	0.00000000e+00	NONE	0.00000000000000e+00	0.00000000000000e+00
891	C0000891	SB 0.00000000000000e+00	0.00000000e+00	NONE	0.00000000000000e+00	0.00000000000000e+00
892	C0000892	SB 0.00000000000000e+00	0.00000000e+00	NONE	0.00000000000000e+00	0.00000000000000e+00
893	C0000893	SB -0.00000000000000e+00	0.00000000e+00	NONE	0.00000000000000e+00	0.00000000000000e+00
894	C0000894	SB 0.00000000000000e+00	0.00000000e+00	NONE	0.00000000000000e+00	0.00000000000000e+00
895	C0000895	SB 7.81357630476114e+09	0.00000000e+00	NONE	1.60276505418241e-10	0.00000000000000e+00
896	C0000896	SB 7.23654754949763e+09	0.00000000e+00	NONE	1.82516582383918e-10	0.00000000000000e+00
897	C0000897	LL 0.00000000000000e+00	0.00000000e+00	NONE	2.14748706463151e+10	0.00000000000000e+00
898	C0000898	SB 0.00000000000000e+00	0.00000000e+00	NONE	0.00000000000000e+00	0.00000000000000e+00
899	C0000899	SB 0.00000000000000e+00	0.00000000e+00	NONE	0.00000000000000e+00	0.00000000000000e+00
900	C0000900	SB 0.00000000000000e+00	0.00000000e+00	NONE	0.00000000000000e+00	0.00000000000000e+00
901	C0000901	SB 0.00000000000000e+00	0.00000000e+00	NONE	0.00000000000000e+00	0.00000000000000e+00
902	C0000902	SB 0.00000000000000e+00	0.00000000e+00	NONE	0.00000000000000e+00	0.00000000000000e+00
903	C0000903	SB 0.00000000000000e+00	0.00000000e+00	NONE	0.00000000000000e+00	0.00000000000000e+00
904	C0000904	SB 0.00000000000000e+00	0.00000000e+00	NONE	0.00000000000000e+00	0.00000000000000e+00
905	C0000905	SB 7.82846095735579e+09	0.00000000e+00	NONE	1.91991691136883e-10	0.00000000000000e+00
906	C0000906	SB -0.00000000000000e+00	0.00000000e+00	NONE	0.00000000000000e+00	0.00000000000000e+00
907	C0000907	SB 0.00000000000000e+00	0.00000000e+00	NONE	0.00000000000000e+00	0.00000000000000e+00
908	C0000908	SB 0.00000000000000e+00	0.00000000e+00	NONE	0.00000000000000e+00	0.00000000000000e+00
909	C0000909	SB -0.00000000000000e+00	0.00000000e+00	NONE	0.00000000000000e+00	0.00000000000000e+00
910	C0000910	SB -0.00000000000000e+00	0.00000000e+00	NONE	0.00000000000000e+00	0.00000000000000e+00
911	C0000911	LL 0.00000000000000e+00	0.00000000e+00	NONE	0.00000000000000e+00	0.00000000000000e+00
912	C0000912	SB -0.00000000000000e+00	0.00000000e+00	NONE	0.00000000000000e+00	0.00000000000000e+00
913	C0000913	SB 0.00000000000000e+00	NONE	NONE	0.00000000000000e+00	0.00000000000000e+00
914	C0000914	SB 0.00000000000000e+00	NONE	NONE	0.00000000000000e+00	0.00000000000000e+00
915	C0000915	SB 0.00000000000000e+00	NONE	NONE	0.00000000000000e+00	0.00000000000000e+00
916	C0000916	SB 2.42945548262619e-10	NONE	NONE	0.00000000000000e+00	0.00000000000000e+00
917	C0000917	SB 0.00000000000000e+00	NONE	NONE	0.00000000000000e+00	0.00000000000000e+00
918	C0000918	LL 2.13351446211510e+00	0.00000000e+00	NONE	3.79644002052449e+00	0.00000000000000e+00
919	C0000919	LL 0.00000000000000e+00	0.00000000e+00	NONE	0.00000000000000e+00	0.00000000000000e+00
920	C0000920	SB 1.52001762370274e-03	0.00000000e+00	NONE	0.00000000000000e+00	0.00000000000000e+00
921	C0000921	LL 0.00000000000000e+00	0.00000000e+00	NONE	0.00000000000000e+00	0.00000000000000e+00
922	C0000922	LL 0.00000000000000e+00	0.00000000e+00	NONE	0.00000000000000e+00	0.00000000000000e+00
923	C0000923	SB 0.00000000000000e+00	0.00000000e+00	NONE	0.00000000000000e+00	0.00000000000000e+00
924	C0000924	SB 0.00000000000000e+00	0.00000000e+00	NONE	0.00000000000000e+00	0.00000000000000e+00
925	C0000925	SB 0.00000000000000e+00	NONE	NONE	0.00000000000000e+00	0.00000000000000e+00
926	C0000926	SB 0.00000000000000e+00	NONE	NONE	0.00000000000000e+00	0.00000000000000e+00
927	C0000927	SB 0.00000000000000e+00	NONE	NONE	0.00000000000000e+00	0.00000000000000e+00
928	C0000928	SB 5.60022549878076e+10	0.00000000e+00	NONE	0.00000000000000e+00	0.00000000000000e+00
929	C0000929	SB 5.60022549878076e+10	0.00000000e+00	NONE	0.00000000000000e+00	0.00000000000000e+00
930	C0000930	SB 0.00000000000000e+00	NONE	NONE	0.00000000000000e+00	0.00000000000000e+00
931	C0000931	SB 0.00000000000000e+00	NONE	NONE	0.00000000000000e+00	0.00000000000000e+00
932	C0000932	SB 0.00000000000000e+00	NONE	NONE	0.00000000000000e+00	0.00000000000000e+00
933	C0000933	SB 3.04759790041274e+11	0.00000000e+00	NONE	0.00000000000000e+00	0.00000000000000e+00
934	C0000934	LL 0.00000000000000e+00	0.00000000e+00	NONE	0.00000000000000e+00	0.00000000000000e+00
935	C0000935	LL 0.00000000000000e+00	0.00000000e+00	NONE	0.00000000000000e+00	0.00000000000000e+00
936	C0000936	LL 0.00000000000000e+00	0.00000000e+00	NONE	0.00000000000000e+00	0.00000000000000e+00
937	C0000937	SB 2.28785848116146e+10	NONE	NONE	0.00000000000000e+00	0.00000000000000e+00
938	C0000938	SB 2.42945548262619e-10	0.00000000e+00	NONE	0.00000000000000e+00	0.00000000000000e+00
939	C0000939	SB -0.00000000000000e+00	0.00000000e+00	NONE	0.00000000000000e+00	0.00000000000000e+00
940	C0000940	SB 2.42945548262619e-10	0.00000000e+00	NONE	0.00000000000000e+00	0.00000000000000e+00
941	C0000941	SB 0.00000000000000e+00	0.00000000e+00	NONE	0.00000000000000e+00	0.00000000000000e+00
942	C0000942	SB 0.00000000000000e+00	NONE	NONE	0.00000000000000e+00	0.00000000000000e+00
943	C0000943	LL 0.00000000000000e+00	0.00000000e+00	NONE	0.00000000000000e+00	0.00000000000000e+00
944	C0000944	SB 9.61183072137312e+00	0.00000000e+00	NONE	0.00000000000000e+00	0.00000000000000e+00
945	C0000945	LL 0.00000000000000e+00	0.00000000e+00	NONE	0.00000000000000e+00	0.00000000000000e+00
946	C0000946	LL 0.00000000000000e+00	0.00000000e+00	NONE	0.00000000000000e+00	0.00000000000000e+00
947	C0000947	SB -0.00000000000000e+00	0.00000000e+00	NONE	0.00000000000000e+00	0.00000000000000e+00
948	C0000948	SB 0.00000000000000e+00	0.00000000e+00	NONE	0.00000000000000e+00	0.00000000000000e+00
949	C0000949	LL 0.00000000000000e+00	0.00000000e+00	NONE	0.00000000000000e+00	0.00000000000000e+00
950	C0000950	SB -0.00000000000000e+00	0.00000000e+00	NONE	0.00000000000000e+00	0.00000000000000e+00
951	C0000951	SB -0.00000000000000e+00	0.00000000e+00	NONE	0.00000000000000e+00	0.00000000000000e+00
952	C0000952	LL 0.00000000000000e+00	0.00000000e+00	NONE	0.00000000000000e+00	0.00000000000000e+00
953	C0000953	SB -5.94406374451319e+00	NONE	NONE	0.00000000000000e+00	0.00000000000000e+00
954	C0000954	SB 0.00000000000000e+00	NONE	NONE	0.00000000000000e+00	0.00000000000000e+00
955	C0000955	SB 8.86510729266539e-03	0.00000000e+00	NONE	0.00000000000000e+00	0.00000000000000e+00
956	C0000956	SB 6.39058046949904e-03	0.00000000e+00	NONE	0.00000000000000e+00	0.00000000000000e+00
957	C0000957	LL 0.00000000000000e+00	0.00000000e+00	NONE	0.00000000000000e+00	0.00000000000000e+00
958	C0000958	LL 0.00000000000000e+00	0.00000000e+00	NONE	0.00000000000000e+00	0.00000000000000e+00
959	C0000959	SB 0.00000000000000e+00	0.00000000e+00	NONE	0.00000000000000e+00	0.00000000000000e+00
960	C0000960	SB 5.94406374451319e+00	NONE	NONE	0.00000000000000e+00	0.00000000000000e+00
961	C0000961	SB 0.00000000000000e+00	0.00000000e+00	NONE	0.00000000000000e+00	0.00000000000000e+00
962	C0000962	SB 0.00000000000000e+00	0.00000000e+00	NONE	0.00000000000000e+00	0.00000000000000e+00
963	C0000963	SB 0.00000000000000e+00	0.00000000e+00	NONE	0.00000000000000e+00	0.00000000000000e+00

964	C0000964	SB -0.00000000000000e+00	0.00000000e+00	NONE	0.00000000000000e+00	0.00000000000000e+00
965	C0000965	LL 0.00000000000000e+00	0.00000000e+00	NONE	0.00000000000000e+00	0.00000000000000e+00
966	C0000966	LL 0.00000000000000e+00	0.00000000e+00	NONE	0.00000000000000e+00	0.00000000000000e+00
967	C0000967	SB 0.00000000000000e+00	0.00000000e+00	NONE	0.00000000000000e+00	0.00000000000000e+00
968	C0000968	LL 0.00000000000000e+00	0.00000000e+00	NONE	0.00000000000000e+00	0.00000000000000e+00
969	C0000969	SB 1.43983079175080e-04	0.00000000e+00	NONE	0.00000000000000e+00	0.00000000000000e+00
970	C0000970	LL 0.00000000000000e+00	0.00000000e+00	NONE	5.13967144641609e+03	0.00000000000000e+00
971	C0000971	SB 0.00000000000000e+00	0.00000000e+00	NONE	0.00000000000000e+00	0.00000000000000e+00
972	C0000972	SB -2.28785848116146e+10	NONE	NONE	0.00000000000000e+00	0.00000000000000e+00
973	C0000973	SB -2.28785848116146e+10	NONE	NONE	0.00000000000000e+00	0.00000000000000e+00
974	C0000974	SB 2.13351446211510e+00	NONE	NONE	0.00000000000000e+00	0.00000000000000e+00
975	C0000975	SB 0.00000000000000e+00	0.00000000e+00	NONE	0.00000000000000e+00	0.00000000000000e+00
976	C0000976	LL 0.00000000000000e+00	0.00000000e+00	NONE	0.00000000000000e+00	0.00000000000000e+00
977	C0000977	SB -0.00000000000000e+00	NONE	NONE	0.00000000000000e+00	0.00000000000000e+00
978	C0000978	SB -0.00000000000000e+00	0.00000000e+00	NONE	0.00000000000000e+00	0.00000000000000e+00
979	C0000979	SB 0.00000000000000e+00	0.00000000e+00	NONE	0.00000000000000e+00	0.00000000000000e+00
980	C0000980	SB 0.00000000000000e+00	0.00000000e+00	NONE	0.00000000000000e+00	0.00000000000000e+00
981	C0000981	SB 0.00000000000000e+00	0.00000000e+00	NONE	0.00000000000000e+00	0.00000000000000e+00
982	C0000982	SB -0.00000000000000e+00	NONE	NONE	0.00000000000000e+00	0.00000000000000e+00
983	C0000983	SB 0.00000000000000e+00	NONE	NONE	0.00000000000000e+00	0.00000000000000e+00
984	C0000984	LL 0.00000000000000e+00	0.00000000e+00	NONE	0.00000000000000e+00	0.00000000000000e+00
985	C0000985	SB 0.00000000000000e+00	NONE	NONE	0.00000000000000e+00	0.00000000000000e+00
986	C0000986	SB 0.00000000000000e+00	NONE	NONE	0.00000000000000e+00	0.00000000000000e+00
987	C0000987	SB 1.68357142837536e-02	0.00000000e+00	NONE	0.00000000000000e+00	0.00000000000000e+00
988	C0000988	SB 0.00000000000000e+00	0.00000000e+00	NONE	0.00000000000000e+00	0.00000000000000e+00
989	C0000989	SB 0.00000000000000e+00	0.00000000e+00	NONE	0.00000000000000e+00	0.00000000000000e+00
990	C0000990	SB 0.00000000000000e+00	0.00000000e+00	NONE	0.00000000000000e+00	0.00000000000000e+00
991	C0000991	SB 0.00000000000000e+00	0.00000000e+00	NONE	0.00000000000000e+00	0.00000000000000e+00
992	C0000992	SB -0.00000000000000e+00	0.00000000e+00	NONE	0.00000000000000e+00	0.00000000000000e+00
993	C0000993	LL 0.00000000000000e+00	0.00000000e+00	NONE	0.00000000000000e+00	0.00000000000000e+00
994	C0000994	SB 0.00000000000000e+00	NONE	NONE	0.00000000000000e+00	0.00000000000000e+00
995	C0000995	LL 0.00000000000000e+00	0.00000000e+00	NONE	0.00000000000000e+00	0.00000000000000e+00
996	C0000996	SB 0.00000000000000e+00	0.00000000e+00	NONE	0.00000000000000e+00	0.00000000000000e+00
997	C0000997	SB -0.00000000000000e+00	0.00000000e+00	NONE	0.00000000000000e+00	0.00000000000000e+00
998	C0000998	LL 0.00000000000000e+00	0.00000000e+00	NONE	3.61384825532765e+05	0.00000000000000e+00
999	C0000999	LL 0.00000000000000e+00	0.00000000e+00	NONE	0.00000000000000e+00	0.00000000000000e+00
1000	C0001000	SB 7.82846095735579e+09	0.00000000e+00	NONE	0.00000000000000e+00	0.00000000000000e+00
1001	C0001001	SB 7.81357630476114e+09	0.00000000e+00	NONE	0.00000000000000e+00	0.00000000000000e+00
1002	C0001002	SB 7.23654754949763e+09	0.00000000e+00	NONE	0.00000000000000e+00	0.00000000000000e+00
1003	C0001003	LL 0.00000000000000e+00	0.00000000e+00	NONE	0.00000000000000e+00	0.00000000000000e+00
1004	C0001004	LL 0.00000000000000e+00	0.00000000e+00	NONE	0.00000000000000e+00	0.00000000000000e+00
1005	C0001005	LL 0.00000000000000e+00	0.00000000e+00	NONE	0.00000000000000e+00	0.00000000000000e+00
1006	C0001006	LL 0.00000000000000e+00	0.00000000e+00	NONE	0.00000000000000e+00	0.00000000000000e+00
1007	C0001007	LL 0.00000000000000e+00	0.00000000e+00	NONE	0.00000000000000e+00	0.00000000000000e+00
1008	C0001008	LL 0.00000000000000e+00	0.00000000e+00	NONE	0.00000000000000e+00	0.00000000000000e+00
1009	C0001009	SB -2.60684352750168e+10	NONE	NONE	0.00000000000000e+00	0.00000000000000e+00
1010	C0001010	SB 0.00000000000000e+00	NONE	NONE	0.00000000000000e+00	0.00000000000000e+00
1011	C0001011	LL 0.00000000000000e+00	0.00000000e+00	NONE	0.00000000000000e+00	0.00000000000000e+00
1012	C0001012	LL 0.00000000000000e+00	0.00000000e+00	NONE	0.00000000000000e+00	0.00000000000000e+00
1013	C0001013	SB 4.63905364633269e-02	0.00000000e+00	NONE	0.00000000000000e+00	0.00000000000000e+00
1014	C0001014	LL 0.00000000000000e+00	0.00000000e+00	NONE	0.00000000000000e+00	0.00000000000000e+00
1015	C0001015	SB 0.00000000000000e+00	0.00000000e+00	NONE	0.00000000000000e+00	0.00000000000000e+00
1016	C0001016	SB 0.00000000000000e+00	0.00000000e+00	NONE	0.00000000000000e+00	0.00000000000000e+00
1017	C0001017	SB 9.14641155588388e-04	0.00000000e+00	NONE	0.00000000000000e+00	0.00000000000000e+00
1018	C0001018	SB 3.56307110380258e+00	NONE	NONE	0.00000000000000e+00	0.00000000000000e+00
1019	C0001019	SB 0.00000000000000e+00	NONE	NONE	0.00000000000000e+00	0.00000000000000e+00
1020	C0001020	SB 0.00000000000000e+00	NONE	NONE	0.00000000000000e+00	0.00000000000000e+00
1021	C0001021	SB 0.00000000000000e+00	NONE	NONE	0.00000000000000e+00	0.00000000000000e+00
1022	C0001022	SB 0.00000000000000e+00	NONE	NONE	0.00000000000000e+00	0.00000000000000e+00
1023	C0001023	SB 0.00000000000000e+00	NONE	NONE	0.00000000000000e+00	0.00000000000000e+00
1024	C0001024	SB 0.00000000000000e+00	NONE	NONE	0.00000000000000e+00	0.00000000000000e+00
1025	C0001025	SB 0.00000000000000e+00	NONE	NONE	0.00000000000000e+00	0.00000000000000e+00
1026	C0001026	SB 0.00000000000000e+00	NONE	NONE	0.00000000000000e+00	0.00000000000000e+00
1027	C0001027	SB 0.00000000000000e+00	NONE	NONE	0.00000000000000e+00	0.00000000000000e+00
1028	C0001028	SB 0.00000000000000e+00	NONE	NONE	0.00000000000000e+00	0.00000000000000e+00
1029	C0001029	SB 0.00000000000000e+00	NONE	NONE	0.00000000000000e+00	0.00000000000000e+00
1030	C0001030	SB 2.36415692618392e+00	NONE	NONE	0.00000000000000e+00	0.00000000000000e+00
1031	C0001031	SB 0.00000000000000e+00	NONE	NONE	0.00000000000000e+00	0.00000000000000e+00
1032	C0001032	SB 0.00000000000000e+00	NONE	NONE	0.00000000000000e+00	0.00000000000000e+00
1033	C0001033	LL 2.43306577406541e-04	0.00000000e+00	NONE	2.27438221000446e+05	0.00000000000000e+00
1034	C0001034	LL 0.00000000000000e+00	0.00000000e+00	NONE	2.27438221000446e+05	0.00000000000000e+00
1035	C0001035	LL 0.00000000000000e+00	0.00000000e+00	NONE	2.27438221000446e+05	0.00000000000000e+00
1036	C0001036	LL 0.00000000000000e+00	0.00000000e+00	NONE	2.27438221000446e+05	0.00000000000000e+00
1037	C0001037	LL 0.00000000000000e+00	0.00000000e+00	NONE	2.27438221000446e+05	0.00000000000000e+00
1038	C0001038	LL 0.00000000000000e+00	0.00000000e+00	NONE	2.27438221000446e+05	0.00000000000000e+00
1039	C0001039	LL 0.00000000000000e+00	0.00000000e+00	NONE	0.00000000000000e+00	0.00000000000000e+00
1040	C0001040	SB -0.00000000000000e+00	0.00000000e+00	NONE	0.00000000000000e+00	0.00000000000000e+00
1041	C0001041	LL 0.00000000000000e+00	0.00000000e+00	NONE	0.00000000000000e+00	0.00000000000000e+00
1042	C0001042	LL 0.00000000000000e+00	0.00000000e+00	NONE	0.00000000000000e+00	0.00000000000000e+00
1043	C0001043	SB 0.00000000000000e+00	0.00000000e+00	NONE	0.00000000000000e+00	0.00000000000000e+00
1044	C0001044	SB -0.00000000000000e+00	0.00000000e+00	NONE	0.00000000000000e+00	0.00000000000000e+00
1045	C0001045	SB 0.00000000000000e+00	0.00000000e+00	NONE	0.00000000000000e+00	0.00000000000000e+00
1046	C0001046	LL 0.00000000000000e+00	0.00000000e+00	NONE	0.00000000000000e+00	0.00000000000000e+00
1047	C0001047	LL 0.00000000000000e+00	0.00000000e+00	NONE	0.00000000000000e+00	0.00000000000000e+00
1048	C0001048	LL 0.00000000000000e+00	0.00000000e+00	NONE	0.00000000000000e+00	0.00000000000000e+00
1049	C0001049	SB -0.00000000000000e+00	0.00000000e+00	NONE	0.00000000000000e+00	0.00000000000000e+00
1050	C0001050	LL 0.00000000000000e+00	0.00000000e+00	NONE	0.00000000000000e+00	0.00000000000000e+00
1051	C0001051	LL 0.00000000000000e+00	0.00000000e+00	NONE	0.00000000000000e+00	0.00000000000000e+00
1052	C0001052	SB 3.56307110380258e+00	NONE	NONE	0.00000000000000e+00	0.00000000000000e+00
1053	C0001053	SB 2.38099264071062e+00	NONE	NONE	0.00000000000000e+00	0.00000000000000e+00
1054	C0001054	SB 0.00000000000000e+00	NONE	NONE	0.00000000000000e+00	0.00000000000000e+00
1055	C0001055	SB 0.00000000000000e+00	NONE	NONE	0.00000000000000e+00	0.00000000000000e+00
1056	C0001056	LL 0.00000000000000e+00	0.00000000e+00	NONE	0.00000000000000e+00	0.00000000000000e+00
1057	C0001057	LL 0.00000000000000e+00	0.00000000e+00	NONE	0.00000000000000e+00	0.00000000000000e+00
1058	C0001058	LL 0.00000000000000e+00	0.00000000e+00	NONE	0.00000000000000e+00	0.00000000000000e+00
1059	C0001059	SB -1.68357145266991e-02	NONE	NONE	0.00000000000000e+00	0.00000000000000e+00
1060	C0001060	SB 0.00000000000000e+00	0.00000000e+00	NONE	0.00000000000000e+00	0.00000000000000e+00
1061	C0001061	LL 0.00000000000000e+00	0.00000000e+00	NONE	0.00000000000000e+00	0.00000000000000e+00

1062	C0001062	SB -0.00000000000000e+00	0.00000000e+00	NONE	0.00000000000000e+00	0.00000000000000e+00
1063	C0001063	LL 0.00000000000000e+00	0.00000000e+00	NONE	0.00000000000000e+00	0.00000000000000e+00
1064	C0001064	SB 0.00000000000000e+00	NONE	NONE	0.00000000000000e+00	0.00000000000000e+00
1065	C0001065	SB 0.00000000000000e+00	0.00000000e+00	NONE	0.00000000000000e+00	0.00000000000000e+00
1066	C0001066	LL 0.00000000000000e+00	0.00000000e+00	NONE	0.00000000000000e+00	0.00000000000000e+00
1067	C0001067	LL 0.00000000000000e+00	0.00000000e+00	NONE	0.00000000000000e+00	0.00000000000000e+00
1068	C0001068	LL 0.00000000000000e+00	0.00000000e+00	NONE	0.00000000000000e+00	0.00000000000000e+00
1069	C0001069	SB 0.00000000000000e+00	0.00000000e+00	NONE	0.00000000000000e+00	0.00000000000000e+00
1070	C0001070	LL 0.00000000000000e+00	0.00000000e+00	NONE	0.00000000000000e+00	0.00000000000000e+00
1071	C0001071	SB 0.00000000000000e+00	0.00000000e+00	NONE	0.00000000000000e+00	0.00000000000000e+00
1072	C0001072	SB -0.00000000000000e+00	0.00000000e+00	NONE	0.00000000000000e+00	0.00000000000000e+00
1073	C0001073	SB 1.52556877621644e-02	0.00000000e+00	NONE	0.00000000000000e+00	0.00000000000000e+00
1074	C0001074	LL 0.00000000000000e+00	0.00000000e+00	NONE	0.00000000000000e+00	0.00000000000000e+00
1075	C0001075	SB 9.14641155588388e-04	0.00000000e+00	NONE	0.00000000000000e+00	0.00000000000000e+00
1076	C0001076	SB 4.76288927091448e+09	0.00000000e+00	NONE	0.00000000000000e+00	0.00000000000000e+00
1077	C0001077	SB 1.34449733375158e+10	0.00000000e+00	NONE	0.00000000000000e+00	0.00000000000000e+00
1078	C0001078	SB 9.71466979059966e+09	0.00000000e+00	NONE	0.00000000000000e+00	0.00000000000000e+00
1079	C0001079	SB 2.05441826536973e+10	0.00000000e+00	NONE	0.00000000000000e+00	0.00000000000000e+00
1080	C0001080	SB 2.05441826536973e+10	0.00000000e+00	NONE	0.00000000000000e+00	0.00000000000000e+00
1081	C0001081	SB 2.05441826536973e+10	0.00000000e+00	NONE	0.00000000000000e+00	0.00000000000000e+00
1082	C0001082	SB 2.05441826536973e+10	0.00000000e+00	NONE	0.00000000000000e+00	0.00000000000000e+00
1083	C0001083	LL 0.00000000000000e+00	0.00000000e+00	NONE	0.00000000000000e+00	0.00000000000000e+00
1084	C0001084	SB 1.18207846309196e+00	0.00000000e+00	NONE	0.00000000000000e+00	0.00000000000000e+00
1085	C0001085	LL 0.00000000000000e+00	0.00000000e+00	NONE	0.00000000000000e+00	0.00000000000000e+00
1086	C0001086	LL 0.00000000000000e+00	0.00000000e+00	NONE	0.00000000000000e+00	0.00000000000000e+00
1087	C0001087	SB 2.42945548262619e-10	0.00000000e+00	NONE	0.00000000000000e+00	0.00000000000000e+00
1088	C0001088	SB 1.43983079175080e-04	0.00000000e+00	NONE	0.00000000000000e+00	0.00000000000000e+00
1089	C0001089	LL 0.00000000000000e+00	0.00000000e+00	NONE	0.00000000000000e+00	0.00000000000000e+00
1090	C0001090	SB 0.00000000000000e+00	NONE	NONE	0.00000000000000e+00	0.00000000000000e+00
1091	C0001091	SB 0.00000000000000e+00	NONE	NONE	0.00000000000000e+00	0.00000000000000e+00
1092	C0001092	SB 0.00000000000000e+00	0.00000000e+00	NONE	0.00000000000000e+00	0.00000000000000e+00
1093	C0001093	SB 0.00000000000000e+00	0.00000000e+00	NONE	0.00000000000000e+00	0.00000000000000e+00
1094	C0001094	SB -0.00000000000000e+00	0.00000000e+00	NONE	0.00000000000000e+00	0.00000000000000e+00
1095	C0001095	LL 0.00000000000000e+00	0.00000000e+00	NONE	0.00000000000000e+00	0.00000000000000e+00
1096	C0001096	SB 0.00000000000000e+00	NONE	NONE	0.00000000000000e+00	0.00000000000000e+00
1097	C0001097	SB -8.74687725186014e+10	NONE	NONE	0.00000000000000e+00	0.00000000000000e+00
1098	C0001098	SB 0.00000000000000e+00	NONE	NONE	0.00000000000000e+00	0.00000000000000e+00
1099	C0001099	SB 0.00000000000000e+00	NONE	NONE	0.00000000000000e+00	0.00000000000000e+00
1100	C0001100	SB 0.00000000000000e+00	NONE	NONE	0.00000000000000e+00	0.00000000000000e+00
1101	C0001101	SB -0.00000000000000e+00	NONE	NONE	0.00000000000000e+00	0.00000000000000e+00
1102	C0001102	SB -0.00000000000000e+00	NONE	NONE	0.00000000000000e+00	0.00000000000000e+00
1103	C0001103	SB -0.00000000000000e+00	NONE	NONE	0.00000000000000e+00	0.00000000000000e+00
1104	C0001104	SB -0.00000000000000e+00	NONE	NONE	0.00000000000000e+00	0.00000000000000e+00
1105	C0001105	SB -0.00000000000000e+00	NONE	NONE	0.00000000000000e+00	0.00000000000000e+00
1106	C0001106	SB -0.00000000000000e+00	NONE	NONE	0.00000000000000e+00	0.00000000000000e+00
1107	C0001107	SB -5.60022549878076e+10	NONE	NONE	0.00000000000000e+00	0.00000000000000e+00
1108	C0001108	SB -0.00000000000000e+00	NONE	NONE	0.00000000000000e+00	0.00000000000000e+00
1109	C0001109	SB -0.00000000000000e+00	NONE	NONE	0.00000000000000e+00	0.00000000000000e+00
1110	C0001110	SB 0.00000000000000e+00	0.00000000e+00	NONE	0.00000000000000e+00	0.00000000000000e+00
1111	C0001111	SB -0.00000000000000e+00	0.00000000e+00	NONE	0.00000000000000e+00	0.00000000000000e+00
1112	C0001112	SB -0.00000000000000e+00	NONE	NONE	0.00000000000000e+00	0.00000000000000e+00
1113	C0001113	SB 0.00000000000000e+00	NONE	NONE	0.00000000000000e+00	0.00000000000000e+00
1114	C0001114	LL 0.00000000000000e+00	0.00000000e+00	NONE	0.00000000000000e+00	0.00000000000000e+00
1115	C0001115	SB -0.00000000000000e+00	NONE	NONE	0.00000000000000e+00	0.00000000000000e+00
1116	C0001116	SB 8.06662658256757e+09	0.00000000e+00	NONE	0.00000000000000e+00	0.00000000000000e+00
1117	C0001117	SB -0.00000000000000e+00	NONE	NONE	0.00000000000000e+00	0.00000000000000e+00
1118	C0001118	SB 0.00000000000000e+00	NONE	NONE	0.00000000000000e+00	0.00000000000000e+00
1119	C0001119	SB 0.00000000000000e+00	NONE	NONE	0.00000000000000e+00	0.00000000000000e+00
1120	C0001120	SB 4.79356284052401e+10	NONE	NONE	0.00000000000000e+00	0.00000000000000e+00
1121	C0001121	LL 0.00000000000000e+00	0.00000000e+00	NONE	0.00000000000000e+00	0.00000000000000e+00
1122	C0001122	SB -1.22678687285069e+10	NONE	NONE	0.00000000000000e+00	0.00000000000000e+00
1123	C0001123	SB -0.00000000000000e+00	NONE	NONE	0.00000000000000e+00	0.00000000000000e+00
1124	C0001124	SB 0.00000000000000e+00	NONE	NONE	0.00000000000000e+00	0.00000000000000e+00
1125	C0001125	SB 0.00000000000000e+00	NONE	NONE	0.00000000000000e+00	0.00000000000000e+00
1126	C0001126	SB 0.00000000000000e+00	NONE	NONE	0.00000000000000e+00	0.00000000000000e+00
1127	C0001127	SB 0.00000000000000e+00	NONE	NONE	0.00000000000000e+00	0.00000000000000e+00
1128	C0001128	SB 0.00000000000000e+00	NONE	NONE	0.00000000000000e+00	0.00000000000000e+00
1129	C0001129	SB 0.00000000000000e+00	NONE	NONE	0.00000000000000e+00	0.00000000000000e+00
1130	C0001130	SB 0.00000000000000e+00	NONE	NONE	0.00000000000000e+00	0.00000000000000e+00
1131	C0001131	SB 0.00000000000000e+00	NONE	NONE	0.00000000000000e+00	0.00000000000000e+00
1132	C0001132	SB 0.00000000000000e+00	NONE	NONE	0.00000000000000e+00	0.00000000000000e+00
1133	C0001133	SB 0.00000000000000e+00	NONE	NONE	0.00000000000000e+00	0.00000000000000e+00
1134	C0001134	SB 0.00000000000000e+00	NONE	NONE	0.00000000000000e+00	0.00000000000000e+00
1135	C0001135	SB 0.00000000000000e+00	NONE	NONE	0.00000000000000e+00	0.00000000000000e+00
1136	C0001136	SB 6.39058046949904e-03	0.00000000e+00	NONE	0.00000000000000e+00	0.00000000000000e+00
1137	C0001137	SB 0.00000000000000e+00	NONE	NONE	0.00000000000000e+00	0.00000000000000e+00
1138	C0001138	LL 0.00000000000000e+00	0.00000000e+00	NONE	0.00000000000000e+00	0.00000000000000e+00
1139	C0001139	SB 0.00000000000000e+00	0.00000000e+00	NONE	0.00000000000000e+00	0.00000000000000e+00
1140	C0001140	SB 0.00000000000000e+00	0.00000000e+00	NONE	0.00000000000000e+00	0.00000000000000e+00
1141	C0001141	SB 0.00000000000000e+00	NONE	NONE	0.00000000000000e+00	0.00000000000000e+00
1142	C0001142	SB 0.00000000000000e+00	NONE	NONE	0.00000000000000e+00	0.00000000000000e+00
1143	C0001143	SB 0.00000000000000e+00	NONE	NONE	0.00000000000000e+00	0.00000000000000e+00
1144	C0001144	SB 0.00000000000000e+00	NONE	NONE	0.00000000000000e+00	0.00000000000000e+00
1145	C0001145	SB 0.00000000000000e+00	NONE	NONE	0.00000000000000e+00	0.00000000000000e+00
1146	C0001146	SB 0.00000000000000e+00	0.00000000e+00	NONE	0.00000000000000e+00	0.00000000000000e+00
1147	C0001147	LL 0.00000000000000e+00	0.00000000e+00	NONE	0.00000000000000e+00	0.00000000000000e+00
1148	C0001148	SB 0.00000000000000e+00	NONE	NONE	0.00000000000000e+00	0.00000000000000e+00
1149	C0001149	LL 0.00000000000000e+00	0.00000000e+00	NONE	0.00000000000000e+00	0.00000000000000e+00
1150	C0001150	LL 0.00000000000000e+00	0.00000000e+00	NONE	0.00000000000000e+00	0.00000000000000e+00
1151	C0001151	LL 0.00000000000000e+00	0.00000000e+00	NONE	0.00000000000000e+00	0.00000000000000e+00
1152	C0001152	LL 0.00000000000000e+00	0.00000000e+00	NONE	0.00000000000000e+00	0.00000000000000e+00
1153	C0001153	SB 9.61183072137312e+00	0.00000000e+00	NONE	0.00000000000000e+00	0.00000000000000e+00
1154	C0001154	SB 1.35530779969139e-01	0.00000000e+00	NONE	0.00000000000000e+00	0.00000000000000e+00
1155	C0001155	SB 0.00000000000000e+00	NONE	NONE	0.00000000000000e+00	0.00000000000000e+00
1156	C0001156	SB 0.00000000000000e+00	NONE	NONE	0.00000000000000e+00	0.00000000000000e+00
1157	C0001157	SB 0.00000000000000e+00	0.00000000e+00	NONE	0.00000000000000e+00	0.00000000000000e+00
1158	C0001158	SB 0.00000000000000e+00	0.00000000e+00	NONE	0.00000000000000e+00	0.00000000000000e+00
1159	C0001159	LL 0.00000000000000e+00	0.00000000e+00	NONE	0.00000000000000e+00	0.00000000000000e+00

1160	C0001160	LL 0.00000000000000e+00	0.00000000e+00	NONE	0.00000000000000e+00	0.00000000000000e+00
1161	C0001161	LL 0.00000000000000e+00	0.00000000e+00	NONE	0.00000000000000e+00	0.00000000000000e+00
1162	C0001162	SB 0.00000000000000e+00	0.00000000e+00	NONE	0.00000000000000e+00	0.00000000000000e+00
1163	C0001163	LL 0.00000000000000e+00	0.00000000e+00	NONE	0.00000000000000e+00	0.00000000000000e+00
1164	C0001164	LL 0.00000000000000e+00	0.00000000e+00	NONE	0.00000000000000e+00	0.00000000000000e+00
1165	C0001165	LL 0.00000000000000e+00	0.00000000e+00	NONE	0.00000000000000e+00	0.00000000000000e+00
1166	C0001166	SB 1.44081779589660e-02	NONE	NONE	0.00000000000000e+00	0.00000000000000e+00
1167	C0001167	SB 6.39058046949904e-03	0.00000000e+00	NONE	0.00000000000000e+00	0.00000000000000e+00
1168	C0001168	LL 0.00000000000000e+00	0.00000000e+00	NONE	0.00000000000000e+00	0.00000000000000e+00
1169	C0001169	LL 0.00000000000000e+00	0.00000000e+00	NONE	0.00000000000000e+00	0.00000000000000e+00
1170	C0001170	LL 0.00000000000000e+00	0.00000000e+00	NONE	0.00000000000000e+00	0.00000000000000e+00
1171	C0001171	LL 0.00000000000000e+00	0.00000000e+00	NONE	0.00000000000000e+00	0.00000000000000e+00
1172	C0001172	LL 0.00000000000000e+00	0.00000000e+00	NONE	0.00000000000000e+00	0.00000000000000e+00
1173	C0001173	SB 2.43467521377637e-01	0.00000000e+00	NONE	0.00000000000000e+00	0.00000000000000e+00
1174	C0001174	LL 0.00000000000000e+00	0.00000000e+00	NONE	0.00000000000000e+00	0.00000000000000e+00
1175	C0001175	LL 0.00000000000000e+00	0.00000000e+00	NONE	0.00000000000000e+00	0.00000000000000e+00
1176	C0001176	LL 0.00000000000000e+00	0.00000000e+00	NONE	0.00000000000000e+00	0.00000000000000e+00
1177	C0001177	SB -8.86510729266539e-03	NONE	NONE	0.00000000000000e+00	0.00000000000000e+00
1178	C0001178	LL 0.00000000000000e+00	0.00000000e+00	NONE	0.00000000000000e+00	0.00000000000000e+00
1179	C0001179	LL 0.00000000000000e+00	0.00000000e+00	NONE	0.00000000000000e+00	0.00000000000000e+00
1180	C0001180	LL 0.00000000000000e+00	0.00000000e+00	NONE	0.00000000000000e+00	0.00000000000000e+00
1181	C0001181	SB 0.00000000000000e+00	0.00000000e+00	NONE	0.00000000000000e+00	0.00000000000000e+00
1182	C0001182	LL 0.00000000000000e+00	0.00000000e+00	NONE	0.00000000000000e+00	0.00000000000000e+00
1183	C0001183	LL 0.00000000000000e+00	0.00000000e+00	NONE	0.00000000000000e+00	0.00000000000000e+00
1184	C0001184	SB 0.00000000000000e+00	0.00000000e+00	NONE	0.00000000000000e+00	0.00000000000000e+00
1185	C0001185	LL 0.00000000000000e+00	0.00000000e+00	NONE	0.00000000000000e+00	0.00000000000000e+00
1186	C0001186	LL 0.00000000000000e+00	0.00000000e+00	NONE	0.00000000000000e+00	0.00000000000000e+00
1187	C0001187	LL 0.00000000000000e+00	0.00000000e+00	NONE	0.00000000000000e+00	0.00000000000000e+00
1188	C0001188	LL 0.00000000000000e+00	0.00000000e+00	NONE	0.00000000000000e+00	0.00000000000000e+00
1189	C0001189	SB 0.00000000000000e+00	0.00000000e+00	NONE	0.00000000000000e+00	0.00000000000000e+00
1190	C0001190	LL 0.00000000000000e+00	0.00000000e+00	NONE	0.00000000000000e+00	0.00000000000000e+00
1191	C0001191	SB -6.39058046949904e-03	NONE	NONE	0.00000000000000e+00	0.00000000000000e+00
1192	C0001192	SB 0.00000000000000e+00	NONE	NONE	0.00000000000000e+00	0.00000000000000e+00
1193	C0001193	SB 0.00000000000000e+00	NONE	NONE	0.00000000000000e+00	0.00000000000000e+00
1194	C0001194	SB -1.05402848635147e+10	NONE	NONE	0.00000000000000e+00	0.00000000000000e+00
1195	C0001195	SB -3.49505567982539e+11	NONE	NONE	0.00000000000000e+00	0.00000000000000e+00
1196	C0001196	SB 0.00000000000000e+00	NONE	NONE	0.00000000000000e+00	0.00000000000000e+00
1197	C0001197	SB -8.64573808366372e+10	NONE	NONE	0.00000000000000e+00	0.00000000000000e+00
1198	C0001198	SB 1.47239793211233e+09	0.00000000e+00	NONE	7.03969365491667e-10	0.00000000000000e+00
1199	C0001199	SB 1.62319748065204e+09	0.00000000e+00	NONE	0.00000000000000e+00	0.00000000000000e+00
1200	C0001200	SB 2.79615745992000e+09	0.00000000e+00	NONE	3.74871207976530e-10	0.00000000000000e+00
1201	C0001201	SB 1.11086453283902e-01	0.00000000e+00	NONE	0.00000000000000e+00	0.00000000000000e+00
1202	C0001202	SB 3.13882183530324e+09	0.00000000e+00	NONE	0.00000000000000e+00	0.00000000000000e+00
1203	C0001203	SB -0.00000000000000e+00	0.00000000e+00	NONE	0.00000000000000e+00	0.00000000000000e+00
1204	C0001204	SB 2.34892352070190e+09	0.00000000e+00	NONE	0.00000000000000e+00	0.00000000000000e+00
1205	C0001205	SB 0.00000000000000e+00	NONE	NONE	0.00000000000000e+00	0.00000000000000e+00
1206	C0001206	SB -0.00000000000000e+00	0.00000000e+00	NONE	0.00000000000000e+00	0.00000000000000e+00
1207	C0001207	SB 0.00000000000000e+00	NONE	NONE	0.00000000000000e+00	0.00000000000000e+00
1208	C0001208	SB 1.05402848635147e+10	NONE	NONE	0.00000000000000e+00	0.00000000000000e+00
1209	C0001209	SB 0.00000000000000e+00	NONE	NONE	0.00000000000000e+00	0.00000000000000e+00
1210	C0001210	SB 1.12646180464762e-01	NONE	NONE	0.00000000000000e+00	0.00000000000000e+00
1211	C0001211	SB 8.86510729266539e-03	NONE	NONE	0.00000000000000e+00	0.00000000000000e+00
1212	C0001212	SB -1.35615394804650e-01	NONE	NONE	0.00000000000000e+00	0.00000000000000e+00
1213	C0001213	SB 0.00000000000000e+00	NONE	NONE	0.00000000000000e+00	0.00000000000000e+00
1214	C0001214	SB 0.00000000000000e+00	NONE	NONE	0.00000000000000e+00	0.00000000000000e+00
1215	C0001215	SB 0.00000000000000e+00	NONE	NONE	0.00000000000000e+00	0.00000000000000e+00
1216	C0001216	SB 0.00000000000000e+00	NONE	NONE	0.00000000000000e+00	0.00000000000000e+00
1217	C0001217	SB 0.00000000000000e+00	0.00000000e+00	NONE	0.00000000000000e+00	0.00000000000000e+00
1218	C0001218	SB 0.00000000000000e+00	0.00000000e+00	NONE	0.00000000000000e+00	0.00000000000000e+00
1219	C0001219	LL 0.00000000000000e+00	0.00000000e+00	NONE	0.00000000000000e+00	0.00000000000000e+00
1220	C0001220	SB 1.57676419749884e+10	NONE	NONE	0.00000000000000e+00	0.00000000000000e+00
1221	C0001221	SB -6.56015793901355e+10	NONE	NONE	0.00000000000000e+00	0.00000000000000e+00
1222	C0001222	SB 0.00000000000000e+00	0.00000000e+00	NONE	0.00000000000000e+00	0.00000000000000e+00
1223	C0001223	SB 1.42005975274149e-01	0.00000000e+00	NONE	0.00000000000000e+00	0.00000000000000e+00
1224	C0001224	LL 0.00000000000000e+00	0.00000000e+00	NONE	0.00000000000000e+00	0.00000000000000e+00
1225	C0001225	LL 0.00000000000000e+00	0.00000000e+00	NONE	0.00000000000000e+00	0.00000000000000e+00
1226	C0001226	LL 0.00000000000000e+00	0.00000000e+00	NONE	0.00000000000000e+00	0.00000000000000e+00
1227	C0001227	LL 0.00000000000000e+00	0.00000000e+00	NONE	0.00000000000000e+00	0.00000000000000e+00
1228	C0001228	SB -0.00000000000000e+00	0.00000000e+00	NONE	0.00000000000000e+00	0.00000000000000e+00
1229	C0001229	SB 0.00000000000000e+00	0.00000000e+00	NONE	0.00000000000000e+00	0.00000000000000e+00
1230	C0001230	SB 0.00000000000000e+00	0.00000000e+00	NONE	0.00000000000000e+00	0.00000000000000e+00
1231	C0001231	LL 0.00000000000000e+00	0.00000000e+00	NONE	0.00000000000000e+00	0.00000000000000e+00
1232	C0001232	SB 0.00000000000000e+00	0.00000000e+00	NONE	0.00000000000000e+00	0.00000000000000e+00
1233	C0001233	SB -0.00000000000000e+00	0.00000000e+00	NONE	0.00000000000000e+00	0.00000000000000e+00
1234	C0001234	SB -0.00000000000000e+00	0.00000000e+00	NONE	0.00000000000000e+00	0.00000000000000e+00
1235	C0001235	SB 0.00000000000000e+00	0.00000000e+00	NONE	0.00000000000000e+00	0.00000000000000e+00
1236	C0001236	SB 0.00000000000000e+00	0.00000000e+00	NONE	0.00000000000000e+00	0.00000000000000e+00
1237	C0001237	LL 0.00000000000000e+00	0.00000000e+00	NONE	0.00000000000000e+00	0.00000000000000e+00
1238	C0001238	LL 0.00000000000000e+00	0.00000000e+00	NONE	0.00000000000000e+00	0.00000000000000e+00
1239	C0001239	SB -0.00000000000000e+00	0.00000000e+00	NONE	0.00000000000000e+00	0.00000000000000e+00
1240	C0001240	LL 0.00000000000000e+00	0.00000000e+00	NONE	0.00000000000000e+00	0.00000000000000e+00
1241	C0001241	LL 0.00000000000000e+00	0.00000000e+00	NONE	0.00000000000000e+00	0.00000000000000e+00
1242	C0001242	LL 0.00000000000000e+00	0.00000000e+00	NONE	0.00000000000000e+00	0.00000000000000e+00
1243	C0001243	LL 0.00000000000000e+00	0.00000000e+00	NONE	0.00000000000000e+00	0.00000000000000e+00
1244	C0001244	LL 0.00000000000000e+00	0.00000000e+00	NONE	0.00000000000000e+00	0.00000000000000e+00
1245	C0001245	SB -0.00000000000000e+00	NONE	NONE	0.00000000000000e+00	0.00000000000000e+00
1246	C0001246	LL 0.00000000000000e+00	0.00000000e+00	NONE	0.00000000000000e+00	0.00000000000000e+00
1247	C0001247	SB -0.00000000000000e+00	0.00000000e+00	NONE	0.00000000000000e+00	0.00000000000000e+00
1248	C0001248	SB -0.00000000000000e+00	0.00000000e+00	NONE	0.00000000000000e+00	0.00000000000000e+00
1249	C0001249	SB 8.06662658256757e+09	NONE	NONE	0.00000000000000e+00	0.00000000000000e+00
1250	C0001250	SB -0.00000000000000e+00	NONE	NONE	0.00000000000000e+00	0.00000000000000e+00
1251	C0001251	SB 8.06662658256757e+09	NONE	NONE	0.00000000000000e+00	0.00000000000000e+00
1252	C0001252	LL 0.00000000000000e+00	0.00000000e+00	NONE	0.00000000000000e+00	0.00000000000000e+00
1253	C0001253	SB 0.00000000000000e+00	0.00000000e+00	NONE	0.00000000000000e+00	0.00000000000000e+00
1254	C0001254	SB 0.00000000000000e+00	0.00000000e+00	NONE	0.00000000000000e+00	0.00000000000000e+00
1255	C0001255	SB 0.00000000000000e+00	0.00000000e+00	NONE	0.00000000000000e+00	0.00000000000000e+00
1256	C0001256	LL 0.00000000000000e+00	0.00000000e+00	NONE	0.00000000000000e+00	0.00000000000000e+00
1257	C0001257	SB 0.00000000000000e+00	0.00000000e+00	NONE	0.00000000000000e+00	0.00000000000000e+00

1258	C0001258	SB -0.00000000000000e+00	0.00000000e+00	NONE	0.00000000000000e+00	0.00000000000000e+00
1259	C0001259	SB -0.00000000000000e+00	0.00000000e+00	NONE	0.00000000000000e+00	0.00000000000000e+00
1260	C0001260	SB -0.00000000000000e+00	0.00000000e+00	NONE	0.00000000000000e+00	0.00000000000000e+00
1261	C0001261	SB 0.00000000000000e+00	0.00000000e+00	NONE	0.00000000000000e+00	0.00000000000000e+00
1262	C0001262	LL 0.00000000000000e+00	0.00000000e+00	NONE	0.00000000000000e+00	0.00000000000000e+00
1263	C0001263	LL 0.00000000000000e+00	0.00000000e+00	NONE	0.00000000000000e+00	0.00000000000000e+00
1264	C0001264	SB 0.00000000000000e+00	0.00000000e+00	NONE	0.00000000000000e+00	0.00000000000000e+00
1265	C0001265	SB 0.00000000000000e+00	0.00000000e+00	NONE	0.00000000000000e+00	0.00000000000000e+00
1266	C0001266	SB 0.00000000000000e+00	NONE	NONE	0.00000000000000e+00	0.00000000000000e+00
1267	C0001267	SB -5.60022549878076e+10	NONE	NONE	0.00000000000000e+00	0.00000000000000e+00
1268	C0001268	LL 0.00000000000000e+00	0.00000000e+00	NONE	0.00000000000000e+00	0.00000000000000e+00
1269	C0001269	SB 0.00000000000000e+00	0.00000000e+00	NONE	0.00000000000000e+00	0.00000000000000e+00
1270	C0001270	SB 0.00000000000000e+00	0.00000000e+00	NONE	0.00000000000000e+00	0.00000000000000e+00
1271	C0001271	LL 0.00000000000000e+00	0.00000000e+00	NONE	0.00000000000000e+00	0.00000000000000e+00
1272	C0001272	LL 0.00000000000000e+00	0.00000000e+00	NONE	0.00000000000000e+00	0.00000000000000e+00
1273	C0001273	LL 0.00000000000000e+00	0.00000000e+00	NONE	0.00000000000000e+00	0.00000000000000e+00
1274	C0001274	LL 0.00000000000000e+00	0.00000000e+00	NONE	0.00000000000000e+00	0.00000000000000e+00
1275	C0001275	SB 0.00000000000000e+00	NONE	NONE	0.00000000000000e+00	0.00000000000000e+00
1276	C0001276	SB -0.00000000000000e+00	0.00000000e+00	NONE	0.00000000000000e+00	0.00000000000000e+00
1277	C0001277	SB -0.00000000000000e+00	0.00000000e+00	NONE	0.00000000000000e+00	0.00000000000000e+00
1278	C0001278	SB 0.00000000000000e+00	0.00000000e+00	NONE	0.00000000000000e+00	0.00000000000000e+00
1279	C0001279	SB 0.00000000000000e+00	0.00000000e+00	NONE	0.00000000000000e+00	0.00000000000000e+00
1280	C0001280	LL 0.00000000000000e+00	0.00000000e+00	NONE	0.00000000000000e+00	0.00000000000000e+00
1281	C0001281	LL 0.00000000000000e+00	0.00000000e+00	NONE	0.00000000000000e+00	0.00000000000000e+00
1282	C0001282	LL 0.00000000000000e+00	0.00000000e+00	NONE	0.00000000000000e+00	0.00000000000000e+00
1283	C0001283	SB -0.00000000000000e+00	0.00000000e+00	NONE	0.00000000000000e+00	0.00000000000000e+00
1284	C0001284	LL 0.00000000000000e+00	0.00000000e+00	NONE	0.00000000000000e+00	0.00000000000000e+00
1285	C0001285	SB 0.00000000000000e+00	NONE	NONE	0.00000000000000e+00	0.00000000000000e+00
1286	C0001286	SB 0.00000000000000e+00	0.00000000e+00	NONE	0.00000000000000e+00	0.00000000000000e+00
1287	C0001287	SB -0.00000000000000e+00	0.00000000e+00	NONE	0.00000000000000e+00	0.00000000000000e+00
1288	C0001288	SB -0.00000000000000e+00	0.00000000e+00	NONE	0.00000000000000e+00	0.00000000000000e+00
1289	C0001289	SB -0.00000000000000e+00	0.00000000e+00	NONE	0.00000000000000e+00	0.00000000000000e+00
1290	C0001290	LL 0.00000000000000e+00	0.00000000e+00	NONE	0.00000000000000e+00	0.00000000000000e+00
1291	C0001291	SB -0.00000000000000e+00	0.00000000e+00	NONE	0.00000000000000e+00	0.00000000000000e+00
1292	C0001292	SB 2.49769249969460e-12	0.00000000e+00	NONE	0.00000000000000e+00	0.00000000000000e+00
1293	C0001293	SB 2.49769249969460e-12	0.00000000e+00	NONE	0.00000000000000e+00	0.00000000000000e+00
1294	C0001294	LL 2.49769249969460e-12	0.00000000e+00	NONE	2.14748706463151e+10	0.00000000000000e+00
1295	C0001295	SB 0.00000000000000e+00	0.00000000e+00	NONE	0.00000000000000e+00	0.00000000000000e+00
1296	C0001296	SB 2.49769249969460e-12	0.00000000e+00	NONE	0.00000000000000e+00	0.00000000000000e+00
1297	C0001297	SB -0.00000000000000e+00	0.00000000e+00	NONE	0.00000000000000e+00	0.00000000000000e+00
1298	C0001298	SB 0.00000000000000e+00	0.00000000e+00	NONE	0.00000000000000e+00	0.00000000000000e+00
1299	C0001299	SB 2.42945548262619e-10	0.00000000e+00	NONE	0.00000000000000e+00	0.00000000000000e+00
1300	C0001300	SB 2.03645884315475e+09	NONE	NONE	0.00000000000000e+00	0.00000000000000e+00
1301	C0001301	SB 2.03645884315475e+09	NONE	NONE	0.00000000000000e+00	0.00000000000000e+00
1302	C0001302	SB -9.53674316406250e-07	NONE	NONE	0.00000000000000e+00	0.00000000000000e+00
1303	C0001303	SB 1.90734863281250e-06	NONE	NONE	0.00000000000000e+00	0.00000000000000e+00
1304	C0001304	SB 7.05428230530968e+07	NONE	NONE	0.00000000000000e+00	0.00000000000000e+00
1305	C0001305	SB 2.66231008434532e+07	NONE	NONE	0.00000000000000e+00	0.00000000000000e+00
1306	C0001306	SB 4.51014269059901e+09	NONE	NONE	0.00000000000000e+00	0.00000000000000e+00
1307	C0001307	SB -3.80680359152839e+09	NONE	NONE	0.00000000000000e+00	0.00000000000000e+00
1308	C0001308	SB 2.83696386612192e+09	NONE	NONE	0.00000000000000e+00	0.00000000000000e+00
1309	C0001309	SB 1.56003400387094e+09	0.00000000e+00	NONE	6.72429613899616e-10	0.00000000000000e+00
1310	C0001310	SB 1.57518001955659e+09	0.00000000e+00	NONE	7.74925186051787e-10	0.00000000000000e+00
1311	C0001311	SB 0.00000000000000e+00	0.00000000e+00	NONE	0.00000000000000e+00	0.00000000000000e+00
1312	C0001312	SB 0.00000000000000e+00	0.00000000e+00	NONE	0.00000000000000e+00	0.00000000000000e+00
1313	C0001313	SB -0.00000000000000e+00	0.00000000e+00	NONE	0.00000000000000e+00	0.00000000000000e+00
1314	C0001314	SB 5.61651238016001e+09	0.00000000e+00	NONE	0.00000000000000e+00	0.00000000000000e+00
1315	C0001315	SB 1.90109348583852e+09	0.00000000e+00	NONE	0.00000000000000e+00	0.00000000000000e+00
1316	C0001316	SB 2.05441826536973e+10	0.00000000e+00	NONE	0.00000000000000e+00	0.00000000000000e+00
1317	C0001317	SB 2.05441826536973e+10	0.00000000e+00	NONE	0.00000000000000e+00	0.00000000000000e+00
1318	C0001318	SB 2.05441826536973e+10	0.00000000e+00	NONE	0.00000000000000e+00	0.00000000000000e+00
1319	C0001319	SB 2.05441826536973e+10	0.00000000e+00	NONE	0.00000000000000e+00	0.00000000000000e+00
1320	C0001320	SB 3.04759790041274e+11	0.00000000e+00	NONE	0.00000000000000e+00	0.00000000000000e+00
1321	C0001321	SB 0.00000000000000e+00	0.00000000e+00	NONE	0.00000000000000e+00	0.00000000000000e+00
1322	C0001322	SB 0.00000000000000e+00	0.00000000e+00	NONE	0.00000000000000e+00	0.00000000000000e+00
1323	C0001323	SB 0.00000000000000e+00	NONE	NONE	0.00000000000000e+00	0.00000000000000e+00
1324	C0001324	SB -2.28785848116146e+10	NONE	NONE	0.00000000000000e+00	0.00000000000000e+00
1325	C0001325	SB 0.00000000000000e+00	NONE	NONE	0.00000000000000e+00	0.00000000000000e+00
1326	C0001326	SB 0.00000000000000e+00	0.00000000e+00	NONE	0.00000000000000e+00	0.00000000000000e+00
1327	C0001327	SB 0.00000000000000e+00	0.00000000e+00	NONE	0.00000000000000e+00	0.00000000000000e+00
1328	C0001328	LL -0.00000000000000e+00	0.00000000e+00	NONE	0.00000000000000e+00	0.00000000000000e+00
1329	C0001329	SB -0.00000000000000e+00	NONE	NONE	0.00000000000000e+00	0.00000000000000e+00
1330	C0001330	SB 0.00000000000000e+00	NONE	NONE	0.00000000000000e+00	0.00000000000000e+00
1331	C0001331	SB 2.77555756156289e-17	NONE	NONE	0.00000000000000e+00	0.00000000000000e+00
1332	C0001332	SB 0.00000000000000e+00	NONE	NONE	0.00000000000000e+00	0.00000000000000e+00
1333	C0001333	SB 0.00000000000000e+00	NONE	NONE	0.00000000000000e+00	0.00000000000000e+00
1334	C0001334	LL 0.00000000000000e+00	0.00000000e+00	NONE	0.00000000000000e+00	0.00000000000000e+00
1335	C0001335	LL 0.00000000000000e+00	0.00000000e+00	NONE	0.00000000000000e+00	0.00000000000000e+00
1336	C0001336	LL 0.00000000000000e+00	0.00000000e+00	NONE	0.00000000000000e+00	0.00000000000000e+00
1337	C0001337	LL 0.00000000000000e+00	0.00000000e+00	NONE	0.00000000000000e+00	0.00000000000000e+00
1338	C0001338	LL 0.00000000000000e+00	0.00000000e+00	NONE	0.00000000000000e+00	0.00000000000000e+00
1339	C0001339	LL 0.00000000000000e+00	0.00000000e+00	NONE	0.00000000000000e+00	0.00000000000000e+00
1340	C0001340	SB 1.35615394804650e-01	0.00000000e+00	NONE	0.00000000000000e+00	0.00000000000000e+00
1341	C0001341	SB 2.43467521377637e-01	0.00000000e+00	NONE	0.00000000000000e+00	0.00000000000000e+00
1342	C0001342	LL 0.00000000000000e+00	0.00000000e+00	NONE	0.00000000000000e+00	0.00000000000000e+00
1343	C0001343	SB 0.00000000000000e+00	0.00000000e+00	NONE	0.00000000000000e+00	0.00000000000000e+00
1344	C0001344	SB 2.42945548262619e-10	0.00000000e+00	NONE	0.00000000000000e+00	0.00000000000000e+00
1345	C0001345	SB 0.00000000000000e+00	NONE	NONE	0.00000000000000e+00	0.00000000000000e+00
1346	C0001346	SB 0.00000000000000e+00	NONE	NONE	0.00000000000000e+00	0.00000000000000e+00
1347	C0001347	SB 0.00000000000000e+00	NONE	NONE	0.00000000000000e+00	0.00000000000000e+00
1348	C0001348	SB 0.00000000000000e+00	NONE	NONE	0.00000000000000e+00	0.00000000000000e+00
1349	C0001349	SB 0.00000000000000e+00	NONE	NONE	0.00000000000000e+00	0.00000000000000e+00
1350	C0001350	SB 0.00000000000000e+00	NONE	NONE	0.00000000000000e+00	0.00000000000000e+00
1351	C0001351	SB 0.00000000000000e+00	NONE	NONE	0.00000000000000e+00	0.00000000000000e+00
1352	C0001352	LL 0.00000000000000e+00	0.00000000e+00	NONE	0.00000000000000e+00	0.00000000000000e+00
1353	C0001353	SB 0.00000000000000e+00	NONE	NONE	0.00000000000000e+00	0.00000000000000e+00
1354	C0001354	LL 0.00000000000000e+00	0.00000000e+00	NONE	0.00000000000000e+00	0.00000000000000e+00
1355	C0001355	LL 0.00000000000000e+00	0.00000000e+00	NONE	0.00000000000000e+00	0.00000000000000e+00

1356	C0001356	LL	0.00000000000000e+00	0.00000000e+00	NONE	0.00000000000000e+00	0.00000000000000e+00
1357	C0001357	SB	0.00000000000000e+00	0.00000000e+00	NONE	0.00000000000000e+00	0.00000000000000e+00
1358	C0001358	SB	0.00000000000000e+00	0.00000000e+00	NONE	0.00000000000000e+00	0.00000000000000e+00
1359	C0001359	SB	6.53392876399171e+09	0.00000000e+00	NONE	0.00000000000000e+00	0.00000000000000e+00
1360	C0001360	SB	0.00000000000000e+00	0.00000000e+00	NONE	0.00000000000000e+00	0.00000000000000e+00
1361	C0001361	SB	0.00000000000000e+00	0.00000000e+00	NONE	0.00000000000000e+00	0.00000000000000e+00
1362	C0001362	SB	3.04759790041274e+11	0.00000000e+00	NONE	0.00000000000000e+00	0.00000000000000e+00
1363	C0001363	SB	-0.00000000000000e+00	0.00000000e+00	NONE	0.00000000000000e+00	0.00000000000000e+00
1364	C0001364	LL	0.00000000000000e+00	0.00000000e+00	NONE	0.00000000000000e+00	0.00000000000000e+00
1365	C0001365	LL	0.00000000000000e+00	0.00000000e+00	NONE	0.00000000000000e+00	0.00000000000000e+00
1366	C0001366	SB	-0.00000000000000e+00	NONE	NONE	0.00000000000000e+00	0.00000000000000e+00
1367	C0001367	SB	-0.00000000000000e+00	NONE	NONE	0.00000000000000e+00	0.00000000000000e+00
1368	C0001368	SB	-0.00000000000000e+00	NONE	NONE	0.00000000000000e+00	0.00000000000000e+00
1369	C0001369	SB	-0.00000000000000e+00	NONE	NONE	0.00000000000000e+00	0.00000000000000e+00
1370	C0001370	LL	0.00000000000000e+00	0.00000000e+00	NONE	0.00000000000000e+00	0.00000000000000e+00
1371	C0001371	LL	0.00000000000000e+00	0.00000000e+00	NONE	0.00000000000000e+00	0.00000000000000e+00
1372	C0001372	SB	0.00000000000000e+00	0.00000000e+00	NONE	0.00000000000000e+00	0.00000000000000e+00
1373	C0001373	SB	2.05441826536964e+10	0.00000000e+00	NONE	0.00000000000000e+00	0.00000000000000e+00
1374	C0001374	SB	-0.00000000000000e+00	0.00000000e+00	NONE	0.00000000000000e+00	0.00000000000000e+00
1375	C0001375	SB	4.15628453506919e+11	NONE	NONE	0.00000000000000e+00	0.00000000000000e+00
1376	C0001376	SB	0.00000000000000e+00	0.00000000e+00	NONE	0.00000000000000e+00	0.00000000000000e+00
1377	C0001377	SB	0.00000000000000e+00	0.00000000e+00	NONE	0.00000000000000e+00	0.00000000000000e+00
1378	C0001378	SB	2.49769249969460e-12	0.00000000e+00	NONE	0.00000000000000e+00	0.00000000000000e+00
1379	C0001379	LL	2.42945548262619e-10	0.00000000e+00	NONE	4.08654947594839e+09	0.00000000000000e+00
1380	C0001380	LL	0.00000000000000e+00	0.00000000e+00	NONE	2.27438221000446e+05	0.00000000000000e+00
1381	C0001381	LL	0.00000000000000e+00	0.00000000e+00	NONE	2.27438221000446e+05	0.00000000000000e+00
1382	C0001382	SB	-0.00000000000000e+00	0.00000000e+00	NONE	0.00000000000000e+00	0.00000000000000e+00
1383	C0001383	SB	0.00000000000000e+00	0.00000000e+00	NONE	0.00000000000000e+00	0.00000000000000e+00
1384	C0001384	LL	0.00000000000000e+00	0.00000000e+00	NONE	0.00000000000000e+00	0.00000000000000e+00
1385	C0001385	SB	-0.00000000000000e+00	NONE	NONE	0.00000000000000e+00	0.00000000000000e+00
1386	C0001386	LL	0.00000000000000e+00	0.00000000e+00	NONE	0.00000000000000e+00	0.00000000000000e+00
1387	C0001387	SB	-0.00000000000000e+00	NONE	NONE	0.00000000000000e+00	0.00000000000000e+00
1388	C0001388	LL	0.00000000000000e+00	0.00000000e+00	NONE	0.00000000000000e+00	0.00000000000000e+00
1389	C0001389	LL	0.00000000000000e+00	0.00000000e+00	NONE	0.00000000000000e+00	0.00000000000000e+00
1390	C0001390	LL	0.00000000000000e+00	0.00000000e+00	NONE	0.00000000000000e+00	0.00000000000000e+00
1391	C0001391	SB	0.00000000000000e+00	NONE	NONE	0.00000000000000e+00	0.00000000000000e+00
1392	C0001392	LL	0.00000000000000e+00	0.00000000e+00	NONE	0.00000000000000e+00	0.00000000000000e+00
1393	C0001393	SB	-0.00000000000000e+00	0.00000000e+00	NONE	0.00000000000000e+00	0.00000000000000e+00
1394	C0001394	SB	0.00000000000000e+00	0.00000000e+00	NONE	0.00000000000000e+00	0.00000000000000e+00
1395	C0001395	SB	0.00000000000000e+00	NONE	NONE	0.00000000000000e+00	0.00000000000000e+00
1396	C0001396	LL	0.00000000000000e+00	0.00000000e+00	NONE	0.00000000000000e+00	0.00000000000000e+00
1397	C0001397	SB	0.00000000000000e+00	0.00000000e+00	NONE	0.00000000000000e+00	0.00000000000000e+00
1398	C0001398	SB	0.00000000000000e+00	NONE	NONE	0.00000000000000e+00	0.00000000000000e+00
1399	C0001399	LL	0.00000000000000e+00	0.00000000e+00	NONE	0.00000000000000e+00	0.00000000000000e+00
1400	C0001400	LL	0.00000000000000e+00	0.00000000e+00	NONE	4.08654947594839e+09	0.00000000000000e+00
1401	C0001401	LL	0.00000000000000e+00	0.00000000e+00	NONE	0.00000000000000e+00	0.00000000000000e+00
1402	C0001402	LL	0.00000000000000e+00	0.00000000e+00	NONE	0.00000000000000e+00	0.00000000000000e+00
1403	C0001403	SB	0.00000000000000e+00	0.00000000e+00	NONE	0.00000000000000e+00	0.00000000000000e+00
1404	C0001404	SB	-0.00000000000000e+00	NONE	NONE	0.00000000000000e+00	0.00000000000000e+00
1405	C0001405	LL	0.00000000000000e+00	0.00000000e+00	NONE	0.00000000000000e+00	0.00000000000000e+00
1406	C0001406	SB	0.00000000000000e+00	NONE	NONE	0.00000000000000e+00	0.00000000000000e+00
1407	C0001407	SB	-0.00000000000000e+00	0.00000000e+00	NONE	0.00000000000000e+00	0.00000000000000e+00
1408	C0001408	SB	0.00000000000000e+00	0.00000000e+00	NONE	0.00000000000000e+00	0.00000000000000e+00
1409	C0001409	LL	0.00000000000000e+00	0.00000000e+00	NONE	0.00000000000000e+00	0.00000000000000e+00
1410	C0001410	SB	0.00000000000000e+00	NONE	NONE	0.00000000000000e+00	0.00000000000000e+00
1411	C0001411	LL	0.00000000000000e+00	0.00000000e+00	NONE	0.00000000000000e+00	0.00000000000000e+00
1412	C0001412	SB	0.00000000000000e+00	0.00000000e+00	NONE	0.00000000000000e+00	0.00000000000000e+00
1413	C0001413	SB	2.42945548262619e-10	0.00000000e+00	NONE	0.00000000000000e+00	0.00000000000000e+00
1414	C0001414	SB	0.00000000000000e+00	0.00000000e+00	NONE	0.00000000000000e+00	0.00000000000000e+00
1415	C0001415	LL	0.00000000000000e+00	0.00000000e+00	NONE	0.00000000000000e+00	0.00000000000000e+00
1416	C0001416	SB	-0.00000000000000e+00	NONE	NONE	0.00000000000000e+00	0.00000000000000e+00
1417	C0001417	SB	0.00000000000000e+00	0.00000000e+00	NONE	0.00000000000000e+00	0.00000000000000e+00
1418	C0001418	SB	-0.00000000000000e+00	0.00000000e+00	NONE	0.00000000000000e+00	0.00000000000000e+00
1419	C0001419	SB	3.80514872176114e+00	NONE	NONE	0.00000000000000e+00	0.00000000000000e+00
1420	C0001420	SB	1.44081779589660e-02	NONE	NONE	0.00000000000000e+00	0.00000000000000e+00
1421	C0001421	SB	0.00000000000000e+00	NONE	NONE	0.00000000000000e+00	0.00000000000000e+00
1422	C0001422	SB	0.00000000000000e+00	NONE	NONE	0.00000000000000e+00	0.00000000000000e+00
1423	C0001423	SB	0.00000000000000e+00	NONE	NONE	0.00000000000000e+00	0.00000000000000e+00
1424	C0001424	SB	-8.86510729266539e-03	NONE	NONE	0.00000000000000e+00	0.00000000000000e+00
1425	C0001425	SB	0.00000000000000e+00	NONE	NONE	0.00000000000000e+00	0.00000000000000e+00
1426	C0001426	SB	1.66781884895788e+11	NONE	NONE	0.00000000000000e+00	0.00000000000000e+00
1427	C0001427	SB	-0.00000000000000e+00	NONE	NONE	0.00000000000000e+00	0.00000000000000e+00
1428	C0001428	LL	0.00000000000000e+00	0.00000000e+00	NONE	0.00000000000000e+00	0.00000000000000e+00
1429	C0001429	SB	0.00000000000000e+00	0.00000000e+00	NONE	0.00000000000000e+00	0.00000000000000e+00
1430	C0001430	SB	0.00000000000000e+00	0.00000000e+00	NONE	0.00000000000000e+00	0.00000000000000e+00
1431	C0001431	SB	-0.00000000000000e+00	0.00000000e+00	NONE	0.00000000000000e+00	0.00000000000000e+00
1432	C0001432	SB	0.00000000000000e+00	0.00000000e+00	NONE	0.00000000000000e+00	0.00000000000000e+00
1433	C0001433	LL	0.00000000000000e+00	0.00000000e+00	NONE	0.00000000000000e+00	0.00000000000000e+00
1434	C0001434	LL	0.00000000000000e+00	0.00000000e+00	NONE	0.00000000000000e+00	0.00000000000000e+00
1435	C0001435	SB	-0.00000000000000e+00	0.00000000e+00	NONE	0.00000000000000e+00	0.00000000000000e+00
1436	C0001436	SB	-0.00000000000000e+00	0.00000000e+00	NONE	0.00000000000000e+00	0.00000000000000e+00
1437	C0001437	LL	0.00000000000000e+00	0.00000000e+00	NONE	0.00000000000000e+00	0.00000000000000e+00
1438	C0001438	SB	0.00000000000000e+00	NONE	NONE	0.00000000000000e+00	0.00000000000000e+00
1439	C0001439	SB	1.28318704507009e-04	0.00000000e+00	NONE	0.00000000000000e+00	0.00000000000000e+00
1440	C0001440	SB	1.05402848635147e+10	0.00000000e+00	NONE	0.00000000000000e+00	0.00000000000000e+00
1441	C0001441	SB	1.44200546058582e+11	0.00000000e+00	NONE	0.00000000000000e+00	0.00000000000000e+00
1442	C0001442	SB	0.00000000000000e+00	0.00000000e+00	NONE	0.00000000000000e+00	0.00000000000000e+00
1443	C0001443	SB	0.00000000000000e+00	0.00000000e+00	NONE	0.00000000000000e+00	0.00000000000000e+00
1444	C0001444	SB	0.00000000000000e+00	0.00000000e+00	NONE	0.00000000000000e+00	0.00000000000000e+00
1445	C0001445	SB	0.00000000000000e+00	0.00000000e+00	NONE	0.00000000000000e+00	0.00000000000000e+00
1446	C0001446	SB	0.00000000000000e+00	0.00000000e+00	NONE	0.00000000000000e+00	0.00000000000000e+00
1447	C0001447	LL	0.00000000000000e+00	0.00000000e+00	NONE	0.00000000000000e+00	0.00000000000000e+00
1448	C0001448	LL	0.00000000000000e+00	0.00000000e+00	NONE	0.00000000000000e+00	0.00000000000000e+00
1449	C0001449	SB	0.00000000000000e+00	0.00000000e+00	NONE	0.00000000000000e+00	0.00000000000000e+00
1450	C0001450	SB	2.65103628670302e-01	0.00000000e+00	NONE	0.00000000000000e+00	0.00000000000000e+00
1451	C0001451	SB	3.73407603944451e-01	0.00000000e+00	NONE	0.00000000000000e+00	0.00000000000000e+00
1452	C0001452	SB	1.09335965648625e+11	0.00000000e+00	NONE	0.00000000000000e+00	0.00000000000000e+00
1453	C0001453	SB	0.00000000000000e+00	0.00000000e+00	NONE	0.00000000000000e+00	0.00000000000000e+00

1454	C0001454	SB 0.00000000000000e+00	0.00000000e+00	NONE	0.00000000000000e+00	0.00000000000000e+00
1455	C0001455	LL 0.00000000000000e+00	0.00000000e+00	NONE	0.00000000000000e+00	0.00000000000000e+00
1456	C0001456	SB 0.00000000000000e+00	0.00000000e+00	NONE	0.00000000000000e+00	0.00000000000000e+00
1457	C0001457	SB 1.56003400387094e+09	0.00000000e+00	NONE	0.00000000000000e+00	0.00000000000000e+00
1458	C0001458	SB 1.57518001955659e+09	0.00000000e+00	NONE	0.00000000000000e+00	0.00000000000000e+00
1459	C0001459	SB 1.47239793211233e+09	0.00000000e+00	NONE	0.00000000000000e+00	0.00000000000000e+00
1460	C0001460	SB 1.62319748065204e+09	0.00000000e+00	NONE	0.00000000000000e+00	0.00000000000000e+00
1461	C0001461	SB 2.79615745992000e+09	0.00000000e+00	NONE	0.00000000000000e+00	0.00000000000000e+00
1462	C0001462	SB 3.13882183530324e+09	0.00000000e+00	NONE	0.00000000000000e+00	0.00000000000000e+00
1463	C0001463	SB 0.00000000000000e+00	0.00000000e+00	NONE	0.00000000000000e+00	0.00000000000000e+00
1464	C0001464	SB 2.34892352070190e+09	0.00000000e+00	NONE	0.00000000000000e+00	0.00000000000000e+00
1465	C0001465	SB 0.00000000000000e+00	0.00000000e+00	NONE	0.00000000000000e+00	0.00000000000000e+00
1466	C0001466	SB 0.00000000000000e+00	0.00000000e+00	NONE	0.00000000000000e+00	0.00000000000000e+00
1467	C0001467	SB 0.00000000000000e+00	0.00000000e+00	NONE	0.00000000000000e+00	0.00000000000000e+00
1468	C0001468	SB 6.63359725366489e-03	0.00000000e+00	NONE	0.00000000000000e+00	0.00000000000000e+00
1469	C0001469	SB 0.00000000000000e+00	0.00000000e+00	NONE	0.00000000000000e+00	0.00000000000000e+00
1470	C0001470	SB 0.00000000000000e+00	0.00000000e+00	NONE	0.00000000000000e+00	0.00000000000000e+00
1471	C0001471	SB 1.00939180221817e-01	0.00000000e+00	NONE	0.00000000000000e+00	0.00000000000000e+00
1472	C0001472	SB 2.43467521377637e-01	0.00000000e+00	NONE	0.00000000000000e+00	0.00000000000000e+00
1473	C0001473	SB 0.00000000000000e+00	0.00000000e+00	NONE	0.00000000000000e+00	0.00000000000000e+00
1474	C0001474	SB 0.00000000000000e+00	0.00000000e+00	NONE	0.00000000000000e+00	0.00000000000000e+00
1475	C0001475	SB 0.00000000000000e+00	0.00000000e+00	NONE	0.00000000000000e+00	0.00000000000000e+00
1476	C0001476	SB 0.00000000000000e+00	0.00000000e+00	NONE	0.00000000000000e+00	0.00000000000000e+00
1477	C0001477	SB 0.00000000000000e+00	0.00000000e+00	NONE	0.00000000000000e+00	0.00000000000000e+00
1478	C0001478	SB 6.39058046949904e-03	0.00000000e+00	NONE	0.00000000000000e+00	0.00000000000000e+00
1479	C0001479	LL 0.00000000000000e+00	0.00000000e+00	NONE	0.00000000000000e+00	0.00000000000000e+00
1480	C0001480	SB 8.86510729266539e-03	0.00000000e+00	NONE	0.00000000000000e+00	0.00000000000000e+00
1481	C0001481	SB 0.00000000000000e+00	0.00000000e+00	NONE	0.00000000000000e+00	0.00000000000000e+00
1482	C0001482	SB 0.00000000000000e+00	0.00000000e+00	NONE	0.00000000000000e+00	0.00000000000000e+00
1483	C0001483	SB 2.17320263216948e-04	0.00000000e+00	NONE	0.00000000000000e+00	0.00000000000000e+00
1484	C0001484	SB 2.96555002775001e-04	0.00000000e+00	NONE	0.00000000000000e+00	0.00000000000000e+00
1485	C0001485	LL 0.00000000000000e+00	0.00000000e+00	NONE	0.00000000000000e+00	0.00000000000000e+00
1486	C0001486	SB 1.54107863180578e-02	0.00000000e+00	NONE	0.00000000000000e+00	0.00000000000000e+00
1487	C0001487	SB 0.00000000000000e+00	0.00000000e+00	NONE	0.00000000000000e+00	0.00000000000000e+00
1488	C0001488	SB 2.57181988925586e-04	0.00000000e+00	NONE	0.00000000000000e+00	0.00000000000000e+00
1489	C0001489	SB 0.00000000000000e+00	0.00000000e+00	NONE	0.00000000000000e+00	0.00000000000000e+00
1490	C0001490	SB 0.00000000000000e+00	0.00000000e+00	NONE	0.00000000000000e+00	0.00000000000000e+00
1491	C0001491	LL 0.00000000000000e+00	0.00000000e+00	NONE	0.00000000000000e+00	0.00000000000000e+00
1492	C0001492	SB 0.00000000000000e+00	0.00000000e+00	NONE	0.00000000000000e+00	0.00000000000000e+00
1493	C0001493	SB 0.00000000000000e+00	0.00000000e+00	NONE	0.00000000000000e+00	0.00000000000000e+00
1494	C0001494	SB 3.04759790041274e+11	0.00000000e+00	NONE	0.00000000000000e+00	0.00000000000000e+00
1495	C0001495	SB 0.00000000000000e+00	0.00000000e+00	NONE	0.00000000000000e+00	0.00000000000000e+00
1496	C0001496	LL 0.00000000000000e+00	0.00000000e+00	NONE	0.00000000000000e+00	0.00000000000000e+00
1497	C0001497	LL 0.00000000000000e+00	0.00000000e+00	NONE	0.00000000000000e+00	0.00000000000000e+00
1498	C0001498	SB 0.00000000000000e+00	0.00000000e+00	NONE	0.00000000000000e+00	0.00000000000000e+00
1499	C0001499	SB 5.72810696151426e-01	0.00000000e+00	NONE	0.00000000000000e+00	0.00000000000000e+00
1500	C0001500	LL 0.00000000000000e+00	0.00000000e+00	NONE	0.00000000000000e+00	0.00000000000000e+00
1501	C0001501	SB 0.00000000000000e+00	0.00000000e+00	NONE	0.00000000000000e+00	0.00000000000000e+00
1502	C0001502	SB 0.00000000000000e+00	0.00000000e+00	NONE	0.00000000000000e+00	0.00000000000000e+00
1503	C0001503	SB 6.92008456840244e-01	0.00000000e+00	NONE	0.00000000000000e+00	0.00000000000000e+00
1504	C0001504	SB 1.11086453283902e-01	0.00000000e+00	NONE	0.00000000000000e+00	0.00000000000000e+00
1505	C0001505	SB 0.00000000000000e+00	0.00000000e+00	NONE	0.00000000000000e+00	0.00000000000000e+00
1506	C0001506	LL 0.00000000000000e+00	0.00000000e+00	NONE	0.00000000000000e+00	0.00000000000000e+00
1507	C0001507	SB 0.00000000000000e+00	0.00000000e+00	NONE	0.00000000000000e+00	0.00000000000000e+00
1508	C0001508	SB 2.05936741026975e+11	0.00000000e+00	NONE	0.00000000000000e+00	0.00000000000000e+00
1509	C0001509	SB 1.68727404444811e-01	0.00000000e+00	NONE	0.00000000000000e+00	0.00000000000000e+00
1510	C0001510	SB 0.00000000000000e+00	0.00000000e+00	NONE	0.00000000000000e+00	0.00000000000000e+00
1511	C0001511	SB 3.58892808889621e-01	0.00000000e+00	NONE	0.00000000000000e+00	0.00000000000000e+00
1512	C0001512	SB 0.00000000000000e+00	0.00000000e+00	NONE	0.00000000000000e+00	0.00000000000000e+00
1513	C0001513	LL 0.00000000000000e+00	0.00000000e+00	NONE	0.00000000000000e+00	0.00000000000000e+00
1514	C0001514	SB 0.00000000000000e+00	0.00000000e+00	NONE	0.00000000000000e+00	0.00000000000000e+00
1515	C0001515	SB 8.08390082205510e-05	0.00000000e+00	NONE	0.00000000000000e+00	0.00000000000000e+00
1516	C0001516	SB 5.23282052395434e-01	0.00000000e+00	NONE	0.00000000000000e+00	0.00000000000000e+00
1517	C0001517	SB 0.00000000000000e+00	0.00000000e+00	NONE	0.00000000000000e+00	0.00000000000000e+00
1518	C0001518	SB 0.00000000000000e+00	0.00000000e+00	NONE	0.00000000000000e+00	0.00000000000000e+00
1519	C0001519	SB 0.00000000000000e+00	0.00000000e+00	NONE	0.00000000000000e+00	0.00000000000000e+00
1520	C0001520	SB 0.00000000000000e+00	0.00000000e+00	NONE	0.00000000000000e+00	0.00000000000000e+00
1521	C0001521	SB 1.08086180219319e-01	0.00000000e+00	NONE	0.00000000000000e+00	0.00000000000000e+00
1522	C0001522	SB 0.00000000000000e+00	0.00000000e+00	NONE	0.00000000000000e+00	0.00000000000000e+00
1523	C0001523	SB 0.00000000000000e+00	0.00000000e+00	NONE	0.00000000000000e+00	0.00000000000000e+00
1524	C0001524	LL 0.00000000000000e+00	0.00000000e+00	NONE	0.00000000000000e+00	0.00000000000000e+00
1525	C0001525	LL 0.00000000000000e+00	0.00000000e+00	NONE	0.00000000000000e+00	0.00000000000000e+00
1526	C0001526	SB 0.00000000000000e+00	0.00000000e+00	NONE	0.00000000000000e+00	0.00000000000000e+00
1527	C0001527	LL 0.00000000000000e+00	0.00000000e+00	NONE	0.00000000000000e+00	0.00000000000000e+00
1528	C0001528	LL 0.00000000000000e+00	0.00000000e+00	NONE	0.00000000000000e+00	0.00000000000000e+00
1529	C0001529	SB 0.00000000000000e+00	0.00000000e+00	NONE	0.00000000000000e+00	0.00000000000000e+00
1530	C0001530	LL 0.00000000000000e+00	0.00000000e+00	NONE	0.00000000000000e+00	0.00000000000000e+00
1531	C0001531	SB 1.57676419749884e+10	0.00000000e+00	NONE	0.00000000000000e+00	0.00000000000000e+00
1532	C0001532	SB 0.00000000000000e+00	0.00000000e+00	NONE	0.00000000000000e+00	0.00000000000000e+00
1533	C0001533	LL 0.00000000000000e+00	0.00000000e+00	NONE	0.00000000000000e+00	0.00000000000000e+00
1534	C0001534	SB 0.00000000000000e+00	0.00000000e+00	NONE	0.00000000000000e+00	0.00000000000000e+00
1535	C0001535	SB 1.12646180464762e-01	0.00000000e+00	NONE	0.00000000000000e+00	0.00000000000000e+00
1536	C0001536	SB 0.00000000000000e+00	0.00000000e+00	NONE	0.00000000000000e+00	0.00000000000000e+00
1537	C0001537	SB 2.35746467731304e-01	0.00000000e+00	NONE	0.00000000000000e+00	0.00000000000000e+00
1538	C0001538	LL 0.00000000000000e+00	0.00000000e+00	NONE	0.00000000000000e+00	0.00000000000000e+00
1539	C0001539	SB 1.72305317596191e-01	0.00000000e+00	NONE	0.00000000000000e+00	0.00000000000000e+00
1540	C0001540	SB 2.49769249969460e-12	0.00000000e+00	NONE	0.00000000000000e+00	0.00000000000000e+00
1541	C0001541	SB 5.60022549880920e+10	0.00000000e+00	NONE	0.00000000000000e+00	0.00000000000000e+00
1542	C0001542	LL 0.00000000000000e+00	0.00000000e+00	NONE	0.00000000000000e+00	0.00000000000000e+00
1543	C0001543	SB 4.63564081912159e+11	0.00000000e+00	NONE	0.00000000000000e+00	0.00000000000000e+00
1544	C0001544	SB 0.00000000000000e+00	0.00000000e+00	NONE	0.00000000000000e+00	0.00000000000000e+00
1545	C0001545	SB 9.61183072137312e+00	0.00000000e+00	NONE	0.00000000000000e+00	0.00000000000000e+00
1546	C0001546	LL 0.00000000000000e+00	0.00000000e+00	NONE	0.00000000000000e+00	0.00000000000000e+00
1547	C0001547	SB 0.00000000000000e+00	0.00000000e+00	NONE	0.00000000000000e+00	0.00000000000000e+00
1548	C0001548	SB 5.53892196433869e-01	0.00000000e+00	NONE	0.00000000000000e+00	0.00000000000000e+00
1549	C0001549	SB 4.63905364633269e-02	0.00000000e+00	NONE	0.00000000000000e+00	0.00000000000000e+00
1550	C0001550	LL 0.00000000000000e+00	0.00000000e+00	NONE	0.00000000000000e+00	0.00000000000000e+00
1551	C0001551	SB 0.00000000000000e+00	0.00000000e+00	NONE	0.00000000000000e+00	0.00000000000000e+00

1552	C0001552	SB 0.00000000000000e+00	0.00000000e+00	NONE	0.00000000000000e+00	0.00000000000000e+00
1553	C0001553	SB 0.00000000000000e+00	0.00000000e+00	NONE	0.00000000000000e+00	0.00000000000000e+00
1554	C0001554	SB 0.00000000000000e+00	0.00000000e+00	NONE	0.00000000000000e+00	0.00000000000000e+00
1555	C0001555	SB 0.00000000000000e+00	0.00000000e+00	NONE	0.00000000000000e+00	0.00000000000000e+00
1556	C0001556	SB 4.07654291706615e-01	0.00000000e+00	NONE	0.00000000000000e+00	0.00000000000000e+00
1557	C0001557	SB 0.00000000000000e+00	0.00000000e+00	NONE	0.00000000000000e+00	0.00000000000000e+00
1558	C0001558	SB 1.35530779969139e-01	0.00000000e+00	NONE	0.00000000000000e+00	0.00000000000000e+00
1559	C0001559	SB 6.49467510486577e-02	0.00000000e+00	NONE	0.00000000000000e+00	0.00000000000000e+00
1560	C0001560	SB 0.00000000000000e+00	0.00000000e+00	NONE	0.00000000000000e+00	0.00000000000000e+00
1561	C0001561	SB 0.00000000000000e+00	0.00000000e+00	NONE	0.00000000000000e+00	0.00000000000000e+00
1562	C0001562	SB 0.00000000000000e+00	0.00000000e+00	NONE	0.00000000000000e+00	0.00000000000000e+00
1563	C0001563	SB 0.00000000000000e+00	0.00000000e+00	NONE	0.00000000000000e+00	0.00000000000000e+00
1564	C0001564	SB 0.00000000000000e+00	0.00000000e+00	NONE	0.00000000000000e+00	0.00000000000000e+00
1565	C0001565	SB 0.00000000000000e+00	0.00000000e+00	NONE	0.00000000000000e+00	0.00000000000000e+00
1566	C0001566	SB 0.00000000000000e+00	0.00000000e+00	NONE	0.00000000000000e+00	0.00000000000000e+00
1567	C0001567	SB 0.00000000000000e+00	0.00000000e+00	NONE	0.00000000000000e+00	0.00000000000000e+00
1568	C0001568	LL 0.00000000000000e+00	0.00000000e+00	NONE	0.00000000000000e+00	0.00000000000000e+00
1569	C0001569	SB 3.47929023474919e-05	0.00000000e+00	NONE	0.00000000000000e+00	0.00000000000000e+00
1570	C0001570	SB -0.00000000000000e+00	0.00000000e+00	NONE	0.00000000000000e+00	0.00000000000000e+00
1571	C0001571	SB -0.00000000000000e+00	0.00000000e+00	NONE	0.00000000000000e+00	0.00000000000000e+00
1572	C0001572	SB -0.00000000000000e+00	0.00000000e+00	NONE	0.00000000000000e+00	0.00000000000000e+00
1573	C0001573	LL 0.00000000000000e+00	0.00000000e+00	NONE	0.00000000000000e+00	0.00000000000000e+00
1574	C0001574	LL 0.00000000000000e+00	0.00000000e+00	NONE	0.00000000000000e+00	0.00000000000000e+00
1575	C0001575	SB -0.00000000000000e+00	0.00000000e+00	NONE	0.00000000000000e+00	0.00000000000000e+00
1576	C0001576	LL 0.00000000000000e+00	0.00000000e+00	NONE	0.00000000000000e+00	0.00000000000000e+00
1577	C0001577	SB 4.15628453506229e+11	0.00000000e+00	NONE	0.00000000000000e+00	0.00000000000000e+00
1578	C0001578	SB -0.00000000000000e+00	0.00000000e+00	NONE	0.00000000000000e+00	0.00000000000000e+00
1579	C0001579	SB -0.00000000000000e+00	0.00000000e+00	NONE	0.00000000000000e+00	0.00000000000000e+00
1580	C0001580	LL 0.00000000000000e+00	0.00000000e+00	NONE	0.00000000000000e+00	0.00000000000000e+00
1581	C0001581	SB -0.00000000000000e+00	0.00000000e+00	NONE	0.00000000000000e+00	0.00000000000000e+00
1582	C0001582	SB -0.00000000000000e+00	0.00000000e+00	NONE	0.00000000000000e+00	0.00000000000000e+00
1583	C0001583	SB -0.00000000000000e+00	0.00000000e+00	NONE	0.00000000000000e+00	0.00000000000000e+00
1584	C0001584	LL 0.00000000000000e+00	0.00000000e+00	NONE	0.00000000000000e+00	0.00000000000000e+00
1585	C0001585	LL 0.00000000000000e+00	0.00000000e+00	NONE	0.00000000000000e+00	0.00000000000000e+00
1586	C0001586	SB 2.31952682316635e+00	0.00000000e+00	NONE	0.00000000000000e+00	0.00000000000000e+00
1587	C0001587	SB -0.00000000000000e+00	0.00000000e+00	NONE	0.00000000000000e+00	0.00000000000000e+00
1588	C0001588	LL 0.00000000000000e+00	0.00000000e+00	NONE	0.00000000000000e+00	0.00000000000000e+00
1589	C0001589	LL 0.00000000000000e+00	0.00000000e+00	NONE	0.00000000000000e+00	0.00000000000000e+00
1590	C0001590	LL 0.00000000000000e+00	0.00000000e+00	NONE	0.00000000000000e+00	0.00000000000000e+00
1591	C0001591	LL 0.00000000000000e+00	0.00000000e+00	NONE	0.00000000000000e+00	0.00000000000000e+00
1592	C0001592	LL 0.00000000000000e+00	0.00000000e+00	NONE	0.00000000000000e+00	0.00000000000000e+00
1593	C0001593	LL 0.00000000000000e+00	0.00000000e+00	NONE	0.00000000000000e+00	0.00000000000000e+00
1594	C0001594	LL 0.00000000000000e+00	0.00000000e+00	NONE	0.00000000000000e+00	0.00000000000000e+00
1595	C0001595	LL 0.00000000000000e+00	0.00000000e+00	NONE	0.00000000000000e+00	0.00000000000000e+00
1596	C0001596	LL 0.00000000000000e+00	0.00000000e+00	NONE	0.00000000000000e+00	0.00000000000000e+00
1597	C0001597	LL 0.00000000000000e+00	0.00000000e+00	NONE	0.00000000000000e+00	0.00000000000000e+00
1598	C0001598	SB -0.00000000000000e+00	0.00000000e+00	NONE	0.00000000000000e+00	0.00000000000000e+00
1599	C0001599	LL 0.00000000000000e+00	0.00000000e+00	NONE	0.00000000000000e+00	0.00000000000000e+00
1600	C0001600	SB -0.00000000000000e+00	0.00000000e+00	NONE	0.00000000000000e+00	0.00000000000000e+00
1601	C0001601	SB 6.56015793901355e+10	0.00000000e+00	NONE	0.00000000000000e+00	0.00000000000000e+00
1602	C0001602	SB -0.00000000000000e+00	0.00000000e+00	NONE	0.00000000000000e+00	0.00000000000000e+00
1603	C0001603	LL 0.00000000000000e+00	0.00000000e+00	NONE	0.00000000000000e+00	0.00000000000000e+00
1604	C0001604	LL 0.00000000000000e+00	0.00000000e+00	NONE	0.00000000000000e+00	0.00000000000000e+00
1605	C0001605	SB -0.00000000000000e+00	0.00000000e+00	NONE	0.00000000000000e+00	0.00000000000000e+00
1606	C0001606	LL 0.00000000000000e+00	0.00000000e+00	NONE	0.00000000000000e+00	0.00000000000000e+00
1607	C0001607	LL 0.00000000000000e+00	0.00000000e+00	NONE	0.00000000000000e+00	0.00000000000000e+00
1608	C0001608	LL 0.00000000000000e+00	0.00000000e+00	NONE	0.00000000000000e+00	0.00000000000000e+00
1609	C0001609	LL 0.00000000000000e+00	0.00000000e+00	NONE	0.00000000000000e+00	0.00000000000000e+00
1610	C0001610	SB -0.00000000000000e+00	0.00000000e+00	NONE	0.00000000000000e+00	0.00000000000000e+00
1611	C0001611	SB -0.00000000000000e+00	0.00000000e+00	NONE	0.00000000000000e+00	0.00000000000000e+00
1612	C0001612	LL 0.00000000000000e+00	0.00000000e+00	NONE	0.00000000000000e+00	0.00000000000000e+00
1613	C0001613	LL 0.00000000000000e+00	0.00000000e+00	NONE	0.00000000000000e+00	0.00000000000000e+00
1614	C0001614	SB -0.00000000000000e+00	0.00000000e+00	NONE	0.00000000000000e+00	0.00000000000000e+00
1615	C0001615	SB -0.00000000000000e+00	0.00000000e+00	NONE	0.00000000000000e+00	0.00000000000000e+00
1616	C0001616	SB -0.00000000000000e+00	0.00000000e+00	NONE	0.00000000000000e+00	0.00000000000000e+00
1617	C0001617	SB -0.00000000000000e+00	0.00000000e+00	NONE	0.00000000000000e+00	0.00000000000000e+00
1618	C0001618	SB 1.05402848635147e+10	0.00000000e+00	NONE	0.00000000000000e+00	0.00000000000000e+00
1619	C0001619	SB -0.00000000000000e+00	0.00000000e+00	NONE	0.00000000000000e+00	0.00000000000000e+00
1620	C0001620	SB -0.00000000000000e+00	0.00000000e+00	NONE	0.00000000000000e+00	0.00000000000000e+00
1621	C0001621	LL 0.00000000000000e+00	0.00000000e+00	NONE	0.00000000000000e+00	0.00000000000000e+00
1622	C0001622	SB -0.00000000000000e+00	0.00000000e+00	NONE	0.00000000000000e+00	0.00000000000000e+00
1623	C0001623	SB 8.64573808366372e+10	0.00000000e+00	NONE	0.00000000000000e+00	0.00000000000000e+00
1624	C0001624	SB -0.00000000000000e+00	0.00000000e+00	NONE	0.00000000000000e+00	0.00000000000000e+00
1625	C0001625	LL 0.00000000000000e+00	0.00000000e+00	NONE	0.00000000000000e+00	0.00000000000000e+00
1626	C0001626	LL 0.00000000000000e+00	0.00000000e+00	NONE	0.00000000000000e+00	0.00000000000000e+00
1627	C0001627	SB -0.00000000000000e+00	0.00000000e+00	NONE	0.00000000000000e+00	0.00000000000000e+00
1628	C0001628	SB -0.00000000000000e+00	0.00000000e+00	NONE	0.00000000000000e+00	0.00000000000000e+00
1629	C0001629	LL 0.00000000000000e+00	0.00000000e+00	NONE	0.00000000000000e+00	0.00000000000000e+00
1630	C0001630	SB -0.00000000000000e+00	0.00000000e+00	NONE	0.00000000000000e+00	0.00000000000000e+00
1631	C0001631	SB -0.00000000000000e+00	0.00000000e+00	NONE	0.00000000000000e+00	0.00000000000000e+00
1632	C0001632	SB 2.60684352750168e+10	0.00000000e+00	NONE	0.00000000000000e+00	0.00000000000000e+00
1633	C0001633	SB -0.00000000000000e+00	0.00000000e+00	NONE	0.00000000000000e+00	0.00000000000000e+00
1634	C0001634	SB -0.00000000000000e+00	0.00000000e+00	NONE	0.00000000000000e+00	0.00000000000000e+00
1635	C0001635	SB -0.00000000000000e+00	0.00000000e+00	NONE	0.00000000000000e+00	0.00000000000000e+00
1636	C0001636	SB 1.36837220879242e-04	0.00000000e+00	NONE	0.00000000000000e+00	0.00000000000000e+00
1637	C0001637	SB -0.00000000000000e+00	0.00000000e+00	NONE	0.00000000000000e+00	0.00000000000000e+00
1638	C0001638	LL 0.00000000000000e+00	0.00000000e+00	NONE	0.00000000000000e+00	0.00000000000000e+00
1639	C0001639	SB -0.00000000000000e+00	0.00000000e+00	NONE	0.00000000000000e+00	0.00000000000000e+00
1640	C0001640	SB -0.00000000000000e+00	0.00000000e+00	NONE	0.00000000000000e+00	0.00000000000000e+00
1641	C0001641	SB 6.39058046949904e-03	0.00000000e+00	NONE	0.00000000000000e+00	0.00000000000000e+00
1642	C0001642	SB -0.00000000000000e+00	0.00000000e+00	NONE	0.00000000000000e+00	0.00000000000000e+00
1643	C0001643	LL 0.00000000000000e+00	0.00000000e+00	NONE	0.00000000000000e+00	0.00000000000000e+00
1644	C0001644	SB -0.00000000000000e+00	0.00000000e+00	NONE	0.00000000000000e+00	0.00000000000000e+00
1645	C0001645	LL 0.00000000000000e+00	0.00000000e+00	NONE	0.00000000000000e+00	0.00000000000000e+00
1646	C0001646	SB -0.00000000000000e+00	0.00000000e+00	NONE	0.00000000000000e+00	0.00000000000000e+00
1647	C0001647	SB -0.00000000000000e+00	0.00000000e+00	NONE	0.00000000000000e+00	0.00000000000000e+00
1648	C0001648	SB 8.06662658205896e+09	0.00000000e+00	NONE	0.00000000000000e+00	0.00000000000000e+00
1649	C0001649	SB -0.00000000000000e+00	0.00000000e+00	NONE	0.00000000000000e+00	0.00000000000000e+00

1650	C0001650	LL	0.00000000000000e+00	0.00000000e+00	NONE	0.00000000000000e+00	0.00000000000000e+00
1651	C0001651	LL	0.00000000000000e+00	0.00000000e+00	NONE	0.00000000000000e+00	0.00000000000000e+00
1652	C0001652	SB	-0.00000000000000e+00	0.00000000e+00	NONE	0.00000000000000e+00	0.00000000000000e+00
1653	C0001653	LL	0.00000000000000e+00	0.00000000e+00	NONE	0.00000000000000e+00	0.00000000000000e+00
1654	C0001654	LL	0.00000000000000e+00	0.00000000e+00	NONE	0.00000000000000e+00	0.00000000000000e+00
1655	C0001655	SB	-0.00000000000000e+00	0.00000000e+00	NONE	0.00000000000000e+00	0.00000000000000e+00
1656	C0001656	SB	-0.00000000000000e+00	0.00000000e+00	NONE	0.00000000000000e+00	0.00000000000000e+00
1657	C0001657	LL	0.00000000000000e+00	0.00000000e+00	NONE	0.00000000000000e+00	0.00000000000000e+00
1658	C0001658	SB	-0.00000000000000e+00	0.00000000e+00	NONE	0.00000000000000e+00	0.00000000000000e+00
1659	C0001659	SB	-0.00000000000000e+00	0.00000000e+00	NONE	0.00000000000000e+00	0.00000000000000e+00
1660	C0001660	SB	1.35615394561704e-01	0.00000000e+00	NONE	0.00000000000000e+00	0.00000000000000e+00
1661	C0001661	SB	-0.00000000000000e+00	0.00000000e+00	NONE	0.00000000000000e+00	0.00000000000000e+00
1662	C0001662	SB	-0.00000000000000e+00	0.00000000e+00	NONE	0.00000000000000e+00	0.00000000000000e+00
1663	C0001663	LL	0.00000000000000e+00	0.00000000e+00	NONE	0.00000000000000e+00	0.00000000000000e+00
1664	C0001664	SB	-0.00000000000000e+00	0.00000000e+00	NONE	0.00000000000000e+00	0.00000000000000e+00
1665	C0001665	SB	-0.00000000000000e+00	0.00000000e+00	NONE	0.00000000000000e+00	0.00000000000000e+00
1666	C0001666	SB	-0.00000000000000e+00	0.00000000e+00	NONE	0.00000000000000e+00	0.00000000000000e+00
1667	C0001667	LL	0.00000000000000e+00	0.00000000e+00	NONE	0.00000000000000e+00	0.00000000000000e+00
1668	C0001668	SB	-0.00000000000000e+00	0.00000000e+00	NONE	0.00000000000000e+00	0.00000000000000e+00
1669	C0001669	SB	-0.00000000000000e+00	0.00000000e+00	NONE	0.00000000000000e+00	0.00000000000000e+00
1670	C0001670	SB	-0.00000000000000e+00	0.00000000e+00	NONE	0.00000000000000e+00	0.00000000000000e+00
1671	C0001671	SB	-0.00000000000000e+00	0.00000000e+00	NONE	0.00000000000000e+00	0.00000000000000e+00
1672	C0001672	SB	-0.00000000000000e+00	0.00000000e+00	NONE	0.00000000000000e+00	0.00000000000000e+00
1673	C0001673	SB	-0.00000000000000e+00	0.00000000e+00	NONE	0.00000000000000e+00	0.00000000000000e+00
1674	C0001674	SB	-0.00000000000000e+00	0.00000000e+00	NONE	0.00000000000000e+00	0.00000000000000e+00
1675	C0001675	LL	0.00000000000000e+00	0.00000000e+00	NONE	0.00000000000000e+00	0.00000000000000e+00
1676	C0001676	LL	0.00000000000000e+00	0.00000000e+00	NONE	0.00000000000000e+00	0.00000000000000e+00
1677	C0001677	SB	-0.00000000000000e+00	0.00000000e+00	NONE	0.00000000000000e+00	0.00000000000000e+00
1678	C0001678	LL	0.00000000000000e+00	0.00000000e+00	NONE	0.00000000000000e+00	0.00000000000000e+00
1679	C0001679	SB	-0.00000000000000e+00	0.00000000e+00	NONE	0.00000000000000e+00	0.00000000000000e+00
1680	C0001680	LL	0.00000000000000e+00	0.00000000e+00	NONE	0.00000000000000e+00	0.00000000000000e+00
1681	C0001681	SB	-0.00000000000000e+00	0.00000000e+00	NONE	0.00000000000000e+00	0.00000000000000e+00
1682	C0001682	LL	0.00000000000000e+00	0.00000000e+00	NONE	0.00000000000000e+00	0.00000000000000e+00
1683	C0001683	LL	0.00000000000000e+00	0.00000000e+00	NONE	0.00000000000000e+00	0.00000000000000e+00
1684	C0001684	SB	3.49505567982539e+11	0.00000000e+00	NONE	0.00000000000000e+00	0.00000000000000e+00
1685	C0001685	LL	0.00000000000000e+00	0.00000000e+00	NONE	0.00000000000000e+00	0.00000000000000e+00
1686	C0001686	LL	0.00000000000000e+00	0.00000000e+00	NONE	0.00000000000000e+00	0.00000000000000e+00
1687	C0001687	SB	-0.00000000000000e+00	0.00000000e+00	NONE	0.00000000000000e+00	0.00000000000000e+00
1688	C0001688	SB	-0.00000000000000e+00	0.00000000e+00	NONE	0.00000000000000e+00	0.00000000000000e+00
1689	C0001689	LL	0.00000000000000e+00	0.00000000e+00	NONE	0.00000000000000e+00	0.00000000000000e+00
1690	C0001690	SB	-0.00000000000000e+00	0.00000000e+00	NONE	0.00000000000000e+00	0.00000000000000e+00
1691	C0001691	SB	9.95869639116647e-01	0.00000000e+00	NONE	0.00000000000000e+00	0.00000000000000e+00
1692	C0001692	LL	0.00000000000000e+00	0.00000000e+00	NONE	0.00000000000000e+00	0.00000000000000e+00
1693	C0001693	SB	-0.00000000000000e+00	0.00000000e+00	NONE	0.00000000000000e+00	0.00000000000000e+00
1694	C0001694	LL	0.00000000000000e+00	0.00000000e+00	NONE	0.00000000000000e+00	0.00000000000000e+00
1695	C0001695	SB	1.22678687280035e+10	0.00000000e+00	NONE	0.00000000000000e+00	0.00000000000000e+00
1696	C0001696	LL	0.00000000000000e+00	0.00000000e+00	NONE	0.00000000000000e+00	0.00000000000000e+00
1697	C0001697	SB	-0.00000000000000e+00	0.00000000e+00	NONE	0.00000000000000e+00	0.00000000000000e+00
1698	C0001698	LL	0.00000000000000e+00	0.00000000e+00	NONE	0.00000000000000e+00	0.00000000000000e+00
1699	C0001699	SB	-0.00000000000000e+00	0.00000000e+00	NONE	0.00000000000000e+00	0.00000000000000e+00
1700	C0001700	SB	-0.00000000000000e+00	0.00000000e+00	NONE	0.00000000000000e+00	0.00000000000000e+00
1701	C0001701	LL	2.16204706413672e+00	0.00000000e+00	NONE	7.94073538028729e+00	0.00000000000000e+00
1702	C0001702	SB	1.00000000000000e+00	0.00000000e+00	NONE	0.00000000000000e+00	0.00000000000000e+00
1703	C0001703	LL	1.57479759029404e-01	0.00000000e+00	NONE	1.58614472178807e+02	0.00000000000000e+00
1704	C0001704	LL	1.31952682316635e+00	0.00000000e+00	NONE	3.62717496874674e+01	0.00000000000000e+00
1705	C0001705	SB	2.31952682316635e+00	0.00000000e+00	NONE	0.00000000000000e+00	0.00000000000000e+00

********** End .sol file ***************

Home
